# Views of democracy and society and support for political violence in the USA: findings from a nationally representative survey

**DOI:** 10.1186/s40621-023-00456-3

**Published:** 2023-09-29

**Authors:** Garen J. Wintemute, Sonia L. Robinson, Andrew Crawford, Daniel Tancredi, Julia P. Schleimer, Elizabeth A. Tomsich, Paul M. Reeping, Aaron B. Shev, Veronica A. Pear

**Affiliations:** 1 UC Davis Violence Prevention Research Program, Sacramento, CA USA; 2 Department of Emergency Medicine, UC Davis, Sacramento, CA USA; 3 California Firearm Violence Research Center, Sacramento, CA USA; 4 Department of Pediatrics, UC Davis, Sacramento, CA USA

**Keywords:** Political violence, Firearm violence, Violence and society, Racism, Domestic extremism, Civil war, QAnon

## Abstract

**Background:**

Current conditions in the USA suggest an increasing risk for political violence. Little is known about the prevalence of beliefs that might lead to political violence, about support for and personal willingness to engage in political violence, and about how those measures vary with individual characteristics, lethality of violence, political objectives that violence might advance, or specific populations as targets.

**Methods:**

This cross-sectional US nationally representative survey was conducted on May 13 to June 2, 2022, of adult members of the Ipsos KnowledgePanel. Outcomes are weighted, population-representative proportions of respondents endorsing selected beliefs about American democracy and society and violence to advance political objectives.

**Results:**

The analytic sample included 8620 respondents; 50.5% (95% confidence interval (CI) 49.3%, 51.7%) were female; and weighted mean (± standard deviation) age was 48.4 (± 18.0) years. Nearly 1 in 5 (18.9%, 95% CI 18.0%, 19.9%) agreed strongly or very strongly that “having a strong leader for America is more important than having a democracy”; 16.2% (95% CI 15.3%, 17.1%) agreed strongly or very strongly that “in America, native-born white people are being replaced by immigrants,” and 13.7% (95% CI 12.9%, 14.6%) agreed strongly or very strongly that “in the next few years, there will be civil war in the United States.” One-third of respondents (32.8%, 95% CI 31.7%, 33.9%) considered violence to be usually or always justified to advance at least 1 of 17 specific political objectives. Among all respondents, 7.7% (95% CI 7.0%, 8.4%) thought it very or extremely likely that within the next few years, in a situation where they believe political violence is justified, “I will be armed with a gun”; 1.1% (95% CI 0.9%, 1.4%) thought it very or extremely likely that “I will shoot someone with a gun.” Support for political violence and for the use of firearms in such violence frequently declined with increasing age, education, and income.

**Conclusions:**

Small but concerning proportions of the population consider violence, including lethal violence, to be usually or always justified to advance political objectives. Prevention efforts should proceed urgently based on the best evidence available.

**Supplementary Information:**

The online version contains supplementary material available at 10.1186/s40621-023-00456-3.

## Background

Recent events in the USA—mass shootings, violence, and threats of violence against elected and other government officials, the January 2021 assault on the Capitol, and others—have reminded Americans of the presence of violence in their nation’s public and political life. This study is motivated by 5 conditions that, in their apparent convergence (Wintemute 2021), create the potential for even greater violence that could put at risk the future of the USA as a free and democratic society.

First is a striking rise in violence, and particularly in firearm violence. The 28% increase in homicide from 2019 to 2020 (Centers for Disease Control and Prevention 2023) was the largest single-year percentage increase ever recorded. By 2021, firearms accounted for 63.7% of violent deaths in the USA: 80.5% of homicides (20,958 of 26,031) and 54.6% of suicides (26,328 of 48,183) (Centers for Disease Control and Prevention 2023).

Second is an equally unprecedented increase in firearm purchasing that began with the onset of the COVID-19 pandemic in January 2020 and, except for a brief respite late in 2021, has continued through July 2023 (Wintemute 2021; Federal Bureau of Investigation 2023). From January 2020 through July 2023, background checks on firearm purchasers have averaged 37.5% above expected levels (Additional file 1: Figure S1); an estimated 16.8 million excess background checks have been conducted, of 61.6 million checks altogether.

Third is uncertainty about the stability and value of democracy in the USA. Most Americans across the political spectrum now perceive a serious threat to democracy in the USA (NPR/PBS NewsHour/Marist National Poll 2021; Grinnell College National Poll 2021). At the same time, nearly 70% of adults—with very similar results for Democrats and Republicans—agree that “American democracy only serves the interests of the wealthy and powerful” (Cox 2021). Approximately 20% of Republicans, conservatives, and voters for Donald Trump (and 9% of Democrats, liberals, and voters for Joe Biden) disagree with the statement that “democracy is [the] best form of government” (The Economist/YouGov Poll 2021).

Fourth is the expansion into the mainstream of American public opinion of extreme, false beliefs about American society. Approximately 1 adult in 5 endorses the core elements of the QAnon belief complex: that the “government, media, and financial worlds in the US are controlled by a group of Satan-worshipping pedophiles” (16%) and that “there is a storm coming soon that will sweep away the elites in power and restore the rightful leaders” (22%) (Public Religion Research Institute 2022). Nearly 1 adult in 3 (32%) endorses the great replacement assertion that “a group of people in this country [is] trying to replace native-born Americans with immigrants.” (Associated Press-NORC Center for Public Affairs Research 2022).

Fifth is concerningly broad support for political violence: the use of physical force or violence to advance political objectives (Armed Conflict Location & Event Data Project 2019). More than a third (36%) of American adults (56% of Republicans and 22% of Democrats) agree that “the traditional American way of life is disappearing so fast that we may have to use force to save it” (Cox 2021). Nearly one-fifth of adults (18%) agree that “because things have gotten so far off track, true American patriots may have to resort to violence in order to save our country.” (Public Religion Research Institute 2022).

Research on the prevalence and determinants of support for political violence in the USA is sparse (Kleinfeld 2021; Kalmoe and Mason 2022; Bright Line Watch 2020, 2021; Westwood et al. 2022). Existing work has been criticized on multiple grounds, including failures to define violence, to determine whether support for political violence reflects support for violence generally, and to determine whether persons who endorse political violence are willing to engage in such violence themselves (Bright Line Watch 2021; Westwood et al. 2022).

Many important and urgent questions remain insufficiently explored, or unexplored altogether. Does support for political violence reflect a general predisposition to violence as a means of solving problems? How prevalent are support for, and willingness to engage in, political violence when that term is defined? How do those prevalences vary with individual sociodemographic characteristics, with specific political objectives for which violence might be employed, with the lethality of that violence, and with its target? What other individual characteristics (e.g., extreme political and social beliefs, firearm ownership) and community characteristics are associated with support for political violence? What specific preparations for political violence have its supporters made?

We conducted the 2022 Life in America survey to answer these and related questions with data from a large nationally representative sample, augmented by oversamples for populations of particular importance, and a series of papers is planned to cover specific topics of interest. This report outlines the study’s overall methods and presents descriptive tabulations of data from the main study sample on measures of respondents’ political and social beliefs, their support for and willingness to engage in political violence, and variation in those measures with respondents’ key sociodemographic characteristics.

## Methods

Data for this cross-sectional survey study are from the 2022 Life in America Survey, which was designed by the authors and administered online in English and Spanish from May 13 to June 2, 2022, by the survey research firm Ipsos (Ipsos 2023). Before participants accessed the questionnaire, they were provided informed consent language that concluded, “[by] continuing, you are agreeing to participate in this study.” The study is reported following American Association for Public Opinion Research guidelines (American Association for Public Opinion Research 2021).

### Participants

Respondents were drawn from the Ipsos KnowledgePanel, an online research panel that has been widely used in population-based research, including studies of violence and firearm ownership (Kravitz-Wirtz et al. 2021; Wintemute et al. 2022c; Schleimer et al. 2020; Miller et al. 2022; Miller and Azrael 2022; Salhi et al. 2019). To establish a nationally representative panel, members are recruited on an ongoing basis through address-based probability sampling using data from the US Postal Service’s Delivery Sequence File (Ipsos 2015). Recruited adults in households without internet access are provided a web-enabled device and free internet service, and a modest, primarily points-based incentive program seeks to encourage participation and promote participants’ retention in KnowledgePanel over time.

A probability-proportional-to-size procedure was used to select a study-specific sample. All panel members who were aged 18 years and older were eligible for selection. Invitations were sent by e-mail; automatic reminders were delivered to non-respondents by e-mail and telephone beginning 3 days later.

A final survey weight variable provided by Ipsos adjusted for the initial probability of selection into KnowledgePanel and for survey-specific non-response and over- or under-coverage using design weights with post-stratification raking ratio adjustments. With weighting, the sample is designed to be statistically representative of the non-institutionalized adult population of the USA as reflected in the 2021 March supplement of the Current Population Survey (Ipsos 2015).

### Measures

Sociodemographic data were collected by Ipsos from profiles created and maintained by KnowledgePanel members. Survey questions that supplied data for this analysis covered 3 broad domains: beliefs regarding democracy and the potential for violence in the USA; beliefs regarding American society and institutions; and support for and willingness to engage in violence, including political violence. Prior surveys on these topics were reviewed, and selected questions were included or adapted in this questionnaire to track trends in opinion and provide context for responses to questions that had not been asked previously.

Our primary outcome measures concerned political and non-political violence. Violence was represented by the phrase “force or violence,” defined in the questionnaire as “physical force strong enough that it could cause pain or injury to a person.” “Force or violence to advance an important political objective that you support” was used in questions about respondents’ support for and willingness to engage in political violence.

Respondents were asked about the extent to which they considered political violence to be justified “in general” and then about justification for its use to advance specified political objectives. Examples include “to return Donald Trump to the presidency this year,” “to preserve an American way of life based on Western European traditions,” and “to stop police violence” (see Additional file 1 and Tables 6, 7). There were 17 specified objectives. Nine were presented to all respondents, and 8 were paired, with each respondent seeing only 1 item from each pair; each respondent was presented with 13 of 17 objectives.

Respondents who considered political violence to be at least sometimes justified for at least 1 of these specified objectives were asked about their personal willingness to engage in political violence: by type of violence (to “damage property,” “threaten or intimidate a person,” “injure a person,” “kill a person”) and by target population (examples: “an elected federal or state government official,” “a police officer,” “a person who does not share your religion”) (see Additional file 1 and Tables 8, 9).

All respondents were asked about the likelihood of their future use of firearms in a situation where they consider political violence to be justified (e.g., “I will be armed with a gun,” “I will shoot someone with a gun”) (see Additional file 1 and Table 10).

The full text of all questions reported on here, including sources for questions from prior surveys, is in the Additional file 1.

### Implementation

Ipsos translated the questionnaire into Spanish, and interpreting services staff at UC Davis Medical Center reviewed the translation. Forty KnowledgePanel members participated in a pretest of the English language version that was administered April 27 to May 2, 2022.

Respondents were randomized 1:1 to receive response options in order from either negative to positive valence (e.g., from “do not agree” to “strongly agree”) or the reverse throughout the questionnaire. Where a question presented multiple statements for respondents to consider, the order in which those statements were presented was randomized unless ordering was necessary. Logic-driving questions (those to which responses might invoke a skip pattern) included non-response prompts.

**Fig. 1:**
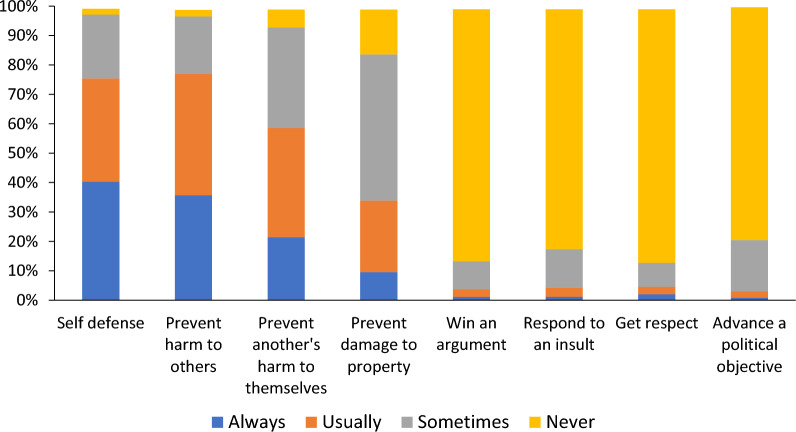
Justifiability of use or force or violence in specific situations. Respondents (*n* = 8620) were asked “What do you think about the use of force or violence in the following situations?” with response options always/usually/sometimes/never justified

To minimize inattentive responses, questions regarding political violence were immediately preceded by a question about the justifiability of the use of force or violence in 7 non-political situations. These situations were presented in a fixed order that, in the judgment of the authors, proceeded from more likely to less likely to be seen by respondents as justifying violence: from “in self-defense” to “to get respect” (see Additional file 1 and Fig. 1). This was done to create an expected response transition from support to nonsupport for violence that respondents would need to reverse to indicate support for political violence.

We employed unipolar response arrays without a neutral midpoint (e.g., do not agree, somewhat agree, strongly agree, very strongly agree). The literature is not in agreement on whether such midpoints should be included (Westwood et al. 2022; Chyung et al. 2017). We were persuaded by the studies reviewed by Chyung et al. (2017), which suggest that such midpoints allow respondents to choose “a minimally acceptable response as soon as it is found, instead of putting effort to find an optimal response,” a behavior known as satisficing. According to those authors, satisficing is particularly common when respondents are uncomfortable with the topics of the survey or under social desirability pressures, and both conditions apply here. Our analyses focus on responses above the “somewhat” or “sometimes” level to minimize the impact of potential satisficing on the results.

### Statistical analysis

To generate prevalence estimates, we calculated weighted percentages and 95% confidence intervals (CIs) using PROC SURVEYFREQ in SAS version 9.4 (SAS Institute, Inc., Cary, NC) and Complex Samples Frequencies in IBM SPSS Statistics, version 28 (IBM Corp., Armonk, NY). Estimated counts of adults in the USA were generated by simple extrapolation from the population-representative results, multiplying weighted percentages and their confidence intervals from our sample by the estimated adult population of the USA as of July 1, 2021 (258.33 million persons) (United States Census Bureau 2022).

We calculated prevalence estimates and their 95% CIs within categories of age, gender, race and ethnicity, education, income, and census region using the methods mentioned above. For dichotomous and ordinal variables, we generated Spearman correlations between demographic characteristics and response options.

## Results

Of 15,449 panel members invited to participate as part of the main study sample, 8620 completed the survey, yielding a 55.8% completion rate. The median survey completion time was 15.7 min (Interquartile Range, 11.4–23.0). Item non-response ranged from 0.3 to 2.3%.

Half of the respondents (50.5%, 95% CI 49.3%, 51.7%) were female; 62.6% (95% CI 61.4%, 63.9%) were white, non-Hispanic (Table 1). The weighted mean (SD) respondent age was 48.4 (18.0) years. Compared to non-respondents, respondents were older and more frequently white, non-Hispanic; were more often married; had higher education and income; and were less likely to be working (Table S1).

**Table 1:** Personal characteristics of respondents

Characteristic	Respondents (*n* = 8620)	
	Unweighted *n*	Weighted % (95% CI)
*Age*		
18–24	447	10.5 (9.6, 11.5)
25–34	1024	16.6 (15.6, 17.6)
35–44	1374	18.5 (17.6, 19.5)
45–54	1215	14.5 (13.7, 15.3)
55–64	1833	17.4 (16.6, 18.2)
65–74	1788	14.4 (13.7, 15.1)
75 +	939	8.0 (7.4, 8.5)
Non-response	0	0
*Gender*		
Female	4300	50.6 (49.4, 51.7)
Male	4159	47.2 (46.1, 48.4)
Transgender	41	0.6 (0.4, 0.8)
Non-binary	44	0.7 (0.5, 0.9)
Other	20	0.3 (0.1, 0.4)
Non-response	56	0.7 (0.5, 0.9)
*Race and ethnicity*		
White, Non-Hispanic	6046	62.6 (61.4, 63.9)
Black, Non-Hispanic	834	11.9 (11.1, 12.8)
Hispanic, any race	1084	16.9 (15.9, 17.8)
American Indian or Alaska Native, Non-Hispanic	54	1.3 (0.9, 1.6)
Asian American/Pacific Islander, non-Hispanic	313	5.4 (4.8, 6.1)
Some other race, Non-Hispanic	22	0.1 (0.1, 0.2)
2 + Races, Non-Hispanic	267	1.7 (1.5, 2.0)
Non-response	0	0
*Marital status*		
Now married	5246	56.1 (54.9, 57.3)
Widowed	443	4.0 (3.6, 4.4)
Divorced	909	8.7 (8.1, 9.3)
Separated	139	1.7 (1.4, 2.1)
Never married	1883	29.5 (28.3, 30.7)
Non-response	0	0
*Education*		
No high school diploma or GED	542	9.5 (8.7, 10.4)
High school graduate (diploma or GED)	2158	28.3 (27.2, 29.4)
Some college or Associate’s degree	2364	27.1 (26.0, 28.1)
Bachelor’s degree	1951	19.7 (18.8, 20.6)
Master’s degree or higher	1605	15.4 (14.7, 16.2)
Non-response	0	0
*Household income*		
Less than $10,000	272	3.9 (3.4, 4.4)
$10,000 to $24,999	745	9.0 (8.3, 9.7)
$25,000 to $49,999	1469	17.0 (16.1, 17.9)
$50,000 to $74,999	1414	16.3 (15.4, 17.2)
$75,000 to $99,999	1214	13.2 (12.4, 14)
$100,000 to $149,999	1500	17.9 (16.9, 18.8)
$150,000 or more	2006	22.8 (21.8, 23.7)
Non-response	0	0
*Employment*		
Working—as a paid employee	4323	54.3 (53.1, 55.4)
Working—self-employed	694	8.0 (7.3, 8.6)
Not working—on temporary layoff from a job	40	0.6 (0.4, 0.8)
Not working—looking for work	312	5.1 (4.5, 5.7)
Not working—retired	2478	20.9 (20.1, 21.8)
Not working—disabled	314	4.2 (3.7, 4.7)
Not working—other	459	7.0 (6.3, 7.7)
Non-response	0	0
*Census region*		
New England	412	4.7 (4.2, 5.2)
Mid-Atlantic	1090	12.5 (11.8, 13.3)
East-North Central	1267	14.3 (13.5, 15.1)
West-North Central	604	6.4 (5.8, 6.9)
South Atlantic	1714	20.5 (19.5, 21.4)
East-South Central	465	5.8 (5.3, 6.4)
West-South Central	904	12.0 (11.1, 12.8)
Mountain	745	7.7 (7.1, 8.2)
Pacific	1419	16.2 (15.3, 17.1)
Non-response	0	0

### Democracy and the potential for violence

Two-thirds of the respondents (67.2%, 95% CI 66.1%, 68.4%) perceived “a serious threat to our democracy,” and 88.9% (95% CI 88.0%, 89.7%) believed it is very or extremely important “for the United States to remain a democracy” (Table 2). At the same time, nearly 1 respondent in 5 (18.9%, 95% CI 18.0%, 19.9%) agreed strongly or very strongly that “having a strong leader for America is more important than having a democracy.” Separately, nearly 1 in 5 (18.4%, 95% CI 17.5%, 19.3%) agreed strongly or very strongly with the statement that “the 2020 election was stolen from Donald Trump, and Joe Biden is an illegitimate president.”

**Table 2:** Beliefs concerning democracy in the USA

Statement	Respondents (*n* = 8620)		Estimated *N* of adults in USA
	Unweighted *n*	Weighted % (95% CI)	*N* (95% CI) (in millions)
Do you believe that things in this country are…			
Generally headed in the wrong direction	7043	81.7 (80.8, 82.7)	211.2 (208.7, 213.6)
Generally headed in the right direction	1486	18.3 (17.3, 19.2)	47.2 (44.7, 49.6)
Non-response	91	1.2 (0.9, 1.4)	3.0 (2.3, 3.7)
When thinking about democracy in the USA these days, do you believe…			
There is a serious threat to our democracy	6117	67.2 (66.1, 68.4)	173.7 (170.7, 176.7)
There may be a threat to our democracy, but it is not serious	1832	23.6 (22.5, 24.6)	60.9 (58.2, 63.7)
There is no threat to our democracy	573	7.8 (7.1, 8.5)	20.1 (18.3, 21.9)
Non-response	98	1.4 (1.1, 1.7)	3.6 (2.8, 4.4)
How important do you think it is for the USA to remain a democracy?			
Not important	145	2.2 (1.8, 2.6)	5.6 (4.6, 6.6)
Somewhat important	510	7.8 (7.1, 8.5)	20.1 (18.3, 22.0)
Very important	1828	24.1 (23.1, 25.2)	62.4 (59.6, 65.1)
Extremely important	6058	64.7 (63.6, 65.9)	167.2 (164.2, 170.3)
Non-response	79	1.1 (0.9, 1.4)	2.9 (2.2, 3.7)
Democracy is the best form of government			
Do not agree	429	6.0 (5.4, 6.6)	15.6 (14.0, 17.2)
Somewhat agree	1757	23.5 (22.4, 24.5)	60.7 (57.9, 63.4)
Strongly agree	2678	31.3 (30.2, 32.4)	80.8 (78, 83.7)
Very strongly agree	3642	37.6 (36.5, 38.7)	97.2 (94.3, 100.1)
Non-response	114	1.6 (1.3, 2.0)	4.1 (3.3, 5.2)
These days, American democracy only serves the interest of the wealthy and powerful			
Do not agree	2566	26.6 (25.6, 27.6)	68.6 (66.0, 71.2)
Somewhat agree	3058	36.2 (35.0, 37.3)	93.4 (90.4, 96.4)
Strongly agree	1638	20.1 (19.1, 21.1)	51.9 (49.4, 54.4)
Very strongly agree	1260	15.9 (15.0, 16.8)	41.0 (38.7, 43.3)
Non-response	98	1.3 (.01, 1.6)	3.4 (2.7, 4.2)
Having a strong leader for America is more important than having a democracy			
Do not agree	5141	56.0 (54.8, 57.2)	144.7 (141.6, 147.8)
Somewhat agree	1835	23.4 (22.3, 24.4)	60.4 (57.7, 63.1)
Strongly agree	821	10.3 (9.5, 11.0)	26.5 (24.6, 28.5)
Very strongly agree	702	8.7 (8.0, 9.4)	22.4 (20.6, 24.2)
Non-response	121	1.7 (1.3, 2.0)	4.3 (3.5, 5.2)
The 2020 election was stolen from Donald Trump, and Joe Biden is an illegitimate president			
Do not agree	5761	66.2 (65.1, 67.4)	171.1 (168.2, 174)
Somewhat agree	1142	13.7 (12.9, 14.5)	35.4 (33.2, 37.6)
Strongly agree	498	5.9 (5.4, 6.5)	15.3 (13.9, 16.8)
Very strongly agree	1083	12.5 (11.7, 13.2)	32.2 (30.2, 34.2)
Non-response	136	1.7 (1.4, 2.0)	4.3 (3.5, 5.1)
Armed citizens should patrol polling places at election time			
Do not agree	7268	82.2 (81.3, 83.2)	212.4 (209.9, 214.9)
Somewhat agree	721	9.5 (8.8, 10.3)	24.7 (22.7, 26.6)
Strongly agree	271	3.6 (3.1, 4.1)	9.3 (8.0, 10.5)
Very strongly agree	226	2.9 (2.5, 3.4)	7.6 (6.5, 8.7)
Non-response	134	1.7 (1.4, 2.0)	4.4 (3.6, 5.2)

Substantial proportions of respondents agreed strongly or very strongly with each of 3 statements about potential conditions in the USA justifying force or violence (Table 3): to “protect American democracy” if “elected leaders will not” (18.7%, 95% CI 17.8%, 19.7%); to save “our American way of life,” which is “disappearing” (16.1%, 95% CI 15.2%, 17.0%); and to “save our country” (a job for “true American patriots”) because “things have gotten so far off track” (8.1%, 95% CI 7.5%, 8.8%). Approximately 1 respondent in 7 (13.7%, 95% CI 12.9%, 14.6%) agreed strongly or very strongly that “in the next few years, there will be civil war in the United States” (Table 3).

**Table 3:** Beliefs concerning the potential need for violence in the USA

Statement	Respondents (*n* = 8620)		Estimated *N* of adults in USA
	Unweighted *n*	Weighted % (95% CI)	*N* (95% CI) (in millions)
If elected leaders will not protect American democracy, the people must do it themselves, even if it requires taking violent actions			
Do not agree	4504	50.0 (48.9, 51.2)	129.3 (126.2, 132.4)
Somewhat agree	2468	29.6 (28.5, 30.7)	76.4 (73.6, 79.2)
Strongly agree	834	10.3 (9.6, 11.1)	26.6 (24.7, 28.6)
Very strongly agree	687	8.4 (7.7, 9.1)	21.7 (19.9, 23.5)
Non-response	127	1.7 (1.4, 2.0)	4.3 (3.5, 5.1)
Our American way of life is disappearing so fast that we may have to use force to save it			
Do not agree	4959	55.6 (54.4, 56.8)	143.7 (140.6, 146.8)
Somewhat agree	2222	26.7 (25.7, 27.8)	69.1 (66.3, 71.8)
Strongly agree	730	8.9 (8.2, 9.6)	23.0 (21.2, 24.9)
Very strongly agree	585	7.2 (6.5, 7.8)	18.5 (16.9, 20.2)
Non-response	124	1.5 (1.2, 1.8)	4.0 (3.2, 4.8)
Because things have gotten so far off track, true American patriots may have to resort to violence in order to save our country			
Do not agree	6404	72.4 (71.3, 73.5)	187 (184.2, 189.9)
Somewhat agree	1423	17.6 (16.6, 18.5)	45.4 (43.0, 47.8)
Strongly agree	369	4.4 (3.9, 4.9)	11.4 (10.1, 12.6)
Very strongly agree	279	3.7 (3.2, 4.2)	9.6 (8.3, 10.9)
Non-response	145	1.9 (1.6, 2.2)	4.9 (4.0, 5.8)
In the next few years, there will be civil war in the USA			
Do not agree	4268	47.8 (46.6, 48.9)	123.4 (120.3, 126.4)
Somewhat agree	3126	36.4 (35.3, 37.6)	94.1 (91.1, 97.0)
Strongly agree	654	8.4 (7.7, 9.1)	21.8 (20.0, 23.6)
Very strongly agree	411	5.3 (4.8, 5.9)	13.7 (12.3, 15.2)
Non-response	161	2.1 (1.7, 2.4)	5.4 (4.5, 6.3)

### American society and institutions

Five items explored beliefs on race and ethnicity and the great replacement assertion (Table 4). Nearly a third (31.8%, 95% CI, 30.7%, 32.9%) of respondents disagreed with the statement that “white people benefit from advantages in society that Black people do not have,” and 40.2% (95% CI, 39.0%, 41.3%) did not agree that “straight white men hold far too much power in America.” More than 1 in 4 (27.2%, 95% CI 26.1%, 28.2%) agreed strongly or very strongly that “discrimination against whites is as big a problem as discrimination against Blacks and other minorities.” Nearly 1 in 5 (18.6%, 95% CI 17.7%, 19.5%) disagreed with the statement that “having more Black Americans, Latinos, and Asian Americans is good for the country,” and 16.2% (95% CI 15.3%, 17.1%) agreed strongly or very strongly with the proposition that “in America, native-born white people are being replaced by immigrants.”

**Table 4:** Beliefs concerning race and ethnicity and American society

Statement	Respondents (*n* = 8620)		Estimated *N* of adults in USA
	Unweighted *n*	Weighted % (95% CI)	*N* (95% CI) (in millions)
White people benefit from advantages in society that Black people do not have			
Do not agree	2866	31.8 (30.7, 32.9)	82.2 (79.3, 85.0)
Somewhat agree	2443	27.9 (26.8, 29.0)	72.1 (69.3, 74.8)
Strongly agree	1414	17.0 (16.1, 17.9)	43.9 (41.5, 46.2)
Very strongly agree	1793	22.0 (21.0, 23.0)	56.8 (54.2, 59.4)
Non-response	104	1.3 (1.1, 1.6)	3.5 (2.7, 4.2)
Straight white men hold far too much power in America			
Do not agree	3679	40.2 (39.0, 41.3)	103.8 (100.8, 106.7)
Somewhat agree	2266	26.4 (25.4, 27.5)	68.3 (65.6, 71.1)
Strongly agree	1181	14.4 (13.6, 15.3)	37.2 (35.0, 39.4)
Very strongly agree	1348	17.2 (16.3, 18.1)	44.4 (42.0, 46.8)
Non-response	146	1.8 (1.5, 2.1)	4.6 (3.8, 5.5)
Discrimination against whites is as big a problem as discrimination against Blacks and other minorities			
Do not agree	4174	48.9 (47.7, 50.1)	126.3 (123.2, 129.3)
Somewhat agree	1986	22.7 (21.7, 23.7)	58.7 (56.2, 61.3)
Strongly agree	1141	13.0 (12.2, 13.8)	33.7 (31.6, 35.7)
Very strongly agree	1225	14.1 (13.3, 15.0)	36.5 (34.3, 38.6)
Non-response	94	1.2 (1.0, 1.5)	3.2 (2.5, 3.9)
Having more Black Americans, Latinos, and Asian Americans is good for the country			
Do not agree	1721	18.6 (17.7, 19.5)	48.2 (45.8, 50.5)
Somewhat agree	2989	34.0 (32.8, 35.1)	87.7 (84.8, 90.6)
Strongly agree	1960	23.2 (22.2, 24.2)	60.0 (57.3, 62.6)
Very strongly agree	1751	21.9 (20.9, 22.9)	56.6 (54.0, 59.2)
Non-response	199	2.3 (1.9, 2.6)	5.9 (5.0, 6.8)
In America, native-born white people are being replaced by immigrants			
Do not agree	4884	57.4 (56.2, 58.6)	148.3 (145.3, 151.3)
Somewhat agree	2206	25.0 (24.0, 26.0)	64.5 (61.9, 67.2)
Strongly agree	835	9.8 (9.1, 10.5)	25.4 (23.5, 27.2)
Very strongly agree	584	6.4 (5.8, 6.9)	16.5 (15.0, 17.9)
Non-response	111	1.4 (1.1, 1.7)	3.7 (2.9, 4.4)

Three items addressed the central elements of QAnon mythology and other beliefs (Table 5). Nearly 1 in 10 respondents (9.1%, 95% CI 8.3%, 9.8%) agreed strongly or very strongly that US institutions are “controlled by a group of Satan-worshipping pedophiles who run a global child sex trafficking operation,” and 10.0% (95% CI 9.3%, 10.8%) agreed strongly or very strongly that “a storm coming soon” will “sweep away the elites in power and restore the rightful leaders.” About 1 in 5 (19.3%, 95% CI 18.3%, 20.3%) agreed strongly or very strongly that “we are living in what the Bible calls ‘the end times.’.”

**Table 5:** Beliefs concerning QAnon and biblical “end times”

Statement	Respondents (*n* = 8620)		Estimated *N* of adults in USA
	Unweighted *n*	Weighted % (95% CI)	*N* (95% CI) (in millions)
The government, media, and financial worlds in the USA are controlled by a group of Satan-worshipping pedophiles who run a global child sex trafficking operation			
Do not agree	6775	74.9 (73.8, 76.0)	193.4 (190.6, 196.2)
Somewhat agree	1000	13.7 (12.8, 14.6)	35.3 (33.0, 37.6)
Strongly agree	329	4.5 (4.0, 5.1)	11.7 (10.3, 13.1)
Very strongly agree	328	4.5 (4.0, 5.1)	11.7 (10.3, 13.1)
Non-response	188	2.4 (2.0, 2.8)	6.2 (5.2, 7.2)
There is a storm coming soon that will sweep away the elites in power and restore the rightful leaders			
Do not agree	6031	67.8 (66.7, 68.9)	175.1 (172.2, 178.1)
Somewhat agree	1610	19.6 (18.6, 20.6)	50.6 (48.1, 53.1)
Strongly agree	429	5.5 (4.9, 6.0)	14.1 (12.6, 15.6)
Very strongly agree	348	4.6 (4.0, 5.1)	11.8 (10.4, 13.2)
Non-response	202	2.6 (2.2, 3.0)	6.7 (5.6, 7.7)
The chaos in America today is evidence that we are living in what the Bible calls “the end times”			
Do not agree	4905	54.7 (53.5, 55.9)	141.4 (138.3, 144.5)
Somewhat agree	2056	24.1 (23.1, 25.2)	62.4 (59.7, 65.0)
Strongly agree	694	8.9 (8.2, 9.6)	23.0 (21.1, 24.8)
Very strongly agree	821	10.4 (9.6, 11.2)	26.9 (24.9, 28.8)
Non-response	144	1.8 (1.5, 2.2)	4.7 (3.9, 5.6)

### Violence

As expected, respondents’ views on the justifiability of non-political violence varied substantially with circumstance (Fig. 1). Large majorities of respondents saw violence as usually or always justified in self-defense (76.1%, 95% CI 75.0%, 77.1%), or to prevent assaultive injury to others (77.9%, 95% CI 76.9%, 78.9%), and most considered it usually or always justified to prevent self-inflicted injury (59.2%, 95% CI 58.0%, 60.4%). Conversely, large majorities reported that violence was never justified to win an argument (85.7%, 95% CI 84.7%, 86.5%), respond to an insult (81.5%, 95% CI 80.5%, 82.5%), or get respect (86.2%, 95% CI 85.2%, 87.0%).

Only 3.0% (95% CI 2.6%, 3.6%) considered political violence to be usually or always justified “in general” (Table 6, Fig. 1). In most cases, slightly larger proportions of respondents considered violence to be usually or always justified to advance each of 17 specific political objectives considered individually (Tables 6, 7). Among those 17 objectives, support was most common for violence “to preserve an American way of life I believe in” (12.1%; 95% CI, 11.3%, 12.9%).

**Table 6:** Justification for political violence, in general and for 9 specific objectives

What do you think about the use of force or violence in the following situations?	Respondents (*n* = 8620)		Estimated *N* of adults in USA
	Unweighted *n*	Weighted % (95% CI)	*N* (95% CI) (in millions)
In general…to advance an important political objective that you support			
Never justified	7073	79.1 (78.1, 80.2)	204.4 (201.7, 207.5)
Sometimes justified	1330	17.5 (16.5, 18.4)	45.1 (42.7, 47.6)
Usually justified	131	2.1 (1.7, 2.6)	5.4 (4.5, 6.6)
Always justified	58	0.9 (0.7, 1.2)	2.4 (1.8, 3.2)
Non-response	28	0.4 (0.3, 0.6)	1.0 (0.8, 1.5)
Thinks violence is usually or always justified to advance at least 1 of 17 objectives	2770	32.8 (31.6, 33.9)	84.7 (81.7, 87.6)
To return Donald Trump to the presidency this year			
Never justified	7615	86.9 (85.9, 87.7)	224.6 (222.0, 226.5)
Sometimes justified	461	6.1 (5.5, 6.7)	15.8 (14.2, 17.4)
Usually justified	134	1.9 (1.6, 2.3)	5.0 (4.3, 6.0)
Always justified	287	3.6 (3.1, 4.1)	9.2 (8.1, 10.5)
Non-response	123	1.6 (1.3, 1.9)	4.1 (3.4, 4.9)
To stop an election from being stolen			
Never justified	6411	73.6 (72.6, 74.7)	190.2 (187.4, 192.9)
Sometimes justified	1397	16.4 (15.6, 17.3)	42.4 (40.2, 44.8)
Usually justified	291	3.7 (3.3, 4.3)	9.7 (8.5, 11.0)
Always justified	406	4.7 (4.2, 5.3)	12.2 ( 11.0, 13.6)
Non-response	114	1.5 (1.2, 1.8)	3.9 (3.1, 4.6)
To stop people who do not share my beliefs from voting			
Never justified	8031	91.8 (90.9, 92.5)	237.0 (235.0, 238.9)
Sometimes justified	329	4.8 (4.3, 5.4)	12.4 (11.0, 14.0)
Usually justified	94	1.5 (1.2, 1.8)	3.8 (3.0, 4.7)
Always justified	68	1.0 (0.8,1.3)	3.1 (2.0, 3.5)
Non-response	98	1.3 (1.0, 1.6)	3.4 (2.6, 4.1)
To prevent discrimination based on race or ethnicity			
Never justified	5592	62.7 (61.5, 63.9)	162.0 (159.0, 165.1)
Sometimes justified	2236	27.2 (26.1, 28.3)	70.2 (67.4, 73.0)
Usually justified	397	5.2 (4.7, 5.8)	13.4 (12.0, 14.9)
Always justified	280	3.8 (3.3, 4.3)	9.8 (8.6, 11.2)
Non-response	115	1.5 (1.2, 1.8)	3.9 (3.1, 4.6)
To preserve an American way of life based on Western European traditions			
Never justified	6354	74.0 (72.9, 75.0)	191.1 (188.4, 193.8)
Sometimes justified	1662	18.6 (17.1, 19.5)	48.1 (44.2, 50.5)
Usually justified	287	3.5 (3.1, 4.0)	9.1 (8.0, 10.3)
Always justified	165	2.1 (1.7, 2.5)	5.3 (4.5, 6.3)
Non-response	152	1.9 (1.6, 2.2)	4.9 (4.1, 5.7)
To preserve an American way of life I believe in			
Never justified	4702	55.4 (54.2, 56.6)	143.1 (140.0, 146.1)
Sometimes justified	2800	31.6 (30.5, 32.7)	81.7 (78.9, 84.6)
Usually justified	623	7.2 (6.6, 7.8)	18.5 (16.9, 20.2)
Always justified	428	4.9 (4.4, 5.4)	12.7 (11.4, 14.0)
Non-response	67	0.9 (0.6, 1.1)	2.3 (1.7, 2.9)
To oppose Americans who do not share my beliefs			
Never justified	7764	88.2 (87.4, 89.0)	227.8 (225.7, 230)
Sometimes justified	620	8.2 (7.5, 8.9)	21.2 (19.4, 23.0)
Usually justified	109	1.7 (1.3, 2.0)	4.4 (3.4, 5.3)
Always justified	70	1.1 (0.8, 1.4)	2.9 (2.1, 3.7)
Non-response	57	0.8 (0.5, 1.0)	2.0 (1.4, 2.5)
To oppose the government when it does not share my beliefs			
Never justified	7055	79.7 (78.7, 80.7)	205.9 (203.2, 208.4)
Sometimes justified	1204	15.3 (14.4, 16.2)	39.5 (37.2, 41.8)
Usually justified	167	2.3 (1.9, 2.7)	5.9 (5.0, 7.0)
Always justified	81	1.2 (1.0, 1.6)	3.2 (2.5, 4.1)
Non-response	113	1.5 (1.2, 1.9)	3.9 (3.1, 4.9)
To oppose the government when it tries to take private land for public purposes			
Never justified	5330	60.5 (59.3, 61.6)	156.2 (153.1, 159.2)
Sometimes justified	2423	28.2 (27.2, 29.3)	72.9 (70.2, 75.7)
Usually justified	438	5.8 (5.2, 6.4)	15.0 (13.5, 16.6)
Always justified	307	4.0 (3.5, 4.5)	10.3 (9.1, 11.6)
Non-response	122	1.5 (1.3,1.9)	3.9 (3.4, 4.9)

**Table 7:** Justification for political violence for 8 additional specific objectives (these objectives were paired, with respondents randomized 1:1 to see 1 item in each pair)

What do you think about the use of force or violence in the following situations?	Respondents (*n* = 8620)		Estimated *N* of adults in USA
	Unweighted *n*	Weighted % (95% CI)	*N* (95% CI) (in millions)
To stop voter fraud			
Never justified	3204	36.6 (35.5, 37.8)	94.6 (91.7, 97.6)
Sometimes justified	662	8.0 (7.4, 8.7)	20.7 (19.0, 22.4)
Usually justified	186	2.1 (1.8, 2.5)	5.5 (4.6, 6.4)
Always justified	224	2.6 (2.2, 3.0)	6.8 (5.8, 7.8)
Non-response	4344	50.6 (49.4, 51.8)	130.7 (127.6, 133.8)
To stop voter intimidation			
Never justified	2619	30.6 (29.5, 31.7)	79.0 (76.2, 81.9)
Sometimes justified	1207	14.0 (13.2, 14.8)	36.1 (34.0, 38.3)
Usually justified	236	2.7 (2.3, 3.1)	7.0 (6.0, 8.0)
Always justified	222	2.5 (2.1, 2.9)	6.5 (5.5, 7.4)
Non-response	4336	50.2 (49, 51.4)	129.7 (126.6, 132.8)
To reinforce the police			
Never justified	1721	20.7 (19.7, 21.7)	53.4 (50.9, 56.0)
Sometimes justified	1707	19.2 (18.3, 20.1)	49.6 (47.2, 52.0)
Usually justified	509	5.6 (5.1, 6.1)	14.4 (13.0, 15.8)
Always justified	313	3.4 (3.0, 3.8)	8.8 (7.7, 9.9)
Non-response	4370	51.1 (49.9, 52.3)	132.1 (129, 135.2)
To stop police violence			
Never justified	2057	23.0 (22.0, 24.0)	59.4 (56.8, 62.0)
Sometimes justified	1749	20.9 (19.9, 21.9)	54 (51.5, 56.6)
Usually justified	301	3.9 (3.4, 4.4)	10.0 (8.8, 11.3)
Always justified	200	2.5 (2.1, 2.9)	6.5 (5.5, 7.6)
Non-response	4313	49.7 (48.5, 50.9)	128.4 (125.3, 131.4)
To stop illegal immigration			
Never justified	2629	30.8 (29.7, 31.9)	79.5 (76.6, 82.3)
Sometimes justified	1156	13.4 (12.6, 14.2)	34.7 (32.6, 36.8)
Usually justified	274	3.2 (2.8, 3.7)	8.3 (7.2, 9.5)
Always justified	247	2.6 (2.3, 3.0)	6.8 (5.9, 7.8)
Non-response	4314	49.9 (48.7, 51.1)	129 (125.9, 132.1)
To keep borders open			
Never justified	2871	32.6 (31.5, 33.8)	84.3 (81.5, 87.2)
Sometimes justified	1051	12.4 (11.6, 13.2)	32.0 (30.0, 34.1)
Usually justified	206	2.5 (2.1, 2.9)	6.5 (5.5, 7.5)
Always justified	120	1.5 (1.2, 1.8)	3.9 (3.1, 4.8)
Non-response	4372	50.9 (49.7, 52.1)	131.5 (128.4, 134.6)
To stop a protest			
Never justified	2426	28.5 (27.4, 29.6)	73.6 (70.8, 76.3)
Sometimes justified	1538	17.6 (16.7, 18.5)	45.4 (43.1, 47.7)
Usually justified	174	2.0 (1.7, 2.3)	5.2 (4.3, 6.0)
Always justified	70	1.0 (0.7, 1.3)	2.5 (1.8, 3.2)
Non-response	4412	51.0 (49.8, 52.2)	131.7 (128.6, 134.7)
To support a protest			
Never justified	3504	39.3 (38.2, 40.5)	101.5 (98.6, 104.5)
Sometimes justified	677	8.6 (7.9, 9.3)	22.1 (20.3, 23.9)
Usually justified	121	1.6 (1.3, 1.9)	4.1 (3.3, 4.9)
Always justified	54	0.8 (0.5, 1.0)	1.9 (1.3, 2.5)
Non-response	4264	49.8 (48.6, 51.0)	128.6 (125.6, 131.7)

A third of respondents (32.8%, 95% CI 31.7%, 33.9%) considered violence to be usually or always justified to advance at least 1 of the 17 specific political objectives. Among these respondents, most (58.0%, 95% CI 55.9%, 60.1%) thought that violence was usually or always justified for 6 or more specific objectives (Additional file 1: Table S2).

Respondents who considered political violence at least somewhat justified to advance any of the 17 specific objectives were presented 2 series of items regarding their personal willingness to use force or violence “in a situation where you think force or violence is justified to advance an important political objective.” The first (Table 8) concerned types of violence: 3.1% of respondents (95% CI 2.6%, 3.5%) were very or completely willing to use force or violence “to damage property,” 2.2% (95% CI 1.8%, 2.6%) “to threaten or intimidate a person,” 2.2% (95% CI 1.8%, 2.6%) “to injure a person,” and 2.1% (95% CI 1.8%, 2.5%) “to kill a person.”

**Table 8:** Personal willingness to engage in political violence, by type of violence

In a situation where you think force or violence is justified to advance an important political objective…How willing would *you personally* be to use force or violence in each of these ways?	Respondents		Estimated *N* of adults in USA
	Unweighted *n*	Weighted % (95% CI)	*N* (95% CI) (in millions)
Political violence is never justified^a^	1852	21.6 (20.6, 22.6)	55.7 (53.2, 58.3)
To damage property			
Not willing	5911	67.0 (65.8, 68.1)	173 (170.1, 176)
Somewhat willing	599	7.7 (7.0, 8.4)	19.9 (18.2, 21.7)
Very willing	127	1.9 (1.6, 2.3)	5.0 (4.0, 6.0)
Completely willing	80	1.1 (0.8, 1.3)	2.8 (2.1, 3.5)
Non-response	51	0.7 (0.5, 0.9)	1.8 (1.3, 2.3)
To threaten or intimidate a person			
Not willing	6016	68.1 (67.0, 69.2)	175.9 (173, 178.9)
Somewhat willing	553	7.4 (6.7, 8.1)	19.1 (17.4, 20.9)
Very willing	77	1.2 (0.9, 1.5)	3.2 (2.4, 4.0)
Completely willing	66	0.9 (0.7, 1.2)	2.4 (1.7, 3.1)
Non-response	56	0.8 (0.5, 1.0)	2.0 (1.4, 2.5)
To injure a person			
Not willing	6110	69.4 (68.3, 70.5)	179.3 (176.4, 182.1)
Somewhat willing	447	6.0 (5.4, 6.6)	15.5 (13.9, 17.1)
Very willing	82	1.3 (1.0, 1.6)	3.3 (2.5, 4.1)
Completely willing	63	0.9 (0.6, 1.1)	2.3 (1.7, 2.9)
Non-response	66	0.9 (0.6, 1.1)	2.2 (1.6, 2.8)
To kill a person			
Not willing	6300	72.1 (71.0, 73.2)	186.2 (183.4, 189.0)
Somewhat willing	253	3.4 (2.9, 3.9)	8.8 (7.6, 10.1)
Very willing	80	1.1 (0.9, 1.4)	3.0 (2.2, 3.7)
Completely willing	79	1.0 (0.7, 1.2)	2.5 (1.9, 3.1)
Non-response	56	0.8 (0.6, 1.1)	2.1 (1.5, 2.7)

^a^These respondents answered “never justified” to all prior questions on the use of force or violence to advance specific political objectives. They were not asked questions on their personal willingness to use political violence

The second series (Table 9) concerned categories of people as potential targets of such violence, based on their occupations, personal beliefs, or race and ethnicity. When asked, again in a situation where they thought political violence was justified, “how willing would *you personally* be to use force or violence against a person because they are…,” between 1.4 and 2.3% of respondents were very or completely willing to commit violence against members of these specified populations.

**Table 9:** Personal willingness to engage in political violence, by target of violence

In a situation where you think force or violence is justified to advance an important political objective…How willing would *you personally* be to use force or violence against a person because they are…	Respondents		Estimated *N* of adults in USA
	Unweighted *n*	Weighted % (95% CI)	*N* (95% CI) (in millions)
Political violence is never justified^a^	1852	21.6 (20.6, 22.6)	55.7 (53.2, 58.3)
An elected federal or state government official			
Not willing	6188	70.6 (69.5, 71.7)	182.3 (179.4, 185.1)
Sometimes willing	361	4.7 (4.2, 5.3)	12.2 (10.8, 13.6)
Very willing	80	1.2 (0.9, 1.5)	3.2 (2.4, 3.9)
Completely willing	52	0.8 (0.5, 1.0)	2.0 (1.4, 2.7)
Non-response	87	1.1 (0.9, 1.4)	2.9 (2.2, 3.6)
An elected local government official			
Not willing	6222	71.1 (70.0, 72.2)	183.6 (180.8, 186.4)
Sometimes willing	327	4.3 (3.8, 4.9)	11.2 (9.9, 12.5)
Very willing	70	1.1 (0.8, 1.3)	2.7 (2.0, 3.5)
Completely willing	51	0.7 (0.5, 0.9)	1.9 (1.3, 2.4)
Non-response	98	1.2 (1.0, 1.5)	3.2 (2.5, 3.9)
An election worker, such as a poll worker or vote counter			
Not willing	6382	73 (71.9, 74.0)	188.5 (185.7, 191.3)
Sometimes willing	199	2.8 (2.3, 3.2)	7.1 (6.0, 8.2)
Very willing	65	1.1 (0.8, 1.3)	2.7 (2.0, 3.5)
Completely willing	39	0.6 (0.4, 0.8)	1.5 (1.0, 2.1)
Non-response	83	1.1 (0.8, 1.3)	2.7 (2.1, 3.4)
A public health official			
Not willing	6311	72.2 (71.1, 73.3)	186.5 (183.7, 189.3)
Sometimes willing	260	3.5 (3.0, 3.9)	8.9 (7.7, 10.1)
Very willing	62	1.0 (0.7, 1.2)	2.5 (1.8, 3.2)
Completely willing	44	0.7 (0.4, 0.9)	1.7 (1.2, 2.3)
Non-response	91	1.2 (0.9, 1.4)	3.0 (2.3, 3.7)
A member of the military or National Guard			
Not willing	6246	71.3 (70.2, 72.4)	184.1 (181.3, 186.9)
Sometimes willing	312	4.1 (3.6, 4.6)	10.7 (9.4, 12.0)
Very willing	76	1.3 (0.9, 1.6)	3.3 (2.4, 4.2)
Completely willing	49	0.7 (0.5, 0.9)	1.8 (1.2, 2.3)
Non-response	85	1.1 (0.8, 1.3)	2.7 (2.1, 3.4)
A police officer			
Not willing	6185	70.4 (69.3, 71.6)	182 (179.1, 184.8)
Sometimes willing	345	4.6 (4.1, 5.1)	11.8 (10.5, 13.2)
Very willing	90	1.3 (1.0, 1.7)	3.5 (2.7, 4.3)
Completely willing	63	0.9 (0.7, 1.2)	2.4 (1.8, 3.1)
Non-response	85	1.1 (0.8, 1.4)	2.8 (2.2, 3.5)
A person who does not share your race or ethnicity			
Not willing	6380	72.8 (71.7, 73.9)	188.1 (185.3, 190.9)
Sometimes willing	202	2.9 (2.5, 3.4)	7.6 (6.4, 8.8)
Very willing	58	0.9 (0.6, 1.1)	2.3 (1.6, 2.9)
Completely willing	43	0.7 (0.5, 0.9)	1.8 (1.2, 2.4)
Non-response	85	1.1 (0.8, 1.4)	2.8 (2.2, 3.5)
A person who does not share your religion			
Not willing	6394	73.1 (72.0, 74.2)	188.8 (186.0, 191.6)
Sometimes willing	180	2.6 (2.2, 3.0)	6.7 (5.6, 7.9)
Very willing	63	1.0 (0.7, 1.3)	2.6 (1.9, 3.4)
Completely willing	35	0.5 (0.3, 0.6)	1.2 (0.8, 1.7)
Non-response	96	1.2 (1.0, 1.5)	3.2 (2.5, 3.9)
A person who does not share your political beliefs			
Not willing	6324	72.3 (71.2, 73.3)	186.7 (183.9, 189.5)
Sometimes willing	266	3.7 (3.2, 4.2)	9.4 (8.2, 10.7)
Very willing	57	0.9 (0.6, 1.1)	2.2 (1.6, 2.9)
Completely willing	37	0.6 (0.4, 0.7)	1.4 (0.9, 1.9)
Non-response	84	1.1 (0.8, 1.4)	2.8 (2.2, 3.5)

^a^These respondents answered “never justified” to all prior questions on the use of force or violence to advance specific political objectives. They were not asked questions on their personal willingness to use political violence

Finally, all respondents, regardless of their position on political violence or firearm ownership status, were asked to predict the likelihood of their future use of a firearm “in a situation where you think force or violence is justified to advance an important political objective”; 7.7% (95% CI 7.0%, 8.4%) thought it very or extremely likely that “I will be armed with a gun,” 4.1% (95% CI 3.6%, 4.7%) that “I will carry a gun openly, so that people know I am armed,” 1.0% (95% CI 0.7%, 1.3%) that “I will threaten someone with a gun,” and 1.1% (95% CI 0.9%, 1.4%) that “I will shoot someone with a gun” (Table 10).

**Table 10:** Future likelihood of firearm possession and use in a situation where political violence is perceived as justified

Thinking now about the future and all the changes it might bring, how likely is it that you will use a gun in any of the following ways in the next few years—in a situation where you think force or violence is justified to advance an important political objective?	Respondents (*n* = 8620)		Estimated *N* of adults in USA
	Unweighted *n*	Weighted % (95% CI)	*N* (95% CI) (in millions)
I will be armed with a gun			
Not likely	7107	80.1 (79.1, 81.1)	206.9 (204.3, 209.5)
Somewhat likely	833	10.8 (10.1, 11.6)	27.9 (26.0, 30.0)
Very likely	254	3.4 (3.0, 3.9)	8.8 (7.7, 10.1)
Extremely likely	318	4.3 (3.8, 4.8)	11.0 (9.8, 12.5)
Non-response	108	1.4 (1.1, 1.7)	3.6 (2.8, 4.4)
I will carry a gun openly, so that people know I am armed			
Not likely	7779	88.7 (87.8, 89.5)	229.0 (226.8, 231.1)
Somewhat likely	435	5.7 (5.1, 6.3)	14.7 (13.2, 16.3)
Very likely	163	2.2 (1.8, 2.6)	5.6 (4.7, 6.6)
Extremely likely	126	2.0 (1.6, 2.4)	5.1 (4.2, 6.2)
Non-response	117	1.5 (1.2, 1.9)	3.9 (3.1, 4.9)
I will threaten someone with a gun			
Not likely	8351	96.2 (95.6, 96.6)	248.4 (247.0, 249.6)
Somewhat likely	93	1.4 (1.1, 1.7)	3.5 (2.8, 4.4)
Very likely	38	0.7 (0.5, 0.9)	1.7 (1.2, 2.4)
Extremely likely	23	0.3 (0.2, 0.5)	0.8 (0.5, 1.3)
Non-response	115	1.5 (1.2, 1.9)	3.9 (3.1, 4.9)
I will shoot someone with a gun			
Not likely	8235	94.6 (94.0, 95.2)	244.4 (242.8, 245.9)
Somewhat likely	198	2.8 (2.4, 3.3)	7.2 (6.2, 8.5)
Very likely	36	0.6 (0.4, 0.8)	1.4 (1.0, 2.1)
Extremely likely	40	0.6 (0.4, 0.8)	1.4 (1.0, 2.0)
Non-response	111	1.5 (1.2, 1.8)	3.9 (3.1, 4.6)

### Variation with sociodemographic characteristics

Bivariate variation on all measures with respondents’ age, gender, race and ethnicity, education, income, and region of residence is presented in detail in Additional file 1: Tables S3–S12 and summarized graphically (Additional file 1: Figure S2) for age, gender, education, and income. Support for violence as potentially justified by conditions in the USA and for political violence reliably decreased as education and income increased and frequently decreased with increasing age. Associations with gender, race and ethnicity, and region of residence were variable.

## Discussion

The motivating premises for this survey were that current conditions in the USA create both perceived and actual threats to its future as a free and democratic society. The findings bear out both premises. As to the former, more than two-thirds of respondents perceived “a serious threat to our democracy”; 1 in 7 strongly or very strongly agreed that there will be civil war in the next few years. As to the latter, 10% thought it only somewhat important or not important for the USA to remain a democracy; nearly 20% strongly or very strongly agreed that “having a strong leader for America is more important than having a democracy”; and 3% believed that, in general, political violence was usually or always justified.

Many findings from this survey are concordant with those of polls taken over the last 2 years (NPR/PBS NewsHour/Marist National Poll 2021; Grinnell College National Poll 2021; Romano 2022; Cox 2021; The Economist/YouGov Poll 2021; Public Religion Research Institute 2022; Associated Press-NORC Center for Public Affairs Research 2022; Survey Center on American Life 2021; Zogby 2021; Pew Research Center 2019; IFYC – PRRI 2021). These include support by substantial proportions of the population for broad statements of the potential need for violence to save a society perceived as heading in the wrong direction and for false beliefs, such as the QAnon complex, great replacement thinking, and the myth that Donald Trump won the 2020 Presidential election. This concordance demonstrates the stability of the findings from the earlier work and provides a foundation for the new results presented here.

Our population-level extrapolations (some based on small numbers and therefore to be interpreted with caution (Hemenway 1997)) suggest that nearly 8 million adults in the USA consider violence to be usually or always justified “in general” to advance political objectives that they support.

These are not abstract beliefs, made without commitment. Our extrapolations also suggest that millions of Americans would be very or completely willing to engage in violence themselves to advance a political objective that they support; between 5 and 6 million people would threaten or intimidate someone, injure them, or kill them.

For many, future situations in which they consider political violence to be justified might call for the use of firearms. We estimate that nearly 20 million Americans think it very or extremely likely that they will be armed in such a situation in the next few years, nearly 11 million that they will carry a gun openly, and nearly 3 million that they will shoot someone. (Given the actual incidence of firearm violence in the USA (Centers for Disease Control and Prevention 2023), we believe it is obvious that the vast majority of those contemplated shootings will never occur.)

In the aggregate, these initial findings suggest a continuing alienation from and mistrust of American democratic society and its institutions, founded in part on false beliefs. They suggest a concerning level of support for violence, including lethal violence, to advance political objectives; this likely increases the risk of large-scale political violence in the near future (Walter 2022). There is important variation with demographic characteristics, and other analyses from this survey suggest that support for political violence may vary substantially with political party affiliation and political ideology (Wintemute et al. 2022a, b). Forthcoming analyses will shed light on additional factors associated with that support and inform efforts to prevent the risk of political violence from being realized.

It is important to emphasize that these findings also provide firm ground for hope. A large majority of respondents rejected political violence altogether, whether generally or to advance any single political objective, and most of those who did endorse political violence in the abstract were unwilling to resort to violence themselves. The challenge now for those large majorities is to recognize the threat posed by those who are willing to engage in political violence and respond adequately to it.

### Limitations

Several technical limitations exist. The findings are cross-sectional and subject to sampling error and non-response bias; this is particularly applicable to our findings related to age, education, and income, as respondents and non-respondents differed on these measures. Many important outcomes are uncommon, with response counts < 100 and weighted prevalences below 5%. The large study sample and small prevalence estimates result in relatively narrow confidence intervals in these cases, but the estimates remain vulnerable to bias from sources such as inattentive or strategic responses. That vulnerability is increased in the national estimates based on extrapolation. Widely publicized mass shootings occurred in Buffalo, NY and Uvalde, TX, while the survey was in the field. The Buffalo shooting is understood to have been a race-related hate crime motivated by great replacement thinking and may have affected respondents’ views on race, violence, and that particular belief. Russia’s war against Ukraine may have influenced responses on violence and democracy.

Follow-up studies are in development to explore the meaning and implications of the findings presented here. For example, does a respondent who expects civil war view that war positively or negatively? Similarly, this survey did not solicit specific information on what gives rise to support for political violence, or on how that support or its causes might best be addressed in prevention efforts.

## Conclusion

Findings from this large, nationally representative survey suggest that concerning proportions of the US population currently support violence, including lethal violence, to advance political objectives. Support varies with demographic characteristics. Efforts to prevent that violence should proceed rapidly based on the best evidence available, while further research identifies factors associated with support for political violence and informs future prevention efforts.

## Supplementary Information

**Additional file 1**. Supplemental materials.

## Data Availability

The datasets generated and/or analyzed during the current study are not publicly available as analyses are continuing but will be made available to qualified researchers subject to the terms of a data use agreement.

## Abbreviations

CI: Confidence interval
