# Correction: Views of democracy and society and support for political violence in the USA: findings from a nationally representative survey

**DOI:** 10.1186/s40621-025-00606-9

**Published:** 2025-08-19

**Authors:** Garen J. Wintemute, Sonia L. Robinson, Andrew Crawford, Daniel Tancredi, Julia P. Schleimer, Elizabeth A. Tomsich, Paul M. Reeping, Aaron B. Shev, Veronica A. Pear

**Affiliations:** 1 UC Davis Violence Prevention Research Program, Sacramento, CA USA; 2 Department of Emergency Medicine, UC Davis, Sacramento, CA USA; 3 California Firearm Violence Research Center, Sacramento, CA USA; 4 Department of Pediatrics, UC Davis, Sacramento, CA USA

**Correction:** **Injury. Epidemiology. (2023) 10:45**

10.1186/s40621-023-00456-3

Following publication of the original article [1], the authors identified an error in the Additional file 1: Supplemental materials. Page 1 of the corrected supplement included this notice: This is a corrected version, submitted January 25, 2024. The original version contained incorrect information on non-respondents in Table S1.

The Additional file 1: Supplemental materials has been updated in this correction and the original article [1] has been corrected.

## Supplementary Information

Supplementary Material 1.
