# Supplementary material for: Views of democracy and society and support for political violence in the USA: findings from a nationally representative survey
Source: Inj Epidemiol. 2023 Sep 29;10:45. doi: 10.1186/s40621-023-00456-3 (PMC10540371; doi:10.1186/s40621-023-00456-3)

Supplement

Views of Democracy and Society and Support for Political Violence in the United States:

Findings from a Nationally Representative Survey

Garen J. Wintemute, MD, MPH*; Sonia L. Robinson, PhD, MPH; Andrew Crawford, PhD; Daniel Tancredi, PhD; Julia P. Schleimer, MPH; Elizabeth A. Tomsich, PhD; Paul M. Reeping, PhD; Aaron B. Shev, PhD; Veronica A. Pear, PhD, MPH, MA.

This supplement has been provided by the authors to give readers additional information about the work.

This is a corrected version, submitted January 25, 2024. The original version contained incorrect information on non-respondents in Table S1.

Page	Title
3	Questions from the 2022 American Life Survey that supplied data for this study
10	References for the question list
11	Table S1. Comparison of respondents and nonrespondents
13	Table S2. Frequency distribution of respondents by the number of objectives perceived as usually or always justifying violence
14	Table S3. Variation with respondent characteristics in beliefs concerning democracy in the US
17	Table S4. Variation with respondent characteristics in beliefs concerning the potential need for violence in the US
19	Table S5. Variation with respondent characteristics in beliefs concerning race and ethnicity and American society
21	Table S6. Variation with respondent characteristics in beliefs concerning QAnon and biblical “end times”
22	Table S7. Variation with respondent characteristics in beliefs concerning justification for violence in non-political situations
27	Table S8. Variation with respondent characteristics in beliefs concerning justification for political violence, in general and for 9 specific objectives
30	Table S9. Variation with respondent characteristics in beliefs concerning justification for political violence for 8 additional specific objectives

34	Table S10. Variation with respondent characteristics in personal willingness to engage in political violence, by type of violence
36	Table S11. Variation with respondent characteristics in personal willingness to engage in political violence, by target of violence
41	Table S12. Variation with respondent characteristics in future likelihood of firearm possession and use in a situation where political violence is perceived as justified
43	Figure S1. Observed and expected monthly counts of National Instant Criminal Background Check System background checks for firearm purchases, January 2014-May 2023
44	Figure S2. Association between respondents' age, gender, education, and income and their views of democracy and society and support for political violence in the United States

Questions from the 2022 American Life Survey that supplied data for this study

For questions that presented a series of items for separate consideration, such as the third question below, all those items are listed, even though not all were used in this analysis.

Response options are presented here in order from negative to positive (e.g., "not important" to "extremely important"). Respondents were randomized 1:1 to receive responses in that order or the reverse.

In the list below, questions or items that were repeated or adapted from prior surveys contain citations to those surveys.

Domain 1: democracy in the United States

Now we'd like to ask you a few questions about the United States as you see it now, in 2022.

Q1: When thinking about democracy in the United States these days, do you believe…?[1]

1. There is a serious threat to our democracy.
2. There may be a threat to our democracy, but it is not serious.
3. There is no threat to our democracy.

Q2: How important do you think it is for the United States to remain a democracy?[2]

1. Not important
2. Somewhat important
3. Very important
4. Extremely important

Q3: How much do you agree or disagree with the following statements about democracy in the United States?

a. Democracy is the best form of government.[3]

b. These days, American democracy only serves the interests of the wealthy and powerful.[4]

c. Having a strong leader for America is more important than having a democracy.

d. If elected leaders will not protect American democracy, the people must do it themselves, even if it requires taking violent actions.[4]

e. In the next few years, there will be civil war in the United States.[5]

1. Do not agree
2. Somewhat agree
3. Strongly agree
4. Very strongly agree

Domain 2: American society and institutions

The next few questions are about your views of American society.

Q4: How much do you agree or disagree with each of the following statements about people in America today?

a. White people benefit from advantages in society that Black people do not have.[4]

b. Discrimination against whites is as big a problem as discrimination against Blacks and other minorities.[4]

c. Our American way of life is disappearing so fast that we may have to use force to save it.[4]

d. In America, native-born white people are being replaced by immigrants.

e. Having more Black Americans, Latinos, and Asian Americans is good for the country.[6]

1. Do not agree
2. Somewhat agree
3. Strongly agree
4. Very strongly agree

Q5: People have many different views about American society. How much do you agree or disagree with each of the following?

a. The government, media, and financial worlds in the U.S. are controlled by a group of Satan-worshipping pedophiles who run a global child sex trafficking operation.[7]

b. There is a storm coming soon that will sweep away the elites in power and restore the rightful leaders.[7]

c. Because things have gotten so far off track, true American patriots may have to resort to violence in order to save our country.7

d. The chaos in America today is evidence that we are living in what the Bible calls "the end times."[8]

e. Capitalism is a system of oppression and should be abolished.

f. Straight white men hold far too much power in America.

g. The 2020 election was stolen from Donald Trump, and Joe Biden is an illegitimate president.

h. Armed citizens should patrol polling places at election time.

1. Do not agree
2. Somewhat agree
3. Strongly agree
4. Very strongly agree

Domain 3: violence, including political violence

Now we have a few questions about the use of force or violence. A reminder: your responses will be kept confidential and anonymous.

Q6: In general, what do you think about the use of force or violence in the following situations—is it never justified, sometimes justified, usually justified, or always justified? "Force or violence" means physical force strong enough that it could cause pain or injury to a person.

(Not randomized)

a. In self defense

b. To prevent someone from injuring or killing another person

c. To prevent someone from injuring or killing themselves

d. To prevent harm or damage to property

e. To win an argument

f. In response to an insult

g. To get respect

1. Never justified
2. Sometimes justified

3. Usually justified
4. Always justified

Q7: People sometimes talk about using force or violence to achieve political objectives. In general, what do you think about using force or violence to advance an important political objective that you support—is it…?

1. Never justified
2. Sometimes justified
3. Usually justified
4. Always justified

Q8: You said that in general, the use of force or violence was [response inserted] to advance an important political objective that you support. Your opinion might depend on the specific objective that was involved. What do you think about the use of force or violence in the following situations—is it never justified, sometimes justified, usually justified, or always justified?

[Items 1a-4b were paired, and each respondent was presented with 1 item from each pair.]

1a. To stop voter fraud

OR

1b. To stop voter intimidation

2a. To stop police violence

OR

2b. To reinforce the police

3a. To stop illegal immigration

OR

3b. To keep our borders open

4a. To stop a protest or demonstration

OR

4b. To support a protest or demonstration

AND

5. To preserve the American way of life l believe in

6. To oppose Americans who do not share my beliefs

1. Never justified
2. Sometimes justified
3. Usually justified
4. Always justified

Q9: Again, your view of the use of force or violence to advance an important political objective might depend on the specific objective that was involved. What do you think about the use of force or violence in the following situations—is it never justified, sometimes justified, usually justified, or always justified?

a. To return Donald Trump to the presidency this year

b. To stop an election from being stolen

c. To stop people who do not share my beliefs from voting

d. To prevent discrimination based on race or ethnicity

e. To preserve an American way of life based on Western European traditions

f. To oppose the government when it does not share my beliefs

g. To oppose the government when it tries to take private land for public purposes

1. Never justified
2. Sometimes justified
3. Usually justified
4. Always justified

The next questions are about your personal willingness to use force or violence.

(Questions asked of respondents who endorsed at least 1 use of violence to achieve a specific political objective.)

Q10: In a situation where you think force or violence is justified to advance an important political objective, how willing would you personally be to use force or violence in each of these ways?

a. To damage property

b. To threaten or intimidate a person

c. To injure a person

d. To kill a person

1. Not willing
2. Somewhat willing
3. Very willing
4. Completely willing

Q11: In a situation where you think force or violence is justified to advance an important political objective, how willing would you personally be to use force or violence against a person because they are…

a. An elected federal or state government official

b. An elected local government official

c. A public health official

d. A member of the military or National Guard

e. A police officer

f. A person who does not share your race or ethnicity

g. A person who does not share your religion

h. An election worker, such as a poll worker or vote counter

i. A person who does not share your political beliefs

1. Not willing
2. Somewhat willing
3. Very willing
4. Completely willing

(Question asked of all respondents.)

Q12: Thinking now about the future and all the changes it might bring, how likely is it that you will use a gun in any of the following ways in the next few years—in a situation where you think force or violence is justified to advance an important political objective?

a. I will be armed with a gun.

b. I will carry a gun openly, so that people know I am armed.

c. I will threaten someone with a gun.

d. I will shoot someone with a gun.

1. Not likely
2. Somewhat likely
3. Very likely
4. Extremely likely

References for the question list

1. NPR/PBS NewsHour/Marist National Poll. Trust in elections, threat to democracy, November 2021. 2021 November 1. https://maristpoll.marist.edu/polls/npr-pbs-newshour-marist-national-poll-trust-in-elections-threat-to-democracy-biden-approval-november-2021/.
2. Grinnell College National Poll. 52% of Americans believe democracy facing “major threat.” Study #2243. 2021 October 20. https://www.grinnell.edu/news/52-americans-believe-democracy-facing-major-threat.
3. The Economist/YouGov Poll. 2021 June 13-16. https://docs.cdn.yougov.com/uagnfc262c/econTabReport.pdf.
4. Survey Center on American Life. January 2021 American Perspectives Survey topline questionnaire. https://www.americansurveycenter.org/wp-content/uploads/2021/03/January-2021-APS-Topline-Questionnaire.pdf.
5. Zogby. Will the US have another civil war? 2021 Feb 4. https://zogbyanalytics.com/news/997-the-zogby-poll-will-the-us-have-another-civil-war
6. Pew Research Center. Americans see advantages and challenges in country’s growing racial and ethnic diversity. 2019 May. https://www.pewresearch.org/social-trends/2019/05/08/americans-see-advantages-and-challenges-in-countrys-growing-racial-and-ethnic-diversity/.
7. Public Religion Research Institute. The persistence of Q-Anon in the post-Trump era: an analysis of who believes the conspiracies. 2022 Feb 24. https://www.prri.org/research/the-persistence-of-qanon-in-the-post-trump-era-an-analysis-of-who-believes-the-conspiracies/.
8. IFYC – PRRI Survey on Religion & COVID-19 Vaccine Trust. 2021 March. https://www.prri.org/wp-content/uploads/2021/05/Topline-IFYC-PRRI-Survey-on-Religion-and-COVID-19-Vaccine-Trust-v2_final.pdf.

Table S1. Comparison of respondents and nonrespondents

Characteristic	Respondents (n = 8,620)		Non-respondents (n = 6,099)	
	Unweighted n	Unweighted %	Unweighted n	Unweighted %
Age				
18-24	447	5.2	977	16.0
25-34	1024	11.9	1129	18.5
35-44	1374	15.9	1340	22.0
45-54	1215	14.1	1151	18.9
55-64	1833	21.3	811	13.3
65-74	1788	20.7	502	8.2
75+	939	10.9	189	3.1
Non-response	0	0.0	0	0.0
Gender				
Male	4247	49.3	3313	54.3
Female	4373	50.7	2786	45.7
Non-response	0	0.0	0	0.0
Race and ethnicity				
White, non-Hispanic	6047	70.2	3528	57.9
Black, non-Hispanic	836	9.7	836	13.7
Other, non-Hispanic	392	4.5	1183	19.4
Hispanic	1084	12.6	304	5.0
2+ races, non-Hispanic	261	3.0	248	4.1
Non-response	0	0.0	0	0.0
Marital status				
Now married	5246	60.9	3128	51.3
Widowed	443	5.1	168	2.8
Divorced	909	10.5	577	9.5
Separated	139	1.6	141	2.3
Never married	1883	21.8	2085	34.2
Non-response	0	0.0	0	0.0
Education				
No high school diploma or GED	542	6.3	625	10.3
High school graduate (HS diploma or GED)	2158	25.0	1759	28.8
Some college or Associate degree	2364	27.4	1769	29.0
Bachelor's degree	1951	22.6	1140	18.7
Master's degree or higher	1605	18.6	806	13.2
Non-response	0	0.0	0	0.0
Household income				
< $10,000	272	3.2	312	5.1
$10,000 - $24,999	745	8.6	609	10.0
$25,000 - $49,999	1469	17.0	1158	19.0
$50,000 - $74,999	1414	16.4	1012	16.6
$75,000 - $99,999	1214	14.1	810	13.3
$100,000 - $149,999	1500	17.4	1031	16.9
>= $150,000	2006	23.3	1167	19.1
Non-response	0	0.0	0	0.0
Employment				
Working full-time	3888	45.1	3377	55.4
Working part-time	1132	13.1	1055	17.3
Not working	3600	41.8	1667	27.3
Non-response	0	0.0	0	0.0
Census division				
New England	412	4.8	258	4.2
Mid-Atlantic	1090	12.6	747	12.3
East-North Central	1267	14.7	840	13.8
West-North Central	604	7.0	420	6.9
South Atlantic	1714	19.9	1232	20.2
East-South Central	465	5.4	432	7.1
West-South Central	904	10.5	840	13.8
Mountain	745	8.6	426	7.0
Pacific	1419	16.5	904	14.8
Non-response	0	0.0	0	0

Note: Weights were not available for non-respondents; this table presents unweighted counts and percentages.

* Unweighted mean (SD) ages were 53.8 (17.2) for respondents and 42.9 (16.2) for nonrespondents.

Table S2. Frequency distribution of respondents by the number of objectives perceived as usually or always justifying violence

Number of objectives justified*	Respondents†		Adults in US
	Unweighted n	Weighted % (95% CI)	N (95% CI) (in millions)
1	1146	13.1 (12.3, 13.9)	33.8 (31.8, 35.9)
2	1021	11.7 (11.0, 12.5)	30.2 (28.4, 32.3)
3	913	10.2 (9.6, 11.0)	26.3 (24.8, 28.4)
4	790	9.0 (8.3, 9.7)	23.2 (21.4, 25.1)
5	652	7.4 (6.8, 8.0)	19.1 (17.6, 20.7)
6	576	6.6 (6.0, 7.2)	17.0 (15.5, 18.6)
7	456	5.2 (4.7, 5.7)	13.4 (12.1, 14.7)
8	379	4.2 (3.8, 4.7)	10.8 (9.8, 12.1)
9	268	3.1 (2.7, 3.5)	8.0 (7.0, 9.0)
10	169	2.0 (1.7, 2.3)	5.2 (4.4, 5.9)
11	116	1.6 (1.3, 2.0)	4.1 (3.4, 5.2)
12	103	1.5 (1.2, 1.8)	3.9 (3.1, 4.6)
13	179	2.9 (2.5, 3.4)	7.5 (6.5, 8.8)

* There were 17 specified objectives. Nine were presented to all respondents, and 8 were paired, with each respondent seeing only 1 item from each pair; each respondent was presented with 13 of 17 objectives.

† A weighted 21.6% (95% CI 20.6-22.6) of the sample considered violence never justified on all political objectives.

Table S3. Variation with respondent characteristics in beliefs concerning democracy in the US

Characteristic	Do you believe that things in this country today are…				When thinking about democracy in the United States these days, do you believe…						How important do you think it is for the United States to remain a democracy?					
	Generally headed in the wrong direction		Generally headed in the right direction		There is a serious threat to our democracy.		There may be a threat to our democracy, but it is not serious.		There is no threat to our democracy.		Not important		Somewhat important		Very or extremely important	
	Unweighted n	Weighted %, 95% CI	Unweighted n	Weighted %, 95% CI	Unweighted n	Weighted %, 95% CI	Unweighted n	Weighted %, 95% CI	Unweighted n	Weighted %, 95% CI	Unweighted n	Weighted %, 95% CI	Unweighted n	Weighted %, 95% CI	Unweighted n	Weighted %, 95% CI
**Age, years**																
18-24	361	79.3 (75.0, 83.5)	81	20.7 (16.5, 25.0)	250	55.5 (50.5, 60.5)	145	33.7 (29.0, 38.5)	43	10.8 (7.6, 14.0)	17	4.3 (2.2, 6.4)	59	14.7 (11.1, 18.3)	365	81.0 (77.0, 85.0)
25-34	847	82.9 (80.2, 85.6)	162	17.1 (14.4, 19.8)	644	61.4 (58.1, 64.8)	279	29.2 (26.0, 32.3)	84	9.4 (7.3, 11.4)	37	4.1 (2.7, 5.5)	114	12.4 (10.1, 14.8)	857	83.5 (80.9, 86.1)
35-44	1117	81.7 (79.5, 83.9)	241	18.3 (16.1, 20.5)	865	62.5 (59.7, 65.3)	369	28.0 (25.5, 30.6)	119	9.5 (7.7, 11.2)	35	2.5 (1.6, 3.3)	120	10.0 (8.2, 11.8)	1201	87.5 (85.6, 89.5)
45-54	999	81.4 (79.0, 83.7)	208	18.6 (16.3, 21.0)	829	67.7 (64.9, 70.5)	280	23.7 (21.1, 26.2)	94	8.6 (6.9, 10.4)	20	1.9 (1.0, 2.9)	84	7.4 (5.8, 9.0)	1099	90.7 (88.8, 92.5)
55-64	1521	83.4 (81.5, 85.2)	298	16.6 (14.8, 18.5)	1360	74.3 (72.1, 76.4)	336	19.0 (17.0, 20.9)	115	6.8 (5.5, 8.1)	21	1.2 (0.7, 1.8)	85	5.3 (4.1, 6.5)	1712	93.5 (92.2, 94.8)
65-74	1453	82.0 (80.1, 83.9)	312	18.0 (16.1, 19.9)	1422	79.6 (77.6, 81.6)	277	15.8 (14.0, 17.6)	78	4.6 (3.5, 5.7)	14	0.8 (0.4, 1.3)	39	2.4 (1.6, 3.2)	1727	96.7 (95.8, 97.6)
75+	745	79.4 (76.6, 82.2)	184	20.6 (17.8, 23.4)	747	78.7 (75.8, 81.6)	146	16.8 (14.2, 19.4)	40	4.5 (3.1, 5.9)	1	0.1 (0.0, 0.3)	9	1.3 (0.4, 2.1)	925	98.7 (97.8, 99.5)
**Gender**																
Male	3358	79.9 (78.5, 81.4)	775	20.1 (18.6, 21.5)	2909	66.9 (65.2, 68.6)	922	24.8 (23.2, 26.3)	292	8.3 (7.3, 9.4)	75	2.4 (1.8, 3.0)	184	5.9 (5.0, 6.9)	3871	91.7 (90.6, 92.8)
Female	3549	83.3 (82.0, 84.6)	693	16.7 (15.4, 18.0)	3095	69.4 (67.8, 71.0)	880	23.2 (21.7, 24.7)	268	7.4 (6.4, 8.3)	65	2.0 (1.5, 2.6)	309	9.4 (8.3, 10.5)	3884	88.6 (87.4, 89.8)
Other	93	84.6 (75.9, 93.3)	13	15.4 (6.7, 24.1)	81	74.8 (65.2, 84.4)	18	17.7 (9.3, 26.1)	6	7.5 (1.5, 13.6)	5	3.8 (0.3, 7.3)	11	12.9 (4.7, 21.2)	88	83.3 (74.6, 91.9)
**Race and ethnicity**																
White, non-Hispanic	5140	86.3 (85.4, 87.2)	855	13.7 (12.8, 14.6)	4506	73.7 (72.5, 74.9)	1192	21.1 (20.0, 22.3)	294	5.2 (4.6, 5.8)	78	1.7 (1.3, 2.1)	266	5.5 (4.9, 6.2)	5655	92.8 (92.0, 93.6)
Black, non-Hispanic	621	77.1 (73.8, 80.3)	200	22.9 (19.7, 26.2)	576	68.2 (64.5, 71.9)	171	22.9 (19.5, 26.4)	70	8.9 (6.6, 11.1)	20	3.1 (1.6, 4.6)	93	13.9 (10.9, 16.8)	707	83.1 (79.9, 86.2)
Hispanic, any race	786	72.5 (69.5, 75.5)	277	27.5 (24.5, 30.5)	599	52.6 (49.3, 56.0)	303	31.0 (27.9, 34.2)	161	16.4 (13.8, 18.9)	34	3.4 (2.1, 4.6)	111	12.3 (10, 14.6)	928	84.3 (81.8, 86.8)
Asian American / Pacific Islander	211	67.8 (61.9, 73.7)	99	32.2 (26.3, 38.1)	178	54.7 (48.3, 61.0)	101	34.1 (28.1, 40.1)	31	11.3 (7.0, 15.5)	2	1.0 (0.0, 2.4)	13	4.8 (2.0, 7.6)	295	94.2 (91.1, 97.3)
Other (American Indian/Alaskan, 2+ races, other)	285	82.0 (75.9, 88.2)	55	18.0 (11.8, 24.1)	258	64.3 (56.4, 72.1)	65	28.3 (20.7, 35.9)	17	7.4 (2.7, 12.1)	11	5.3 (1.2, 9.4)	27	13.6 (7.4, 19.8)	301	81.1 (74.1, 88.1)
**Education**																
No high school diploma or GED	394	72.2 (67.9, 76.5)	137	27.8 (23.5, 32.1)	299	52.6 (47.9, 57.4)	152	31.5 (27.0, 36.0)	75	15.9 (12.3, 19.4)	25	4.9 (2.9, 6.9)	82	17.4 (13.6, 21.1)	425	77.8 (73.7, 81.8)
High school graduate	1794	83.1 (81.2, 84.9)	335	16.9 (15.1, 18.8)	1509	67.6 (65.2, 69.9)	452	23.8 (21.6, 25.9)	158	8.7 (7.2, 10.1)	54	3.2 (2.2, 4.1)	205	11.7 (10, 13.3)	1856	85.2 (83.3, 87.0)
Some college or Associates degree	1976	84.7 (83.0, 86.4)	359	15.3 (13.6, 17.0)	1742	72.3 (70.2, 74.4)	469	21.9 (20.0, 23.9)	128	5.8 (4.7, 6.9)	46	2.3 (1.6, 3.1)	124	6.4 (5.2, 7.6)	2178	91.3 (89.9, 92.6)
Bachelors degree	1617	82.6 (80.7, 84.5)	326	17.4 (15.5, 19.3)	1391	68.9 (66.5, 71.2)	426	23.9 (21.8, 26.1)	123	7.2 (5.9, 8.6)	14	0.8 (0.4, 1.3)	67	4.5 (3.3, 5.6)	1866	94.7 (93.5, 95.9)
Masters degree or higher	1262	78.9 (76.7, 81.1)	329	21.1 (18.9, 23.3)	1176	70.6 (68.1, 73.1)	333	23.1 (20.8, 25.5)	89	6.2 (4.9, 7.6)	6	0.4 (0.0, 0.7)	32	2.4 (1.5, 3.2)	1561	97.3 (96.3, 98.2)
**Income**																
<$25,000	801	80.0 (77.2, 82.9)	199	20.0 (17.1, 22.8)	664	63.0 (59.6, 66.5)	232	25.0 (21.9, 28.1)	101	12.0 (9.5, 14.5)	43	5.6 (3.7, 7.4)	142	17.9 (15.0, 20.8)	817	76.5 (73.3, 79.8)
$25,000 - $49,999	1219	83.2 (80.9, 85.5)	233	16.8 (14.5, 19.1)	1026	65.6 (62.7, 68.6)	325	25.8 (23.0, 28.5)	99	8.6 (6.8, 10.4)	30	2.4 (1.5, 3.4)	117	10.4 (8.4, 12.4)	1302	87.2 (85.0, 89.4)
$50,000 - $74,999	1168	83.5 (81.2, 85.8)	228	16.5 (14.2, 18.8)	1036	70.6 (67.8, 73.5)	271	21.4 (18.8, 23.9)	89	8.0 (6.2, 9.8)	25	2.5 (1.4, 3.6)	78	7.7 (5.8, 9.5)	1298	89.8 (87.8, 91.9)
$75,000 - $99,999	1003	82.1 (79.6, 84.7)	198	17.9 (15.3, 20.4)	887	70.5 (67.5, 73.6)	247	23.3 (20.5, 26.1)	64	6.2 (4.5, 7.8)	20	2.1 (1.0, 3.3)	55	5.8 (4.2, 7.4)	1128	92.0 (90.1, 94.0)
$100,000 - $149,999	1242	83.1 (80.9, 85.3)	248	16.9 (14.7, 19.1)	1105	71.2 (68.6, 73.9)	304	23.1 (20.6, 25.6)	79	5.7 (4.3, 7.0)	16	1.2 (0.6, 1.9)	55	4.9 (3.5, 6.3)	1420	93.9 (92.3, 95.4)
>$150,000	1610	79.1 (77.0, 81.2)	380	20.9 (18.8, 23.0)	1399	67.5 (65.1, 69.8)	453	24.8 (22.6, 27.0)	141	7.7 (6.4, 9.1)	11	0.7 (0.3, 1.2)	63	4.2 (3.1, 5.3)	1921	95.1 (93.9, 96.3)
**Census Region**																
New England	337	80.9 (76.3, 85.4)	72	19.1 (14.6, 23.7)	292	67.7 (62.3, 73.1)	87	24.3 (19.4, 29.2)	28	8.0 (4.6, 11.4)	4	1.3 (0.0, 2.7)	22	7.0 (3.9, 10.2)	381	91.6 (88.3, 95.0)
Mid-Atlantic	909	84.3 (81.8, 86.8)	162	15.7 (13.2, 18.2)	773	69.2 (66.0, 72.3)	224	21.6 (18.9, 24.4)	80	9.2 (7.0, 11.3)	14	1.6 (0.6, 2.5)	65	7.0 (5.2, 8.9)	997	91.4 (89.3, 93.4)
East-North Central	1034	81.9 (79.5, 84.2)	225	18.1 (15.8, 20.5)	908	69.8 (66.9, 72.7)	275	23.8 (21.1, 26.6)	71	6.4 (4.8, 8.0)	19	2.0 (1.0, 3.1)	55	5.3 (3.8, 6.7)	1186	92.7 (90.9, 94.5)
West-North Central	493	81.7 (78.2, 85.2)	108	18.3 (14.8, 21.8)	421	68.8 (64.6, 73.0)	145	23.9 (20.1, 27.6)	36	7.3 (4.7, 9.9)	15	3.5 (1.6, 5.4)	26	4.8 (2.8, 6.7)	560	91.7 (89.1, 94.4)
South Atlantic	1423	83.8 (81.8, 85.8)	264	16.2 (14.2, 18.2)	1247	70.6 (68.0, 73.1)	337	22.6 (20.2, 24.9)	102	6.8 (5.4, 8.3)	26	2.0 (1.2, 2.9)	98	8.6 (6.8, 10.4)	1569	89.4 (87.4, 91.3)
East-South Central	395	85.8 (82.2, 89.4)	67	14.2 (10.6, 17.8)	363	76.0 (71.4, 80.7)	75	18.9 (14.5, 23.4)	23	5.0 (2.8, 7.2)	6	1.3 (0.2, 2.5)	35	10.9 (7.1, 14.7)	416	87.8 (83.8, 91.7)
West-South Central	737	82.2 (79.3, 85.0)	158	17.8 (15.0, 20.7)	631	67.7 (64.1, 71.2)	187	22.5 (19.3, 25.6)	79	9.9 (7.6, 12.1)	19	2.5 (1.4, 3.7)	68	9.7 (7.3, 12.1)	814	87.8 (85.2, 90.4)
Mountain	629	84.2 (81.1, 87.3)	110	15.8 (12.7, 18.9)	539	67.6 (63.6, 71.6)	158	25.3 (21.6, 29.1)	42	7.1 (4.7, 9.4)	14	2.6 (1.0, 4.2)	43	7.1 (4.8, 9.4)	684	90.3 (87.6, 93.0)
Pacific	1086	74.4 (71.7, 77.2)	320	25.6 (22.8, 28.3)	943	60.8 (57.7, 63.8)	344	29.6 (26.6, 32.5)	112	9.7 (7.7, 11.7)	28	2.7 (1.5, 3.8)	98	9.3 (7.4, 11.3)	1279	88.0 (85.8, 90.2)

Table S3, continued

Characteristic	Democracy is the best form of government						These days, American democracy only serves the interest of the wealthy and powerful						Having a strong leader for America is more important than having a democracy.					
	Do not agree		Somewhat agree		Strongly or very strongly agree		Do not agree		Somewhat agree		Strongly or very strongly agree		Do not agree		Somewhat agree		Strongly or very strongly agree	
	Unweighted n	Weighted %, 95% CI	Unweighted n	Weighted %, 95% CI	Unweighted n	Weighted %, 95% CI	Unweighted n	Weighted %, 95% CI	Unweighted n	Weighted %, 95% CI	Unweighted n	Weighted %, 95% CI	Unweighted n	Weighted %, 95% CI	Unweighted n	Weighted %, 95% CI	Unweighted n	Weighted %, 95% CI
**Age, years**																		
18-24	42	10.5 (7.4, 13.6)	148	34.7 (29.9, 39.5)	242	54.8 (49.8, 59.9)	82	17.2 (13.6, 20.9)	157	36.9 (32.0, 41.8)	199	45.9 (40.9, 50.9)	219	47.1 (42.1, 52.1)	120	29.0 (24.4, 33.7)	95	23.9 (19.4, 28.4)
25-34	81	8.6 (6.6, 10.6)	349	35.2 (31.9, 38.4)	578	56.2 (52.8, 59.6)	176	18.4 (15.7, 21.0)	343	35.4 (32.1, 38.6)	488	46.3 (42.9, 49.7)	596	56.1 (52.7, 59.5)	239	25.3 (22.3, 28.3)	171	18.6 (15.9, 21.3)
35-44	100	7.9 (6.4, 9.5)	364	28.4 (25.7, 31.0)	886	63.7 (61.0, 66.5)	253	18.5 (16.3, 20.7)	520	39.3 (36.5, 42.1)	580	42.2 (39.4, 45.0)	770	54.9 (52.0, 57.7)	354	27.2 (24.6, 29.8)	227	17.9 (15.7, 20.2)
45-54	61	5.2 (3.8, 6.5)	255	21.9 (19.4, 24.4)	892	72.9 (70.2, 75.6)	314	25.8 (23.2, 28.4)	475	39.6 (36.7, 42.5)	418	34.6 (31.8, 37.4)	698	55.8 (52.8, 58.7)	284	25.2 (22.6, 27.8)	222	19.0 (16.7, 21.4)
55-64	73	4.2 (3.2, 5.2)	342	19.5 (17.5, 21.5)	1394	76.4 (74.2, 78.5)	598	32.1 (29.8, 34.3)	671	36.8 (34.4, 39.1)	545	31.2 (28.8, 33.5)	1097	59.0 (56.6, 61.5)	381	21.7 (19.6, 23.8)	330	19.3 (17.3, 21.3)
65-74	51	3.1 (2.2, 4)	232	13.6 (11.8, 15.3)	1485	83.3 (81.5, 85.2)	685	38.1 (35.7, 40.4)	599	34.2 (31.8, 36.5)	488	27.8 (25.5, 30.0)	1133	62.5 (60.1, 64.9)	307	18.0 (16.1, 19.9)	330	19.5 (17.5, 21.4)
75+	21	2.6 (1.3, 3.8)	67	8.0 (6.0, 9.9)	843	89.5 (87.2, 91.7)	458	47.5 (44.1, 50.9)	293	31.7 (28.5, 34.9)	180	20.8 (17.9, 23.7)	628	64.0 (60.5, 67.4)	150	18.4 (15.5, 21.2)	148	17.7 (14.9, 20.4)
**Gender**																		
Male	200	5.6 (4.8, 6.5)	746	21.1 (19.6, 22.6)	3165	73.3 (71.7, 74.9)	1320	28.5 (27.0, 30.0)	1438	36.1 (34.4, 37.8)	1360	35.3 (33.7, 37.0)	2674	60.9 (59.1, 62.6)	775	21.4 (19.9, 22.9)	656	17.8 (16.4, 19.2)
Female	208	6.2 (5.3, 7.1)	963	25.9 (24.3, 27.4)	3070	67.9 (66.3, 69.6)	1221	26.1 (24.7, 27.6)	1580	37.6 (35.9, 39.2)	1447	36.3 (34.7, 38.0)	2371	53.3 (51.6, 55.0)	1038	26.3 (24.8, 27.9)	830	20.4 (19.0, 21.8)
Other	16	14.9 (7.2, 22.6)	38	41.9 (31.2, 52.6)	52	43.2 (32.8, 53.5)	10	5.4 (1.9, 8.8)	24	21.6 (12.6, 30.6)	72	73.0 (63.7, 82.3)	67	60.8 (50.1, 71.5)	17	18.6 (9.7, 27.4)	22	20.6 (11.6, 29.6)
**Race and ethnicity**																		
White, non-Hispanic	261	5.2 (4.5, 5.9)	1050	20.0 (18.8, 21.1)	4668	74.8 (73.6, 76.0)	1997	30.5 (29.2, 31.7)	2086	35.2 (33.9, 36.5)	1904	34.3 (33.0, 35.6)	3847	62.6 (61.3, 63.9)	1168	20.8 (19.6, 21.9)	962	16.6 (15.6, 17.7)
Black, non-Hispanic	54	7.5 (5.3, 9.7)	258	35.2 (31.4, 39.1)	497	57.2 (53.3, 61.2)	152	17.6 (14.7, 20.5)	327	40.6 (36.7, 44.5)	338	41.8 (38.0, 45.7)	400	45.0 (41.1, 48.9)	207	28.4 (24.8, 32.0)	205	26.6 (23.0, 30.2)
Hispanic, any race	73	7.7 (5.9, 9.6)	280	29.0 (25.9, 32.1)	715	63.3 (60.0, 66.6)	273	23.9 (21.1, 26.7)	394	36.8 (33.6, 40.0)	401	39.3 (36.0, 42.6)	544	49.1 (45.8, 52.4)	287	28.7 (25.6, 31.8)	235	22.2 (19.4, 25.0)
Asian American / Pacific Islander	18	6.7 (3.3, 10.2)	67	21.7 (16.5, 26.8)	224	71.6 (65.9, 77.3)	63	20.7 (15.5, 25.8)	136	41.4 (35.2, 47.5)	111	38.0 (31.8, 44.2)	154	48.3 (41.9, 54.6)	90	30.5 (24.6, 36.5)	63	21.2 (15.9, 26.5)
Other (American Indian/Alaskan, 2+ races, other)	23	9.6 (4.7, 14.6)	102	35.0 (27.5, 42.6)	216	55.4 (47.6, 63.1)	81	18.5 (13.3, 23.8)	115	41.1 (33.2, 49.0)	144	40.3 (33.0, 47.6)	196	46.3 (38.8, 53.8)	83	28.6 (21.4, 35.9)	58	25.1 (17.6, 32.6)
**Education**																		
No high school diploma or GED	60	12.2 (9, 15.4)	172	34.0 (29.5, 38.5)	292	53.8 (49.1, 58.5)	110	20.5 (16.7, 24.3)	211	40.7 (36.1, 45.3)	210	38.7 (34.2, 43.3)	232	42.9 (38.2, 47.5)	145	28.5 (24.3, 32.8)	149	28.6 (24.3, 33.0)
High school graduate	157	8.1 (6.8, 9.5)	536	28.8 (26.6, 31.1)	1419	63 (60.6, 65.4)	580	25.4 (23.3, 27.4)	753	36.2 (33.9, 38.6)	782	38.4 (36.0, 40.7)	1011	46.0 (43.6, 48.4)	575	29.6 (27.3, 31.8)	519	24.5 (22.4, 26.6)
Some college or Associates degree	111	5.8 (4.7, 7)	469	22.3 (20.4, 24.3)	1753	71.8 (69.7, 73.9)	663	25.7 (23.8, 27.6)	845	36.0 (33.8, 38.2)	826	38.3 (36.1, 40.5)	1337	55.3 (53.0, 57.5)	543	24.5 (22.5, 26.5)	455	20.3 (18.4, 22.1)
Bachelors degree	66	3.7 (2.7, 4.7)	350	20.3 (18.2, 22.3)	1525	76.0 (73.9, 78.2)	642	29.4 (27.3, 31.6)	679	36.3 (33.9, 38.6)	622	34.3 (32.0, 36.6)	1344	67.7 (65.4, 70.0)	349	19.1 (17.1, 21.1)	245	13.2 (11.6, 14.9)
Masters degree or higher	35	2.4 (1.6, 3.3)	230	16.1 (14.0, 18.1)	1331	81.5 (79.3, 83.7)	571	32.5 (30.1, 34.9)	570	36.5 (33.9, 39.1)	458	31.0 (28.4, 33.5)	1217	74.5 (72.1, 76.9)	223	15.3 (13.3, 17.3)	155	10.2 (8.5, 11.9)
**Income**																		
<$25,000	108	13 (10.5, 15.6)	299	34.0 (30.6, 37.5)	588	52.9 (49.4, 56.5)	221	20.7 (17.9, 23.5)	361	35.7 (32.3, 39.1)	418	43.6 (40.1, 47.1)	456	43.3 (39.9, 46.8)	272	29.0 (25.8, 32.3)	267	27.7 (24.5, 30.8)
$25,000 - $49,999	98	8.1 (6.4, 9.8)	343	27.3 (24.5, 30.0)	1000	64.6 (61.7, 67.5)	362	21.1 (18.8, 23.3)	507	37.0 (34.1, 40.0)	578	41.9 (38.9, 44.8)	760	49.5 (46.5, 52.5)	345	25.4 (22.8, 28.1)	337	25.1 (22.4, 27.8)
$50,000 - $74,999	70	6 (4.4, 7.6)	285	24.5 (21.8, 27.3)	1038	69.5 (66.5, 72.4)	382	24.6 (22.1, 27.1)	526	38.1 (35.2, 41.0)	491	37.3 (34.4, 40.3)	798	53.0 (50.0, 56.1)	326	26.1 (23.3, 28.9)	269	20.9 (18.3, 23.4)
$75,000 - $99,999	49	5.5 (3.9, 7.2)	229	21.8 (19.0, 24.5)	921	72.7 (69.7, 75.7)	368	28.1 (25.4, 30.9)	430	35.7 (32.6, 38.7)	399	36.2 (33.1, 39.3)	709	55.5 (52.4, 58.7)	286	26.7 (23.8, 29.7)	201	17.7 (15.2, 20.2)
$100,000 - $149,999	53	3.9 (2.8, 5.1)	277	21.7 (19.2, 24.2)	1161	74.4 (71.8, 77.0)	464	28.1 (25.6, 30.6)	552	37.3 (34.6, 40.1)	476	34.6 (31.8, 37.3)	985	63.0 (60.2, 65.8)	283	21.2 (18.8, 23.6)	219	15.8 (13.6, 18.0)
>$150,000	51	3 (2.1, 3.9)	324	18.2 (16.2, 20.1)	1612	78.9 (76.8, 80.9)	769	34.8 (32.6, 37.0)	682	35.9 (33.5, 38.2)	536	29.3 (27.1, 31.6)	1433	68.9 (66.6, 71.3)	323	18.3 (16.3, 20.3)	230	12.8 (11.1, 14.5)
**Census Region**																		
New England	18	5.8 (3.1, 8.6)	73	23.7 (18.5, 28.9)	315	70.5 (65.1, 75.9)	112	23.5 (19.1, 27.9)	160	40.9 (35.4, 46.3)	135	35.6 (30.3, 40.9)	252	60.3 (54.9, 65.8)	79	21.3 (16.7, 25.8)	74	18.4 (14.1, 22.7)
Mid-Atlantic	48	4.8 (3.3, 6.3)	208	21.8 (18.9, 24.7)	819	73.5 (70.4, 76.5)	334	29.5 (26.5, 32.5)	383	35.7 (32.5, 39.0)	355	34.7 (31.5, 38.0)	637	56.8 (53.4, 60.2)	220	22.4 (19.5, 25.4)	215	20.8 (18.0, 23.5)
East-North Central	56	4.9 (3.5, 6.4)	247	22.2 (19.6, 24.9)	955	72.8 (70.0, 75.7)	353	25.5 (22.9, 28.0)	474	38.5 (35.6, 41.5)	434	36.0 (33.0, 39.0)	767	57.1 (54.1, 60.2)	269	23.7 (21.0, 26.5)	223	19.1 (16.7, 21.6)
West-North Central	28	6.4 (3.8, 9.1)	127	22.6 (18.8, 26.4)	445	71.0 (66.7, 75.2)	168	26.8 (22.9, 30.8)	231	38.1 (33.8, 42.4)	202	35.0 (30.8, 39.2)	366	59.0 (54.6, 63.5)	125	22.5 (18.6, 26.4)	108	18.4 (15.0, 21.9)
South Atlantic	75	5.6 (4.2, 6.9)	326	23.2 (20.8, 25.6)	1279	71.3 (68.7, 73.8)	546	28.0 (25.7, 30.3)	578	36.0 (33.4, 38.6)	560	36.0 (33.5, 38.6)	1058	59.1 (56.4, 61.8)	329	21.6 (19.3, 23.9)	295	19.4 (17.1, 21.6)
East-South Central	35	9.2 (5.9, 12.5)	101	26.4 (21.6, 31.2)	319	64.4 (59.3, 69.6)	135	27.4 (22.9, 32.0)	157	34.0 (29.1, 38.9)	165	38.6 (33.5, 43.7)	244	51.2 (46.0, 56.4)	101	22.6 (18.3, 26.9)	111	26.1 (21.4, 30.8)
West-South Central	46	6.6 (4.5, 8.6)	196	24.6 (21.3, 27.9)	648	68.8 (65.3, 72.4)	302	30.1 (26.9, 33.4)	294	33.5 (30.0, 37.0)	298	36.4 (32.8, 40.0)	506	54.7 (51.0, 58.4)	229	27.6 (24.2, 31.0)	154	17.6 (14.8, 20.5)
Mountain	38	6.5 (4.2, 8.8)	162	25.2 (21.5, 28.9)	540	68.3 (64.4, 72.3)	221	28.3 (24.7, 31.9)	256	35.3 (31.4, 39.2)	264	36.4 (32.5, 40.4)	457	58.5 (54.4, 62.5)	155	22.8 (19.3, 26.3)	126	18.7 (15.4, 22.0)
Pacific	85	7.3 (5.6, 9)	317	26.3 (23.4, 29.1)	1000	66.5 (63.5, 69.5)	395	22.8 (20.4, 25.1)	525	38.6 (35.6, 41.6)	485	38.6 (35.6, 41.6)	854	55.5 (52.4, 58.6)	328	26.9 (24.1, 29.7)	217	17.6 (15.1, 20.1)

Table S3, continued

Characteristic	The 2020 election was stolen from Donald Trump, and Joe Biden is an illegitimate president.						Armed citizens should patrol polling places at election time					
	Do not agree		Somewhat agree		Strongly or very strongly agree		Do not agree		Somewhat agree		Strongly or very strongly agree	
	Unweighted n	Weighted %, 95% CI	Unweighted n	Weighted %, 95% CI	Unweighted n	Weighted %, 95% CI	Unweighted n	Weighted %, 95% CI	Unweighted n	Weighted %, 95% CI	Unweighted n	Weighted %, 95% CI
**Age, years**												
18-24	293	67.4 (62.7, 72.1)	69	16.0 (12.3, 19.8)	76	16.6 (12.8, 20.3)	358	80.2 (76.1, 84.4)	50	12.0 (8.6, 15.4)	32	7.7 (4.9, 10.5)
25-34	716	68.7 (65.5, 71.9)	130	14.2 (11.7, 16.7)	167	17.1 (14.5, 19.7)	811	78.1 (75.2, 81.0)	109	11.4 (9.2, 13.7)	91	10.5 (8.3, 12.7)
35-44	940	69.0 (66.4, 71.7)	166	12.7 (10.8, 14.6)	247	18.3 (16.1, 20.5)	1123	81.9 (79.6, 84.1)	132	10.8 (8.9, 12.7)	97	7.3 (5.9, 8.8)
45-54	793	65.9 (63.1, 68.7)	169	14.1 (12.1, 16.2)	234	20.0 (17.6, 22.4)	1027	85.3 (83.2, 87.4)	97	8.5 (6.9, 10.2)	73	6.1 (4.7, 7.6)
55-64	1196	65.6 (63.3, 68.0)	261	14.8 (13.1, 16.6)	346	19.5 (17.5, 21.5)	1587	87.2 (85.4, 89.0)	138	8.6 (7.1, 10.1)	75	4.2 (3.2, 5.2)
65-74	1210	68.1 (65.8, 70.4)	215	12.1 (10.5, 13.7)	337	19.7 (17.7, 21.7)	1552	87.3 (85.6, 88.9)	119	7.4 (6.0, 8.7)	92	5.4 (4.3, 6.5)
75+	613	65.8 (62.5, 69.1)	132	14.3 (11.9, 16.8)	174	19.9 (17.0, 22.7)	810	86.5 (84.0, 89.0)	76	9.2 (7.0, 11.4)	37	4.2 (2.8, 5.6)
**Gender**												
Male	2767	66.7 (65.1, 68.4)	570	14.6 (13.3, 15.9)	764	18.6 (17.3, 20.0)	3541	84.4 (83.1, 85.8)	334	9 (8, 10.1)	229	6.5 (5.6, 7.4)
Female	2885	67.7 (66.2, 69.3)	550	13.2 (12.1, 14.4)	797	19.1 (17.7, 20.4)	3604	83.0 (81.6, 84.3)	372	10.3 (9.2, 11.4)	254	6.7 (5.8, 7.6)
Other	81	76.6 (67.0, 86.2)	16	17.3 (8.2, 26.5)	8	6.0 (1.7, 10.3)	84	81.0 (72.3, 89.7)	13	12.3 (4.7, 20)	8	6.7 (1.6, 11.7)
**Race and ethnicity**												
White, non-Hispanic	3839	62.5 (61.2, 63.9)	873	15.2 (14.2, 16.2)	1254	22.2 (21.1, 23.4)	5177	85.4 (84.4, 86.4)	483	8.8 (8, 9.6)	313	5.8 (5.1, 6.5)
Black, non-Hispanic	699	83.8 (80.6, 87.0)	63	10.5 (7.7, 13.3)	45	5.7 (3.8, 7.6)	674	80.0 (76.5, 83.4)	78	12.4 (9.5, 15.4)	53	7.6 (5.3, 9.8)
Hispanic, any race	785	74.3 (71.3, 77.2)	119	11.7 (9.5, 13.9)	158	14.0 (11.8, 16.3)	877	81.6 (79.0, 84.2)	102	10.3 (8.2, 12.4)	84	8.1 (6.3, 9.8)
Asian American / Pacific Islander	227	72.5 (66.7, 78.3)	37	11.3 (7.4, 15.2)	44	16.2 (11.2, 21.2)	252	80.9 (75.7, 86.1)	30	10.7 (6.6, 14.8)	24	8.4 (4.7, 12.1)
Other (American Indian/Alaskan, 2+ races, other)	211	57.0 (49.2, 64.7)	50	17.4 (11.4, 23.4)	80	25.7 (18.5, 32.8)	288	77.5 (70.1, 84.9)	28	13 (6.9, 19.2)	23	9.5 (4.2, 14.8)
**Education**												
No high school diploma or GED	318	60.7 (56.1, 65.4)	94	19.5 (15.6, 23.3)	112	19.8 (16.1, 23.5)	373	70.1 (65.8, 74.5)	89	17.4 (13.8, 20.9)	63	12.5 (9.3, 15.7)
High school graduate	1184	57.6 (55.2, 60.0)	333	15.6 (13.8, 17.4)	586	26.8 (24.7, 28.9)	1627	75.9 (73.7, 78.0)	283	14.4 (12.7, 16.2)	192	9.7 (8.2, 11.2)
Some college or Associates degree	1472	63.7 (61.5, 65.9)	350	15.4 (13.7, 17.0)	504	20.9 (19.1, 22.7)	2007	85.5 (83.9, 87.1)	178	8.3 (7.0, 9.7)	139	6.2 (5.1, 7.2)
Bachelors degree	1481	77.5 (75.5, 79.5)	231	11.5 (10.0, 13.0)	226	11.0 (9.6, 12.5)	1765	90.7 (89.3, 92.2)	109	5.7 (4.6, 6.9)	68	3.5 (2.6, 4.5)
Masters degree or higher	1306	82.4 (80.4, 84.4)	134	8.2 (6.7, 9.7)	153	9.4 (7.8, 10.9)	1496	93.5 (92.1, 94.8)	62	4.0 (3, 5.1)	35	2.5 (1.6, 3.4)
**Income**												
<$25,000	587	57.9 (54.4, 61.4)	154	16.9 (14.1, 19.7)	254	25.2 (22.2, 28.2)	726	69.2 (65.8, 72.5)	144	16.6 (13.9, 19.4)	129	14.2 (11.7, 16.7)
$25,000 - $49,999	913	64.7 (61.9, 67.6)	189	12.8 (10.8, 14.7)	335	22.5 (20.0, 24.9)	1157	78.1 (75.6, 80.7)	157	12.4 (10.4, 14.5)	122	9.4 (7.6, 11.3)
$50,000 - $74,999	921	66.3 (63.5, 69.2)	197	13.6 (11.5, 15.6)	268	20.1 (17.6, 22.6)	1185	82.5 (79.9, 85.0)	126	11.1 (9, 13.3)	75	6.4 (4.8, 8)
$75,000 - $99,999	816	67.1 (64.1, 70.2)	164	15.1 (12.7, 17.5)	218	17.8 (15.4, 20.1)	1040	85.1 (82.7, 87.5)	91	8.5 (6.6, 10.4)	68	6.5 (4.8, 8.1)
$100,000 - $149,999	1037	68.5 (65.8, 71.2)	205	14.4 (12.4, 16.5)	245	17.1 (15.0, 19.2)	1336	89.5 (87.8, 91.3)	99	6.7 (5.3, 8.2)	52	3.7 (2.6, 4.9)
>$150,000	1487	74.6 (72.4, 76.7)	233	12.3 (10.7, 14.0)	261	13.1 (11.5, 14.7)	1824	91.2 (89.8, 92.7)	104	5.8 (4.6, 7.1)	51	2.9 (2.1, 3.8)
**Census Region**												
New England	304	76.1 (71.5, 80.8)	39	9.9 (6.4, 13.3)	63	14.0 (10.4, 17.6)	355	87.5 (83.9, 91.1)	28	7.5 (4.4, 10.6)	24	5 (2.9, 7.1)
Mid-Atlantic	719	68.0 (64.9, 71.1)	159	15.3 (12.9, 17.8)	190	16.7 (14.2, 19.1)	917	84.0 (81.3, 86.7)	92	10.2 (7.9, 12.4)	57	5.8 (4.1, 7.6)
East-North Central	853	66.9 (64.0, 69.8)	172	15.1 (12.8, 17.4)	225	18.0 (15.8, 20.3)	1092	85.1 (82.8, 87.5)	95	8.8 (6.8, 10.7)	68	6.1 (4.6, 7.6)
West-North Central	384	62.6 (58.3, 67.0)	87	15.0 (11.8, 18.2)	129	22.3 (18.6, 26.1)	508	83.7 (80.3, 87.1)	55	10 (7.2, 12.8)	33	6.3 (4.1, 8.5)
South Atlantic	1104	64.9 (62.3, 67.5)	242	14.7 (12.8, 16.7)	329	20.4 (18.2, 22.6)	1423	81.5 (79.2, 83.8)	155	11.1 (9.2, 12.9)	103	7.5 (5.9, 9)
East-South Central	265	55.0 (49.8, 60.2)	73	16.6 (12.8, 20.5)	117	28.4 (23.6, 33.2)	383	81.1 (76.8, 85.4)	38	9.3 (6.2, 12.4)	35	9.6 (6.2, 13.0)
West-South Central	563	63.6 (60.0, 67.1)	140	16.3 (13.5, 19.1)	194	20.1 (17.3, 23.0)	744	82.1 (79.2, 85.1)	84	10.4 (8, 12.8)	61	7.5 (5.5, 9.5)
Mountain	496	68.9 (65.2, 72.6)	94	11.6 (9.2, 14.1)	146	19.5 (16.3, 22.7)	640	87.1 (84.4, 89.8)	64	9 (6.6, 11.3)	34	3.9 (2.5, 5.3)
Pacific	1073	76.3 (73.7, 78.9)	136	10.0 (8.1, 11.9)	188	13.7 (11.7, 15.8)	1206	84.1 (81.8, 86.4)	110	9 (7.2, 10.8)	82	7 (5.4, 8.6)

Table S4. Variation with respondent characteristics in beliefs concerning the potential need for violence in the US

Characteristic	If elected leaders will not protect American democracy, the people must do it themselves, even if it requires taking violent actions.						Our American way of life is disappearing so fast that we may have to use force to save it.					
	Do not agree		Somewhat agree		Strongly or very strongly agree		Do not agree		Somewhat agree		Strongly or very strongly agree	
	Unweighted n	Weighted %, 95% CI	Unweighted n	Weighted %, 95% CI	Unweighted n	Weighted %, 95% CI	Unweighted n	Weighted %, 95% CI	Unweighted n	Weighted %, 95% CI	Unweighted n	Weighted %, 95% CI
**Age, years**												
18-24	166	39.6 (34.7, 44.6)	153	34.2 (29.5, 39.0)	118	26.1 (21.7, 30.5)	222	50.3 (45.3, 55.3)	135	30.2 (25.6, 34.7)	80	19.5 (15.4, 23.7)
25-34	395	39.8 (36.5, 43.1)	379	37.0 (33.8, 40.3)	232	23.2 (20.3, 26.1)	623	59.2 (55.8, 62.5)	236	24.5 (21.6, 27.4)	149	16.3 (13.7, 18.9)
35-44	643	48.4 (45.6, 51.2)	437	31.7 (29.1, 34.3)	271	19.9 (17.6, 22.2)	789	56.9 (54.1, 59.7)	334	25.8 (23.3, 28.3)	231	17.3 (15.1, 19.5)
45-54	652	54.2 (51.2, 57.1)	348	29.1 (26.4, 31.8)	201	16.7 (14.5, 18.9)	670	55.1 (52.2, 58.1)	327	28.1 (25.4, 30.8)	204	16.8 (14.6, 19.0)
55-64	1025	56.3 (53.9, 58.8)	511	28.4 (26.2, 30.7)	270	15.3 (13.5, 17.0)	1061	56.9 (54.4, 59.4)	480	28.1 (25.8, 30.4)	264	15.0 (13.2, 16.8)
65-74	1062	59.9 (57.5, 62.3)	437	24.5 (22.4, 26.6)	272	15.6 (13.8, 17.4)	1042	57.5 (55.1, 60.0)	470	27.3 (25.1, 29.5)	260	15.2 (13.4, 16.9)
75+	561	60.4 (57.0, 63.8)	203	21.9 (19.1, 24.8)	157	17.6 (14.9, 20.4)	552	57.8 (54.3, 61.2)	240	27.9 (24.7, 31.1)	127	14.3 (11.9, 16.8)
**Gender**												
Male	1968	46.5 (44.7, 48.2)	1298	32.6 (30.9, 34.2)	832	21.0 (19.5, 22.4)	2487	58.4 (56.7, 60.2)	1000	25.4 (23.9, 26.9)	614	16.1 (14.8, 17.5)
Female	2478	55.8 (54.1, 57.5)	1120	27.6 (26.1, 29.1)	642	16.6 (15.3, 17.9)	2380	54.5 (52.8, 56.1)	1189	29.1 (27.6, 30.7)	672	16.4 (15.1, 17.7)
Other	31	23.8 (15.4, 32.2)	35	36.6 (26.0, 47.2)	40	39.6 (29.2, 50.0)	60	60.6 (50.2, 71.0)	26	21.4 (13.3, 29.5)	19	18.0 (9.2, 26.8)
**Race and ethnicity**												
White, non-Hispanic	3082	48.9 (47.6, 50.3)	1828	32.0 (30.7, 33.3)	1059	19.1 (18.0, 20.2)	3458	56.1 (54.8, 57.5)	1574	27.4 (26.2, 28.7)	934	16.5 (15.4, 17.5)
Black, non-Hispanic	495	57.6 (53.6, 61.5)	176	23.9 (20.5, 27.4)	139	18.5 (15.3, 21.7)	471	54.0 (50.1, 58.0)	222	28.5 (24.9, 32.0)	121	17.5 (14.3, 20.7)
Hispanic, any race	592	53.9 (50.6, 57.3)	289	28.8 (25.8, 31.9)	186	17.2 (14.7, 19.7)	638	58.9 (55.6, 62.2)	285	26.6 (23.6, 29.5)	147	14.5 (12.2, 16.9)
Asian American / Pacific Islander	171	56.0 (49.7, 62.3)	78	24.1 (18.8, 29.5)	59	19.8 (14.6, 25.0)	207	64.5 (58.3, 70.7)	63	22.8 (17.3, 28.4)	37	12.7 (8.2, 17.1)
Other (American Indian/Alaskan, 2+ races, other)	164	39.7 (32.4, 47.0)	97	32.0 (24.8, 39.2)	78	28.3 (20.9, 35.7)	185	46.6 (39.0, 54.2)	78	27.6 (20.6, 34.6)	76	25.8 (18.6, 33.0)
**Education**												
No high school diploma or GED	261	48.0 (43.3, 52.7)	152	29.5 (25.3, 33.8)	116	22.5 (18.5, 26.5)	238	45.6 (40.9, 50.3)	173	31.4 (27.1, 35.7)	118	23.0 (19.0, 27.0)
High school graduate	1016	46.3 (43.9, 48.7)	642	31.4 (29.1, 33.6)	450	22.3 (20.2, 24.3)	885	41.8 (39.4, 44.2)	716	34.6 (32.3, 36.9)	506	23.6 (21.5, 25.7)
Some college or Associates degree	1224	50.9 (48.6, 53.1)	656	28.8 (26.7, 30.9)	450	20.3 (18.5, 22.2)	1266	54.2 (51.9, 56.4)	669	28.9 (26.8, 31.0)	393	16.9 (15.2, 18.7)
Bachelors degree	1061	53.4 (51.0, 55.8)	582	31.1 (28.8, 33.4)	292	15.5 (13.7, 17.3)	1334	69.3 (67.0, 71.5)	420	21.2 (19.2, 23.1)	180	9.6 (8.1, 11.0)
Masters degree or higher	942	57.8 (55.1, 60.4)	436	28.9 (26.5, 31.4)	213	13.3 (11.5, 15.1)	1236	77.2 (75.0, 79.5)	244	15.9 (13.9, 17.9)	118	6.9 (5.6, 8.2)
**Income**												
<$25,000	481	46.1 (42.7, 49.6)	300	31.2 (28.0, 34.5)	218	22.6 (19.7, 25.5)	418	39.3 (35.9, 42.7)	335	35.2 (31.8, 38.5)	245	25.5 (22.4, 28.6)
$25,000 - $49,999	705	47.3 (44.3, 50.3)	428	29.9 (27.2, 32.6)	304	22.8 (20.2, 25.4)	722	50.2 (47.3, 53.2)	420	29.2 (26.5, 31.8)	302	20.6 (18.2, 23)
$50.0,000 - $74,999	737	50.2 (47.2, 53.2)	390	29.0 (26.3, 31.8)	267	20.8 (18.3, 23.3)	753	52.0 (49.0, 55.0)	410	30.0 (27.2, 32.8)	229	18.0 (15.6, 20.4)
$75,000 - $99,999	622	49.7 (46.5, 52.9)	363	31.7 (28.7, 34.7)	211	18.7 (16.1, 21.2)	706	56.6 (53.5, 59.8)	315	28.0 (25.1, 30.9)	176	15.4 (13.1, 17.7)
$100.0,000 - $149,999	815	51.8 (48.9, 54.7)	419	30.1 (27.4, 32.7)	251	18.1 (15.8, 20.4)	943	61.0 (58.2, 63.9)	358	25.8 (23.2, 28.3)	182	13.2 (11.2, 15.3)
>$150.0,000	1144	56.7 (54.3, 59.1)	568	29.4 (27.2, 31.6)	270	13.9 (12.2, 15.5)	1417	70.3 (68.1, 72.6)	384	19.8 (17.9, 21.7)	181	9.9 (8.3, 11.4)
**Census Region**												
New England	235	56.1 (50.6, 61.6)	108	28.1 (23.1, 33.1)	63	15.8 (11.7, 20.0)	248	60.6 (55.1, 66.0)	97	25.7 (20.6, 30.7)	59	13.8 (10.1, 17.5)
Mid-Atlantic	588	53.2 (49.8, 56.5)	308	30.2 (27.1, 33.4)	175	16.6 (14.1, 19.1)	602	55.4 (52.1, 58.7)	297	28.0 (25.0, 31.1)	173	16.6 (14.1, 19.1)
East-North Central	671	51.0 (47.9, 54.0)	355	28.7 (25.9, 31.4)	229	20.4 (17.8, 23.0)	757	58.2 (55.2, 61.3)	307	25.1 (22.4, 27.8)	189	16.7 (14.3, 19.1)
West-North Central	305	48.2 (43.8, 52.7)	183	31.7 (27.6, 35.9)	111	20.0 (16.3, 23.7)	327	52.8 (48.4, 57.2)	177	31.3 (27.2, 35.4)	96	15.9 (12.8, 19.0)
South Atlantic	883	50.1 (47.4, 52.8)	499	31.1 (28.6, 33.6)	298	18.8 (16.6, 20.9)	975	54.7 (52.0, 57.4)	444	28.1 (25.7, 30.6)	267	17.1 (15.0, 19.3)
East-South Central	234	49.2 (44.0, 54.5)	140	32.1 (27.2, 37.0)	79	18.6 (14.5, 22.8)	234	47.2 (42.0, 52.3)	142	32.2 (27.4, 37.1)	82	20.6 (16.2, 25.0)
West-South Central	456	49.5 (45.8, 53.2)	259	29.6 (26.2, 33.0)	177	20.9 (17.9, 24.0)	484	53.6 (49.9, 57.2)	256	27.9 (24.6, 31.2)	153	18.5 (15.5, 21.6)
Mountain	353	46.1 (42.1, 50.2)	233	33.2 (29.4, 37.1)	152	20.7 (17.4, 23.9)	420	57.0 (52.9, 61.0)	196	27.2 (23.6, 30.9)	121	15.8 (12.9, 18.7)
Pacific	779	53.6 (50.5, 56.6)	383	27.9 (25.2, 30.7)	237	18.5 (16.1, 20.9)	912	63.6 (60.6, 66.6)	306	23.4 (20.7, 26.1)	175	12.9 (10.9, 15.0)

Table S4, continued

Characteristic	Because things have gotten so far off track, true American patriots may have to resort to violence in order to save our country.						In the next few years, there will be civil war in the United States.					
	Do not agree		Somewhat agree		Strongly or very strongly agree		Do not agree		Somewhat agree		Strongly or very strongly agree	
	Unweighted n	Weighted %, 95% CI	Unweighted n	Weighted %, 95% CI	Unweighted n	Weighted %, 95% CI	Unweighted n	Weighted %, 95% CI	Unweighted n	Weighted %, 95% CI	Unweighted n	Weighted %, 95% CI
**Age, years**												
18-24	300	67.4 (62.6, 72.2)	95	22.8 (18.5, 27.1)	40	9.8 (6.7, 12.9)	191	43.6 (38.6, 48.6)	165	38.1 (33.2, 43.0)	75	18.3 (14.3, 22.3)
25-34	728	70.2 (67.0, 73.3)	178	19.5 (16.7, 22.3)	102	10.3 (8.3, 12.3)	466	45.0 (41.6, 48.4)	367	37.2 (34.0, 40.5)	169	17.7 (15.1, 20.4)
35-44	990	72.6 (70.0, 75.1)	231	17.4 (15.2, 19.6)	134	10.1 (8.3, 11.8)	654	47.5 (44.7, 50.4)	482	35.8 (33.1, 38.5)	213	16.7 (14.5, 18.8)
45-54	905	75.6 (73.1, 78.2)	188	16.0 (13.8, 18.2)	102	8.3 (6.7, 9.9)	586	48.6 (45.6, 51.6)	445	37.3 (34.4, 40.1)	167	14.1 (12.0, 16.2)
55-64	1375	76.1 (74.0, 78.3)	314	17.8 (15.9, 19.7)	109	6.1 (4.9, 7.3)	917	50.9 (48.4, 53.4)	684	38.2 (35.8, 40.6)	194	10.8 (9.3, 12.4)
65-74	1386	77.8 (75.7, 79.9)	268	15.8 (14, 17.7)	109	6.4 (5.2, 7.6)	923	51.9 (49.5, 54.4)	663	37.5 (35.1, 39.9)	180	10.6 (9.0, 12.1)
75+	720	77.0 (74.0, 80.0)	149	16.9 (14.2, 19.6)	52	6.1 (4.3, 7.8)	531	56.1 (52.6, 59.5)	320	36.2 (32.8, 39.5)	67	7.8 (5.9, 9.7)
**Gender**												
Male	3124	74.2 (72.7, 75.8)	661	17.4 (16, 18.8)	322	8.4 (7.4, 9.4)	2282	54.4 (52.6, 56.1)	1362	33.3 (31.7, 35.0)	448	12.3 (11.1, 13.5)
Female	3177	73.5 (72.0, 75.1)	727	18.3 (17, 19.6)	312	8.2 (7.2, 9.1)	1924	43.9 (42.3, 45.6)	1704	40.7 (39.0, 42.4)	585	15.4 (14.1, 16.7)
Other	69	69.1 (59.6, 78.6)	26	23.3 (14.7, 32)	10	7.6 (2.4, 12.7)	35	31.2 (21.4, 40.9)	43	44.8 (34.1, 55.5)	27	24.0 (15.1, 32.9)
**Race and ethnicity**												
White, non-Hispanic	4493	73.6 (72.4, 74.8)	1033	18.5 (17.4, 19.6)	436	7.9 (7.1, 8.6)	3013	49.1 (47.8, 50.5)	2242	38.0 (36.7, 39.4)	690	12.8 (11.9, 13.8)
Black, non-Hispanic	625	74.1 (70.5, 77.8)	113	16.2 (13.1, 19.3)	69	9.7 (7.2, 12.1)	356	42.0 (38.1, 45.9)	311	38.2 (34.4, 42.0)	143	19.8 (16.5, 23.0)
Hispanic, any race	806	74.7 (71.7, 77.7)	170	17.1 (14.5, 19.7)	84	8.2 (6.4, 10.0)	566	51.7 (48.4, 55.1)	359	34.4 (31.2, 37.6)	135	13.8 (11.5, 16.2)
Asian American / Pacific Islander	241	76.3 (70.6, 82.0)	43	15.5 (10.6, 20.4)	22	8.2 (4.4, 12)	186	57.1 (50.7, 63.5)	87	30.7 (24.6, 36.7)	34	12.2 (7.9, 16.6)
Other (American Indian/Alaskan, 2+ races, other)	239	67.6 (60.2, 75.0)	64	20.2 (13.7, 26.7)	37	12.2 (7, 17.3)	147	36.4 (29.3, 43.5)	127	42.4 (34.7, 50.1)	63	21.2 (14.6, 27.8)
**Education**												
No high school diploma or GED	324	61.3 (56.6, 65.9)	133	26.4 (22.2, 30.7)	66	12.3 (9.2, 15.4)	222	43.0 (38.3, 47.7)	212	38.9 (34.3, 43.4)	94	18.1 (14.4, 21.8)
High school graduate	1349	64.3 (62.0, 66.6)	509	24.2 (22.1, 26.3)	236	11.5 (10.0, 13.1)	851	39.7 (37.3, 42.1)	841	40.1 (37.7, 42.5)	400	20.2 (18.2, 22.2)
Some college or Associates degree	1714	73.0 (70.9, 75.0)	416	18.3 (16.5, 20.1)	196	8.7 (7.4, 10.0)	1073	45.9 (43.6, 48.1)	934	40.4 (38.1, 42.6)	307	13.8 (12.2, 15.4)
Bachelors degree	1604	82.8 (80.9, 84.6)	246	12.7 (11.1, 14.4)	86	4.5 (3.5, 5.5)	1118	56.8 (54.4, 59.2)	640	33.4 (31.1, 35.7)	175	9.8 (8.3, 11.4)
Masters degree or higher	1413	88.3 (86.5, 90.0)	119	7.5 (6.1, 8.9)	64	4.2 (3.1, 5.4)	1004	63.3 (60.7, 65.8)	499	30.4 (28.0, 32.9)	89	6.3 (4.9, 7.7)
**Income**												
<$25,000	617	59.6 (56.2, 63.1)	238	26.2 (23.0, 29.4)	138	14.1 (11.7, 16.5)	385	37.4 (34, 40.8)	405	41.2 (37.7, 44.6)	204	21.4 (18.5, 24.3)
$25,000 - $49,999	982	67.6 (64.8, 70.5)	308	21.4 (18.9, 23.8)	145	11.0 (9.1, 12.9)	648	42.7 (39.8, 45.7)	549	39.7 (36.8, 42.7)	238	17.5 (15.3, 19.8)
$50.0,000 - $74,999	1021	71.2 (68.3, 74.1)	232	18.1 (15.7, 20.6)	132	10.7 (8.7, 12.7)	670	46.6 (43.6, 49.6)	527	37.6 (34.7, 40.5)	189	15.7 (13.3, 18.1)
$75,000 - $99,999	918	75.2 (72.4, 78.0)	204	17.7 (15.3, 20.2)	77	7.1 (5.4, 8.8)	576	47.4 (44.2, 50.6)	465	38.5 (35.4, 41.6)	144	14.1 (11.7, 16.5)
$100.0,000 - $149,999	1179	77.8 (75.3, 80.2)	221	16.1 (13.9, 18.3)	86	6.2 (4.8, 7.6)	784	52.0 (49.1, 54.8)	551	36.9 (34.1, 39.6)	146	11.1 (9.3, 13.0)
>$150.0,000	1687	84.2 (82.3, 86.0)	220	12.1 (10.5, 13.8)	70	3.7 (2.8, 4.6)	1205	59.3 (56.9, 61.7)	629	32.3 (30.0, 34.6)	144	8.3 (6.9, 9.8)
**Census Region**												
New England	332	81.8 (77.4, 86.2)	48	12.2 (8.3, 16.1)	27	6.0 (3.5, 8.5)	202	47.7 (42.2, 53.2)	164	42.5 (37.1, 48.0)	41	9.8 (6.6, 12.9)
Mid-Atlantic	796	73.4 (70.3, 76.4)	187	18.6 (15.9, 21.3)	80	8.1 (6.1, 10.0)	529	49.0 (45.7, 52.4)	388	36.2 (33.0, 39.5)	148	14.7 (12.3, 17.2)
East-North Central	962	74.6 (71.8, 77.4)	198	17.2 (14.7, 19.6)	91	8.2 (6.4, 10.0)	650	49.7 (46.6, 52.7)	451	37.0 (34.0, 40.0)	148	13.4 (11.1, 15.6)
West-North Central	429	71.0 (67.0, 75.1)	126	21.8 (18.1, 25.5)	42	7.2 (4.9, 9.4)	284	46.0 (41.6, 50.4)	243	41.7 (37.3, 46.1)	71	12.3 (9.5, 15.2)
South Atlantic	1267	73.4 (70.9, 75.9)	281	17.7 (15.6, 19.8)	128	8.9 (7.2, 10.6)	825	47.1 (44.4, 49.8)	635	38.5 (35.9, 41.1)	210	14.4 (12.4, 16.4)
East-South Central	318	66.1 (61.1, 71.1)	95	24.0 (19.4, 28.7)	44	9.9 (6.9, 12.9)	220	46.6 (41.4, 51.8)	157	35.7 (30.6, 40.8)	77	17.7 (13.8, 21.6)
West-South Central	655	71.7 (68.3, 75.1)	153	18.5 (15.5, 21.4)	85	9.9 (7.6, 12.2)	455	48.8 (45.2, 52.5)	299	32.4 (29.0, 35.7)	136	18.8 (15.7, 21.9)
Mountain	542	74.0 (70.4, 77.5)	136	18.0 (14.9, 21.1)	57	8.0 (5.8, 10.2)	348	47.7 (43.6, 51.7)	308	41.5 (37.5, 45.5)	83	10.9 (8.3, 13.4)
Pacific	1103	77.0 (74.3, 79.6)	199	15.8 (13.4, 18.2)	94	7.2 (5.7, 8.8)	755	52.5 (49.4, 55.6)	481	35.2 (32.3, 38.2)	151	12.3 (10.2, 14.3)

Table S5. Variation with respondent characteristics in beliefs concerning race and ethnicity and American society

Characteristic	White people benefit from advantages in society that Black people do not have.						Straight white men hold far too much power in America.						Discrimination against whites is as big a problem as discrimination against Blacks and other minorities.					
	Do not agree		Somewhat agree		Strongly or very strongly agree		Do not agree		Somewhat agree		Strongly or very strongly agree		Do not agree		Somewhat agree		Strongly or very strongly agree	
	Unweighted n	Weighted %, 95% CI	Unweighted n	Weighted %, 95% CI	Unweighted n	Weighted %, 95% CI	Unweighted n	Weighted %, 95% CI	Unweighted n	Weighted %, 95% CI	Unweighted n	Weighted %, 95% CI	Unweighted n	Weighted %, 95% CI	Unweighted n	Weighted %, 95% CI	Unweighted n	Weighted %, 95% CI
**Age, years**																		
18-24	122	26.7 (22.3, 31.1)	113	26.5 (22.0, 31.0)	206	46.8 (41.8, 51.8)	142	30.3 (25.8, 34.8)	110	27.7 (23.1, 32.4)	186	42.0 (37.0, 46.9)	235	52.8 (47.8, 57.8)	99	23.1 (18.9, 27.4)	107	24.0 (19.7, 28.3)
25-34	223	23.0 (20.1, 25.9)	265	27.1 (24.1, 30.1)	520	49.9 (46.5, 53.3)	293	29.8 (26.6, 32.9)	256	25.4 (22.5, 28.4)	459	44.8 (41.4, 48.2)	581	55.7 (52.4, 59.1)	205	20.6 (17.8, 23.4)	222	23.6 (20.7, 26.6)
35-44	398	28.9 (26.3, 31.4)	346	25.7 (23.2, 28.1)	610	45.5 (42.7, 48.3)	488	35.3 (32.6, 38.0)	363	27.3 (24.8, 29.9)	501	37.3 (34.6, 40.1)	723	52.6 (49.8, 55.5)	279	20.5 (18.2, 22.8)	356	26.9 (24.3, 29.4)
45-54	424	35.2 (32.4, 38.0)	348	28.7 (26.0, 31.3)	433	36.1 (33.3, 39.0)	520	43.3 (40.4, 46.3)	345	29.1 (26.4, 31.8)	330	27.6 (24.9, 30.2)	593	49.5 (46.5, 52.5)	272	22.6 (20.1, 25.0)	337	27.9 (25.3, 30.6)
55-64	723	39.6 (37.2, 42.0)	509	28.4 (26.2, 30.7)	576	31.9 (29.6, 34.3)	886	49.6 (47.1, 52.1)	453	25.2 (23.1, 27.4)	461	25.2 (23.0, 27.3)	816	44.4 (41.9, 46.9)	465	25.7 (23.6, 27.9)	536	29.9 (27.6, 32.1)
65-74	632	36.1 (33.7, 38.4)	552	31.0 (28.8, 33.3)	591	32.9 (30.6, 35.2)	850	48.3 (45.8, 50.7)	471	26.8 (24.6, 29.0)	436	24.9 (22.8, 27.1)	814	45.5 (43.1, 47.9)	428	24.3 (22.2, 26.4)	530	30.2 (27.9, 32.4)
75+	344	37.9 (34.6, 41.3)	310	33.1 (29.9, 36.3)	271	29.0 (25.8, 32.1)	500	54.2 (50.8, 57.7)	268	28 (25.0, 31.1)	156	17.7 (15.0, 20.5)	412	43.1 (39.7, 46.5)	238	26.2 (23.1, 29.2)	278	30.8 (27.6, 34.0)
**Gender**																		
Male	1488	35.0 (33.4, 36.6)	1145	27.9 (26.4, 29.5)	1479	37.1 (35.4, 38.8)	2001	46.3 (44.6, 48.0)	1088	27.1 (25.5, 28.6)	1005	26.6 (25.1, 28.2)	1971	48.1 (46.4, 49.9)	970	23.5 (22.1, 25.0)	1176	28.3 (26.8, 29.9)
Female	1341	30.2 (28.6, 31.7)	1265	29.0 (27.5, 30.5)	1645	40.8 (39.2, 42.5)	1627	36.5 (34.9, 38.2)	1153	27.2 (25.6, 28.7)	1448	36.3 (34.6, 38.0)	2118	50.2 (48.5, 51.9)	978	22.6 (21.1, 24.0)	1161	27.2 (25.7, 28.7)
Other	19	16.2 (8.2, 24.3)	20	16.6 (8.9, 24.2)	66	67.2 (57.2, 77.2)	24	18.5 (10.9, 26.0)	18	16.9 (8.3, 25.5)	63	64.6 (54.5, 74.8)	71	70.1 (60.2, 80.0)	20	19.7 (10.5, 28.9)	14	10.2 (4.6, 15.8)
**Race and ethnicity**																		
White, non-Hispanic	2360	39.9 (38.5, 41.2)	1776	29.2 (27.9, 30.4)	1845	31.0 (29.7, 32.2)	2966	48.7 (47.3, 50.1)	1494	24.4 (23.2, 25.5)	1502	26.9 (25.7, 28.2)	2602	42.9 (41.5, 44.2)	1535	25.5 (24.4, 26.7)	1855	31.6 (30.3, 32.9)
Black, non-Hispanic	49	6.9 (4.7, 9.1)	163	20.6 (17.4, 23.9)	604	72.5 (68.9, 76.1)	143	18.5 (15.4, 21.6)	228	28.0 (24.4, 31.6)	433	53.5 (49.6, 57.5)	609	72.8 (69.2, 76.4)	113	14.1 (11.3, 16.9)	92	13.1 (10.2, 16.0)
Hispanic, any race	287	24.7 (21.9, 27.5)	326	30.4 (27.3, 33.5)	458	44.9 (41.6, 48.2)	359	33.4 (30.2, 36.5)	348	32.0 (28.9, 35.2)	354	34.6 (31.4, 37.8)	603	55.9 (52.5, 59.2)	216	21.5 (18.7, 24.4)	252	22.6 (19.9, 25.3)
Asian American / Pacific Islander	65	20.5 (15.4, 25.7)	97	30.4 (24.6, 36.3)	147	49.0 (42.7, 55.4)	82	25.1 (19.6, 30.5)	108	36.6 (30.4, 42.8)	118	38.3 (32.2, 44.5)	181	56.5 (50.2, 62.9)	61	18.8 (13.9, 23.6)	67	24.7 (18.9, 30.4)
Other (American Indian/Alaskan, 2+ races, other)	105	36.1 (28.4, 43.8)	81	24.1 (17.4, 30.8)	153	39.8 (32.6, 47.0)	129	36.4 (29.2, 43.7)	88	30.1 (22.7, 37.6)	122	33.4 (26.4, 40.5)	179	47.4 (39.8, 55.0)	61	21.4 (14.6, 28.1)	100	31.2 (23.9, 38.5)
**Education**																		
No high school diploma or GED	191	33.6 (29.2, 38.0)	168	31.2 (26.9, 35.5)	175	35.2 (30.7, 39.7)	217	39.7 (35.1, 44.3)	161	30.7 (26.4, 35.1)	146	29.6 (25.2, 33.9)	229	43.7 (39.0, 48.3)	135	26.0 (21.8, 30.1)	169	30.3 (26.1, 34.6)
High school graduate	923	39.7 (37.4, 42.0)	622	29.2 (27.0, 31.4)	568	31.1 (28.8, 33.4)	1066	46.4 (44.0, 48.8)	524	26.0 (23.8, 28.1)	507	27.6 (25.4, 29.9)	762	39.5 (37.1, 41.8)	566	25.3 (23.3, 27.4)	791	35.2 (32.9, 37.5)
Some college or Associates degree	861	35.4 (33.3, 37.5)	686	28.1 (26.1, 30.1)	785	36.5 (34.3, 38.7)	1085	43.7 (41.5, 46.0)	617	26.7 (24.7, 28.7)	624	29.6 (27.4, 31.7)	1020	45.6 (43.3, 47.8)	589	24.6 (22.7, 26.5)	726	29.8 (27.8, 31.8)
Bachelors degree	537	25.6 (23.5, 27.6)	532	26.7 (24.5, 28.8)	873	47.7 (45.3, 50.2)	760	36.7 (34.4, 39.0)	499	25.0 (22.9, 27.1)	677	38.3 (35.9, 40.7)	1121	58.9 (56.5, 61.2)	418	20.9 (19.0, 22.9)	403	20.2 (18.3, 22.1)
Masters degree or higher	354	21.0 (18.9, 23.1)	435	27.1 (24.7, 29.5)	806	51.9 (49.3, 54.6)	551	32.2 (29.7, 34.6)	465	29.2 (26.8, 31.6)	575	38.6 (36.0, 41.3)	1042	66.0 (63.5, 68.5)	278	16.9 (14.9, 18.8)	277	17.1 (15.1, 19.2)
**Income**																		
<$25,000	364	35.6 (32.3, 39.0)	297	30.0 (26.7, 33.2)	338	34.4 (31.1, 37.7)	406	38.1 (34.7, 41.4)	277	27.7 (24.5, 30.9)	307	34.2 (30.8, 37.7)	407	41.5 (38.1, 45.0)	249	25.4 (22.3, 28.6)	341	33.0 (29.8, 36.3)
$25,000 - $49,999	539	34.2 (31.4, 36.9)	402	27.0 (24.4, 29.6)	507	38.8 (35.8, 41.8)	633	40.4 (37.5, 43.3)	397	28.0 (25.3, 30.7)	406	31.7 (28.8, 34.5)	609	44.1 (41.1, 47.1)	362	23.9 (21.4, 26.3)	477	32.0 (29.3, 34.8)
$50.0,000 - $74,999	479	31.9 (29.2, 34.7)	428	30.6 (27.8, 33.4)	492	37.5 (34.5, 40.4)	625	42.8 (39.9, 45.8)	362	27.1 (24.3, 29.8)	399	30.1 (27.3, 32.9)	636	46.4 (43.3, 49.4)	355	25.3 (22.7, 27.9)	407	28.3 (25.6, 31.0)
$75,000 - $99,999	392	32.3 (29.4, 35.3)	369	29.2 (26.3, 32.0)	442	38.5 (35.4, 41.6)	518	41.2 (38.1, 44.3)	317	26.0 (23.3, 28.8)	363	32.7 (29.7, 35.8)	601	50.7 (47.5, 53.8)	290	23.2 (20.5, 25.9)	313	26.2 (23.4, 28.9)
$100.0,000 - $149,999	468	30.3 (27.7, 32.9)	411	27.0 (24.5, 29.6)	606	42.6 (39.8, 45.5)	663	42.7 (39.8, 45.5)	361	24.3 (21.8, 26.8)	459	33.1 (30.3, 35.8)	793	53.2 (50.4, 56.1)	307	20.2 (17.9, 22.4)	391	26.6 (24.1, 29.2)
>$150.0,000	624	30.6 (28.4, 32.8)	536	27.1 (24.9, 29.3)	822	42.4 (40.0, 44.8)	834	39.9 (37.6, 42.3)	552	28.2 (26.0, 30.4)	595	31.9 (29.6, 34.2)	1128	56.5 (54.1, 58.9)	423	21.6 (19.5, 23.6)	437	21.9 (19.9, 23.9)
**Census Region**																		
New England	122	27.2 (22.5, 31.8)	123	30.2 (25.0, 35.3)	161	42.7 (37.2, 48.2)	165	35.5 (30.4, 40.6)	102	25.9 (21.0, 30.8)	140	38.6 (33.2, 44.1)	210	53.5 (48.0, 59.0)	86	21.9 (17.2, 26.6)	111	24.6 (20.1, 29.1)
Mid-Atlantic	365	31.3 (28.3, 34.3)	317	28.3 (25.3, 31.2)	386	40.4 (37.1, 43.8)	491	43 (39.7, 46.3)	284	26.0 (23.1, 28.9)	285	31.0 (27.7, 34.2)	506	49.8 (46.4, 53.1)	270	24.4 (21.5, 27.2)	295	25.8 (23.0, 28.7)
East-North Central	422	33.2 (30.4, 36.0)	362	28.4 (25.6, 31.1)	473	38.4 (35.4, 41.4)	553	41.7 (38.7, 44.6)	347	28.4 (25.6, 31.3)	349	29.9 (27.0, 32.8)	592	47.4 (44.3, 50.4)	308	23.9 (21.3, 26.5)	360	28.7 (26.0, 31.5)
West-North Central	206	33.6 (29.4, 37.7)	172	27.8 (23.8, 31.7)	223	38.7 (34.3, 43.0)	277	44.9 (40.4, 49.3)	144	24.4 (20.5, 28.3)	177	30.7 (26.6, 34.9)	276	44.9 (40.5, 49.3)	158	27.0 (23.1, 30.9)	166	28.1 (24.0, 32.2)
South Atlantic	604	34.0 (31.5, 36.5)	465	27.4 (25.0, 29.8)	616	38.6 (36.0, 41.3)	752	42.3 (39.7, 44.9)	444	26.3 (23.9, 28.7)	486	31.4 (28.9, 33.9)	832	49.1 (46.4, 51.8)	350	20.5 (18.4, 22.6)	507	30.4 (27.9, 32.9)
East-South Central	204	45.7 (40.6, 50.9)	113	25.5 (21.0, 30.1)	142	28.7 (24.1, 33.3)	237	51.6 (46.4, 56.8)	111	24.2 (19.8, 28.6)	108	24.1 (19.7, 28.6)	191	39.8 (34.7, 44.8)	109	23.6 (19.2, 27.9)	160	36.7 (31.7, 41.7)
West-South Central	346	36.3 (32.9, 39.8)	258	28.7 (25.3, 32.0)	291	35.0 (31.4, 38.6)	420	43.7 (40.0, 47.3)	216	24.5 (21.3, 27.7)	254	31.8 (28.3, 35.4)	409	48.1 (44.4, 51.8)	211	21.9 (19.0, 24.8)	278	30.0 (26.6, 33.3)
Mountain	260	33.1 (29.3, 36.8)	220	29.3 (25.6, 33.0)	260	37.7 (33.7, 41.7)	320	41.5 (37.5, 45.4)	195	25.2 (21.7, 28.6)	221	33.3 (29.3, 37.3)	359	50.1 (46.1, 54.2)	178	23.4 (19.9, 26.8)	203	26.5 (23.0, 30.0)
Pacific	337	22.6 (20.1, 25.1)	413	29.2 (26.4, 31.9)	655	48.2 (45.1, 51.3)	464	30.7 (27.9, 33.4)	423	32.0 (29.0, 34.9)	509	37.4 (34.4, 40.3)	799	56.5 (53.4, 59.5)	316	23.5 (20.9, 26.2)	286	20.0 (17.6, 22.4)

Table S5, continued

Characteristic	Having more Black Americans, Latinos, and Asian Americans is good for the country.						In America, native-born white people are being replaced by immigrants.					
	Do not agree		Somewhat agree		Strongly or very strongly agree		Do not agree		Somewhat agree		Strongly or very strongly agree	
	Unweighted n	Weighted %, 95% CI	Unweighted n	Weighted %, 95% CI	Unweighted n	Weighted %, 95% CI	Unweighted n	Weighted %, 95% CI	Unweighted n	Weighted %, 95% CI	Unweighted n	Weighted %, 95% CI
**Age, years**												
18-24	62	13.9 (10.5, 17.3)	136	31.6 (26.9, 36.3)	238	54.5 (49.5, 59.5)	278	62.9 (58.0, 67.8)	100	22.9 (18.6, 27.1)	60	14.2 (10.6, 17.8)
25-34	119	12.0 (9.8, 14.2)	288	29.1 (26.1, 32.2)	599	58.9 (55.5, 62.2)	660	64.2 (61.0, 67.5)	224	22.1 (19.3, 25.0)	128	13.6 (11.2, 16.0)
35-44	177	13.7 (11.7, 15.6)	446	32.6 (30.0, 35.3)	728	53.7 (50.9, 56.5)	838	61.6 (58.8, 64.3)	283	21.3 (19.0, 23.6)	232	17.1 (15.0, 19.3)
45-54	239	20.3 (18.0, 22.7)	434	36.2 (33.3, 39.1)	519	43.5 (40.5, 46.4)	702	57.7 (54.8, 60.7)	305	26.2 (23.5, 28.8)	195	16.1 (13.9, 18.2)
55-64	411	23.1 (21.0, 25.1)	680	38.9 (36.5, 41.4)	690	38.0 (35.6, 40.5)	990	54.4 (51.9, 56.8)	524	28.8 (26.6, 31.1)	300	16.8 (14.9, 18.6)
65-74	443	25.9 (23.8, 28.1)	651	37.2 (34.9, 39.6)	653	36.8 (34.4, 39.2)	960	54.1 (51.7, 56.6)	479	27.3 (25.1, 29.5)	329	18.6 (16.7, 20.5)
75+	270	30.1 (26.9, 33.3)	354	39.4 (36.0, 42.8)	284	30.4 (27.2, 33.6)	456	48.6 (45.2, 52.1)	291	31.9 (28.7, 35.1)	175	19.5 (16.8, 22.2)
**Gender**												
Male	891	20.4 (19.1, 21.7)	1440	34.6 (33.0, 36.3)	1746	45.0 (43.2, 46.7)	2344	57.8 (56.1, 59.5)	1071	25.5 (23.9, 27.0)	692	16.7 (15.4, 18.0)
Female	802	18.0 (16.7, 19.3)	1515	35.4 (33.8, 37.0)	1879	46.6 (44.9, 48.3)	2441	58.3 (56.6, 60.0)	1099	25.3 (23.8, 26.8)	708	16.4 (15.2, 17.6)
Other	17	13.4 (6.5, 20.4)	21	19.8 (11.2, 28.3)	67	66.8 (56.8, 76.8)	72	70.4 (60.4, 80.3)	23	21.1 (12.5, 29.7)	10	8.5 (1.8, 15.2)
**Race and ethnicity**												
White, non-Hispanic	1375	22.6 (21.5, 23.7)	2216	37.0 (35.7, 38.3)	2317	40.4 (39.1, 41.8)	3162	53.2 (51.8, 54.5)	1705	28.1 (26.9, 29.4)	1107	18.7 (17.6, 19.8)
Black, non-Hispanic	102	12.4 (9.8, 15.1)	210	27.8 (24.2, 31.4)	497	59.8 (55.9, 63.7)	619	73.4 (69.8, 77.0)	136	17.6 (14.5, 20.6)	61	9 (6.5, 11.5)
Hispanic, any race	152	13.5 (11.2, 15.8)	345	31.5 (28.5, 34.6)	565	55.0 (51.6, 58.3)	707	66.3 (63.2, 69.5)	213	20.2 (17.5, 22.9)	152	13.5 (11.3, 15.7)
Asian American / Pacific Islander	39	10.9 (7.3, 14.6)	98	32.3 (26.3, 38.3)	169	56.8 (50.5, 63.1)	208	63.3 (56.9, 69.6)	63	23.8 (18.1, 29.6)	36	12.9 (8.4, 17.4)
Other (American Indian/Alaskan, 2+ races, other)	53	18.4 (12.5, 24.3)	120	37.8 (30.3, 45.2)	163	43.8 (36.2, 51.5)	188	49.8 (42.2, 57.5)	89	29.2 (22.2, 36.1)	63	21.0 (14.2, 27.8)
**Education**												
No high school diploma or GED	119	20.6 (16.9, 24.3)	223	39.8 (35.3, 44.3)	189	39.6 (34.9, 44.3)	271	52.8 (48.2, 57.5)	141	25.6 (21.6, 29.6)	118	21.6 (17.7, 25.4)
High school graduate	592	25.0 (23.0, 27.0)	797	37.1 (34.8, 39.4)	693	37.9 (35.5, 40.3)	971	49.1 (46.7, 51.5)	647	29.1 (26.9, 31.2)	498	21.9 (19.9, 23.8)
Some college or Associates degree	514	20.2 (18.5, 22)	848	35.8 (33.6, 37.9)	949	44.0 (41.7, 46.3)	1248	55.5 (53.2, 57.7)	648	27.2 (25.2, 29.2)	435	17.4 (15.7, 19)
Bachelors degree	293	14 (12.4, 15.7)	607	30.5 (28.2, 32.7)	1022	55.5 (53.1, 57.9)	1267	66.8 (64.5, 69.0)	455	22.5 (20.5, 24.5)	218	10.7 (9.2, 12.2)
Masters degree or higher	203	11.9 (10.3, 13.6)	514	31.1 (28.7, 33.6)	858	56.9 (54.3, 59.6)	1127	72.0 (69.6, 74.3)	315	18.9 (16.8, 20.9)	150	9.1 (7.6, 10.7)
**Income**												
<$25,000	246	23.6 (20.7, 26.6)	368	37.1 (33.7, 40.4)	375	39.3 (35.9, 42.8)	479	48.5 (45.0, 52.0)	299	30.5 (27.3, 33.8)	219	21.0 (18.2, 23.7)
$25,000 - $49,999	328	20.2 (18.0, 22.5)	548	38.1 (35.2, 41.0)	548	41.7 (38.7, 44.7)	738	54.1 (51.1, 57.0)	386	25.0 (22.5, 27.5)	319	21.0 (18.6, 23.4)
$50.0,000 - $74,999	297	20.0 (17.7, 22.3)	483	34.1 (31.3, 37.0)	600	45.9 (42.8, 48.9)	761	54.9 (51.9, 57.9)	386	26.9 (24.3, 29.5)	251	18.3 (15.9, 20.6)
$75,000 - $99,999	242	19.8 (17.3, 22.2)	405	32.0 (29.1, 34.9)	540	48.2 (45.0, 51.4)	717	60.2 (57.1, 63.3)	304	25.2 (22.5, 27.9)	181	14.6 (12.4, 16.8)
$100.0,000 - $149,999	264	16.7 (14.7, 18.8)	503	34.4 (31.7, 37.2)	708	48.8 (46.0, 51.7)	915	62.2 (59.4, 65.0)	373	24.2 (21.7, 26.6)	201	13.6 (11.7, 15.6)
>$150.0,000	344	16.4 (14.7, 18.2)	682	33.3 (31.0, 35.5)	940	50.3 (47.8, 52.7)	1274	64.9 (62.6, 67.2)	458	22.6 (20.6, 24.6)	248	12.5 (10.9, 14.1)
**Census Region**												
New England	83	18.7 (14.6, 22.7)	144	36.5 (31.2, 41.9)	174	44.8 (39.3, 50.4)	241	61.6 (56.3, 66.8)	92	23.1 (18.5, 27.7)	75	15.3 (11.8, 18.9)
Mid-Atlantic	233	20.0 (17.5, 22.6)	394	35.4 (32.2, 38.6)	429	44.6 (41.2, 47.9)	572	55.0 (51.6, 58.3)	317	29.1 (26.0, 32.1)	177	15.9 (13.5, 18.4)
East-North Central	246	18.2 (16.0, 20.5)	455	36.5 (33.6, 39.5)	544	45.2 (42.2, 48.3)	724	58.2 (55.2, 61.2)	322	25.2 (22.6, 27.8)	211	16.6 (14.3, 18.8)
West-North Central	118	19.1 (15.7, 22.4)	223	37.0 (32.7, 41.3)	257	43.9 (39.5, 48.3)	331	55.1 (50.6, 59.5)	163	28.1 (24.0, 32.1)	106	16.9 (13.8, 20.0)
South Atlantic	359	20.2 (18.1, 22.3)	613	35.4 (32.9, 38.0)	693	44.4 (41.7, 47.1)	993	59.4 (56.8, 62.1)	427	24.4 (22.1, 26.7)	268	16.1 (14.1, 18.2)
East-South Central	120	27.9 (23.1, 32.6)	159	33.8 (28.9, 38.7)	173	38.4 (33.3, 43.4)	236	50.4 (45.2, 55.6)	121	25.7 (21.2, 30.2)	100	23.9 (19.3, 28.4)
West-South Central	195	19.7 (16.9, 22.5)	301	32.8 (29.3, 36.2)	392	47.6 (43.9, 51.3)	494	57.1 (53.5, 60.8)	234	24.7 (21.6, 27.8)	169	18.1 (15.3, 20.9)
Mountain	155	19.4 (16.3, 22.6)	254	34.5 (30.6, 38.3)	321	46.1 (42.0, 50.2)	406	57.5 (53.5, 61.5)	216	27.9 (24.3, 31.5)	115	14.6 (11.9, 17.3)
Pacific	212	14.1 (12.0, 16.1)	446	32.4 (29.5, 35.3)	728	53.5 (50.5, 56.6)	887	63.4 (60.5, 66.4)	314	22.4 (19.8, 25.0)	198	14.2 (12.0, 16.3)

Table S6. Variation with respondent characteristics in beliefs concerning QAnon and biblical “end times”

The government, media, and financial worlds in the U.S. are controlled by a group of Satan-worshipping pedophiles who run a global child sex trafficking operation.

Characteristic	Do not agree		Somewhat agree		Strongly or very strongly agree	
	Unweighted n	Weighted %, 95% CI	Unweighted n	Weighted %, 95% CI	Unweighted n	Weighted %, 95% CI
**Age, years**						
18-24	295	65.5 (60.6, 70.5)	92	22.8 (18.4, 27.1)	46	11.7 (8.2, 15.1)
25-34	744	70.2 (67.0, 73.4)	144	15.7 (13.2, 18.3)	120	14.1 (11.5, 16.6)
35-44	1014	73.1 (70.5, 75.7)	190	15.6 (13.4, 17.7)	141	11.3 (9.5, 13.2)
45-54	958	79.5 (77.0, 82.0)	136	12.3 (10.3, 14.3)	91	8.2 (6.5, 9.9)
55-64	1483	82.5 (80.6, 84.5)	199	11.6 (10.0, 13.3)	104	5.8 (4.7, 7.0)
65-74	1493	83.7 (81.8, 85.6)	154	9.6 (8.0, 11.1)	111	6.8 (5.5, 8.0)
75+	788	82.8 (79.9, 85.7)	85	11.8 (9.2, 14.3)	44	5.4 (3.7, 7.1)
**Gender**						
Male	3344	78.0 (76.5, 79.6)	433	12.8 (11.5, 14.0)	304	9.2 (8.1, 10.3)
Female	3310	75.4 (73.9, 77.0)	547	15.1 (13.8, 16.4)	342	9.4 (8.3, 10.5)
Other	82	77.7 (68.1, 87.2)	14	13.9 (6.1, 21.8)	9	8.4 (1.6, 15.2)
**Race and ethnicity**						
White, non-Hispanic	4923	80.6 (79.4, 81.7)	610	11.6 (10.6, 12.5)	407	7.9 (7.1, 8.6)
Black, non-Hispanic	591	69.9 (66.1, 73.7)	135	18.3 (15.2, 21.5)	72	11.8 (8.9, 14.6)
Hispanic, any race	760	69.1 (65.9, 72.3)	182	20.1 (17.2, 22.9)	109	10.8 (8.7, 12.9)
Asian American / Pacific Islander	246	76.0 (70.1, 82.0)	31	12.1 (7.6, 16.7)	28	11.8 (7.2, 16.5)
Other (American Indian/Alaskan, 2+ races, other)	255	66.3 (58.5, 74.0)	42	17.8 (11.2, 24.5)	41	15.9 (9.7, 22.1)
**Education**						
No high school diploma or GED	307	57.5 (52.7, 62.2)	121	24.9 (20.6, 29.1)	90	17.7 (14.1, 21.3)
High school graduate	1450	66.8 (64.4, 69.1)	364	19.3 (17.3, 21.3)	258	13.9 (12.1, 15.8)
Some college or Associates degree	1837	77.5 (75.5, 79.5)	294	14 (12.3, 15.7)	183	8.5 (7.2, 9.8)
Bachelors degree	1698	86.4 (84.7, 88.2)	150	8.6 (7.2, 10.1)	86	4.9 (3.8, 6.1)
Masters degree or higher	1483	91.9 (90.4, 93.5)	71	5.1 (3.9, 6.4)	40	2.9 (1.9, 3.9)
**Income**						
<$25,000	610	56.6 (53.1, 60.2)	217	25.4 (22.2, 28.7)	153	17.9 (15.1, 20.8)
$25,000 - $49,999	1038	69.0 (66.2, 71.9)	230	18.8 (16.3, 21.2)	156	12.2 (10.1, 14.3)
$50.0,000 - $74,999	1089	74.9 (72.1, 77.7)	165	13.9 (11.7, 16.1)	123	11.2 (9, 13.3)
$75,000 - $99,999	995	80.7 (78.0, 83.4)	120	11.7 (9.5, 13.9)	81	7.6 (5.8, 9.4)
$100.0,000 - $149,999	1259	82.0 (79.7, 84.4)	138	11.4 (9.4, 13.3)	85	6.6 (5.1, 8.1)
>$150.0,000	1784	88.2 (86.4, 90.0)	130	7.7 (6.3, 9.1)	59	4.1 (2.9, 5.3)
**Census Region**						
New England	346	83.2 (78.9, 87.5)	38	11.6 (7.9, 15.3)	21	5.2 (2.7, 7.7)
Mid-Atlantic	868	79.0 (76.0, 81.9)	111	12.2 (9.9, 14.6)	81	8.8 (6.7, 11)
East-North Central	1014	78.8 (76.2, 81.5)	137	12.3 (10.1, 14.4)	99	8.9 (7.0, 10.7)
West-North Central	478	77.2 (73.3, 81.1)	74	14.1 (10.8, 17.3)	43	8.7 (6.1, 11.4)
South Atlantic	1332	75.8 (73.3, 78.3)	196	13.8 (11.8, 15.8)	137	10.4 (8.5, 12.2)
East-South Central	350	73.9 (69.2, 78.7)	64	15.2 (11.4, 18.9)	40	10.9 (7.3, 14.5)
West-South Central	663	71.8 (68.3, 75.3)	144	17.8 (14.9, 20.8)	78	10.4 (8.0, 12.8)
Mountain	601	80.6 (77.2, 83.9)	80	12.6 (9.7, 15.4)	52	6.9 (4.8, 8.9)
Pacific	1123	74.9 (71.9, 77.8)	156	15.3 (12.8, 17.8)	106	9.9 (7.8, 11.9)

There is a storm coming soon that will sweep away the elites in power and restore the rightful leaders.

Characteristic	Do not agree		Somewhat agree		Strongly or very strongly agree	
	Unweighted n	Weighted %, 95% CI	Unweighted n	Weighted %, 95% CI	Unweighted n	Weighted %, 95% CI
**Age, years**						
18-24	292	65.9 (61.0, 70.8)	84	20.3 (16.1, 24.4)	55	13.8 (10.2, 17.5)
25-34	719	68.8 (65.6, 72.0)	168	18.1 (15.4, 20.7)	115	13.1 (10.7, 15.5)
35-44	966	69.6 (66.9, 72.3)	254	20.3 (18.0, 22.7)	129	10.1 (8.3, 11.8)
45-54	880	72.6 (69.9, 75.3)	212	19.1 (16.7, 21.5)	96	8.3 (6.6, 10.0)
55-64	1282	70.7 (68.4, 73.0)	357	21.0 (18.9, 23.0)	148	8.3 (6.9, 9.7)
65-74	1256	70.4 (68.1, 72.7)	338	20.2 (18.2, 22.2)	150	9.3 (7.8, 10.9)
75+	636	66.7 (63.3, 70.1)	197	23.4 (20.3, 26.4)	84	9.9 (7.8, 12.1)
**Gender**						
Male	3003	71.5 (69.9, 73.1)	699	18.0 (16.6, 19.3)	377	10.5 (9.4, 11.7)
Female	2931	68.1 (66.5, 69.7)	879	22.0 (20.6, 23.5)	380	9.9 (8.8, 10.9)
Other	70	66.6 (56.3, 76.8)	21	23.1 (13.7, 32.4)	12	10.4 (4.1, 16.6)
**Race and ethnicity**						
White, non-Hispanic	4349	72.2 (71.0, 73.5)	1091	19.0 (17.9, 20.1)	485	8.8 (8, 9.6)
Black, non-Hispanic	503	59.3 (55.4, 63.3)	178	24.5 (21.0, 28.0)	116	16.2 (13.1, 19.2)
Hispanic, any race	701	65.3 (62.1, 68.5)	237	23.1 (20.3, 25.9)	116	11.6 (9.4, 13.8)
Asian American / Pacific Islander	239	77.6 (72.2, 83.1)	39	11.9 (8.0, 15.8)	26	10.5 (6.1, 14.9)
Other (American Indian/Alaskan, 2+ races, other)	239	64.4 (56.6, 72.2)	65	23.8 (16.6, 31)	34	11.8 (6.6, 17.1)
**Education**						
No high school diploma or GED	278	54.4 (49.7, 59.1)	155	29.1 (24.8, 33.4)	85	16.5 (13, 20.1)
High school graduate	1174	55.8 (53.4, 58.3)	588	28.0 (25.8, 30.1)	317	16.2 (14.3, 18)
Some college or Associates degree	1601	69.4 (67.3, 71.5)	473	20.4 (18.6, 22.2)	229	10.2 (8.7, 11.6)
Bachelors degree	1575	81.7 (79.8, 83.6)	253	13.3 (11.7, 15.0)	96	4.9 (3.9, 6.0)
Masters degree or higher	1403	88.0 (86.2, 89.8)	141	8.9 (7.3, 10.5)	50	3.1 (2.2, 4.1)
**Income**						
<$25,000	539	51.9 (48.4, 55.4)	271	28.8 (25.6, 32.0)	178	19.3 (16.4, 22.2)
$25,000 - $49,999	892	60.6 (57.6, 63.5)	357	26.2 (23.6, 28.9)	177	13.2 (11.1, 15.2)
$50.0,000 - $74,999	929	65.9 (62.9, 68.8)	289	21.2 (18.7, 23.6)	155	12.9 (10.7, 15.2)
$75,000 - $99,999	879	71.9 (69.0, 74.9)	214	19.1 (16.5, 21.7)	94	9.0 (7.0, 10.9)
$100.0,000 - $149,999	1139	75.9 (73.4, 78.4)	246	17.4 (15.2, 19.6)	87	6.7 (5.2, 8.3)
>$150.0,000	1653	82.4 (80.5, 84.3)	233	12.7 (11.0, 14.4)	86	4.9 (3.7, 6.0)
**Census Region**						
New England	297	72.3 (67.2, 77.3)	73	19.6 (15.0, 24.1)	34	8.1 (5.1, 11.1)
Mid-Atlantic	761	70.0 (66.9, 73.2)	207	20.1 (17.4, 22.9)	97	9.9 (7.7, 12.0)
East-North Central	914	71.4 (68.6, 74.2)	233	19.6 (17.1, 22.1)	101	9.0 (7.2, 10.8)
West-North Central	424	69.5 (65.4, 73.7)	120	21.2 (17.5, 24.8)	51	9.3 (6.6, 11.9)
South Atlantic	1177	68.6 (66.0, 71.2)	310	19.4 (17.3, 21.6)	172	12 (10.0, 13.9)
East-South Central	310	67.4 (62.5, 72.3)	92	20.2 (16.1, 24.4)	51	12.4 (8.9, 15.9)
West-South Central	598	66 (62.4, 69.5)	183	20.5 (17.6, 23.5)	101	13.5 (10.7, 16.3)
Mountain	530	72.1 (68.5, 75.7)	144	20.4 (17.1, 23.7)	60	7.5 (5.4, 9.5)
Pacific	1020	70.4 (67.4, 73.3)	248	20.7 (18.0, 23.3)	110	9.0 (7.2, 10.8)

The chaos in America today is evidence that we are living in what the Bible calls “the end times.”

Characteristic	Do not agree		Somewhat agree		Strongly or very strongly agree	
	Unweighted n	Weighted %, 95% CI	Unweighted n	Weighted %, 95% CI	Unweighted n	Weighted %, 95% CI
**Age, years**						
18-24	248	55.3 (50.3, 60.3)	100	23.9 (19.6, 28.2)	87	20.8 (16.7, 24.9)
25-34	643	59.4 (56.1, 62.8)	196	20.5 (17.7, 23.3)	172	20.1 (17.2, 22.9)
35-44	763	54.5 (51.7, 57.4)	322	24.2 (21.8, 26.6)	266	21.3 (18.9, 23.6)
45-54	678	54.8 (51.8, 57.8)	294	25.6 (23.0, 28.3)	220	19.5 (17.1, 22.0)
55-64	1005	54.6 (52.1, 57.1)	466	26.2 (24.0, 28.4)	334	19.2 (17.2, 21.2)
65-74	985	54.0 (51.5, 56.5)	459	26.8 (24.6, 29.0)	317	19.2 (17.2, 21.2)
75+	583	58.9 (55.4, 62.3)	219	25.5 (22.4, 28.6)	119	15.6 (12.9, 18.4)
**Gender**						
Male	2588	60.6 (58.8, 62.3)	892	22.3 (20.9, 23.8)	619	17.1 (15.7, 18.5)
Female	2214	50.7 (49.0, 52.4)	1137	27.0 (25.5, 28.5)	874	22.3 (20.8, 23.7)
Other	80	77.5 (68.2, 86.8)	16	16.2 (7.6, 24.7)	9	6.3 (1.5, 11.2)
**Race and ethnicity**						
White, non-Hispanic	3710	61.0 (59.7, 62.3)	1392	23.6 (22.5, 24.8)	855	15.4 (14.4, 16.4)
Black, non-Hispanic	236	28.5 (24.9, 32.0)	254	32.3 (28.6, 36.1)	319	39.2 (35.4, 43.0)
Hispanic, any race	560	51.8 (48.5, 55.1)	275	26.1 (23.1, 29.0)	230	22.1 (19.4, 24.9)
Asian American / Pacific Islander	220	70.8 (65.0, 76.6)	44	14.0 (9.6, 18.3)	42	15.2 (10.5, 19.9)
Other (American Indian/Alaskan, 2+ races, other)	179	48.9 (41.3, 56.6)	91	25.5 (19.1, 31.8)	69	25.6 (18.3, 32.9)
**Education**						
No high school diploma or GED	196	40.6 (35.9, 45.3)	170	30.5 (26.2, 34.8)	157	28.9 (24.6, 33.1)
High school graduate	860	40.1 (37.8, 42.5)	665	31.6 (29.3, 33.8)	572	28.3 (26.1, 30.5)
Some college or Associates degree	1234	53.4 (51.1, 55.6)	632	26.2 (24.2, 28.2)	456	20.4 (18.6, 22.3)
Bachelors degree	1386	71.6 (69.4, 73.8)	342	17.0 (15.2, 18.8)	211	11.5 (9.8, 13.1)
Masters degree or higher	1229	76.8 (74.5, 79.1)	247	15.5 (13.6, 17.5)	119	7.7 (6.2, 9.2)
**Income**						
<$25,000	368	36.3 (33.0, 39.7)	289	29.3 (26.1, 32.5)	337	34.4 (31.0, 37.7)
$25,000 - $49,999	665	44.0 (41.1, 47.0)	427	29.7 (26.9, 32.4)	350	26.3 (23.6, 29.0)
$50.0,000 - $74,999	721	49.7 (46.6, 52.7)	384	28.0 (25.3, 30.8)	281	22.3 (19.7, 24.9)
$75,000 - $99,999	702	55.8 (52.6, 59.0)	297	25.4 (22.6, 28.2)	194	18.8 (16.2, 21.4)
$100.0,000 - $149,999	955	62.4 (59.6, 65.2)	330	22.6 (20.2, 25.0)	198	15.0 (12.9, 17.1)
>$150.0,000	1494	74.5 (72.3, 76.6)	329	16.8 (15.0, 18.6)	155	8.7 (7.3, 10.1)
**Census Region**						
New England	289	68.6 (63.5, 73.8)	78	19.8 (15.4, 24.2)	40	11.5 (7.7, 15.3)
Mid-Atlantic	628	57.4 (54.1, 60.8)	271	26.2 (23.1, 29.2)	166	16.4 (13.9, 19.0)
East-North Central	746	59.1 (56.1, 62.1)	299	23.7 (21.1, 26.3)	203	17.2 (14.8, 19.5)
West-North Central	352	56.3 (51.9, 60.8)	154	26.3 (22.4, 30.2)	90	17.4 (13.7, 21.0)
South Atlantic	922	51.6 (49.0, 54.3)	413	25.4 (23.0, 27.8)	345	23.0 (20.6, 25.3)
East-South Central	201	41.7 (36.6, 46.8)	115	23.4 (19.2, 27.6)	143	34.9 (29.9, 40.0)
West-South Central	443	47.9 (44.2, 51.6)	247	28.0 (24.6, 31.4)	198	24.1 (20.9, 27.4)
Mountain	414	55.9 (51.9, 60.0)	198	26.6 (23.0, 30.2)	126	17.5 (14.4, 20.6)
Pacific	910	63.5 (60.5, 66.4)	281	20.9 (18.4, 23.4)	204	15.7 (13.4, 17.9)

Table S7. Variation with respondent characteristics in beliefs concerning justification for violence in non-political situations

Characteristic	In self defense						To prevent someone from injuring or killing another person						To prevent someone from injuring or killing themselves					
	Never justified		Sometimes justified		Usually or always justified		Never justified		Sometimes justified		Usually or always justified		Never justified		Sometimes justified		Usually or always justified	
	Unweighted n	Weighted %, 95% CI	Unweighted n	Weighted %, 95% CI	Unweighted n	Weighted %, 95% CI	Unweighted n	Weighted %, 95% CI	Unweighted n	Weighted %, 95% CI	Unweighted n	Weighted %, 95% CI	Unweighted n	Weighted %, 95% CI	Unweighted n	Weighted %, 95% CI	Unweighted n	Weighted %, 95% CI
**Age, years**																		
18-24	9	1.9 (1.0, 3.8)	89	21.4 (17.5, 25.9)	346	76.7 (72.1, 80.7)	12	2.9 (1.6, 5.2)	91	20.7 (17.0, 25.0)	337	76.4 (71.9, 80.4)	27	5.9 (3.9, 8.7)	164	38.2 (33.5, 43.2)	251	55.9 (50.9, 60.8)
25-34	14	1.6 (0.9, 2.8)	234	24.4 (21.5, 27.5)	762	74.0 (70.8, 77.0)	23	2.8 (1.8, 4.2)	209	21.8 (19.1, 24.9)	777	75.4 (72.3, 78.3)	72	7.8 (6.1, 10.0)	410	40.2 (36.9, 43.5)	527	52.0 (48.6, 55.4)
35-44	30	2.6 (1.8, 3.7)	302	23.4 (21.1, 26.0)	1026	74.0 (71.4, 76.5)	34	3.0 (2.1, 4.2)	287	21.8 (19.5, 24.2)	1035	75.2 (72.7, 77.6)	96	7.5 (6.1, 9.2)	515	37.3 (34.6, 40.1)	744	55.1 (52.3, 57.9)
45-54	30	2.8 (1.9, 4.0)	254	21.8 (19.4, 24.4)	924	75.4 (72.8, 77.9)	21	1.9 (1.2, 2.9)	245	21.6 (19.2, 24.2)	940	76.5 (73.8, 79.0)	72	5.9 (4.7, 7.5)	427	35.4 (32.6, 38.3)	706	58.7 (55.7, 61.5)
55-64	24	1.4 (0.9, 2.1)	369	20.7 (18.7, 22.8)	1429	78.0 (75.8, 80.0)	25	1.5 (1.0, 2.3)	353	19.9 (18.0, 22.0)	1435	78.6 (76.4, 80.6)	103	5.7 (4.6, 6.9)	611	33.6 (31.3, 36.0)	1105	60.7 (58.3, 63.1)
65-74	35	2.1 (1.5, 3.0)	357	20.2 (18.3, 22.2)	1391	77.7 (75.6, 79.7)	32	2.0 (1.4, 2.9)	280	16.2 (14.4, 18.2)	1464	81.8 (79.7, 83.6)	81	4.5 (3.6, 5.7)	538	30.3 (28.1, 32.7)	1159	65.1 (62.7, 67.4)
75+	15	1.8 (1.1, 3.1)	193	20.0 (17.4, 22.8)	724	78.2 (75.3, 80.9)	9	1.0 (0.5, 2.0)	125	13.5 (11.3, 16.0)	791	85.5 (82.9, 87.8)	33	3.4 (2.4, 4.8)	211	22.0 (19.3, 25.0)	683	74.6 (71.5, 77.4)
**Gender**																		
Male	62	1.7 (1.3, 2.2)	775	19.7 (18.3, 21.2)	3294	78.6 (77.1, 80.0)	56	1.7 (1.3, 2.4)	669	17.5 (16.2, 18.9)	3389	80.8 (79.3, 82.1)	259	6.6 (5.8, 7.5)	1470	36.6 (34.9, 38.3)	2393	56.8 (55.1, 58.6)
Female	90	2.3 (1.8, 2.9)	986	23.7 (22.3, 25.2)	3192	74.0 (72.5, 75.5)	95	2.7 (2.2, 3.3)	888	21.9 (20.5, 23.4)	3271	75.4 (73.9, 76.9)	213	5.5 (4.7, 6.3)	1336	32.5 (31.0, 34.2)	2707	62.0 (60.3, 63.6)
Other	5	4.2 (1.7, 10.4)	24	24.6 (16.4, 35.2)	77	71.1 (60.4, 79.9)	5	3.2 (1.3, 7.8)	26	25.3 (17.1, 35.7)	75	71.5 (61.1, 80.1)	10	9.6 (5.0, 17.4)	54	51.5 (41.0, 61.9)	42	38.9 (29.2, 49.6)
**Race and ethnicity**																		
White, non-Hispanic	66	1.2 (0.9, 1.6)	1132	18.5 (17.4, 19.5)	4823	80.3 (79.2, 81.4)	65	1.2 (0.9, 1.6)	932	15.7 (14.8, 16.8)	5000	83.0 (82.0, 84.0)	265	4.5 (4.0, 5.1)	1959	33.3 (32.0, 34.6)	3780	62.2 (60.9, 63.5)
Black, non-Hispanic	23	3.0 (1.9, 4.6)	239	31.4 (27.8, 35.3)	551	65.6 (61.7, 69.3)	24	4.0 (2.6, 6.2)	237	30.6 (27.0, 34.4)	548	65.4 (61.5, 69.1)	69	9.1 (7.0, 11.7)	305	39.2 (35.4, 43.2)	435	51.7 (47.7, 55.6)
Hispanic, any race	57	4.6 (3.5, 6.1)	281	26.2 (23.4, 29.3)	737	69.1 (66.0, 72.1)	56	5.0 (3.8, 6.5)	286	26.5 (23.7, 29.6)	731	68.5 (65.4, 71.5)	103	9.6 (7.8, 11.7)	367	34.4 (31.3, 37.7)	605	56.0 (52.7, 59.3)
Asian American / Pacific Islander	6	1.5 (0.6, 3.4)	78	25.2 (20.2, 31.0)	225	73.3 (67.4, 78.4)	5	1.1 (0.4, 2.8)	72	22.3 (17.6, 27.8)	231	76.6 (71.0, 81.3)	16	4.8 (2.7, 8.4)	118	39.9 (33.9, 46.3)	174	55.2 (48.8, 61.4)
Other (American Indian/Alaskan, 2+ races, other)	5	2.5 (0.9, 6.5)	68	26.3 (19.7, 34.3)	266	71.2 (63.2, 78.0)	6	3.0 (1.2, 7.3)	63	22.1 (16.2, 29.5)	269	74.9 (67.3, 81.2)	31	10.2 (6.3, 16.2)	127	38.8 (31.6, 46.6)	181	50.9 (43.3, 58.5)
**Education**																		
No high school diploma or GED	31	5.5 (3.8, 7.8)	145	30.1 (25.8, 34.7)	356	64.5 (59.7, 68.9)	34	6.8 (4.8, 9.6)	129	25.9 (22.0, 30.4)	368	67.3 (62.7, 71.6)	50	10.3 (7.6, 13.7)	170	32.8 (28.5, 37.3)	312	57.0 (52.2, 61.6)
High school graduate	58	2.9 (2.2, 3.8)	427	21.1 (19.1, 23.2)	1643	76.0 (73.8, 78.0)	59	3.1 (2.4, 4.1)	419	21.2 (19.2, 23.3)	1641	75.7 (73.5, 77.8)	144	7.5 (6.3, 8.9)	676	33.4 (31.1, 35.7)	1303	59.1 (56.7, 61.5)
Some college or Associates degree	32	1.4 (1.0, 2.1)	452	19.6 (17.8, 21.4)	1867	79.0 (77.1, 80.8)	34	1.5 (1.0, 2.2)	387	16.7 (15.1, 18.4)	1922	81.8 (80.0, 83.5)	121	5.0 (4.1, 6.0)	760	33.9 (31.7, 36.1)	1464	61.1 (58.9, 63.3)
Bachelors degree	18	0.8 (0.5, 1.3)	382	20.1 (18.2, 22.2)	1544	79.1 (77.0, 81.0)	13	0.7 (0.4, 1.3)	345	19.1 (17.2, 21.2)	1578	80.2 (78.1, 82.1)	86	4.1 (3.3, 5.1)	675	36.2 (33.9, 38.6)	1178	59.7 (57.3, 62.1)
Masters degree or higher	18	1.2 (0.7, 1.9)	392	24.7 (22.4, 27.1)	1192	74.2 (71.8, 76.4)	16	1.1 (0.6, 1.8)	310	20.3 (18.2, 22.5)	1270	78.7 (76.4, 80.8)	83	5.4 (4.3, 6.8)	595	38.0 (35.4, 40.6)	918	56.6 (53.9, 59.2)
**Income**																		
<$25,000	38	4.5 (3.2, 6.3)	257	26.9 (23.8, 30.2)	710	68.6 (65.2, 71.8)	45	5.2 (3.8, 7.1)	220	23.0 (20.1, 26.1)	735	71.8 (68.5, 74.9)	92	9.3 (7.5, 11.6)	322	34.6 (31.3, 38.1)	588	56.1 (52.6, 59.5)
$25,000 - $49,999	44	3.3 (2.4, 4.5)	279	21.5 (19.0, 24.2)	1131	75.2 (72.4, 77.8)	41	3.8 (2.7, 5.3)	265	20.6 (18.2, 23.2)	1138	75.6 (72.8, 78.2)	98	8.1 (6.5, 10.1)	437	31.3 (28.6, 34.2)	915	60.6 (57.6, 63.5)
$50,000 - $74,999	31	2.3 (1.5, 3.3)	294	22.1 (19.6, 24.7)	1077	75.7 (72.9, 78.2)	18	1.6 (0.9, 2.6)	256	19.8 (17.4, 22.4)	1124	78.6 (75.9, 81.1)	74	5.8 (4.5, 7.6)	469	35.0 (32.2, 38.0)	857	59.1 (56.1, 62.1)
$75,000 - $99,999	13	1.4 (0.8, 2.5)	247	19.5 (17.2, 22.0)	944	79.2 (76.5, 81.6)	21	2.1 (1.3, 3.3)	223	18.2 (15.9, 20.7)	960	79.7 (77.1, 82.1)	62	6.0 (4.6, 7.8)	395	31.6 (28.8, 34.6)	746	62.3 (59.2, 65.3)
$100,000 - $149,999	16	0.9 (0.5, 1.6)	308	20.9 (18.7, 23.4)	1173	78.1 (75.6, 80.4)	14	0.9 (0.5, 1.6)	276	19.4 (17.2, 21.9)	1200	79.6 (77.2, 81.9)	58	3.5 (2.6, 4.6)	511	35.5 (32.8, 38.3)	922	61.0 (58.2, 63.8)
>$150,000	15	0.8 (0.5, 1.4)	413	21.4 (19.5, 23.5)	1567	77.8 (75.6, 79.7)	17	1.0 (0.6, 1.7)	350	18.9 (17.0, 20.9)	1622	80.1 (78.1, 82.1)	100	5.0 (4.1, 6.2)	742	38.3 (36.0, 40.7)	1147	56.7 (54.2, 59.1)
**Census Region**																		
New England	7	1.6 (0.7, 3.7)	81	19.1 (15.3, 23.5)	321	79.3 (74.7, 83.3)	11	2.4 (1.2, 4.6)	71	18.6 (14.7, 23.3)	324	79.0 (74.1, 83.1)	28	5.7 (3.8, 8.4)	115	30.1 (25.2, 35.5)	265	64.2 (58.8, 69.3)
Mid-Atlantic	18	1.7 (1.0, 2.9)	233	22.7 (19.9, 25.7)	829	75.6 (72.6, 78.4)	18	2.1 (1.3, 3.6)	208	20.8 (18.1, 23.7)	849	77.1 (74.0, 79.9)	53	5.6 (4.1, 7.7)	342	33.3 (30.2, 36.6)	680	61.0 (57.7, 64.3)
East-North Central	15	1.4 (0.8, 2.5)	268	21.9 (19.5, 24.6)	978	76.6 (73.9, 79.2)	20	2.3 (1.4, 3.8)	250	21.1 (18.7, 23.8)	987	76.6 (73.8, 79.2)	59	4.9 (3.7, 6.6)	425	35.4 (32.5, 38.4)	776	59.7 (56.6, 62.7)
West-North Central	8	1.7 (0.8, 3.6)	129	22.1 (18.5, 26.0)	465	76.2 (72.1, 79.9)	7	1.6 (0.7, 3.5)	96	16.9 (13.7, 20.7)	498	81.4 (77.5, 84.8)	29	5.2 (3.5, 7.6)	205	35.5 (31.3, 39.9)	367	59.3 (54.8, 63.6)
South Atlantic	27	1.7 (1.2, 2.6)	368	22.4 (20.2, 24.8)	1302	75.9 (73.4, 78.1)	32	2.0 (1.4, 2.9)	327	20.6 (18.4, 22.9)	1334	77.4 (75.0, 79.6)	103	6.7 (5.4, 8.2)	557	33.3 (30.9, 35.9)	1029	60.0 (57.3, 62.6)
East-South Central	10	2.8 (1.5, 5.4)	90	19.5 (15.7, 24.0)	360	77.7 (72.9, 81.7)	9	2.7 (1.4, 5.2)	74	16.7 (13.1, 20.9)	375	80.7 (76.2, 84.5)	28	5.8 (3.9, 8.5)	137	31.4 (26.7, 36.5)	293	62.8 (57.6, 67.7)
West-South Central	22	2.3 (1.4, 3.5)	191	23.6 (20.5, 27.0)	688	74.1 (70.7, 77.3)	17	1.7 (1.0, 2.8)	185	22.6 (19.6, 26.0)	699	75.7 (72.3, 78.7)	53	5.8 (4.3, 7.7)	330	39.0 (35.4, 42.7)	516	55.2 (51.5, 58.9)
Mountain	11	1.7 (0.9, 3.2)	120	16.9 (13.9, 20.4)	613	81.4 (77.8, 84.5)	9	1.5 (0.7, 3.1)	108	16.0 (13.1, 19.4)	625	82.5 (78.9, 85.5)	45	6.3 (4.6, 8.5)	255	35.1 (31.3, 39.1)	444	58.6 (54.5, 62.5)
Pacific	39	3.2 (2.3, 4.5)	318	23.3 (20.8, 26.1)	1046	73.5 (70.7, 76.1)	33	3.3 (2.3, 4.7)	271	19.5 (17.2, 22.0)	1088	77.2 (74.5, 79.7)	86	7.4 (5.9, 9.3)	510	35.8 (33.0, 38.8)	805	56.8 (53.7, 59.8)

Table S7, continued

Characteristic	To prevent harm or damage to property						To win an argument						In response to an insult					
	Never justified		Sometimes justified		Usually or always justified		Never justified		Sometimes justified		Usually or always justified		Never justified		Sometimes justified		Usually or always justified	
	Unweighted n	Weighted %, 95% CI	Unweighted n	Weighted %, 95% CI	Unweighted n	Weighted %, 95% CI	Unweighted n	Weighted %, 95% CI	Unweighted n	Weighted %, 95% CI	Unweighted n	Weighted %, 95% CI	Unweighted n	Weighted %, 95% CI	Unweighted n	Weighted %, 95% CI	Unweighted n	Weighted %, 95% CI
**Age, years**																		
18-24	69	15.3 (12.1, 19.2)	236	52.6 (47.6, 57.6)	135	32.1 (27.5, 37.0)	381	83.9 (79.6, 87.4)	43	10.9 (8.1, 14.7)	17	5.2 (3.2, 8.2)	337	74.2 (69.5, 78.4)	85	20.2 (16.4, 24.5)	21	5.6 (3.6, 8.7)
25-34	175	16.9 (14.6, 19.6)	549	53.1 (49.7, 56.5)	286	30.0 (26.9, 33.2)	887	86.6 (84.0, 88.8)	83	8.9 (7.1, 11.1)	39	4.5 (3.2, 6.2)	816	79.6 (76.7, 82.2)	140	14.4 (12.2, 16.9)	53	6.0 (4.5, 8.0)
35-44	244	17.7 (15.7, 20.0)	718	52.1 (49.3, 54.9)	396	30.2 (27.6, 32.9)	1181	85.8 (83.6, 87.7)	117	9.3 (7.8, 11.1)	59	4.9 (3.8, 6.4)	1110	80.6 (78.3, 82.8)	192	15.1 (13.1, 17.3)	55	4.3 (3.3, 5.6)
45-54	180	15.2 (13.2, 17.5)	620	50.6 (47.6, 53.5)	406	34.2 (31.5, 37.1)	1069	87.8 (85.7, 89.7)	97	8.7 (7.1, 10.6)	37	3.5 (2.5, 4.8)	1024	83.5 (81.1, 85.7)	137	12.7 (10.7, 14.9)	44	3.8 (2.8, 5.1)
55-64	279	14.8 (13.1, 16.6)	907	50.3 (47.8, 52.8)	632	34.9 (32.6, 37.3)	1640	88.9 (87.0, 90.5)	137	8.7 (7.2, 10.4)	42	2.5 (1.8, 3.4)	1605	86.6 (84.7, 88.3)	151	9.3 (7.9, 11.0)	64	4.0 (3.1, 5.2)
65-74	254	14.0 (12.4, 15.8)	818	46.0 (43.5, 48.4)	703	40.0 (37.6, 42.4)	1561	86.9 (85.1, 88.5)	170	10.1 (8.7, 11.7)	49	3.0 (2.2, 4.0)	1549	86.3 (84.4, 87.9)	180	10.9 (9.4, 12.6)	47	2.8 (2.1, 3.8)
75+	106	11.3 (9.3, 13.6)	429	45.0 (41.6, 48.4)	392	43.8 (40.4, 47.2)	811	85.4 (82.6, 87.8)	89	11.0 (8.8, 13.5)	30	3.7 (2.5, 5.3)	813	85.5 (82.7, 87.9)	86	10.9 (8.8, 13.5)	30	3.6 (2.5, 5.2)
**Gender**																		
Male	536	13.3 (12.1, 14.5)	2119	51.1 (49.3, 52.8)	1471	35.7 (34.0, 37.3)	3667	87.3 (86.0, 88.4)	338	9.1 (8.1, 10.2)	119	3.7 (3.0, 4.5)	3517	83.1 (81.6, 84.4)	475	13.0 (11.9, 14.3)	131	3.9 (3.2, 4.7)
Female	727	16.7 (15.5, 18.0)	2083	49.8 (48.1, 51.5)	1442	33.5 (31.9, 35.1)	3725	86.1 (84.9, 87.3)	384	9.8 (8.8, 10.9)	149	4.1 (3.4, 4.8)	3608	82.1 (80.7, 83.4)	473	13.2 (12.0, 14.5)	178	4.7 (4.0, 5.5)
Other	35	36.2 (26.7, 47.0)	50	45.9 (35.5, 56.6)	20	17.9 (11.1, 27.6)	91	84.7 (74.1, 91.5)	11	11.3 (5.6, 21.8)	4	3.9 (1.3, 11.2)	84	75.2 (64.0, 83.8)	17	16.9 (10.3, 26.5)	5	7.9 (3.0, 19.3)
**Race and ethnicity**																		
White, non-Hispanic	840	14.0 (13.1, 15.0)	3064	51.4 (50.1, 52.8)	2101	34.5 (33.3, 35.8)	5432	90.0 (89.1, 90.8)	449	7.6 (6.9, 8.3)	130	2.4 (2.0, 2.9)	5295	86.8 (85.8, 87.7)	557	10.3 (9.4, 11.2)	156	2.9 (2.5, 3.5)
Black, non-Hispanic	166	20.6 (17.7, 24.0)	374	47.2 (43.2, 51.1)	270	32.2 (28.7, 35.9)	648	79.0 (75.6, 82.0)	107	14.1 (11.6, 17.2)	56	6.9 (5.1, 9.1)	595	72.0 (68.3, 75.4)	150	19.7 (16.7, 23.1)	66	8.3 (6.3, 10.8)
Hispanic, any race	197	18.1 (15.7, 20.8)	501	46.9 (43.6, 50.2)	376	35.0 (31.9, 38.2)	890	81.9 (79.2, 84.3)	120	11.8 (9.8, 14.1)	62	6.3 (4.9, 8.2)	837	76.3 (73.3, 79.0)	178	17.7 (15.2, 20.4)	60	6.1 (4.6, 8.0)
Asian American / Pacific Islander	33	9.8 (6.9, 13.9)	166	56.0 (49.7, 62.2)	108	34.1 (28.4, 40.4)	255	82.2 (76.6, 86.6)	35	11.4 (8.0, 16.1)	16	6.4 (3.7, 10.8)	233	75.0 (69.0, 80.2)	54	18.5 (14.1, 24.0)	19	6.5 (3.9, 10.7)
Other (American Indian/Alaskan, 2+ races, other)	71	18.2 (13.4, 24.3)	172	49.0 (41.4, 56.6)	95	32.8 (25.7, 40.8)	305	82.3 (74.4, 88.2)	25	14.4 (9.0, 22.2)	9	3.3 (1.3, 7.9)	294	80.2 (72.7, 86.1)	32	15.4 (10.3, 22.5)	13	4.3 (1.8, 9.8)
**Education**																		
No high school diploma or GED	83	15.1 (12.1, 18.7)	245	49.0 (44.4, 53.7)	204	35.8 (31.5, 40.4)	392	72.1 (67.6, 76.1)	88	17.6 (14.2, 21.6)	51	10.3 (7.7, 13.7)	367	67.6 (63.1, 71.9)	124	23.9 (20.1, 28.1)	42	8.5 (6.1, 11.7)
High school graduate	327	15.9 (14.2, 17.7)	906	42.9 (40.5, 45.3)	891	41.2 (38.8, 43.6)	1728	80.8 (78.9, 82.6)	285	13.5 (11.9, 15.2)	111	5.7 (4.7, 7.0)	1674	77.1 (75.0, 79.1)	320	16.3 (14.6, 18.2)	130	6.5 (5.4, 7.9)
Some college or Associates degree	343	14.5 (13.0, 16.2)	1119	49.3 (47.0, 51.6)	877	36.2 (34.0, 38.4)	2102	89.5 (88.0, 90.8)	178	7.7 (6.6, 9.1)	66	2.8 (2.1, 3.7)	2030	85.2 (83.4, 86.9)	235	11.4 (9.9, 13.0)	80	3.4 (2.7, 4.3)
Bachelors degree	294	15.4 (13.7, 17.3)	1092	56.7 (54.3, 59.1)	556	27.9 (25.8, 30.1)	1801	92.4 (90.9, 93.6)	116	6.2 (5.1, 7.6)	24	1.4 (0.9, 2.1)	1737	88.0 (86.2, 89.5)	165	9.5 (8.1, 11.1)	38	2.5 (1.8, 3.5)
Masters degree or higher	260	16.2 (14.3, 18.2)	915	58.2 (55.6, 60.8)	422	25.6 (23.4, 28.0)	1507	93.9 (92.4, 95.1)	69	4.5 (3.5, 5.8)	21	1.6 (1.0, 2.6)	1446	89.2 (87.3, 90.8)	127	9.1 (7.6, 10.8)	24	1.8 (1.1, 2.7)
**Income**																		
<$25,000	181	17.9 (15.4, 20.7)	436	44.3 (40.8, 47.8)	385	37.9 (34.5, 41.3)	764	74.5 (71.3, 77.4)	146	15.4 (13.0, 18.2)	94	10.1 (8.2, 12.5)	726	69.3 (65.9, 72.5)	178	20.3 (17.6, 23.4)	98	10.4 (8.4, 12.8)
$25,000 - $49,999	224	16.6 (14.5, 19.0)	645	44.7 (41.8, 47.7)	581	38.7 (35.9, 41.6)	1224	82.5 (80.1, 84.7)	164	12.7 (10.7, 14.9)	62	4.8 (3.6, 6.3)	1187	79.0 (76.4, 81.4)	195	15.5 (13.4, 17.9)	69	5.5 (4.2, 7.1)
$50,000 - $74,999	223	15.3 (13.3, 17.6)	662	48.0 (45.0, 51.0)	512	36.6 (33.8, 39.6)	1254	88.7 (86.5, 90.5)	108	8.2 (6.7, 10.1)	34	3.1 (2.1, 4.5)	1211	84.5 (82.0, 86.6)	151	12.5 (10.6, 14.8)	36	3.0 (2.0, 4.4)
$75,000 - $99,999	191	16.3 (14.1, 18.8)	593	48.7 (45.6, 51.9)	415	35.0 (32.0, 38.1)	1068	86.9 (84.5, 89.0)	107	9.9 (8.1, 12.1)	29	3.2 (2.1, 4.8)	1015	82.5 (79.9, 84.9)	149	13.3 (11.2, 15.7)	40	4.2 (3.0, 5.8)
$100,000 - $149,999	201	13.4 (11.6, 15.4)	822	54.6 (51.8, 57.5)	471	32.0 (29.4, 34.7)	1357	89.9 (88.0, 91.6)	108	7.6 (6.2, 9.3)	29	2.4 (1.6, 3.6)	1320	86.0 (83.7, 88.0)	135	10.9 (9.1, 13.0)	38	3.1 (2.2, 4.3)
>$150,000	287	14.2 (12.6, 16.0)	1119	57.1 (54.7, 59.4)	586	28.7 (26.6, 30.9)	1863	92.5 (90.9, 93.8)	103	5.8 (4.7, 7.2)	25	1.7 (1.1, 2.7)	1795	88.1 (86.2, 89.7)	163	9.8 (8.3, 11.5)	33	2.1 (1.4, 3.2)
**Census Region**																		
New England	62	13.7 (10.5, 17.7)	212	53.4 (47.9, 58.8)	135	32.9 (28.0, 38.2)	370	90.0 (86.1, 92.9)	25	5.8 (3.8, 8.7)	14	4.2 (2.3, 7.5)	353	82.4 (77.4, 86.5)	41	12.8 (9.3, 17.3)	14	4.8 (2.7, 8.5)
Mid-Atlantic	168	15.7 (13.5, 18.3)	485	46.6 (43.2, 49.9)	422	37.7 (34.5, 40.9)	928	85.3 (82.7, 87.6)	105	10.6 (8.7, 12.9)	42	4.1 (3.0, 5.6)	907	81.7 (78.9, 84.3)	127	13.8 (11.5, 16.4)	43	4.5 (3.3, 6.1)
East-North Central	200	16.5 (14.3, 18.9)	653	52.3 (49.2, 55.3)	403	31.2 (28.5, 34.1)	1126	89.0 (86.9, 90.7)	103	8.4 (6.8, 10.2)	30	2.7 (1.8, 3.9)	1086	85.3 (82.9, 87.3)	125	10.7 (8.9, 12.8)	47	4.0 (3.0, 5.4)
West-North Central	79	13.6 (10.8, 17.0)	309	50.3 (45.9, 54.8)	212	36.1 (31.9, 40.5)	536	87.7 (84.3, 90.5)	52	10.0 (7.5, 13.3)	13	2.3 (1.3, 3.9)	524	85.3 (81.6, 88.3)	60	11.6 (8.9, 15.1)	17	3.1 (1.9, 5.0)
South Atlantic	252	15.0 (13.2, 16.9)	875	52.2 (49.5, 54.8)	565	32.9 (30.4, 35.4)	1492	86.7 (84.6, 88.5)	154	10.3 (8.6, 12.2)	46	3.1 (2.2, 4.2)	1413	80.9 (78.5, 83.0)	211	14.7 (12.8, 16.9)	66	4.4 (3.3, 5.8)
East-South Central	68	14.7 (11.4, 18.7)	234	49.5 (44.4, 54.7)	157	35.8 (30.9, 41.0)	403	85.0 (80.6, 88.5)	39	10.1 (7.3, 13.8)	18	4.9 (2.9, 8.2)	394	83.8 (79.3, 87.4)	49	11.7 (8.7, 15.6)	16	4.6 (2.6, 7.8)
West-South Central	117	13.5 (11.2, 16.3)	432	48.1 (44.4, 51.7)	352	38.4 (34.9, 42.0)	779	85.4 (82.4, 87.9)	82	9.5 (7.5, 11.9)	38	5.1 (3.6, 7.3)	740	80.0 (76.7, 82.9)	114	14.1 (11.7, 17.0)	47	5.9 (4.2, 8.1)
Mountain	134	17.8 (14.9, 21.1)	354	48.2 (44.2, 52.3)	254	34.0 (30.3, 37.9)	679	90.3 (87.4, 92.6)	52	7.5 (5.5, 10.0)	12	2.3 (1.2, 4.3)	654	85.6 (82.2, 88.5)	72	12.2 (9.5, 15.5)	16	2.2 (1.3, 3.7)
Pacific	227	16.5 (14.3, 18.9)	723	51.3 (48.2, 54.3)	450	32.3 (29.5, 35.2)	1217	84.2 (81.6, 86.4)	124	10.1 (8.3, 12.3)	60	5.7 (4.3, 7.5)	1183	81.2 (78.6, 83.6)	172	14.3 (12.2, 16.6)	48	4.5 (3.3, 6.2)

Table S7, continued

Characteristic	To get respect					
	Never justified		Sometimes justified		Usually or always justified	
	Unweighted n	Weighted %, 95% CI	Unweighted n	Weighted %, 95% CI	Unweighted n	Weighted %, 95% CI
**Age, years**						
18-24	373	81.2 (76.7, 85.0)	49	12.4 (9.4, 16.3)	21	6.3 (4.1, 9.7)
25-34	885	85.8 (83.2, 88.1)	75	7.9 (6.3, 10.0)	50	6.3 (4.7, 8.4)
35-44	1185	86.0 (83.9, 87.9)	109	9.1 (7.5, 10.9)	63	4.9 (3.8, 6.3)
45-54	1067	87.4 (85.2, 89.3)	90	8.5 (6.9, 10.4)	45	4.1 (3.0, 5.5)
55-64	1665	90.6 (88.9, 92.1)	99	5.9 (4.8, 7.3)	56	3.5 (2.6, 4.7)
65-74	1599	89.2 (87.5, 90.6)	128	7.6 (6.3, 9.0)	52	3.3 (2.5, 4.3)
75+	842	88.5 (85.9, 90.6)	53	6.3 (4.8, 8.3)	35	5.2 (3.7, 7.3)
**Gender**						
Male	3697	87.3 (86.0, 88.5)	286	8.1 (7.1, 9.2)	141	4.5 (3.8, 5.5)
Female	3780	87.0 (85.7, 88.1)	307	8.2 (7.3, 9.2)	173	4.8 (4.1, 5.6)
Other	95	87.4 (76.6, 93.6)	6	7.1 (3.0, 15.9)	5	5.5 (1.7, 16.3)
**Race and ethnicity**						
White, non-Hispanic	5551	91.7 (90.8, 92.4)	312	5.5 (4.9, 6.2)	148	2.8 (2.4, 3.3)
Black, non-Hispanic	626	75.2 (71.6, 78.6)	116	15.2 (12.6, 18.4)	70	9.5 (7.3, 12.3)
Hispanic, any race	880	80.5 (77.7, 83.0)	121	12.0 (10.0, 14.3)	73	7.6 (5.9, 9.5)
Asian American / Pacific Islander	254	84.0 (79.0, 88.1)	30	9.0 (6.1, 13.1)	22	6.9 (4.3, 11.1)
Other (American Indian/Alaskan, 2+ races, other)	305	81.5 (73.3, 87.6)	24	12.9 (8.0, 20.2)	9	5.6 (2.3, 12.8)
**Education**						
No high school diploma or GED	391	71.7 (67.2, 75.7)	77	15.3 (12.2, 19.0)	64	13.0 (10.1, 16.7)
High school graduate	1750	81.3 (79.4, 83.2)	235	11.4 (10.0, 13.1)	139	7.2 (6.0, 8.7)
Some college or Associates degree	2120	89.5 (88.0, 90.9)	155	7.4 (6.2, 8.8)	70	3.0 (2.3, 4.0)
Bachelors degree	1820	92.6 (91.1, 93.8)	92	5.3 (4.3, 6.6)	31	2.1 (1.4, 3.0)
Masters degree or higher	1535	95.7 (94.5, 96.7)	44	2.9 (2.1, 4.0)	18	1.3 (0.8, 2.2)
**Income**						
<$25,000	761	72.5 (69.1, 75.6)	131	15.7 (13.2, 18.7)	111	11.8 (9.7, 14.3)
$25,000 - $49,999	1235	83.2 (80.7, 85.4)	137	10.3 (8.5, 12.3)	78	6.5 (5.1, 8.3)
$50,000 - $74,999	1258	88.4 (86.2, 90.3)	96	7.8 (6.3, 9.7)	45	3.8 (2.7, 5.3)
$75,000 - $99,999	1078	87.9 (85.5, 89.9)	93	8.1 (6.5, 10.0)	33	4.0 (2.7, 5.8)
$100,000 - $149,999	1399	91.7 (89.8, 93.3)	65	5.4 (4.2, 7.0)	30	2.9 (1.9, 4.2)
>$150,000	1885	93.2 (91.7, 94.5)	81	4.9 (3.9, 6.1)	25	1.9 (1.2, 3.0)
**Census Region**						
New England	369	88.4 (84.1, 91.6)	24	6.8 (4.4, 10.2)	16	4.9 (2.8, 8.3)
Mid-Atlantic	943	86.1 (83.5, 88.3)	84	9.1 (7.3, 11.4)	50	4.8 (3.5, 6.3)
East-North Central	1140	89.8 (87.8, 91.6)	75	6.3 (5.0, 8.0)	43	3.9 (2.8, 5.3)
West-North Central	546	89.4 (86.0, 92.0)	39	7.4 (5.3, 10.3)	16	3.2 (1.8, 5.6)
South Atlantic	1486	84.6 (82.4, 86.7)	144	10.3 (8.6, 12.2)	64	5.1 (3.8, 6.7)
East-South Central	406	86.5 (82.3, 89.8)	36	8.5 (6.0, 11.9)	17	5.0 (3.0, 8.4)
West-South Central	783	84.9 (81.8, 87.6)	78	9.6 (7.6, 12.2)	39	5.4 (3.8, 7.7)
Mountain	692	92.1 (89.3, 94.2)	32	4.6 (3.1, 6.8)	18	3.3 (1.9, 5.5)
Pacific	1251	86.9 (84.5, 88.9)	91	7.6 (6.1, 9.5)	59	5.5 (4.2, 7.2)

Table S8. Variation with respondent characteristics in beliefs concerning justification for political violence, in general and for 9 specific objectives

Characteristic	In general…to advance an important political objective that you support						Thinks violence is usually or always justified to advance at least 1 of 17 objectives		To return Donald Trump to the presidency this year					
	Never justified		Sometimes justified		Usually or always justified				Never justified		Sometimes justified		Usually or always justified	
	Unweighted n	Weighted %, 95% CI	Unweighted n	Weighted %, 95% CI	Unweighted n	Weighted %, 95% CI	Unweighted n	Weighted %, 95% CI	Unweighted n	Weighted %, 95% CI	Unweighted n	Weighted %, 95% CI	Unweighted n	Weighted %, 95% CI
**Age, years**														
18-24	306	66.6 (61.7, 71.2)	116	26.8 (22.6, 31.5)	23	6.5 (4.3, 9.8)	174	40.0 (35.2, 45.0)	376	82.9 (78.5, 86.5)	34	8.3 (5.8, 11.7)	32	8.8 (6.2, 12.5)
25-34	705	68.7 (65.5, 71.8)	279	26.9 (24.0, 29.9)	36	4.4 (3.1, 6.2)	298	30.6 (27.6, 33.8)	883	86.1 (83.6, 88.4)	78	8.7 (6.9, 11.0)	46	5.1 (3.8, 6.8)
35-44	1019	74.9 (72.4, 77.3)	297	21.0 (18.8, 23.4)	49	4.0 (3.0, 5.4)	352	26.6 (24.2, 29.2)	1198	88.0 (86.0, 89.7)	91	7.1 (5.7, 8.7)	61	4.9 (3.8, 6.3)
45-54	1004	82.8 (80.5, 84.9)	180	14.6 (12.7, 16.7)	30	2.6 (1.8, 3.7)	351	30.0 (27.4, 32.8)	1083	89.1 (87.1, 90.9)	55	5.0 (3.8, 6.5)	66	5.9 (4.6, 7.5)
55-64	1578	85.5 (83.5, 87.2)	220	12.9 (11.2, 14.7)	28	1.7 (1.1, 2.5)	571	31.7 (29.4, 34.0)	1646	90.9 (89.3, 92.2)	93	5.3 (4.2, 6.6)	67	3.8 (3.0, 5.0)
65-74	1618	90.2 (88.6, 91.6)	154	9.0 (7.7, 10.6)	13	0.8 (0.4, 1.3)	641	36.6 (34.3, 39.0)	1598	89.8 (88.2, 91.3)	71	4.2 (3.3, 5.3)	95	6.0 (4.8, 7.4)
75+	843	90.3 (88.1, 92.1)	84	8.5 (6.8, 10.5)	10	1.2 (0.6, 2.4)	383	42.5 (39.1, 45.9)	831	89.7 (87.4, 91.6)	39	3.9 (2.8, 5.4)	54	6.4 (4.8, 8.4)
**Gender**														
Male	3313	76.8 (75.2, 78.3)	735	19.9 (18.5, 21.4)	97	3.3 (2.7, 4.1)	1375	33.3 (31.7, 34.9)	3722	88.9 (87.6, 90.0)	206	5.9 (5.1, 6.9)	177	5.2 (4.4, 6.1)
Female	3657	82.8 (81.4, 84.1)	546	14.5 (13.3, 15.8)	87	2.7 (2.1, 3.4)	1326	31.8 (30.2, 33.4)	3751	87.4 (86.2, 88.5)	252	6.7 (5.8, 7.6)	234	5.9 (5.1, 6.8)
Other	62	53.2 (42.5, 63.7)	42	43.2 (33.1, 54.0)	2	3.5 (0.6, 17.2)	46	45.3 (35.0, 56.0)	100	96.3 (91.7, 98.4)	3	1.5 (0.5, 4.5)	3	2.2 (0.7, 6.9)
**Race and ethnicity**														
White, non-Hispanic	5065	82.0 (80.9, 83.1)	881	16.3 (15.3, 17.4)	84	1.7 (1.3, 2.1)	1916	32.0 (30.7, 33.3)	5390	89.5 (88.6, 90.3)	296	5.3 (4.7, 6.0)	291	5.2 (4.6, 5.8)
Black, non-Hispanic	657	75.4 (71.7, 78.8)	130	17.9 (15.0, 21.2)	41	6.7 (4.8, 9.4)	296	36.1 (32.5, 39.9)	703	84.1 (80.7, 87.0)	63	8.8 (6.7, 11.4)	42	7.1 (5.1, 9.9)
Hispanic, any race	851	75.9 (72.9, 78.7)	186	19.4 (16.8, 22.3)	45	4.7 (3.5, 6.4)	358	33.3 (30.3, 36.5)	951	88.3 (86.0, 90.3)	62	6.3 (4.8, 8.2)	52	5.3 (4.0, 7.1)
Asian American / Pacific Islander	242	75.9 (69.9, 81.1)	57	19.8 (15.1, 25.5)	11	4.2 (2.2, 8.1)	88	29.6 (24.1, 35.8)	266	85.6 (80.3, 89.7)	16	5.6 (3.3, 9.3)	24	8.8 (5.6, 13.6)
Other (American Indian/Alaskan, 2+ races, other)	258	68.6 (60.7, 75.5)	76	26.6 (20.2, 34.2)	8	4.8 (2.0, 11.0)	112	38.2 (31.0, 45.9)	305	82.2 (74.1, 88.1)	24	13.9 (8.5, 22.0)	12	3.9 (1.9, 7.8)
**Education**														
No high school diploma or GED	406	72.4 (67.9, 76.4)	96	19.9 (16.3, 24.1)	38	7.7 (5.5, 10.7)	229	41.9 (37.4, 46.5)	405	76.2 (71.9, 80.1)	62	12.3 (9.5, 16.0)	58	11.4 (8.7, 14.9)
High school graduate	1754	79.0 (76.9, 80.9)	323	16.7 (15.0, 18.7)	68	4.3 (3.3, 5.6)	920	42.0 (39.6, 44.3)	1728	81.3 (79.4, 83.2)	195	9.6 (8.2, 11.1)	184	9.1 (7.8, 10.6)
Some college or Associates degree	1970	80.8 (78.8, 82.6)	343	17.1 (15.3, 19.0)	42	2.2 (1.5, 3.0)	815	33.5 (31.4, 35.6)	2108	90.1 (88.6, 91.4)	117	5.5 (4.5, 6.8)	110	4.3 (3.5, 5.3)
Bachelors degree	1615	80.4 (78.3, 82.3)	308	18.1 (16.2, 20.2)	26	1.5 (1.0, 2.3)	482	24.4 (22.4, 26.5)	1836	94.9 (93.7, 95.9)	52	2.7 (2.0, 3.6)	44	2.4 (1.7, 3.3)
Masters degree or higher	1328	81.2 (78.9, 83.2)	260	17.5 (15.5, 19.6)	15	1.4 (0.8, 2.4)	324	19.7 (17.7, 21.9)	1538	96.0 (94.7, 97.0)	35	2.0 (1.4, 2.8)	25	2.0 (1.3, 3.1)
**Income**														
<$25,000	770	72.1 (68.7, 75.2)	183	20.9 (18.1, 24.0)	57	7.1 (5.4, 9.3)	465	46.9 (43.5, 50.4)	772	76.0 (72.7, 79.0)	104	11.9 (9.7, 14.6)	113	12.1 (9.9, 14.7)
$25,000 - $49,999	1208	78.7 (75.9, 81.2)	215	17.2 (14.9, 19.7)	42	4.1 (2.9, 5.7)	584	39.8 (36.9, 42.7)	1238	84.6 (82.3, 86.7)	105	8.4 (6.8, 10.4)	99	6.9 (5.6, 8.6)
$50,000 - $74,999	1189	82.1 (79.5, 84.4)	195	15.4 (13.3, 17.8)	27	2.5 (1.6, 4.0)	447	32.7 (30.0, 35.6)	1253	88.7 (86.5, 90.6)	82	6.0 (4.7, 7.6)	58	5.3 (3.9, 7.1)
$75,000 - $99,999	995	81.0 (78.3, 83.4)	192	17.1 (14.8, 19.7)	20	1.9 (1.2, 3.0)	390	32.3 (29.4, 35.3)	1090	89.6 (87.3, 91.5)	63	6.1 (4.6, 8.1)	49	4.3 (3.2, 5.8)
$100,000 - $149,999	1233	78.9 (76.3, 81.3)	245	19.3 (17.0, 21.8)	19	1.8 (1.1, 2.8)	417	28.0 (25.5, 30.6)	1372	91.1 (89.2, 92.6)	64	4.9 (3.8, 6.4)	52	4.0 (3.0, 5.4)
>$150,000	1678	81.8 (79.7, 83.7)	300	16.2 (14.5, 18.1)	24	2.0 (1.2, 3.2)	467	23.6 (21.6, 25.8)	1890	94.3 (92.9, 95.4)	43	2.5 (1.8, 3.5)	50	3.2 (2.3, 4.4)
**Census Region**														
New England	349	83.0 (78.3, 86.9)	52	14.5 (10.9, 19.1)	8	2.4 (1.2, 4.9)	127	30.0 (25.3, 35.2)	362	87.9 (83.6, 91.2)	18	5.2 (3.0, 8.8)	27	6.9 (4.6, 10.3)
Mid-Atlantic	891	79.8 (76.8, 82.4)	170	16.9 (14.6, 19.6)	25	3.3 (2.1, 5.1)	355	32.3 (29.3, 35.5)	965	88.7 (86.2, 90.8)	51	5.4 (4.0, 7.2)	58	5.9 (4.4, 7.9)
East-North Central	1043	80.1 (77.4, 82.5)	198	17.5 (15.2, 20.0)	25	2.4 (1.6, 3.7)	406	31.5 (28.8, 34.3)	1122	88.8 (86.7, 90.7)	64	5.7 (4.4, 7.4)	63	5.4 (4.2, 7.0)
West-North Central	493	78.5 (74.2, 82.3)	96	19.2 (15.6, 23.5)	13	2.3 (1.3, 4.0)	215	37.8 (33.5, 42.3)	538	89.1 (85.8, 91.6)	37	6.5 (4.6, 9.2)	24	4.4 (2.8, 6.8)
South Atlantic	1404	78.8 (76.4, 81.1)	273	18.4 (16.3, 20.7)	29	2.8 (1.8, 4.2)	567	33.7 (31.2, 36.3)	1502	87.5 (85.4, 89.3)	97	6.7 (5.4, 8.4)	85	5.8 (4.5, 7.3)
East-South Central	386	81.2 (76.4, 85.2)	64	15.4 (11.8, 19.8)	11	3.5 (1.7, 6.8)	181	42.2 (37.1, 47.4)	407	86.2 (82.0, 89.6)	29	7.6 (5.1, 11.2)	24	6.2 (4.1, 9.3)
West-South Central	735	78.4 (75.0, 81.4)	145	18.0 (15.2, 21.1)	23	3.6 (2.3, 5.7)	321	35.6 (32.2, 39.2)	786	85.9 (82.8, 88.5)	62	8.0 (6.1, 10.5)	45	6.1 (4.4, 8.5)
Mountain	616	80.6 (77.0, 83.7)	115	17.3 (14.3, 20.8)	14	2.1 (1.2, 3.8)	221	30.1 (26.6, 34.0)	667	90.4 (87.7, 92.6)	37	5.3 (3.7, 7.5)	33	4.3 (3.0, 6.1)
Pacific	1156	78.4 (75.7, 80.9)	217	17.6 (15.3, 20.1)	41	4.0 (2.9, 5.6)	377	27.7 (25.0, 30.5)	1266	89.3 (87.2, 91.2)	66	5.3 (4.0, 6.9)	62	5.4 (4.1, 7.1)

Table S8, continued

Characteristic	To stop an election from being stolen						To stop people who do not share my beliefs from voting					
	Never justified		Sometimes justified		Usually or always justified		Never justified		Sometimes justified		Usually or always justified	
	Unweighted n	Weighted %, 95% CI	Unweighted n	Weighted %, 95% CI	Unweighted n	Weighted %, 95% CI	Unweighted n	Weighted %, 95% CI	Unweighted n	Weighted %, 95% CI	Unweighted n	Weighted %, 95% CI
**Age, years**												
18-24	333	74.5 (69.8, 78.7)	75	17.0 (13.6, 21.1)	33	8.4 (5.9, 12.0)	404	88.9 (85.0, 91.9)	21	6.3 (4.1, 9.7)	17	4.8 (3.0, 7.6)
25-34	737	72.6 (69.5, 75.5)	197	19.3 (16.8, 22.1)	73	8.0 (6.3, 10.1)	919	89.5 (87.1, 91.5)	57	6.6 (5.1, 8.6)	33	3.9 (2.7, 5.6)
35-44	1003	74.4 (71.9, 76.8)	245	17.8 (15.7, 20.0)	98	7.8 (6.4, 9.5)	1239	90.7 (88.9, 92.3)	76	6.2 (4.9, 7.8)	36	3.1 (2.2, 4.3)
45-54	929	76.3 (73.7, 78.7)	181	15.0 (13.0, 17.2)	95	8.7 (7.1, 10.6)	1130	93.0 (91.2, 94.4)	56	5.0 (3.9, 6.6)	21	2.0 (1.3, 3.1)
55-64	1395	76.6 (74.4, 78.6)	272	15.1 (13.4, 17.0)	146	8.3 (7.0, 9.9)	1725	94.6 (93.2, 95.7)	63	4.0 (3.1, 5.3)	25	1.4 (0.9, 2.1)
65-74	1365	76.7 (74.5, 78.7)	257	14.6 (13.0, 16.4)	147	8.7 (7.4, 10.3)	1705	96.1 (94.9, 97.0)	41	2.5 (1.8, 3.5)	22	1.4 (0.9, 2.2)
75+	650	69.7 (66.4, 72.8)	170	18.4 (15.9, 21.2)	105	11.9 (9.8, 14.4)	909	97.4 (96.1, 98.3)	15	1.8 (1.1, 3.1)	8	0.7 (0.4, 1.5)
**Gender**												
Male	3012	72.8 (71.2, 74.3)	723	17.8 (16.5, 19.2)	367	9.4 (8.4, 10.5)	3891	92.8 (91.7, 93.7)	146	4.6 (3.8, 5.5)	79	2.7 (2.1, 3.4)
Female	3298	76.9 (75.4, 78.3)	640	15.4 (14.2, 16.6)	312	7.8 (6.9, 8.8)	3994	92.6 (91.6, 93.5)	179	5.2 (4.4, 6.0)	77	2.2 (1.7, 2.9)
Other	77	75.0 (64.4, 83.2)	20	18.8 (11.5, 29.2)	8	6.3 (2.9, 13.1)	100	92.7 (81.8, 97.3)	2	3.6 (0.7, 17.1)	4	3.7 (1.2, 10.9)
**Race and ethnicity**												
White, non-Hispanic	4510	75.3 (74.1, 76.4)	1026	17.2 (16.2, 18.3)	444	7.5 (6.8, 8.2)	5766	95.7 (95.1, 96.3)	162	3.1 (2.6, 3.6)	63	1.2 (0.9, 1.5)
Black, non-Hispanic	622	75.6 (72.0, 78.8)	98	13.5 (11.0, 16.5)	90	10.9 (8.7, 13.6)	706	84.5 (81.2, 87.3)	67	9.9 (7.6, 12.8)	41	5.6 (4.0, 7.8)
Hispanic, any race	790	72.7 (69.6, 75.5)	182	17.4 (15.1, 20.0)	98	9.9 (8.0, 12.2)	962	88.7 (86.4, 90.7)	68	6.8 (5.3, 8.6)	41	4.5 (3.3, 6.3)
Asian American / Pacific Islander	231	73.9 (67.9, 79.2)	41	14.6 (10.7, 19.7)	33	11.5 (7.9, 16.4)	275	89.7 (85.0, 93.0)	20	7.3 (4.5, 11.7)	10	3.0 (1.5, 5.8)
Other (American Indian/Alaskan, 2+ races, other)	259	73.1 (65.5, 79.6)	50	16.9 (11.4, 24.4)	32	9.9 (6.4, 15.2)	322	87.9 (80.4, 92.8)	12	6.8 (3.4, 13.1)	7	5.3 (2.3, 11.8)
**Education**												
No high school diploma or GED	351	65.4 (60.8, 69.8)	106	20.6 (17.0, 24.7)	70	14.0 (11.0, 17.7)	445	82.3 (78.3, 85.7)	54	10.9 (8.3, 14.2)	30	6.8 (4.6, 9.9)
High school graduate	1471	69.7 (67.4, 71.8)	394	18.4 (16.6, 20.3)	253	11.9 (10.5, 13.6)	1912	88.7 (87.1, 90.2)	141	7.6 (6.4, 9.1)	65	3.6 (2.8, 4.7)
Some college or Associates degree	1753	75.5 (73.6, 77.4)	377	15.9 (14.3, 17.6)	207	8.6 (7.4, 9.9)	2231	94.8 (93.6, 95.8)	74	3.8 (2.9, 4.9)	34	1.4 (1.0, 2.1)
Bachelors degree	1559	80.8 (78.9, 82.7)	278	14.4 (12.8, 16.2)	96	4.8 (3.8, 5.9)	1874	96.1 (94.9, 97.0)	38	2.3 (1.6, 3.2)	26	1.7 (1.1, 2.5)
Masters degree or higher	1278	80.4 (78.1, 82.4)	242	15.5 (13.6, 17.6)	71	4.1 (3.2, 5.3)	1569	97.8 (96.7, 98.5)	22	1.4 (0.9, 2.3)	7	0.7 (0.3, 1.7)
**Income**												
<$25,000	680	67.5 (64.1, 70.7)	175	18.2 (15.7, 21.1)	140	14.3 (12.0, 16.9)	848	82.2 (79.2, 84.9)	86	10.0 (8.0, 12.4)	62	7.8 (6.0, 10.2)
$25,000 - $49,999	1035	71.4 (68.7, 74.0)	257	18.4 (16.2, 20.9)	155	10.1 (8.6, 12.0)	1334	90.5 (88.4, 92.2)	83	7.1 (5.6, 9.0)	31	2.4 (1.6, 3.6)
$50,000 - $74,999	1056	75.2 (72.5, 77.8)	229	16.5 (14.4, 18.8)	109	8.3 (6.7, 10.3)	1331	94.0 (92.2, 95.4)	46	4.3 (3.1, 6.0)	18	1.7 (1.0, 2.9)
$75,000 - $99,999	889	73.4 (70.4, 76.1)	220	18.9 (16.5, 21.5)	89	7.7 (6.2, 9.7)	1148	94.5 (92.7, 95.9)	32	3.0 (2.0, 4.4)	22	2.5 (1.6, 3.9)
$100,000 - $149,999	1159	77.5 (75.0, 79.8)	239	15.6 (13.7, 17.8)	89	6.8 (5.4, 8.6)	1435	95.1 (93.6, 96.3)	44	3.6 (2.6, 5.0)	13	1.3 (0.7, 2.2)
>$150,000	1593	79.4 (77.3, 81.4)	277	14.2 (12.6, 16.0)	115	6.4 (5.2, 7.8)	1935	96.1 (94.7, 97.1)	38	2.8 (1.9, 4.0)	16	1.1 (0.7, 2.0)
**Census Region**												
New England	318	78.1 (73.2, 82.3)	53	13.2 (9.8, 17.5)	36	8.7 (6.1, 12.3)	383	91.7 (87.5, 94.6)	11	4.3 (2.3, 7.9)	13	4.0 (2.2, 7.1)
Mid-Atlantic	799	73.5 (70.4, 76.4)	191	18.2 (15.7, 21.0)	83	8.3 (6.5, 10.4)	1012	92.7 (90.4, 94.4)	42	5.2 (3.7, 7.1)	20	2.2 (1.3, 3.5)
East-North Central	949	75.7 (73.0, 78.2)	212	16.6 (14.5, 18.9)	93	7.7 (6.2, 9.6)	1180	93.4 (91.6, 94.8)	47	4.5 (3.3, 6.1)	26	2.2 (1.4, 3.3)
West-North Central	446	74.9 (70.9, 78.5)	94	15.1 (12.3, 18.3)	58	10.1 (7.6, 13.3)	574	95.2 (92.5, 96.9)	16	2.9 (1.6, 5.1)	10	2.0 (1.0, 4.1)
South Atlantic	1271	74.0 (71.5, 76.3)	269	17.0 (15.0, 19.2)	150	9.1 (7.6, 10.8)	1591	92.0 (90.1, 93.6)	71	5.8 (4.4, 7.5)	27	2.2 (1.5, 3.4)
East-South Central	337	70.6 (65.4, 75.2)	80	18.3 (14.5, 22.8)	43	11.1 (8.0, 15.2)	431	92.5 (89.3, 94.9)	26	6.4 (4.2, 9.4)	4	1.1 (0.4, 3.1)
West-South Central	654	72.4 (68.9, 75.6)	158	17.9 (15.2, 20.9)	82	9.7 (7.6, 12.3)	835	91.7 (89.1, 93.7)	46	6.1 (4.4, 8.4)	15	2.2 (1.3, 3.8)
Mountain	550	74.7 (71.1, 78.1)	124	16.9 (14.1, 20.2)	65	8.3 (6.4, 10.7)	714	95.6 (93.4, 97.1)	18	2.5 (1.5, 4.2)	10	1.9 (0.9, 3.8)
Pacific	1088	78.0 (75.4, 80.4)	216	15.2 (13.2, 17.5)	87	6.8 (5.4, 8.5)	1311	91.5 (89.4, 93.2)	52	4.4 (3.3, 5.9)	37	4.1 (2.9, 5.9)

Table S8, continued

Characteristic	To prevent discrimination based on race or ethnicity						To preserve an American way of life based on Western European traditions					
	Never justified		Sometimes justified		Usually or always justified		Never justified		Sometimes justified		Usually or always justified	
	Unweighted n	Weighted %, 95% CI	Unweighted n	Weighted %, 95% CI	Unweighted n	Weighted %, 95% CI	Unweighted n	Weighted %, 95% CI	Unweighted n	Weighted %, 95% CI	Unweighted n	Weighted %, 95% CI
**Age, years**												
18-24	231	52.9 (47.9, 57.8)	145	31.7 (27.3, 36.4)	67	15.4 (12.1, 19.4)	344	77.0 (72.5, 81.0)	74	16.8 (13.3, 20.9)	24	6.2 (4.1, 9.3)
25-34	534	52.9 (49.5, 56.3)	355	34.8 (31.6, 38.1)	115	12.3 (10.2, 14.7)	800	78.1 (75.2, 80.9)	154	15.7 (13.4, 18.3)	54	6.2 (4.6, 8.1)
35-44	838	61.4 (58.6, 64.1)	410	30.7 (28.2, 33.4)	101	7.9 (6.5, 9.6)	1060	78.6 (76.2, 80.8)	220	16.1 (14.2, 18.3)	67	5.3 (4.1, 6.7)
45-54	816	67.8 (65.0, 70.5)	293	23.9 (21.5, 26.5)	92	8.2 (6.7, 10.1)	926	77.1 (74.5, 79.5)	220	18.0 (15.9, 20.4)	55	4.9 (3.7, 6.4)
55-64	1264	69.6 (67.3, 71.9)	434	24.3 (22.2, 26.5)	112	6.1 (5.0, 7.4)	1331	73.5 (71.2, 75.6)	387	22.0 (20.0, 24.1)	78	4.6 (3.6, 5.8)
65-74	1258	70.3 (67.9, 72.5)	387	22.0 (20.0, 24.1)	126	7.7 (6.4, 9.2)	1262	71.2 (68.9, 73.4)	395	22.6 (20.6, 24.7)	104	6.3 (5.1, 7.7)
75+	651	69.8 (66.5, 72.9)	212	22.4 (19.6, 25.4)	64	7.8 (6.0, 10.0)	631	68.7 (65.4, 71.9)	212	23.6 (20.8, 26.7)	70	7.6 (6.0, 9.7)
**Gender**												
Male	2695	64.2 (62.5, 65.8)	1101	27.3 (25.7, 28.8)	312	8.5 (7.6, 9.6)	2990	73.4 (71.8, 74.9)	851	20.3 (18.9, 21.7)	256	6.3 (5.5, 7.3)
Female	2815	63.5 (61.8, 65.1)	1086	27.3 (25.8, 28.9)	340	9.2 (8.2, 10.3)	3241	77.1 (75.7, 78.5)	792	18.0 (16.8, 19.3)	185	4.9 (4.2, 5.7)
Other	48	40.6 (30.9, 51.1)	39	38.6 (28.7, 49.5)	19	20.9 (13.3, 31.3)	87	82.1 (71.2, 89.5)	13	11.4 (6.2, 20.2)	5	6.4 (2.1, 17.8)
**Race and ethnicity**												
White, non-Hispanic	4049	66.4 (65.1, 67.7)	1558	26.7 (25.5, 27.9)	373	6.9 (6.2, 7.7)	4391	74.1 (72.9, 75.3)	1249	20.5 (19.4, 21.6)	313	5.4 (4.8, 6.1)
Black, non-Hispanic	459	55.0 (51.1, 58.9)	219	28.4 (25.0, 32.1)	132	16.6 (13.8, 19.7)	625	76.1 (72.5, 79.4)	132	16.6 (13.9, 19.8)	50	7.3 (5.3, 9.9)
Hispanic, any race	694	61.3 (58.0, 64.6)	271	27.7 (24.7, 30.8)	104	11.0 (9.0, 13.3)	836	78.9 (76.0, 81.4)	170	15.4 (13.2, 17.9)	57	5.7 (4.4, 7.5)
Asian American / Pacific Islander	181	55.8 (49.3, 62.1)	88	31.8 (26.0, 38.2)	36	12.4 (8.7, 17.3)	241	78.7 (72.9, 83.6)	49	16.3 (12.0, 21.7)	14	5.0 (2.9, 8.7)
Other (American Indian/Alaskan, 2+ races, other)	209	58.9 (51.3, 66.1)	100	30.7 (24.2, 38.1)	32	10.4 (6.8, 15.4)	261	73.5 (66.0, 79.8)	62	20.7 (15.0, 27.9)	18	5.8 (3.1, 10.5)
**Education**												
No high school diploma or GED	317	58.3 (53.7, 62.9)	131	25.9 (22.0, 30.2)	79	15.8 (12.6, 19.5)	369	70.3 (65.9, 74.3)	113	20.8 (17.3, 24.8)	48	8.9 (6.6, 12.0)
High school graduate	1379	62.4 (60.0, 64.7)	516	26.0 (23.9, 28.2)	218	11.6 (10.1, 13.3)	1453	70.4 (68.2, 72.6)	485	21.8 (19.9, 23.8)	155	7.7 (6.5, 9.2)
Some college or Associates degree	1555	64.0 (61.7, 66.2)	598	27.7 (25.7, 29.9)	189	8.3 (7.1, 9.7)	1676	73.3 (71.3, 75.2)	523	21.3 (19.5, 23.2)	127	5.4 (4.5, 6.6)
Bachelors degree	1281	64.8 (62.4, 67.1)	539	28.5 (26.3, 30.7)	113	6.7 (5.5, 8.2)	1559	81.9 (79.9, 83.6)	300	14.5 (12.9, 16.3)	69	3.6 (2.8, 4.7)
Masters degree or higher	1060	65.6 (63.0, 68.1)	452	29.2 (26.8, 31.7)	78	5.2 (4.1, 6.6)	1297	82.7 (80.7, 84.6)	241	14.2 (12.5, 16.1)	53	3.0 (2.3, 4.1)
**Income**												
<$25,000	587	56.1 (52.6, 59.6)	260	27.4 (24.3, 30.6)	149	16.5 (14.0, 19.4)	665	66.6 (63.2, 69.8)	230	23.7 (20.8, 26.8)	92	9.7 (7.8, 12.1)
$25,000 - $49,999	914	61.1 (58.2, 64.0)	368	26.6 (24.0, 29.3)	160	12.3 (10.4, 14.4)	1030	72.6 (69.9, 75.2)	317	21.2 (18.8, 23.7)	86	6.3 (5.0, 7.9)
$50,000 - $74,999	941	64.1 (61.0, 67.0)	342	26.7 (24.0, 29.5)	110	9.3 (7.6, 11.3)	1024	75.4 (72.7, 77.8)	290	19.1 (16.9, 21.4)	73	5.6 (4.3, 7.3)
$75,000 - $99,999	782	63.0 (59.9, 66.1)	332	28.8 (26.0, 31.8)	89	8.1 (6.5, 10.1)	894	76.1 (73.3, 78.6)	231	18.2 (16.0, 20.8)	69	5.7 (4.4, 7.3)
$100,000 - $149,999	972	63.9 (61.0, 66.6)	443	31.0 (28.4, 33.8)	71	5.1 (4.0, 6.5)	1160	78.0 (75.6, 80.3)	271	17.9 (15.8, 20.2)	54	4.0 (3.0, 5.4)
>$150,000	1396	68.6 (66.2, 70.8)	491	25.1 (23.1, 27.3)	98	6.3 (5.1, 7.8)	1581	79.8 (77.8, 81.7)	323	15.8 (14.2, 17.7)	78	4.3 (3.4, 5.6)
**Census Region**												
New England	266	62.9 (57.3, 68.1)	116	30.4 (25.4, 35.8)	24	6.8 (4.4, 10.3)	309	76.6 (71.7, 80.9)	75	17.7 (14.0, 22.2)	23	5.7 (3.6, 8.8)
Mid-Atlantic	721	65.0 (61.7, 68.1)	262	25.4 (22.6, 28.5)	89	9.6 (7.7, 12.0)	796	76.6 (73.7, 79.3)	210	18.6 (16.1, 21.3)	57	4.8 (3.7, 6.3)
East-North Central	833	65.2 (62.2, 68.1)	325	26.7 (24.1, 29.5)	94	8.0 (6.5, 9.9)	936	74.9 (72.1, 77.4)	242	19.3 (17.0, 21.8)	70	5.8 (4.5, 7.5)
West-North Central	383	60.7 (56.1, 65.0)	161	28.9 (24.9, 33.3)	56	10.4 (7.9, 13.6)	430	72.1 (67.9, 76.0)	137	23.4 (19.8, 27.4)	30	4.5 (3.0, 6.8)
South Atlantic	1122	63.6 (60.9, 66.2)	433	28.0 (25.6, 30.6)	129	8.4 (7.0, 10.1)	1271	75.3 (72.9, 77.6)	312	18.2 (16.2, 20.3)	97	6.5 (5.2, 8.2)
East-South Central	301	64.2 (59.1, 69.0)	110	24.1 (19.9, 28.8)	50	11.7 (8.7, 15.7)	328	69.3 (64.1, 74.0)	103	23.8 (19.5, 28.6)	28	6.9 (4.6, 10.4)
West-South Central	574	60.8 (57.1, 64.4)	241	27.8 (24.6, 31.2)	79	11.4 (9.0, 14.4)	649	73.8 (70.4, 76.9)	187	19.5 (16.8, 22.6)	53	6.7 (5.0, 9.0)
Mountain	484	62.4 (58.3, 66.3)	193	27.7 (24.2, 31.6)	64	9.9 (7.6, 12.8)	545	75.9 (72.4, 79.1)	162	19.9 (17.0, 23.2)	31	4.2 (2.8, 6.1)
Pacific	908	63.8 (60.8, 66.7)	395	28.4 (25.7, 31.3)	92	7.8 (6.2, 9.7)	1090	79.1 (76.6, 81.5)	234	15.8 (13.8, 18.1)	63	5.0 (3.8, 6.6)

Table S8, continued

Characteristic	To preserve the American way of life I believe in						To oppose Americans who do not share my beliefs					
	Never justified		Sometimes justified		Usually or always justified		Never justified		Sometimes justified		Usually or always justified	
	Unweighted n	Weighted %, 95% CI	Unweighted n	Weighted %, 95% CI	Unweighted n	Weighted %, 95% CI	Unweighted n	Weighted %, 95% CI	Unweighted n	Weighted %, 95% CI	Unweighted n	Weighted %, 95% CI
**Age, years**												
18-24	274	61.1 (56.2, 66.0)	122	26.9 (22.5, 31.3)	48	12.0 (8.6, 15.4)	384	84.5 (80.7, 88.3)	41	9.4 (6.5, 12.4)	20	6.1 (3.4, 8.7)
25-34	626	61.0 (57.7, 64.3)	298	29.3 (26.2, 32.4)	92	9.7 (7.7, 11.8)	888	86.0 (83.5, 88.4)	92	9.9 (7.8, 12.0)	35	4.1 (2.6, 5.6)
35-44	810	59.8 (57.0, 62.6)	427	31.3 (28.6, 33.9)	119	8.9 (7.3, 10.5)	1190	86.6 (84.6, 88.6)	128	10.2 (8.4, 11.9)	40	3.3 (2.2, 4.3)
45-54	688	56.8 (53.9, 59.7)	381	31.1 (28.4, 33.8)	141	12.1 (10.2, 14.1)	1095	89.4 (87.5, 91.3)	87	7.8 (6.1, 9.4)	29	2.8 (1.8, 3.9)
55-64	965	52.2 (49.7, 54.7)	628	35.0 (32.6, 37.3)	227	12.8 (11.1, 14.5)	1685	91.5 (90.0, 93.1)	119	7.5 (6.0, 8.9)	17	1.0 (0.4, 1.6)
65-74	916	50.3 (47.9, 52.8)	598	34.3 (31.9, 36.6)	264	15.4 (13.6, 17.2)	1660	92.8 (91.5, 94.1)	98	5.8 (4.6, 7.0)	22	1.4 (0.8, 2.0)
75+	423	45.6 (42.2, 49.0)	346	36.2 (33.0, 39.5)	160	18.2 (15.4, 20.9)	862	92.0 (90.1, 93.9)	55	6.2 (4.5, 7.9)	16	1.7 (0.8, 2.6)
**Gender**												
Male	2101	51.7 (50.0, 53.4)	1450	34.2 (32.6, 35.9)	576	14.0 (12.8, 15.2)	3715	87.9 (86.7, 89.2)	322	9.0 (7.9, 10.1)	92	3.1 (2.4, 3.8)
Female	2506	59.5 (57.9, 61.2)	1305	30.0 (28.5, 31.6)	459	10.5 (9.5, 11.5)	3915	90.0 (88.9, 91.1)	282	7.4 (6.5, 8.3)	80	2.6 (2.0, 3.3)
Other	69	65.6 (55.2, 75.9)	29	24.8 (15.9, 33.8)	8	9.6 (1.9, 17.3)	89	83.6 (75.6, 91.6)	12	13.6 (5.9, 21.4)	5	2.7 (0.3, 5.2)
**Race and ethnicity**												
White, non-Hispanic	3194	53.8 (52.5, 55.2)	2081	34.2 (32.9, 35.5)	732	12.0 (11.1, 12.9)	5568	91.8 (91.1, 92.6)	370	6.7 (6.0, 7.4)	76	1.4 (1.1, 1.8)
Black, non-Hispanic	492	59.4 (55.5, 63.2)	212	26.2 (22.7, 29.6)	117	14.4 (11.6, 17.2)	681	80.8 (77.6, 84.1)	94	12.5 (9.8, 15.2)	46	6.7 (4.5, 8.8)
Hispanic, any race	646	60.8 (57.6, 64.1)	303	27.8 (24.8, 30.8)	125	11.3 (9.3, 13.4)	931	84.7 (82.2, 87.2)	100	9.9 (7.9, 12.0)	45	5.4 (3.7, 7.0)
Asian American / Pacific Islander	192	60.4 (54.1, 66.6)	86	29.4 (23.6, 35.2)	32	10.2 (6.2, 14.2)	271	86.6 (82.2, 91.0)	31	10.6 (6.7, 14.5)	8	2.8 (0.5, 5.2)
Other (American Indian/Alaskan, 2+ races, other)	178	49.6 (41.9, 57.2)	118	34.6 (27.2, 41.9)	45	15.9 (10.3, 21.4)	313	86.3 (80.4, 92.1)	25	10.9 (6.0, 15.9)	4	2.8 (0.0, 6.3)
**Education**												
No high school diploma or GED	281	53.4 (48.8, 58.0)	167	29.9 (25.7, 34.1)	89	16.7 (13.2, 20.3)	433	79.0 (75.1, 82.9)	70	13.3 (10.1, 16.6)	34	7.7 (4.9, 10.4)
High school graduate	1038	50.8 (48.4, 53.2)	705	32.1 (29.8, 34.3)	383	17.1 (15.4, 18.9)	1832	84.1 (82.3, 86.0)	230	11.9 (10.3, 13.5)	68	4.0 (3.0, 5.0)
Some college or Associates degree	1177	52.1 (49.8, 54.3)	860	35.8 (33.7, 38.0)	312	12.1 (10.7, 13.5)	2159	91.0 (89.7, 92.4)	151	6.8 (5.6, 7.9)	43	2.2 (1.4, 3.0)
Bachelors degree	1175	61.7 (59.4, 64.0)	607	30.3 (28.1, 32.5)	162	8.0 (6.7, 9.2)	1825	93.3 (92.0, 94.5)	95	5.3 (4.2, 6.5)	23	1.4 (0.8, 2.0)
Masters degree or higher	1031	65.8 (63.3, 68.3)	461	28.1 (25.7, 30.5)	105	6.1 (4.8, 7.3)	1515	94.1 (92.8, 95.4)	74	5.1 (3.9, 6.4)	11	0.8 (0.3, 1.3)
**Income**												
<$25,000	473	46.7 (43.2, 50.1)	328	33.4 (30.1, 36.7)	207	20.0 (17.2, 22.7)	816	78.4 (75.3, 81.4)	137	14.7 (12.1, 17.2)	55	7.0 (5.0, 8.9)
$25,000 - $49,999	747	54.0 (51.0, 56.9)	471	30.3 (27.6, 32.9)	240	15.8 (13.7, 17.9)	1278	85.7 (83.5, 87.9)	139	10.7 (8.8, 12.7)	40	3.6 (2.3, 4.9)
$50,000 - $74,999	775	57.0 (54.0, 60.0)	464	32.1 (29.3, 34.9)	162	10.9 (9.0, 12.7)	1298	91.1 (89.3, 93.0)	80	6.5 (4.9, 8.1)	26	2.3 (1.3, 3.4)
$75,000 - $99,999	670	57.0 (53.9, 60.1)	384	30.4 (27.5, 33.2)	148	12.7 (10.5, 14.8)	1104	90.3 (88.3, 92.3)	80	7.3 (5.6, 9.1)	21	2.4 (1.3, 3.5)
$100,000 - $149,999	841	56.4 (53.6, 59.3)	517	34.3 (31.6, 37.0)	135	9.3 (7.6, 10.9)	1392	91.6 (89.9, 93.4)	86	6.9 (5.3, 8.4)	16	1.5 (0.7, 2.4)
>$150,000	1196	60.6 (58.2, 62.9)	636	31.3 (29.1, 33.5)	159	8.1 (6.7, 9.6)	1876	92.5 (91.1, 94.0)	98	5.8 (4.6, 7.0)	21	1.7 (0.8, 2.5)
**Census Region**												
New England	252	62.5 (57.2, 67.9)	112	27.1 (22.2, 32.0)	44	10.4 (7.0, 13.7)	370	88.8 (84.9, 92.6)	29	8.6 (5.1, 12.2)	9	2.6 (0.9, 4.4)
Mid-Atlantic	609	58.2 (55.0, 61.5)	329	29.2 (26.2, 32.2)	136	12.5 (10.3, 14.8)	984	89.7 (87.5, 91.9)	72	7.9 (6.0, 9.8)	21	2.4 (1.2, 3.5)
East-North Central	672	55.2 (52.2, 58.2)	437	33.6 (30.8, 36.4)	153	11.2 (9.4, 13.0)	1148	90.1 (88.3, 92.0)	90	7.5 (5.9, 9.1)	23	2.4 (1.3, 3.5)
West-North Central	305	50.4 (45.9, 54.8)	210	36.2 (31.9, 40.5)	86	13.4 (10.4, 16.4)	549	90.1 (87.3, 92.9)	40	7.5 (5.1, 9.9)	12	2.4 (0.8, 3.9)
South Atlantic	931	54.7 (52.0, 57.4)	550	32.0 (29.5, 34.5)	220	13.3 (11.4, 15.2)	1546	88.9 (87.0, 90.7)	124	8.5 (6.9, 10.1)	32	2.6 (1.5, 3.6)
East-South Central	227	48.0 (42.8, 53.1)	161	34.1 (29.2, 39.0)	73	17.9 (13.8, 22.1)	411	87.0 (83.3, 90.8)	41	9.4 (6.4, 12.5)	10	3.6 (1.1, 6.0)
West-South Central	471	54.0 (50.3, 57.7)	302	32.3 (29.0, 35.7)	127	13.7 (11.1, 16.2)	796	86.9 (84.3, 89.6)	81	9.2 (7.0, 11.4)	24	3.9 (2.2, 5.6)
Mountain	396	56.2 (52.2, 60.1)	260	32.1 (28.5, 35.8)	86	11.7 (9.1, 14.3)	689	91.2 (88.7, 93.7)	44	7.0 (4.7, 9.3)	10	1.8 (0.6, 3.0)
Pacific	839	60.5 (57.5, 63.5)	439	30.9 (28.0, 33.7)	126	8.6 (7.0, 10.3)	1271	87.6 (85.4, 89.8)	99	8.7 (6.8, 10.5)	38	3.7 (2.4, 5.0)

Table 8, continued

Characteristic	To oppose the government when it does not share my beliefs						To oppose the government when it tries to take private land for public purposes					
	Never justified		Sometimes justified		Usually or always justified		Never justified		Sometimes justified		Usually or always justified	
	Unweighted n	Weighted %, 95% CI	Unweighted n	Weighted %, 95% CI	Unweighted n	Weighted %, 95% CI	Unweighted n	Weighted %, 95% CI	Unweighted n	Weighted %, 95% CI	Unweighted n	Weighted %, 95% CI
**Age, years**												
18-24	322	71.4 (66.6, 75.8)	95	22.7 (18.7, 27.3)	26	5.9 (3.9, 8.7)	252	57.0 (51.9, 61.8)	130	28.9 (24.6, 33.7)	60	14.1 (10.9, 18.1)
25-34	740	73.0 (69.9, 75.9)	213	21.0 (18.4, 23.9)	51	6.0 (4.5, 8.0)	565	55.8 (52.4, 59.1)	317	31.0 (27.9, 34.2)	124	13.2 (11.0, 15.8)
35-44	1065	79.1 (76.7, 81.3)	227	16.5 (14.5, 18.7)	56	4.4 (3.3, 5.8)	812	60.3 (57.5, 63.0)	399	29.2 (26.7, 31.9)	136	10.5 (8.9, 12.4)
45-54	1021	84.7 (82.5, 86.8)	152	12.7 (10.8, 14.8)	27	2.6 (1.8, 3.8)	776	64.6 (61.7, 67.4)	322	26.4 (23.9, 29.1)	104	9.0 (7.4, 10.8)
55-64	1575	86.1 (84.3, 87.8)	205	12.0 (10.4, 13.8)	34	1.8 (1.3, 2.6)	1167	64.0 (61.6, 66.4)	523	29.5 (27.2, 31.8)	121	6.5 (5.4, 7.9)
65-74	1534	86.5 (84.7, 88.1)	205	11.8 (10.3, 13.5)	31	1.7 (1.2, 2.5)	1164	64.9 (62.4, 67.2)	479	27.5 (25.4, 29.8)	121	7.6 (6.3, 9.1)
75+	798	85.6 (82.9, 87.9)	107	11.8 (9.7, 14.3)	23	2.6 (1.7, 3.9)	594	63.7 (60.3, 66.9)	253	26.7 (23.8, 29.8)	79	9.7 (7.7, 12.0)
**Gender**												
Male	3333	79.1 (77.6, 80.5)	652	17.1 (15.8, 18.6)	126	3.8 (3.1, 4.5)	2533	60.5 (58.8, 62.2)	1209	29.4 (27.9, 31.0)	361	10.0 (9.0, 11.2)
Female	3613	83.2 (81.9, 84.5)	518	13.7 (12.5, 14.9)	110	3.1 (2.5, 3.8)	2711	62.5 (60.9, 64.2)	1169	27.9 (26.4, 29.5)	361	9.6 (8.5, 10.7)
Other	68	61.5 (50.4, 71.5)	26	26.0 (17.6, 36.6)	11	12.5 (6.4, 23.1)	64	58.3 (47.2, 68.6)	26	27.3 (18.5, 38.2)	14	14.4 (7.9, 24.8)
**Race and ethnicity**												
White, non-Hispanic	5013	82.3 (81.2, 83.3)	830	15.0 (14.0, 16.0)	141	2.8 (2.3, 3.3)	3738	61.2 (59.9, 62.6)	1747	29.5 (28.3, 30.8)	488	9.2 (8.4, 10.1)
Black, non-Hispanic	656	79.0 (75.5, 82.1)	109	14.3 (11.7, 17.4)	45	6.7 (4.8, 9.2)	530	63.3 (59.4, 67.0)	197	25.5 (22.1, 29.1)	83	11.2 (8.9, 14.2)
Hispanic, any race	877	80.0 (77.1, 82.6)	149	15.4 (13.1, 18.0)	43	4.6 (3.4, 6.4)	670	61.8 (58.6, 65.0)	289	27.2 (24.3, 30.2)	111	11.0 (9.0, 13.3)
Asian American / Pacific Islander	247	78.3 (72.2, 83.5)	48	19.0 (14.1, 25.1)	9	2.7 (1.3, 5.4)	197	61.6 (55.0, 67.7)	84	30.2 (24.4, 36.6)	24	8.2 (5.3, 12.6)
Other (American Indian/Alaskan, 2+ races, other)	262	70.3 (62.3, 77.3)	68	25.4 (18.7, 33.4)	10	4.3 (2.1, 8.7)	195	55.1 (47.4, 62.6)	106	29.0 (22.8, 36.1)	39	15.9 (10.6, 23.2)
**Education**												
No high school diploma or GED	408	74.6 (70.2, 78.6)	83	17.6 (14.2, 21.7)	39	7.8 (5.6, 10.7)	304	58.3 (53.7, 62.9)	140	26.4 (22.5, 30.8)	84	15.2 (12.2, 18.9)
High school graduate	1685	78.2 (76.1, 80.1)	334	16.5 (14.7, 18.4)	92	5.4 (4.3, 6.7)	1183	55.1 (52.7, 57.5)	645	30.2 (28.1, 32.4)	283	14.7 (13.0, 16.6)
Some college or Associates degree	1926	80.6 (78.6, 82.4)	352	17.0 (15.3, 18.9)	58	2.4 (1.8, 3.2)	1390	59.2 (56.9, 61.4)	726	31.4 (29.3, 33.6)	217	9.4 (8.2, 10.8)
Bachelors degree	1643	83.9 (81.9, 85.6)	250	13.7 (12.1, 15.6)	40	2.4 (1.7, 3.3)	1315	67.1 (64.7, 69.4)	519	27.1 (25.0, 29.3)	100	5.8 (4.7, 7.1)
Masters degree or higher	1393	86.5 (84.5, 88.3)	185	12.1 (10.4, 14.0)	19	1.4 (0.8, 2.4)	1138	71.3 (68.9, 73.7)	393	24.5 (22.3, 26.8)	61	4.2 (3.2, 5.5)
**Income**												
<$25,000	743	72.6 (69.3, 75.7)	180	18.9 (16.2, 21.9)	74	8.5 (6.6, 10.8)	551	54.2 (50.7, 57.7)	281	28.0 (25.0, 31.3)	163	17.7 (15.2, 20.6)
$25,000 - $49,999	1154	78.1 (75.4, 80.6)	237	17.6 (15.4, 20.1)	51	4.3 (3.1, 5.8)	829	57.3 (54.3, 60.2)	430	28.9 (26.3, 31.6)	185	13.9 (11.9, 16.1)
$50,000 - $74,999	1160	82.2 (79.6, 84.5)	193	14.6 (12.5, 16.9)	37	3.3 (2.3, 4.7)	852	59.7 (56.7, 62.6)	424	30.2 (27.5, 33.1)	119	10.1 (8.3, 12.3)
$75,000 - $99,999	991	80.4 (77.7, 82.9)	181	16.4 (14.2, 19.0)	31	3.2 (2.2, 4.6)	735	60.7 (57.5, 63.7)	363	30.5 (27.7, 33.5)	100	8.9 (7.2, 10.9)
$100,000 - $149,999	1277	83.4 (81.0, 85.6)	191	14.9 (12.9, 17.2)	20	1.7 (1.1, 2.7)	987	64.5 (61.7, 67.2)	410	28.4 (25.8, 31.1)	89	7.1 (5.7, 8.9)
>$150,000	1730	85.1 (83.1, 86.9)	222	12.6 (11.0, 14.4)	35	2.3 (1.6, 3.4)	1376	67.7 (65.3, 70.0)	515	27.0 (24.9, 29.2)	89	5.3 (4.2, 6.7)
**Census Region**												
New England	338	81.6 (76.7, 85.7)	53	13.5 (10.0, 17.9)	16	4.9 (2.9, 8.2)	268	64.2 (58.6, 69.4)	103	26.3 (21.6, 31.6)	33	9.6 (6.6, 13.6)
Mid-Atlantic	908	83.0 (80.2, 85.4)	135	13.9 (11.6, 16.5)	29	3.2 (2.1, 4.7)	679	62.7 (59.4, 65.9)	308	28.8 (25.8, 32.0)	85	8.5 (6.8, 10.6)
East-North Central	1053	82.7 (80.2, 85.0)	165	14.3 (12.2, 16.7)	34	3.0 (2.1, 4.2)	756	60.5 (57.4, 63.4)	383	30.4 (27.7, 33.3)	112	9.1 (7.5, 11.0)
West-North Central	495	80.2 (76.1, 83.8)	86	16.5 (13.2, 20.4)	17	3.3 (1.9, 5.6)	356	56.8 (52.3, 61.2)	178	30.3 (26.3, 34.5)	63	13.0 (9.9, 16.7)
South Atlantic	1411	81.6 (79.3, 83.7)	233	15.3 (13.3, 17.4)	43	3.2 (2.3, 4.4)	1099	63.1 (60.4, 65.7)	455	27.2 (24.9, 29.7)	135	9.7 (8.0, 11.6)
East-South Central	370	78.0 (73.1, 82.3)	72	16.6 (12.9, 21.0)	18	5.4 (3.2, 8.9)	281	58.9 (53.7, 63.9)	129	27.4 (23.1, 32.1)	50	13.7 (10.2, 18.3)
West-South Central	708	77.5 (74.1, 80.6)	163	19.3 (16.5, 22.5)	21	3.2 (2.0, 5.1)	537	59.4 (55.7, 63.0)	252	29.1 (25.8, 32.6)	100	11.5 (9.3, 14.0)
Mountain	591	79.6 (76.1, 82.6)	124	16.6 (13.8, 19.7)	26	3.9 (2.5, 5.9)	451	60.5 (56.5, 64.4)	229	30.7 (27.1, 34.5)	62	8.8 (6.7, 11.4)
Pacific	1181	81.2 (78.5, 83.7)	173	14.6 (12.4, 17.2)	44	4.1 (3.0, 5.8)	903	62.9 (59.9, 65.9)	386	28.1 (25.4, 30.9)	105	9.0 (7.3, 11.0)

Table S9. Variation with respondent characteristics in beliefs concerning justification for political violence for 8 additional specific objectives

Characteristic	Stop voter fraud						Stop voter intimidation					
	Never justified		Sometimes justified		Usually or always justified		Never justified		Sometimes justified		Usually or always justified	
	Unweighted n	Weighted %, 95% CI	Unweighted n	Weighted %, 95% CI	Unweighted n	Weighted %, 95% CI	Unweighted n	Weighted %, 95% CI	Unweighted n	Weighted %, 95% CI	Unweighted n	Weighted %, 95% CI
**Age, years**												
18-24	160	73.7 (67.4, 79.9)	39	16.3 (11.2, 21.4)	21	10.0 (5.5, 14.5)	135	60.4 (53.5, 67.2)	67	28.1 (21.9, 34.3)	22	11.5 (6.7, 16.4)
25-34	387	74.9 (70.6, 79.2)	83	18.2 (14.4, 22.0)	29	6.9 (4.2, 9.6)	297	58.8 (54.2, 63.4)	169	31.4 (27.0, 35.7)	50	9.8 (7.1, 12.6)
35-44	545	74.4 (71.0, 77.9)	121	17.5 (14.5, 20.5)	52	8.1 (5.8, 10.3)	400	63.2 (59.2, 67.1)	189	29.1 (25.3, 32.8)	50	7.8 (5.6, 10.0)
45-54	465	76.4 (72.7, 80.0)	79	13.9 (10.9, 16.9)	54	9.7 (7.1, 12.3)	403	65.7 (61.8, 69.7)	166	27.0 (23.3, 30.7)	44	7.3 (5.1, 9.4)
55-64	710	77.2 (74.2, 80.1)	109	13.1 (10.6, 15.6)	90	9.7 (7.7, 11.8)	580	63.8 (60.4, 67.2)	244	26.3 (23.2, 29.4)	87	9.9 (7.7, 12.1)
65-74	622	71.4 (68.2, 74.5)	142	16.7 (14.1, 19.4)	101	11.9 (9.6, 14.2)	544	58.9 (55.5, 62.3)	243	26.6 (23.6, 29.6)	129	14.5 (12.0, 16.9)
75+	315	66.8 (62.2, 71.4)	89	19.2 (15.4, 23.0)	63	14.0 (10.6, 17.5)	260	56.2 (51.4, 61.1)	129	27.9 (23.5, 32.3)	76	15.9 (12.4, 19.3)
**Gender**												
Male	1545	73.5 (71.3, 75.7)	321	16.2 (14.4, 18.1)	214	10.3 (8.8, 11.8)	1201	59.1 (56.7, 61.5)	613	29.6 (27.4, 31.8)	235	11.3 (9.7, 12.9)
Female	1602	74.8 (72.7, 76.9)	332	16.5 (14.7, 18.3)	187	8.8 (7.4, 10.2)	1384	64.6 (62.3, 66.9)	562	26.1 (24.0, 28.2)	207	9.3 (8.0, 10.7)
Other	42	80.1 (68.1, 92.2)	5	9.3 (1.2, 17.5)	5	10.5 (0.7, 20.4)	19	31.9 (18.5, 45.3)	25	51.2 (36.2, 66.2)	10	16.9 (6.1, 27.7)
**Race and ethnicity**												
White, non-Hispanic	2276	76.1 (74.5, 77.7)	451	15.1 (13.7, 16.5)	279	8.8 (7.8, 9.9)	1830	61.4 (59.5, 63.3)	865	28.8 (27.1, 30.5)	311	9.8 (8.7, 10.9)
Black, non-Hispanic	289	67.2 (61.8, 72.6)	62	17.4 (13.0, 21.8)	59	15.4 (11.1, 19.7)	247	60.5 (55.1, 65.8)	111	28.4 (23.3, 33.4)	54	11.2 (8.1, 14.3)
Hispanic, any race	400	73.0 (68.8, 77.1)	94	18.3 (14.7, 21.9)	44	8.7 (6.0, 11.4)	354	65.6 (61.2, 70.1)	123	22.6 (18.7, 26.5)	60	11.8 (8.7, 14.9)
Asian American / Pacific Islander	116	72.5 (64.2, 80.7)	24	18.0 (10.8, 25.1)	14	9.6 (4.0, 15.1)	87	56.0 (47.1, 64.9)	50	29.6 (21.7, 37.6)	18	14.4 (7.3, 21.5)
Other (American Indian/Alaskan, 2+ races, other)	123	70.4 (60.3, 80.5)	31	21.4 (12.1, 30.7)	14	8.2 (2.4, 14.0)	101	53.4 (42.7, 64.1)	58	39.3 (28.6, 50.0)	15	7.3 (2.5, 12.2)
**Education**												
No high school diploma or GED	167	61.9 (55.4, 68.4)	55	20.4 (15.1, 25.6)	44	17.8 (12.5, 23.1)	173	65.6 (59.5, 71.7)	69	23.9 (18.5, 29.2)	29	10.6 (6.5, 14.6)
High school graduate	700	66.0 (62.8, 69.2)	218	20.9 (18.1, 23.6)	143	13.1 (10.8, 15.4)	635	59.4 (56.0, 62.7)	283	26.4 (23.4, 29.4)	150	14.2 (11.8, 16.6)
Some college or Associates degree	857	75.2 (72.5, 77.9)	177	15.2 (12.9, 17.5)	130	9.6 (7.9, 11.3)	738	62.6 (59.5, 65.7)	322	27.8 (24.9, 30.7)	127	9.6 (7.8, 11.5)
Bachelors degree	782	82.7 (80.0, 85.4)	113	12.5 (10.1, 14.9)	50	4.8 (3.4, 6.2)	624	62.4 (59.2, 65.7)	289	29.8 (26.6, 32.9)	85	7.8 (6.0, 9.5)
Masters degree or higher	698	82.9 (80.0, 85.8)	99	12.2 (9.6, 14.8)	43	4.9 (3.3, 6.4)	449	59.2 (55.4, 63.0)	244	32.5 (28.8, 36.1)	67	8.3 (6.2, 10.4)
**Income**												
<$25,000	333	62.5 (57.8, 67.3)	85	18.0 (14.2, 21.8)	95	19.4 (15.4, 23.4)	277	55.3 (50.4, 60.3)	144	30.7 (26.1, 35.4)	72	14.0 (10.6, 17.4)
$25,000.0 - $49,999	481	66.7 (62.6, 70.8)	138	21.3 (17.7, 24.9)	92	12.0 (9.3, 14.7)	461	62.3 (58.3, 66.2)	197	27.0 (23.3, 30.6)	88	10.8 (8.4, 13.1)
$50.0,000.0 - $74,999	507	73.7 (69.8, 77.5)	109	16.1 (13.0, 19.3)	67	10.2 (7.4, 13.0)	456	64.1 (60.0, 68.2)	192	25.6 (21.9, 29.2)	73	10.3 (7.6, 13.0)
$75,000.0 - $99,999	455	75.5 (71.5, 79.4)	85	15.3 (12.0, 18.6)	56	9.2 (6.6, 11.8)	360	60.7 (56.4, 65.0)	178	27.7 (23.9, 31.6)	70	11.6 (8.7, 14.5)
$100.0,000.0 - $149,999	604	77.8 (74.4, 81.1)	118	16.2 (13.2, 19.2)	47	6.0 (4.1, 8.0)	451	62.5 (58.5, 66.4)	204	28.4 (24.7, 32.1)	70	9.1 (6.9, 11.4)
>$150.0,000	824	82.5 (79.9, 85.0)	127	12.2 (10.0, 14.4)	53	5.3 (3.8, 6.9)	614	62.0 (58.7, 65.4)	292	29.3 (26.2, 32.4)	85	8.7 (6.6, 10.7)
**Census Region**												
New England	154	77.1 (70.4, 83.8)	28	14.3 (8.6, 20.1)	16	8.5 (4.3, 12.8)	130	63.3 (56.0, 70.7)	59	26.1 (19.6, 32.7)	21	10.5 (5.6, 15.5)
Mid-Atlantic	387	73.7 (69.3, 78.0)	83	15.8 (12.3, 19.3)	55	10.5 (7.3, 13.7)	337	60.9 (56.4, 65.4)	152	28.3 (24.1, 32.5)	61	10.8 (8.0, 13.5)
East-North Central	454	72.5 (68.7, 76.4)	98	16.2 (12.9, 19.4)	75	11.3 (8.7, 13.9)	381	60.8 (56.7, 65.0)	179	27.8 (24.0, 31.6)	74	11.3 (8.7, 14.0)
West-North Central	219	72.2 (66.8, 77.6)	45	14.8 (10.6, 19.1)	41	13.0 (9.0, 16.9)	181	58.9 (52.5, 65.4)	86	30.0 (24.1, 35.9)	28	11.1 (6.2, 16.0)
South Atlantic	608	69.9 (66.4, 73.5)	147	18.9 (15.9, 22.0)	89	11.1 (8.5, 13.7)	548	64.2 (60.6, 67.8)	220	26.6 (23.2, 29.9)	91	9.2 (7.2, 11.2)
East-South Central	181	73.9 (67.1, 80.6)	35	15.5 (10.0, 21.1)	20	10.6 (5.6, 15.6)	126	58.1 (50.9, 65.2)	71	30.4 (23.8, 37.0)	29	11.6 (7.1, 16.0)
West-South Central	353	74.5 (70.1, 78.9)	74	17.0 (13.1, 20.8)	43	8.6 (5.8, 11.3)	262	61.5 (56.2, 66.7)	119	26.9 (22.2, 31.6)	51	11.6 (8.0, 15.2)
Mountain	288	81.0 (76.4, 85.6)	50	13.3 (9.4, 17.2)	23	5.7 (2.9, 8.6)	239	61.6 (56.1, 67.2)	109	29.1 (23.9, 34.3)	34	9.3 (5.9, 12.6)
Pacific	560	77.7 (74.1, 81.3)	102	15.5 (12.3, 18.6)	48	6.8 (4.7, 9.0)	415	60.2 (55.9, 64.5)	212	29.6 (25.6, 33.5)	69	10.2 (7.5, 13.0)

Table S9, continued

Characteristic	Reinforce the police						Stop police violence					
	Never justified		Sometimes justified		Usually or always justified		Never justified		Sometimes justified		Usually or always justified	
	Unweighted n	Weighted %, 95% CI	Unweighted n	Weighted %, 95% CI	Unweighted n	Weighted %, 95% CI	Unweighted n	Weighted %, 95% CI	Unweighted n	Weighted %, 95% CI	Unweighted n	Weighted %, 95% CI
**Age, years**												
18-24	126	54.2 (47.2, 61.2)	67	30.4 (23.9, 36.9)	32	15.3 (10.1, 20.6)	75	33.2 (26.6, 39.9)	115	52.3 (45.2, 59.4)	29	14.5 (9.3, 19.6)
25-34	279	53.0 (48.3, 57.8)	169	35.0 (30.4, 39.6)	55	12.0 (8.8, 15.1)	199	39.8 (35.1, 44.6)	225	43.0 (38.3, 47.7)	89	17.2 (13.6, 20.7)
35-44	318	47.4 (43.3, 51.4)	273	41.1 (37.1, 45.1)	76	11.5 (9.0, 14.1)	308	44.7 (40.8, 48.7)	304	43.8 (39.8, 47.7)	78	11.5 (9.0, 14.0)
45-54	266	44.1 (39.9, 48.2)	231	38.1 (34.0, 42.2)	98	17.8 (14.5, 21.2)	324	52.2 (48.0, 56.3)	225	36.2 (32.3, 40.2)	66	11.6 (8.9, 14.3)
55-64	321	35.9 (32.5, 39.2)	388	44.5 (41.0, 48.0)	177	19.7 (16.9, 22.4)	483	50.7 (47.3, 54.2)	356	38.5 (35.1, 41.9)	92	10.7 (8.4, 13.0)
65-74	300	31.8 (28.6, 34.9)	369	40.5 (37.1, 43.8)	247	27.8 (24.7, 30.9)	454	51.6 (48.1, 55.2)	320	37.5 (34.0, 40.9)	90	10.9 (8.6, 13.1)
75+	111	23.5 (19.4, 27.6)	210	44.7 (39.8, 49.6)	137	31.8 (27.0, 36.6)	214	43.1 (38.4, 47.8)	204	43.3 (38.6, 48.0)	57	13.6 (10.1, 17.1)
**Gender**												
Male	794	40.1 (37.7, 42.5)	851	40.2 (37.8, 42.6)	434	19.7 (17.8, 21.6)	1065	49.6 (47.2, 52.1)	779	39.1 (36.7, 41.5)	204	11.2 (9.6, 12.9)
Female	883	43.8 (41.4, 46.2)	835	39.0 (36.6, 41.3)	377	17.3 (15.5, 19.1)	969	43.0 (40.6, 45.3)	932	43.5 (41.2, 45.9)	277	13.5 (11.8, 15.2)
Other	33	62.4 (47.0, 77.8)	13	26.6 (12.1, 41.1)	6	11.0 (1.3, 20.7)	17	27.8 (15.3, 40.2)	23	43.4 (29.1, 57.7)	14	28.8 (15.2, 42.5)
**Race and ethnicity**												
White, non-Hispanic	1132	39.4 (37.5, 41.3)	1256	40.5 (38.6, 42.3)	636	20.1 (18.6, 21.6)	1445	46.9 (44.9, 48.8)	1241	42.6 (40.7, 44.5)	299	10.5 (9.3, 11.7)
Black, non-Hispanic	198	48.8 (43.3, 54.3)	160	38.7 (33.3, 44.1)	55	12.5 (8.9, 16.0)	175	40.0 (34.6, 45.3)	150	38.9 (33.5, 44.4)	83	21.1 (16.6, 25.7)
Hispanic, any race	260	50.4 (45.7, 55.2)	174	32.4 (28.0, 36.8)	89	17.1 (13.5, 20.7)	282	48.6 (44.0, 53.3)	198	36.5 (32.1, 41.0)	73	14.8 (11.4, 18.3)
Asian American / Pacific Islander	64	39.9 (31.0, 48.8)	55	43.6 (34.2, 53.1)	21	16.5 (8.8, 24.1)	77	40.7 (32.4, 48.9)	74	47.7 (39.1, 56.3)	18	11.6 (5.7, 17.5)
Other (American Indian/Alaskan, 2+ races, other)	67	37.0 (26.5, 47.4)	62	47.8 (36.2, 59.5)	21	15.2 (7.3, 23.0)	78	38.2 (28.3, 48.1)	86	46.3 (36.1, 56.5)	28	15.5 (8.7, 22.3)
**Education**												
No high school diploma or GED	98	42.8 (35.9, 49.6)	86	34.9 (28.3, 41.5)	55	22.3 (16.5, 28.1)	122	41.7 (35.5, 48.0)	114	37.5 (31.5, 43.6)	61	20.8 (15.7, 25.9)
High school graduate	378	38.2 (34.8, 41.5)	425	37.9 (34.7, 41.2)	272	23.9 (21.0, 26.7)	479	44.7 (41.3, 48.1)	410	40.0 (36.7, 43.4)	165	15.2 (12.8, 17.7)
Some college or Associates degree	410	37.1 (34.0, 40.2)	510	42.7 (39.6, 45.9)	273	20.1 (17.8, 22.5)	545	44.4 (41.2, 47.6)	487	43.7 (40.5, 47.0)	125	11.9 (9.7, 14.1)
Bachelors degree	443	47.7 (44.2, 51.1)	370	39.1 (35.7, 42.5)	145	13.2 (11.0, 15.4)	495	47.9 (44.4, 51.3)	396	41.6 (38.2, 45.0)	94	10.6 (8.4, 12.8)
Masters degree or higher	392	52.5 (48.7, 56.2)	316	38.3 (34.6, 41.9)	77	9.3 (7.1, 11.4)	416	49.5 (45.8, 53.2)	342	43.2 (39.5, 46.9)	56	7.3 (5.3, 9.3)
**Income**												
<$25,000	200	40.4 (35.6, 45.2)	182	35.2 (30.5, 39.8)	126	24.5 (20.3, 28.7)	199	38.9 (34.0, 43.7)	195	40.5 (35.6, 45.5)	105	20.6 (16.6, 24.5)
$25,000.0 - $49,999	260	39.7 (35.5, 43.8)	292	39.3 (35.3, 43.4)	177	21.0 (17.8, 24.2)	315	41.8 (37.6, 45.9)	297	41.3 (37.2, 45.5)	115	16.9 (13.7, 20.1)
$50.0,000.0 - $74,999	261	39.4 (35.2, 43.6)	305	41.4 (37.2, 45.5)	135	19.2 (15.7, 22.7)	346	48.1 (43.9, 52.3)	280	40.3 (36.1, 44.4)	77	11.6 (8.8, 14.4)
$75,000.0 - $99,999	241	43.8 (39.2, 48.3)	240	37.7 (33.4, 42.0)	117	18.5 (15.2, 21.9)	286	45.1 (40.7, 49.5)	246	40.8 (36.4, 45.1)	73	14.2 (10.8, 17.5)
$100.0,000.0 - $149,999	326	45.0 (40.9, 49.0)	295	40.2 (36.2, 44.2)	113	14.8 (12.1, 17.6)	387	48.2 (44.2, 52.2)	308	43.1 (39.1, 47.1)	64	8.7 (6.5, 10.9)
>$150.0,000	433	44.7 (41.3, 48.1)	393	40.2 (36.9, 43.6)	154	15.1 (12.6, 17.5)	524	49.1 (45.7, 52.5)	423	42.5 (39.1, 45.9)	67	8.4 (6.2, 10.6)
**Census Region**												
New England	90	45.1 (37.5, 52.8)	81	39.2 (31.7, 46.7)	37	15.7 (10.4, 20.9)	91	40.4 (32.9, 47.9)	84	45.2 (37.4, 53.1)	25	14.4 (8.5, 20.3)
Mid-Atlantic	204	42.9 (38.0, 47.8)	204	38.4 (33.7, 43.1)	100	18.6 (14.9, 22.4)	269	45.9 (41.4, 50.5)	240	44.1 (39.5, 48.7)	58	10.0 (7.3, 12.7)
East-North Central	261	42.4 (38.2, 46.6)	251	38.2 (34.1, 42.2)	128	19.4 (16.1, 22.7)	301	47.2 (42.8, 51.6)	249	41.0 (36.7, 45.4)	70	11.7 (8.9, 14.6)
West-North Central	109	35.9 (30.1, 41.8)	119	39.4 (33.3, 45.4)	78	24.7 (19.2, 30.2)	129	42.0 (35.8, 48.2)	137	47.0 (40.7, 53.4)	29	11.0 (6.7, 15.3)
South Atlantic	369	42.1 (38.5, 45.7)	350	40.0 (36.4, 43.7)	169	17.9 (15.1, 20.7)	393	46.0 (42.2, 49.8)	315	39.5 (35.7, 43.2)	106	14.5 (11.7, 17.4)
East-South Central	79	33.5 (26.5, 40.4)	95	42.1 (34.9, 49.3)	53	24.4 (18.1, 30.7)	95	39.8 (32.8, 46.9)	99	41.7 (34.5, 48.9)	41	18.4 (12.6, 24.3)
West-South Central	171	43.8 (38.6, 49.1)	170	36.8 (31.9, 41.7)	91	19.4 (15.3, 23.4)	215	43.8 (38.7, 48.9)	179	38.7 (33.8, 43.7)	76	17.5 (13.4, 21.5)
Mountain	128	40.7 (34.8, 46.6)	171	44.9 (39.1, 50.6)	56	14.5 (10.5, 18.4)	194	47.5 (41.9, 53.2)	160	42.9 (37.3, 48.5)	34	9.6 (6.2, 12.9)
Pacific	310	46.8 (42.4, 51.2)	266	38.0 (33.7, 42.3)	110	15.1 (12.1, 18.2)	370	49.5 (45.3, 53.7)	286	40.9 (36.7, 45.1)	62	9.6 (7.0, 12.2)

Table S9, continued

Characteristic	Stop illegal immigration						Keep borders open					
	Never justified		Sometimes justified		Usually or always justified		Never justified		Sometimes justified		Usually or always justified	
	Unweighted n	Weighted %, 95% CI	Unweighted n	Weighted %, 95% CI	Unweighted n	Weighted %, 95% CI	Unweighted n	Weighted %, 95% CI	Unweighted n	Weighted %, 95% CI	Unweighted n	Weighted %, 95% CI
**Age, years**												
18-24	143	62.2 (55.2, 69.2)	55	26.1 (19.7, 32.5)	25	11.7 (7.0, 16.4)	141	62.4 (55.5, 69.3)	62	28.1 (21.8, 34.5)	18	9.5 (5.2, 13.8)
25-34	354	66.3 (61.9, 70.7)	127	24.5 (20.5, 28.4)	47	9.3 (6.5, 12.0)	328	64.2 (59.4, 69.0)	121	27.2 (22.7, 31.7)	38	8.6 (5.8, 11.4)
35-44	460	68.5 (64.8, 72.2)	161	23.5 (20.1, 26.9)	51	8.0 (5.8, 10.2)	489	70.1 (66.4, 73.8)	154	23.4 (20.0, 26.8)	40	6.5 (4.4, 8.6)
45-54	374	60.1 (56.0, 64.2)	163	27.0 (23.3, 30.7)	73	12.9 (10.1, 15.8)	435	72.7 (69.0, 76.5)	128	20.9 (17.4, 24.3)	36	6.4 (4.3, 8.5)
55-64	520	56.4 (52.9, 59.8)	282	31.0 (27.8, 34.3)	119	12.6 (10.3, 14.9)	607	66.5 (63.2, 69.8)	236	26.7 (23.6, 29.8)	58	6.8 (5.0, 8.7)
65-74	550	59.9 (56.6, 63.3)	234	26.3 (23.2, 29.3)	125	13.8 (11.4, 16.1)	566	64.2 (60.8, 67.6)	224	25.8 (22.8, 28.9)	80	10.0 (7.8, 12.2)
75+	228	50.1 (45.2, 55.1)	134	32.0 (27.3, 36.7)	81	17.9 (14.2, 21.6)	305	61.3 (56.6, 66.0)	126	25.8 (21.6, 29.9)	56	13.0 (9.6, 16.4)
**Gender**												
Male	1215	58.0 (55.6, 60.5)	591	28.5 (26.3, 30.7)	277	13.5 (11.8, 15.1)	1442	69.6 (67.3, 71.9)	452	22.8 (20.7, 24.9)	153	7.6 (6.3, 8.9)
Female	1358	64.1 (61.8, 66.3)	550	25.8 (23.7, 27.9)	235	10.1 (8.7, 11.5)	1387	64.2 (61.9, 66.5)	573	27.2 (25.0, 29.3)	165	8.6 (7.2, 10.0)
Other	41	81.4 (68.8, 94.0)	6	8.2 (1.4, 15.1)	5	10.4 (0.0, 21.8)	26	45.2 (30.6, 59.7)	21	40.2 (25.7, 54.7)	6	14.6 (2.4, 26.9)
**Race and ethnicity**												
White, non-Hispanic	1739	58.1 (56.2, 60.0)	860	29.3 (27.5, 31.0)	391	12.7 (11.4, 13.9)	2098	70.2 (68.5, 72.0)	703	22.7 (21.1, 24.3)	219	7.1 (6.1, 8.1)
Black, non-Hispanic	297	69.4 (64.2, 74.5)	85	21.1 (16.6, 25.7)	40	9.5 (6.2, 12.8)	221	53.0 (47.4, 58.6)	135	35.2 (29.8, 40.6)	43	11.8 (8.0, 15.6)
Hispanic, any race	382	67.7 (63.4, 72.0)	120	21.6 (17.9, 25.4)	57	10.7 (7.8, 13.5)	341	64.8 (60.1, 69.4)	133	26.5 (22.2, 30.8)	40	8.7 (5.8, 11.6)
Asian American / Pacific Islander	105	64.3 (55.6, 72.9)	40	25.0 (17.3, 32.7)	16	10.8 (4.5, 17.0)	97	61.2 (52.2, 70.3)	40	29.7 (21.0, 38.3)	12	9.1 (3.7, 14.4)
Other (American Indian/Alaskan, 2+ races, other)	106	59.3 (48.5, 70.0)	51	31.9 (21.4, 42.5)	17	8.8 (3.5, 14.1)	114	60.1 (49.2, 71.0)	40	25.8 (15.9, 35.7)	12	14.1 (6.0, 22.2)
**Education**												
No high school diploma or GED	137	51.3 (44.8, 57.9)	91	34.0 (27.8, 40.2)	40	14.7 (9.9, 19.4)	167	62.3 (55.8, 68.8)	70	26.0 (20.1, 31.9)	30	11.7 (7.3, 16.1)
High school graduate	558	54.8 (51.4, 58.2)	307	27.5 (24.5, 30.5)	206	17.7 (15.2, 20.2)	656	61.7 (58.4, 65.1)	285	27.4 (24.4, 30.5)	117	10.9 (8.8, 13.0)
Some college or Associates degree	677	58.4 (55.3, 61.5)	345	29.4 (26.5, 32.4)	162	12.2 (10.2, 14.1)	773	65.7 (62.6, 68.8)	291	25.2 (22.4, 28.1)	102	9.1 (7.2, 11.0)
Bachelors degree	660	70.6 (67.5, 73.7)	235	23.2 (20.3, 26.0)	66	6.2 (4.6, 7.8)	699	70.7 (67.5, 73.8)	236	24.7 (21.7, 27.7)	46	4.6 (3.3, 6.0)
Masters degree or higher	597	73.7 (70.5, 77.0)	178	21.2 (18.1, 24.2)	47	5.1 (3.6, 6.6)	576	73.7 (70.3, 77.2)	169	21.7 (18.5, 24.9)	31	4.6 (2.8, 6.4)
**Income**												
<$25,000	273	52.2 (47.3, 57.2)	143	31.2 (26.5, 36.0)	86	16.6 (12.9, 20.2)	293	57.4 (52.5, 62.2)	155	31.3 (26.8, 35.9)	55	11.3 (8.1, 14.5)
$25,000.0 - $49,999	424	59.6 (55.6, 63.7)	207	28.2 (24.4, 31.9)	108	12.2 (9.8, 14.6)	438	59.2 (55.0, 63.4)	198	29.0 (25.0, 32.9)	82	11.9 (9.1, 14.6)
$50.0,000.0 - $74,999	416	59.3 (55.1, 63.5)	203	28.4 (24.6, 32.2)	84	12.2 (9.3, 15.2)	469	66.1 (62.0, 70.2)	174	25.9 (22.1, 29.7)	53	8.0 (5.6, 10.4)
$75,000.0 - $99,999	379	64.0 (59.7, 68.2)	140	22.8 (19.1, 26.4)	80	13.2 (10.1, 16.3)	398	65.3 (61.0, 69.6)	162	26.7 (22.7, 30.6)	44	8.0 (5.5, 10.6)
$100.0,000.0 - $149,999	506	65.7 (62.1, 69.4)	197	24.2 (20.9, 27.5)	78	10.1 (7.8, 12.4)	504	69.6 (65.7, 73.5)	164	23.1 (19.5, 26.6)	46	7.3 (5.0, 9.6)
>$150.0,000	631	64.7 (61.4, 68.0)	266	26.6 (23.6, 29.7)	85	8.7 (6.7, 10.6)	769	75.4 (72.4, 78.4)	198	19.7 (16.9, 22.4)	46	5.0 (3.3, 6.6)
**Census Region**												
New England	135	66.0 (58.7, 73.3)	45	20.8 (14.6, 27.0)	28	13.2 (8.0, 18.5)	136	66.7 (59.1, 74.2)	48	26.3 (19.1, 33.6)	16	7.0 (3.5, 10.5)
Mid-Atlantic	316	62.9 (58.2, 67.6)	123	24.3 (20.2, 28.5)	70	12.8 (9.5, 16.0)	376	65.7 (61.3, 70.1)	145	26.6 (22.4, 30.7)	46	7.8 (5.4, 10.1)
East-North Central	400	61.3 (57.2, 65.3)	176	26.9 (23.2, 30.6)	81	11.8 (9.2, 14.4)	398	66.3 (62.1, 70.4)	157	25.5 (21.6, 29.3)	48	8.3 (5.9, 10.7)
West-North Central	186	55.9 (49.7, 62.1)	87	28.0 (22.4, 33.7)	49	16.1 (11.2, 21.0)	182	63.8 (57.6, 70.1)	73	26.9 (21.2, 32.6)	23	9.3 (5.1, 13.5)
South Atlantic	525	62.6 (58.9, 66.2)	219	25.8 (22.5, 29.1)	105	11.6 (9.2, 14.0)	571	65.5 (61.8, 69.1)	209	25.4 (22.0, 28.8)	73	9.1 (6.9, 11.4)
East-South Central	120	50.5 (43.2, 57.8)	83	36.6 (29.5, 43.7)	26	12.9 (7.9, 18.0)	146	60.3 (53.0, 67.6)	62	26.2 (19.9, 32.6)	23	13.5 (7.7, 19.3)
West-South Central	257	57.5 (52.4, 62.6)	155	32.7 (27.9, 37.5)	46	9.8 (6.8, 12.9)	311	69.7 (64.8, 74.6)	101	23.9 (19.3, 28.5)	28	6.4 (3.7, 9.0)
Mountain	219	61.3 (55.7, 66.9)	104	27.5 (22.4, 32.6)	44	11.2 (7.7, 14.7)	261	69.8 (64.5, 75.0)	92	24.4 (19.5, 29.4)	23	5.8 (3.4, 8.2)
Pacific	471	67.1 (63.1, 71.1)	164	23.0 (19.4, 26.6)	72	9.9 (7.5, 12.4)	490	68.0 (63.8, 72.2)	164	24.0 (20.1, 27.8)	46	8.0 (5.4, 10.6)

Table S9, continued

Characteristic	Stop a protest						Support a protest					
	Never justified		Sometimes justified		Usually or always justified		Never justified		Sometimes justified		Usually or always justified	
	Unweighted n	Weighted %, 95% CI	Unweighted n	Weighted %, 95% CI	Unweighted n	Weighted %, 95% CI	Unweighted n	Weighted %, 95% CI	Unweighted n	Weighted %, 95% CI	Unweighted n	Weighted %, 95% CI
**Age, years**												
18-24	144	63.1 (56.3, 69.9)	65	27.6 (21.4, 33.7)	17	9.3 (4.9, 13.8)	149	68.4 (61.8, 75.1)	58	27.0 (20.6, 33.4)	11	4.6 (1.8, 7.4)
25-34	309	62.8 (58.1, 67.6)	153	31.5 (27.0, 36.0)	26	5.6 (3.3, 7.9)	386	72.6 (68.4, 76.8)	110	20.0 (16.4, 23.7)	32	7.3 (4.7, 10.0)
35-44	406	60.6 (56.7, 64.6)	235	35.4 (31.5, 39.3)	28	3.9 (2.4, 5.5)	527	76.1 (72.7, 79.5)	128	18.8 (15.7, 21.9)	33	5.1 (3.4, 6.9)
45-54	325	55.4 (51.1, 59.7)	213	37.8 (33.6, 41.9)	36	6.8 (4.6, 9.0)	516	79.6 (76.2, 83.0)	94	15.9 (12.9, 19.0)	27	4.4 (2.7, 6.1)
55-64	519	56.1 (52.6, 59.5)	354	39.1 (35.7, 42.5)	48	4.8 (3.4, 6.2)	766	84.1 (81.4, 86.8)	112	12.9 (10.4, 15.3)	25	3.0 (1.6, 4.4)
65-74	478	53.5 (50.0, 57.0)	340	39.9 (36.4, 43.3)	56	6.6 (4.8, 8.3)	760	83.2 (80.5, 85.8)	115	13.0 (10.6, 15.4)	33	3.8 (2.5, 5.2)
75+	245	52.6 (47.7, 57.5)	178	39.3 (34.5, 44.1)	33	8.1 (5.3, 11.0)	400	83.9 (80.4, 87.5)	60	12.7 (9.5, 15.8)	14	3.4 (1.6, 5.2)
**Gender**												
Male	1206	59.5 (57.1, 62.0)	741	35.1 (32.8, 37.4)	97	5.4 (4.1, 6.7)	1696	78.7 (76.6, 80.8)	313	16.7 (14.8, 18.6)	78	4.7 (3.5, 5.8)
Female	1177	56.6 (54.2, 59.0)	768	36.8 (34.5, 39.1)	141	6.6 (5.4, 7.8)	1764	79.2 (77.2, 81.1)	338	16.4 (14.7, 18.2)	88	4.4 (3.4, 5.4)
Other	36	72.3 (59.7, 84.8)	18	26.5 (14.1, 38.8)	1	1.3 (0.0, 3.8)	26	45.7 (30.7, 60.7)	20	45.5 (30.0, 61.0)	5	8.8 (0.5, 17.1)
**Race and ethnicity**												
White, non-Hispanic	1640	56.4 (54.4, 58.3)	1141	38.5 (36.6, 40.4)	156	5.2 (4.3, 6.0)	2557	82.1 (80.6, 83.6)	435	15.0 (13.6, 16.4)	84	2.9 (2.3, 3.6)
Black, non-Hispanic	274	63.5 (58.2, 68.8)	121	28.3 (23.5, 33.0)	25	8.2 (4.7, 11.8)	280	67.3 (61.9, 72.7)	84	23.3 (18.3, 28.3)	40	9.4 (6.3, 12.5)
Hispanic, any race	320	61.3 (56.7, 65.9)	170	32.2 (27.8, 36.7)	37	6.4 (4.2, 8.7)	405	71.9 (67.7, 76.2)	106	20.7 (16.9, 24.5)	37	7.3 (4.9, 9.8)
Asian American / Pacific Islander	97	59.2 (50.4, 68.0)	45	30.8 (22.5, 39.1)	15	10.0 (4.4, 15.6)	123	78.8 (71.3, 86.3)	22	16.1 (9.2, 22.9)	8	5.1 (1.3, 8.9)
Other (American Indian/Alaskan, 2+ races, other)	95	51.3 (40.2, 62.4)	61	41.9 (30.7, 53.2)	11	6.7 (1.4, 12.0)	139	76.4 (67.2, 85.5)	30	17.1 (9.9, 24.3)	6	6.5 (0.0, 13.4)
**Education**												
No high school diploma or GED	144	54.0 (47.6, 60.4)	99	34.7 (28.6, 40.7)	31	11.3 (7.1, 15.5)	195	74.8 (68.9, 80.7)	48	17.2 (12.2, 22.2)	20	8.0 (4.1, 11.9)
High school graduate	540	53.3 (49.8, 56.8)	398	38.1 (34.7, 41.5)	91	8.6 (6.6, 10.5)	821	72.5 (69.4, 75.5)	203	20.1 (17.4, 22.9)	79	7.4 (5.7, 9.1)
Some college or Associates degree	644	57.2 (54.0, 60.4)	452	37.9 (34.8, 41.0)	59	4.9 (3.4, 6.3)	962	78.8 (76.1, 81.5)	190	17.5 (15.0, 20.0)	45	3.7 (2.5, 4.8)
Bachelors degree	607	63.0 (59.7, 66.3)	336	33.0 (29.8, 36.2)	41	4.0 (2.7, 5.3)	814	83.5 (80.8, 86.1)	126	14.0 (11.5, 16.5)	20	2.5 (1.3, 3.7)
Masters degree or higher	491	64.4 (60.8, 68.1)	253	32.5 (28.9, 36.1)	22	3.0 (1.7, 4.4)	712	83.6 (80.7, 86.5)	110	14.3 (11.7, 17.0)	11	2.1 (0.7, 3.4)
**Income**												
<$25,000	247	48.0 (43.0, 52.9)	187	38.9 (34.0, 43.8)	58	13.2 (9.6, 16.7)	358	68.5 (63.9, 73.0)	110	21.5 (17.5, 25.6)	48	10.0 (7.0, 12.9)
$25,000.0 - $49,999	403	57.1 (52.9, 61.3)	259	36.1 (32.1, 40.2)	49	6.8 (4.4, 9.1)	572	74.8 (71.1, 78.4)	128	18.1 (14.9, 21.2)	48	7.2 (4.9, 9.5)
$50.0,000.0 - $74,999	395	58.5 (54.3, 62.7)	251	35.5 (31.4, 39.6)	43	6.0 (4.0, 8.1)	583	80.2 (76.7, 83.7)	108	16.2 (13.0, 19.4)	23	3.6 (2.0, 5.3)
$75,000.0 - $99,999	347	59.1 (54.8, 63.4)	234	35.6 (31.4, 39.7)	33	5.4 (3.3, 7.4)	477	76.8 (72.8, 80.9)	91	17.9 (14.2, 21.5)	24	5.3 (3.0, 7.6)
$100.0,000.0 - $149,999	428	61.3 (57.3, 65.4)	250	34.7 (30.7, 38.6)	29	4.0 (2.4, 5.6)	660	81.3 (78.1, 84.5)	114	16.8 (13.6, 19.9)	12	1.9 (0.8, 3.0)
>$150.0,000	606	61.1 (57.8, 64.5)	357	35.2 (32.0, 38.5)	32	3.6 (2.2, 5.0)	854	83.4 (80.6, 86.1)	126	14.2 (11.6, 16.8)	20	2.4 (1.3, 3.6)
**Census Region**												
New England	126	60.5 (52.7, 68.3)	62	31.4 (24.1, 38.8)	15	8.0 (3.4, 12.7)	168	80.3 (74.1, 86.5)	30	16.2 (10.4, 22.0)	7	3.6 (0.8, 6.3)
Mid-Atlantic	295	59.2 (54.5, 63.9)	191	35.1 (30.6, 39.7)	29	5.7 (3.3, 8.0)	447	78.0 (74.1, 81.9)	89	17.4 (13.8, 21.1)	24	4.5 (2.6, 6.5)
East-North Central	342	57.6 (53.3, 61.8)	234	37.2 (33.1, 41.4)	29	5.2 (3.2, 7.1)	536	80.9 (77.6, 84.3)	97	15.5 (12.4, 18.6)	23	3.6 (2.0, 5.1)
West-North Central	152	51.5 (45.0, 58.0)	111	39.0 (32.6, 45.4)	26	9.5 (5.2, 13.8)	248	76.6 (71.2, 82.0)	53	18.7 (13.9, 23.5)	11	4.7 (1.6, 7.8)
South Atlantic	492	57.9 (54.2, 61.6)	317	36.4 (32.8, 39.9)	45	5.8 (3.9, 7.6)	681	77.0 (73.5, 80.5)	132	18.0 (14.9, 21.2)	37	5.0 (3.1, 6.8)
East-South Central	135	53.1 (45.9, 60.3)	96	40.0 (32.9, 47.0)	14	6.9 (2.8, 11.1)	169	77.4 (71.2, 83.6)	35	15.6 (10.2, 20.9)	13	7.0 (3.2, 10.8)
West-South Central	270	60.7 (55.7, 65.7)	165	35.0 (30.1, 39.9)	22	4.2 (2.2, 6.3)	358	77.2 (72.6, 81.9)	72	19.6 (15.1, 24.1)	15	3.2 (1.4, 5.0)
Mountain	218	56.4 (50.7, 62.0)	137	37.0 (31.5, 42.4)	23	6.7 (3.6, 9.7)	293	80.7 (76.2, 85.2)	57	15.4 (11.3, 19.6)	14	3.8 (1.7, 5.9)
Pacific	396	60.6 (56.2, 65.0)	225	32.9 (28.7, 37.2)	41	6.5 (4.3, 8.7)	604	77.8 (74.2, 81.3)	112	16.0 (12.9, 19.1)	31	6.2 (3.9, 8.5)

Table S10. Variation with respondent characteristics in personal willingness to engage in political violence, by type of violence

Characteristic	To damage property								To threaten or intimidate a person							
	Not asked the question		Not willing		Somewhat willing		Very or completely willing		Not asked the question		Not willing		Somewhat willing		Very or completely willing	
	Unweighted n	Weighted %, 95% CI	Unweighted n	Weighted %, 95% CI	Unweighted n	Weighted %, 95% CI	Unweighted n	Weighted %, 95% CI	Unweighted n	Weighted %, 95% CI	Unweighted n	Weighted %, 95% CI	Unweighted n	Weighted %, 95% CI	Unweighted n	Weighted %, 95% CI
**Age, years**																
18-24	273	60.0 (55.0, 64.8)	119	26.9 (22.7, 31.5)	35	8.2 (5.8, 11.5)	20	4.9 (3.1, 7.5)	273	60.2 (55.2, 64.9)	113	24.9 (20.9, 29.4)	46	11.3 (8.4, 15.0)	14	3.6 (2.1, 6.1)
25-34	726	69.5 (66.4, 72.6)	210	21.6 (19.0, 24.5)	41	4.0 (2.9, 5.6)	45	4.8 (3.5, 6.6)	726	69.5 (66.4, 72.6)	218	22.0 (19.4, 24.9)	54	5.6 (4.2, 7.5)	24	2.8 (1.8, 4.3)
35-44	1022	73.8 (71.3, 76.3)	263	19.9 (17.8, 22.3)	43	3.5 (2.5, 4.7)	38	2.8 (2.0, 3.9)	1022	73.7 (71.2, 76.2)	251	19.2 (17.1, 21.6)	63	4.6 (3.6, 6.0)	32	2.4 (1.7, 3.5)
45-54	864	70.2 (67.4, 72.8)	289	24.8 (22.4, 27.5)	38	3.3 (2.3, 4.5)	21	1.7 (1.1, 2.7)	864	70.1 (67.3, 72.8)	291	25.2 (22.7, 27.9)	43	3.4 (2.5, 4.6)	15	1.3 (0.8, 2.1)
55-64	1262	68.5 (66.2, 70.8)	505	28.0 (25.9, 30.3)	43	2.3 (1.7, 3.2)	18	1.1 (0.7, 1.8)	1262	68.5 (66.2, 70.8)	504	28.0 (25.9, 30.3)	44	2.3 (1.7, 3.1)	18	1.1 (0.7, 1.9)
65-74	1147	63.5 (61.1, 65.8)	569	32.5 (30.3, 34.9)	47	2.6 (1.9, 3.4)	22	1.5 (0.9, 2.3)	1147	63.5 (61.1, 65.8)	583	33.3 (31.0, 35.6)	44	2.7 (2.0, 3.8)	10	0.5 (0.3, 1.0)
75+	556	58.0 (54.5, 61.3)	348	38.8 (35.4, 42.2)	20	2.0 (1.3, 3.1)	10	1.3 (0.7, 2.5)	556	58.2 (54.8, 61.6)	347	38.6 (35.2, 42.0)	18	2.1 (1.3, 3.4)	9	1.1 (0.6, 2.2)
**Gender**																
Male	2784	67.0 (65.3, 68.6)	1135	26.7 (25.2, 28.3)	134	3.5 (2.9, 4.3)	92	2.8 (2.2, 3.5)	2784	66.9 (65.3, 68.5)	1125	26.2 (24.7, 27.7)	166	4.7 (4.0, 5.6)	70	2.1 (1.6, 2.8)
Female	2973	68.5 (66.9, 70.0)	1127	26.2 (24.7, 27.7)	119	3.4 (2.8, 4.1)	69	2.0 (1.5, 2.6)	2973	68.5 (66.9, 70.1)	1138	26.3 (24.9, 27.8)	127	3.7 (3.1, 4.5)	47	1.5 (1.1, 2.0)
Other	60	54.7 (44.0, 65.0)	25	21.9 (14.6, 31.5)	9	7.8 (3.8, 15.4)	12	5.6 (8.6, 26.8)	60	54.7 (44.0, 65.0)	27	24.2 (16.4, 34.1)	16	17.8 (10.5, 28.6)	3	3.3 (0.9, 11.0)
**Race and ethnicity**																
White, non-Hispanic	4130	68.2 (66.9, 69.5)	1614	26.2 (25.0, 27.4)	179	3.4 (2.9, 4.0)	106	2.2 (1.8, 2.7)	4130	68.2 (66.9, 69.5)	1615	26.3 (25.1, 27.5)	216	4.2 (3.6, 4.8)	66	1.3 (1.0, 1.7)
Black, non-Hispanic	538	64.4 (60.6, 68.0)	226	26.7 (23.4, 30.2)	26	3.3 (2.1, 5.1)	37	5.6 (3.9, 8.0)	538	64.5 (60.7, 68.1)	230	26.8 (23.5, 30.3)	31	4.4 (2.9, 6.6)	28	4.3 (2.9, 6.5)
Hispanic, any race	726	66.8 (63.7, 69.9)	295	26.8 (24.0, 29.8)	36	3.7 (2.6, 5.2)	25	2.7 (1.8, 4.1)	726	66.9 (63.7, 69.9)	290	25.5 (22.8, 28.4)	43	5.3 (3.9, 7.3)	22	2.3 (1.5, 3.6)
Asian American / Pacific Islander	225	70.4 (64.2, 75.9)	76	24.7 (19.7, 30.6)	11	4.6 (2.3, 9.0)	1	0.3 (0.0, 1.8)	225	70.4 (64.2, 75.9)	79	26.1 (20.9, 32.1)	5	1.8 (0.6, 5.5)	4	1.7 (0.6, 4.5)
Other (American Indian/Alaskan, 2+ races, other)	231	61.8 (54.1, 69.0)	92	29.9 (23.5, 37.3)	15	5.8 (2.8, 11.6)	5	2.4 (0.9, 6.4)	231	61.8 (54.1, 69.0)	93	27.8 (21.8, 34.7)	17	9.5 (5.2, 16.8)	2	0.8 (0.2, 4.5)
**Education**																
No high school diploma or GED	313	58.4 (53.8, 62.9)	174	30.5 (26.5, 34.9)	29	5.7 (3.8, 8.4)	23	5.4 (3.5, 8.2)	313	58.5 (53.8, 63.0)	182	31.3 (27.2, 35.7)	25	6.2 (4.1, 9.3)	18	4.0 (2.5, 6.5)
High school graduate	1238	58.4 (56.0, 60.7)	754	33.3 (31.2, 35.6)	94	4.8 (3.8, 6.0)	60	3.5 (2.6, 4.6)	1238	58.5 (56.1, 60.8)	743	32.3 (30.2, 34.5)	123	6.8 (5.6, 8.3)	40	2.4 (1.7, 3.4)
Some college or Associates degree	1549	66.7 (64.5, 68.8)	696	28.2 (26.2, 30.2)	71	3.3 (2.6, 4.3)	41	1.8 (1.3, 2.5)	1549	66.7 (64.5, 68.7)	688	27.9 (26.0, 30.0)	86	3.9 (3.1, 4.9)	34	1.5 (1.0, 2.1)
Bachelors degree	1469	75.7 (73.5, 77.7)	398	19.5 (17.7, 21.5)	47	2.7 (2.0, 3.6)	35	2.1 (1.5, 3.0)	1469	75.6 (73.5, 77.6)	414	20.5 (18.6, 22.5)	46	2.7 (2.0, 3.6)	21	1.2 (0.8, 1.9)
Masters degree or higher	1281	80.4 (78.2, 82.4)	281	16.7 (14.9, 18.8)	26	1.8 (1.2, 2.7)	15	1.1 (0.7, 1.9)	1281	80.4 (78.2, 82.4)	280	16.5 (14.6, 18.5)	32	2.3 (1.6, 3.3)	9	0.9 (0.4, 1.8)
**Income**																
<$25,000	552	53.4 (49.9, 56.8)	360	35.0 (31.8, 38.4)	50	5.7 (4.2, 7.7)	50	5.9 (4.4, 8.0)	552	53.5 (50.0, 57.0)	361	34.7 (31.5, 38.1)	66	7.9 (6.1, 10.2)	30	3.9 (2.6, 5.7)
$25,000 - $49,999	885	60.4 (57.5, 63.3)	493	32.7 (30.0, 35.5)	57	4.4 (3.2, 5.9)	30	2.4 (1.6, 3.6)	885	60.4 (57.5, 63.3)	489	32.2 (29.6, 35.0)	71	5.7 (4.4, 7.5)	20	1.6 (1.0, 2.7)
$50,000 - $74,999	967	67.5 (64.6, 70.3)	372	26.2 (23.7, 28.9)	45	4.0 (2.8, 5.5)	25	2.3 (1.4, 3.5)	967	67.6 (64.7, 70.4)	372	26.1 (23.6, 28.8)	46	4.3 (3.1, 6.0)	23	2.0 (1.3, 3.3)
$75,000 - $99,999	824	67.8 (64.8, 70.7)	323	25.9 (23.2, 28.7)	38	3.5 (2.4, 4.9)	26	2.8 (1.9, 4.3)	824	67.8 (64.8, 70.7)	322	25.7 (23.1, 28.5)	46	4.3 (3.1, 5.9)	19	2.1 (1.3, 3.5)
$100,000 - $149,999	1083	72.2 (69.6, 74.7)	355	23.2 (20.9, 25.6)	37	2.8 (1.9, 4.0)	21	1.8 (1.1, 2.8)	1083	72.1 (69.5, 74.6)	354	23.1 (20.8, 25.5)	41	3.3 (2.3, 4.6)	19	1.5 (0.9, 2.4)
>$150,000	1539	76.6 (74.4, 78.6)	400	19.7 (17.9, 21.7)	40	2.2 (1.6, 3.1)	22	1.5 (0.9, 2.4)	1539	76.6 (74.4, 78.6)	409	20.0 (18.1, 21.9)	42	2.6 (1.8, 3.7)	11	0.9 (0.5, 1.6)
**Census Region**																
New England	285	70.2 (65.0, 74.9)	110	25.8 (21.3, 30.8)	8	1.8 (0.8, 3.7)	8	2.3 (1.1, 4.6)	285	70.2 (65.0, 74.9)	108	24.7 (20.4, 29.6)	13	3.9 (2.1, 6.9)	5	1.3 (0.5, 3.2)
Mid-Atlantic	735	68.1 (64.9, 71.2)	299	26.7 (23.9, 29.8)	35	3.6 (2.5, 5.1)	16	1.6 (0.9, 2.7)	735	68.4 (65.2, 71.4)	284	25.4 (22.6, 28.4)	47	4.5 (3.2, 6.1)	16	1.7 (1.0, 3.0)
East-North Central	861	68.8 (66.0, 71.5)	330	25.1 (22.6, 27.7)	38	3.1 (2.2, 4.3)	31	3.0 (2.0, 4.3)	861	68.8 (66.0, 71.5)	331	25.3 (22.8, 27.9)	37	3.2 (2.2, 4.5)	31	2.8 (1.9, 4.0)
West-North Central	389	62.3 (57.8, 66.6)	179	29.6 (25.7, 33.7)	22	5.7 (3.4, 9.4)	13	2.4 (1.4, 4.1)	389	62.2 (57.7, 66.5)	188	31.7 (27.7, 36.0)	20	4.7 (2.8, 7.8)	7	1.4 (0.6, 2.9)
South Atlantic	1147	66.6 (64.0, 69.1)	482	27.8 (25.5, 30.3)	48	3.2 (2.3, 4.5)	31	2.4 (1.6, 3.6)	1147	66.6 (64.0, 69.1)	473	27.1 (24.8, 29.5)	66	4.7 (3.6, 6.2)	19	1.5 (0.9, 2.5)
East-South Central	284	57.9 (52.7, 62.9)	139	31.3 (26.6, 36.3)	20	4.8 (3.0, 7.5)	21	6.1 (3.8, 9.7)	284	57.9 (52.7, 63.0)	147	31.8 (27.2, 36.7)	21	6.3 (3.9, 10.0)	12	4.0 (2.1, 7.5)
West-South Central	583	64.7 (61.1, 68.1)	270	28.5 (25.3, 31.9)	22	3.1 (1.9, 4.8)	25	3.8 (2.4, 5.8)	583	64.6 (61.0, 68.0)	276	29.3 (26.1, 32.7)	33	4.5 (3.1, 6.5)	10	1.7 (0.8, 3.3)
Mountain	524	69.9 (66.0, 73.4)	188	25.5 (22.1, 29.1)	25	3.7 (2.4, 5.7)	8	1.0 (0.5, 2.0)	524	69.9 (66.0, 73.4)	189	24.6 (21.4, 28.2)	29	5.2 (3.5, 7.7)	3	0.3 (0.1, 0.9)
Pacific	1042	72.3 (69.5, 75.0)	306	21.5 (19.1, 24.1)	49	4.1 (3.0, 5.6)	21	2.0 (1.3, 3.2)	1042	72.3 (69.5, 75.0)	311	21.6 (19.2, 24.1)	46	4.2 (3.0, 5.8)	19	1.9 (1.2, 3.2)

Table S10, continued

Characteristic	To injure a person								To kill a person							
	Not asked the question		Not willing		Somewhat willing		Very or completely willing		Not asked the question		Not willing		Somewhat willing		Very or completely willing	
	Unweighted n	Weighted %, 95% CI	Unweighted n	Weighted %, 95% CI	Unweighted n	Weighted %, 95% CI	Unweighted n	Weighted %, 95% CI	Unweighted n	Weighted %, 95% CI	Unweighted n	Weighted %, 95% CI	Unweighted n	Weighted %, 95% CI	Unweighted n	Weighted %, 95% CI
**Age, years**																
18-24	273	60.0 (55.0, 64.8)	130	29.0 (24.7, 33.7)	29	7.3 (5.0, 10.5)	15	3.7 (2.2, 6.2)	273	60.3 (55.3, 65.0)	149	34.5 (29.9, 39.4)	11	2.7 (1.4, 5.0)	12	2.6 (1.4, 4.6)
25-34	726	69.5 (66.4, 72.6)	225	22.8 (20.1, 25.8)	53	5.4 (4.0, 7.2)	18	2.2 (1.3, 3.6)	726	69.5 (66.4, 72.6)	248	25.2 (22.4, 28.2)	28	3.1 (2.1, 4.6)	20	2.2 (1.4, 3.5)
35-44	1022	73.8 (71.2, 76.2)	258	19.8 (17.6, 22.2)	50	3.5 (2.6, 4.7)	37	2.9 (2.1, 4.0)	1022	73.7 (71.1, 76.1)	279	21.4 (19.1, 23.8)	32	2.2 (1.5, 3.1)	36	2.7 (2.0, 3.9)
45-54	864	70.2 (67.4, 72.8)	300	26.0 (23.5, 28.7)	32	2.5 (1.8, 3.6)	16	1.3 (0.8, 2.1)	864	70.2 (67.4, 72.8)	314	27.4 (24.8, 30.1)	18	1.2 (0.7, 1.9)	16	1.3 (0.8, 2.1)
55-64	1262	68.7 (66.3, 70.9)	514	28.6 (26.4, 30.8)	31	1.6 (1.1, 2.3)	17	1.2 (0.7, 2.0)	1262	68.6 (66.2, 70.8)	530	29.4 (27.2, 31.7)	12	0.5 (0.3, 0.9)	22	1.5 (0.9, 2.3)
65-74	1147	63.6 (61.2, 65.9)	594	34.2 (31.9, 36.6)	29	1.6 (1.1, 2.4)	11	0.6 (0.3, 1.1)	1147	63.5 (61.1, 65.8)	600	34.4 (32.1, 36.8)	20	1.1 (0.7, 1.8)	17	1.0 (0.6, 1.6)
75+	556	58.1 (54.6, 61.5)	352	39.5 (36.2, 43.0)	17	1.6 (1.0, 2.6)	7	0.8 (0.4, 1.7)	556	58.0 (54.6, 61.4)	357	40.0 (36.7, 43.5)	12	1.1 (0.6, 2.0)	8	0.8 (0.4, 1.6)
**Gender**																
Male	2784	67.0 (65.4, 68.6)	1150	27.1 (25.6, 28.6)	133	3.6 (3.0, 4.4)	73	2.3 (1.8, 2.9)	2784	67.0 (65.3, 68.6)	1205	28.8 (27.3, 30.4)	76	1.9 (1.5, 2.5)	80	2.3 (1.8, 2.9)
Female	2973	68.5 (66.9, 70.0)	1173	27.4 (25.9, 28.9)	95	2.8 (2.2, 3.5)	44	1.4 (1.0, 1.9)	2973	68.5 (66.9, 70.1)	1213	28.8 (27.3, 30.4)	52	1.3 (1.0, 1.8)	47	1.3 (0.9, 1.8)
Other	60	54.7 (44.0, 65.0)	33	31.9 (22.9, 42.4)	10	9.9 (4.5, 20.2)	3	3.6 (1.1, 11.2)	60	54.7 (44.0, 65.0)	39	36.1 (26.7, 46.6)	5	7.9 (2.9, 19.7)	2	1.4 (0.3, 5.4)
**Race and ethnicity**																
White, non-Hispanic	4130	68.3 (67.0, 69.5)	1669	27.4 (26.2, 28.6)	163	3.2 (2.7, 3.7)	62	1.2 (0.9, 1.6)	4130	68.2 (66.9, 69.5)	1742	28.8 (27.6, 30.1)	86	1.7 (1.3, 2.1)	70	1.3 (1.0, 1.7)
Black, non-Hispanic	538	64.5 (60.7, 68.1)	234	27.3 (24.0, 30.9)	28	4.3 (2.8, 6.5)	25	3.9 (2.6, 6.0)	538	64.5 (60.7, 68.2)	241	28.9 (25.5, 32.5)	19	3.0 (1.7, 5.0)	28	3.6 (2.4, 5.4)
Hispanic, any race	726	66.9 (63.7, 69.9)	302	27.1 (24.3, 30.1)	24	2.7 (1.8, 4.2)	28	3.2 (2.2, 4.7)	726	66.9 (63.7, 69.9)	320	29.5 (26.6, 32.6)	12	1.0 (0.6, 1.9)	23	2.6 (1.7, 3.9)
Asian American / Pacific Islander	225	70.4 (64.2, 75.9)	81	27.6 (22.2, 33.8)	6	1.7 (0.7, 3.9)	1	0.3 (0.0, 2.2)	225	70.4 (64.2, 75.9)	80	27.4 (22.0, 33.6)	5	1.5 (0.6, 3.7)	3	0.7 (0.2, 2.2)
Other (American Indian/Alaskan, 2+ races, other)	231	61.8 (54.1, 69.0)	87	27.4 (21.3, 34.3)	20	9.0 (4.9, 16.1)	5	1.8 (0.6, 5.3)	231	61.8 (54.1, 69.0)	94	33.6 (26.7, 41.3)	11	2.0 (1.0, 4.1)	7	2.6 (1.0, 6.3)
**Education**																
No high school diploma or GED	313	58.5 (53.8, 63.0)	189	33.2 (29.0, 37.6)	18	4.4 (2.7, 7.3)	18	3.9 (2.4, 6.3)	313	58.4 (53.8, 62.9)	201	36.3 (32.0, 40.9)	11	2.3 (1.2, 4.2)	14	3.0 (1.7, 5.3)
High school graduate	1238	58.4 (56.0, 60.7)	762	33.7 (31.5, 35.9)	100	5.2 (4.2, 6.5)	44	2.7 (2.0, 3.7)	1238	58.5 (56.1, 60.8)	817	36.8 (34.6, 39.1)	44	2.4 (1.7, 3.3)	45	2.3 (1.7, 3.2)
Some college or Associates degree	1549	66.8 (64.6, 68.9)	710	29.0 (27.0, 31.0)	64	3.0 (2.3, 3.9)	30	1.3 (0.9, 1.9)	1549	66.7 (64.6, 68.8)	728	30.0 (28.0, 32.1)	39	1.6 (1.1, 2.2)	41	1.7 (1.2, 2.4)
Bachelors degree	1469	75.7 (73.5, 77.7)	420	21.0 (19.1, 23.0)	42	2.2 (1.6, 3.0)	18	1.1 (0.7, 1.9)	1469	75.7 (73.5, 77.7)	434	21.8 (19.9, 23.9)	24	1.2 (0.7, 1.8)	22	1.4 (0.9, 2.2)
Masters degree or higher	1281	80.5 (78.3, 82.5)	292	17.4 (15.5, 19.5)	17	1.3 (0.7, 2.1)	11	0.8 (0.4, 1.5)	1281	80.4 (78.2, 82.4)	297	17.9 (15.9, 20.0)	15	1.1 (0.6, 1.9)	9	0.6 (0.3, 1.3)
**Income**																
<$25,000	552	53.5 (50.0, 56.9)	364	36.2 (32.9, 39.7)	59	6.2 (4.6, 8.2)	34	4.1 (2.9, 5.9)	552	53.4 (50.0, 56.9)	401	40.7 (37.3, 44.2)	24	2.4 (1.6, 3.7)	35	3.5 (2.4, 4.9)
$25,000 - $49,999	885	60.5 (57.6, 63.3)	506	33.6 (30.9, 36.4)	52	4.4 (3.2, 6.1)	20	1.5 (0.9, 2.5)	885	60.4 (57.5, 63.3)	528	36.0 (33.2, 38.9)	30	1.8 (1.2, 2.7)	22	1.7 (1.0, 2.8)
$50,000 - $74,999	967	67.5 (64.6, 70.3)	394	28.1 (25.5, 30.9)	31	2.8 (1.9, 4.1)	17	1.6 (0.9, 2.8)	967	67.5 (64.6, 70.3)	407	29.7 (27.0, 32.5)	19	1.5 (0.9, 2.6)	16	1.2 (0.7, 2.2)
$75,000 - $99,999	824	67.9 (64.9, 70.8)	332	26.9 (24.2, 29.8)	37	3.1 (2.2, 4.4)	17	2.1 (1.3, 3.5)	824	67.9 (64.9, 70.8)	341	27.6 (24.9, 30.5)	21	1.9 (1.2, 3.1)	24	2.6 (1.7, 3.9)
$100,000 - $149,999	1083	72.3 (69.7, 74.8)	365	23.7 (21.4, 26.2)	29	2.5 (1.7, 3.8)	17	1.5 (0.9, 2.4)	1083	72.3 (69.6, 74.7)	377	24.7 (22.3, 27.2)	17	1.4 (0.8, 2.5)	18	1.6 (1.0, 2.7)
>$150,000	1539	76.6 (74.4, 78.6)	412	20.3 (18.4, 22.3)	33	2.1 (1.4, 3.1)	16	1.1 (0.6, 1.9)	1539	76.6 (74.5, 78.6)	423	21.0 (19.1, 23.0)	22	1.5 (0.9, 2.4)	16	1.0 (0.6, 1.7)
**Census Region**																
New England	285	70.4 (65.2, 75.1)	114	27.3 (22.7, 32.4)	8	1.6 (0.7, 3.4)	3	0.8 (0.2, 2.7)	285	70.2 (65.0, 74.9)	119	28.1 (23.5, 33.3)	3	0.6 (0.2, 1.9)	4	1.1 (0.4, 3.1)
Mid-Atlantic	735	68.2 (65.1, 71.3)	303	27.1 (24.2, 30.1)	30	2.8 (1.9, 4.1)	15	1.9 (1.0, 3.3)	735	68.3 (65.1, 71.3)	320	28.7 (25.8, 31.8)	16	1.8 (1.1, 3.0)	13	1.2 (0.7, 2.1)
East-North Central	861	68.9 (66.0, 71.6)	338	26.1 (23.5, 28.8)	33	2.8 (2.0, 4.0)	27	2.2 (1.5, 3.4)	861	68.8 (66.0, 71.5)	354	27.6 (25.0, 30.4)	16	1.2 (0.7, 2.0)	29	2.4 (1.6, 3.5)
West-North Central	389	62.4 (57.9, 66.7)	182	31.4 (27.3, 35.8)	25	4.9 (3.2, 7.4)	6	1.3 (0.6, 3.0)	389	62.3 (57.9, 66.6)	196	34.1 (30.0, 38.6)	10	2.0 (1.0, 4.2)	8	1.5 (0.7, 3.1)
South Atlantic	1147	66.6 (64.0, 69.1)	494	28.4 (26.0, 30.8)	43	3.3 (2.3, 4.6)	22	1.8 (1.1, 2.7)	1147	66.6 (64.0, 69.1)	513	29.8 (27.4, 32.3)	26	2.1 (1.3, 3.2)	21	1.5 (1.0, 2.4)
East-South Central	284	58.0 (52.8, 63.0)	155	34.3 (29.6, 39.3)	15	4.4 (2.4, 8.0)	10	3.2 (1.6, 6.5)	284	58.1 (52.9, 63.1)	162	37.3 (32.4, 42.5)	6	1.0 (0.4, 2.3)	11	3.6 (1.9, 7.0)
West-South Central	583	64.7 (61.1, 68.2)	277	30.4 (27.1, 33.9)	29	3.6 (2.4, 5.4)	10	1.3 (0.7, 2.5)	583	64.7 (61.1, 68.1)	287	31.9 (28.5, 35.4)	14	1.4 (0.7, 2.6)	16	2.1 (1.2, 3.5)
Mountain	524	69.9 (66.0, 73.4)	196	25.8 (22.5, 29.4)	21	3.8 (2.3, 6.1)	4	0.6 (0.2, 1.8)	524	69.9 (66.0, 73.4)	198	27.3 (23.8, 31.1)	16	2.3 (1.3, 3.9)	7	0.6 (0.3, 1.3)
Pacific	1042	72.4 (69.6, 75.0)	314	21.8 (19.5, 24.4)	37	3.3 (2.3, 4.8)	24	2.5 (1.6, 3.9)	1042	72.3 (69.5, 75.0)	328	23.5 (21.0, 26.2)	26	2.1 (1.4, 3.2)	22	2.1 (1.3, 3.3)

Table S11. Variation with respondent characteristics in personal willingness to engage in political violence, by target of violence

Characteristic	An elected federal or state government official								An elected local government official							
	Not asked the question		Not willing		Somewhat willing		Very or completely willing		Not asked the question		Not willing		Somewhat willing		Very or completely willing	
	Unweighted n	Weighted %, 95% CI	Unweighted n	Weighted %, 95% CI	Unweighted n	Weighted %, 95% CI	Unweighted n	Weighted %, 95% CI	Unweighted n	Weighted %, 95% CI	Unweighted n	Weighted %, 95% CI	Unweighted n	Weighted %, 95% CI	Unweighted n	Weighted %, 95% CI
**Age, years**																
18-24	273	60.2 (55.2, 65.0)	137	30.8 (26.5, 35.6)	19	4.2 (2.6, 6.9)	17	4.7 (2.9, 7.6)	273	60.5 (55.6, 65.3)	140	32.1 (27.6, 36.9)	20	4.3 (2.7, 6.8)	11	3.1 (1.7, 5.6)
25-34	726	69.7 (66.5, 72.7)	221	22.7 (20.0, 25.6)	46	4.6 (3.4, 6.3)	27	3.0 (2.0, 4.6)	726	69.7 (66.6, 72.8)	227	23.3 (20.6, 26.3)	41	4.1 (3.0, 5.7)	25	2.8 (1.8, 4.3)
35-44	1022	74.0 (71.4, 76.4)	261	20.1 (17.9, 22.5)	44	3.4 (2.5, 4.6)	36	2.5 (1.8, 3.5)	1022	73.8 (71.2, 76.2)	267	20.5 (18.3, 22.9)	43	3.3 (2.4, 4.4)	34	2.5 (1.7, 3.5)
45-54	864	70.3 (67.5, 72.9)	300	25.9 (23.3, 28.6)	34	2.8 (2.0, 3.9)	13	1.1 (0.6, 1.9)	864	70.2 (67.4, 72.8)	306	26.3 (23.8, 29.1)	31	2.5 (1.8, 3.7)	11	0.9 (0.5, 1.7)
55-64	1262	68.8 (66.4, 71.0)	511	28.7 (26.5, 31.1)	35	1.8 (1.3, 2.6)	12	0.7 (0.3, 1.2)	1262	68.8 (66.4, 71.0)	511	28.7 (26.5, 31.0)	35	2.0 (1.4, 2.8)	12	0.6 (0.3, 1.1)
65-74	1147	63.7 (61.3, 66.0)	579	32.8 (30.6, 35.2)	41	2.7 (1.9, 3.6)	13	0.9 (0.5, 1.6)	1147	63.8 (61.4, 66.2)	579	32.9 (30.7, 35.3)	36	2.3 (1.6, 3.3)	13	0.9 (0.5, 1.6)
75+	556	58.0 (54.6, 61.4)	345	38.2 (34.9, 41.6)	26	3.0 (2.0, 4.6)	6	0.7 (0.3, 1.7)	556	58.0 (54.6, 61.4)	351	38.8 (35.5, 42.2)	20	2.5 (1.5, 4.0)	6	0.7 (0.3, 1.7)
**Gender**																
Male	2784	67.2 (65.5, 68.8)	1163	27.7 (26.2, 29.2)	121	3.1 (2.5, 3.7)	64	2.1 (1.6, 2.7)	2784	67.1 (65.5, 68.7)	1167	27.6 (26.1, 29.2)	125	3.4 (2.8, 4.1)	60	1.9 (1.4, 2.5)
Female	2973	68.6 (67.0, 70.1)	1148	26.9 (25.4, 28.4)	108	3.0 (2.4, 3.7)	52	1.6 (1.1, 2.1)	2973	68.7 (67.1, 70.2)	1165	27.5 (26.0, 29.0)	89	2.5 (2.0, 3.1)	48	1.4 (1.0, 1.9)
Other	60	54.7 (44.0, 65.0)	26	24.0 (16.2, 34.2)	13	12.5 (7.1, 21.1)	7	8.8 (3.6, 19.6)	60	55.0 (44.2, 65.3)	30	30.9 (21.7, 42.0)	11	8.8 (4.8, 15.7)	4	5.2 (1.8, 14.4)
**Race and ethnicity**																
White, non-Hispanic	4130	68.3 (67.0, 69.6)	1662	27.4 (26.2, 28.7)	167	3.1 (2.6, 3.6)	59	1.1 (0.9, 1.5)	4130	68.3 (67.0, 69.6)	1693	28.0 (26.8, 29.2)	142	2.6 (2.2, 3.1)	54	1.1 (0.8, 1.5)
Black, non-Hispanic	538	64.6 (60.8, 68.3)	233	27.5 (24.1, 31.0)	24	3.0 (1.9, 4.7)	28	4.9 (3.2, 7.3)	538	64.7 (60.9, 68.4)	231	28.4 (25.0, 32.0)	27	3.3 (2.1, 5.0)	26	3.6 (2.4, 5.6)
Hispanic, any race	726	67.0 (63.8, 70.1)	296	27.3 (24.5, 30.4)	33	3.1 (2.1, 4.4)	23	2.6 (1.6, 4.0)	726	67.3 (64.1, 70.3)	291	26.7 (23.9, 29.7)	36	3.9 (2.8, 5.5)	21	2.1 (1.3, 3.4)
Asian American / Pacific Islander	225	70.9 (64.7, 76.4)	74	24.0 (19.0, 29.8)	8	3.5 (1.5, 8.0)	5	1.5 (0.6, 3.8)	225	70.9 (64.7, 76.4)	76	24.8 (19.7, 30.8)	5	2.0 (0.8, 4.8)	6	2.3 (0.9, 5.9)
Other (American Indian/Alaskan, 2+ races, other)	231	62.3 (54.6, 69.4)	89	28.7 (22.3, 36.0)	13	5.7 (2.7, 11.8)	9	3.3 (1.6, 6.8)	231	61.9 (54.2, 69.0)	90	29.5 (23.0, 36.9)	16	6.4 (3.3, 12.1)	5	2.2 (0.8, 5.8)
**Education**																
No high school diploma or GED	313	58.5 (53.9, 63.0)	178	31.4 (27.3, 35.8)	25	5.2 (3.3, 7.9)	22	4.9 (3.1, 7.7)	313	58.4 (53.8, 62.9)	174	31.2 (27.0, 35.6)	34	6.5 (4.5, 9.3)	17	3.9 (2.3, 6.4)
High school graduate	1238	58.7 (56.3, 61.0)	776	35.0 (32.8, 37.3)	81	3.8 (3.0, 4.8)	39	2.5 (1.7, 3.6)	1238	58.8 (56.4, 61.1)	784	35.6 (33.3, 37.9)	75	3.8 (2.9, 4.8)	36	1.9 (1.3, 2.7)
Some college or Associates degree	1549	66.8 (64.6, 68.8)	701	28.5 (26.5, 30.6)	75	3.6 (2.8, 4.6)	30	1.2 (0.8, 1.7)	1549	66.8 (64.7, 68.9)	712	29.1 (27.1, 31.1)	62	2.7 (2.1, 3.6)	27	1.4 (0.9, 2.2)
Bachelors degree	1469	75.8 (73.6, 77.8)	415	20.7 (18.8, 22.7)	41	2.3 (1.6, 3.2)	20	1.3 (0.8, 2.0)	1469	75.7 (73.6, 77.7)	423	21.1 (19.2, 23.1)	31	1.6 (1.1, 2.4)	23	1.5 (1.0, 2.4)
Masters degree or higher	1281	80.5 (78.3, 82.5)	284	17.0 (15.1, 19.1)	23	1.4 (0.9, 2.2)	13	1.1 (0.6, 2.0)	1281	80.4 (78.2, 82.4)	288	17.3 (15.4, 19.4)	24	1.5 (1.0, 2.4)	9	0.7 (0.3, 1.5)
**Income**																
<$25,000	552	53.5 (50.0, 56.9)	370	36.5 (33.2, 39.9)	51	5.7 (4.3, 7.5)	37	4.3 (3.0, 6.2)	552	53.7 (50.2, 57.2)	366	36.5 (33.2, 40.0)	58	6.4 (4.9, 8.4)	30	3.3 (2.2, 4.9)
$25,000 - $49,999	885	60.5 (57.6, 63.4)	492	33.0 (30.3, 35.9)	64	4.7 (3.6, 6.2)	22	1.8 (1.1, 2.9)	885	60.5 (57.6, 63.3)	501	33.5 (30.8, 36.4)	56	4.4 (3.3, 5.8)	21	1.6 (0.9, 2.6)
$50,000 - $74,999	967	67.7 (64.8, 70.5)	383	27.4 (24.8, 30.1)	41	3.4 (2.3, 4.8)	14	1.6 (0.8, 3.0)	967	67.7 (64.8, 70.5)	394	28.5 (25.9, 31.3)	33	2.8 (1.9, 4.1)	10	1.0 (0.5, 2.1)
$75,000 - $99,999	824	68.3 (65.3, 71.1)	335	27.5 (24.8, 30.4)	30	2.7 (1.8, 4.0)	16	1.6 (0.9, 2.6)	824	68.3 (65.3, 71.2)	335	27.3 (24.6, 30.2)	29	2.5 (1.7, 3.8)	17	1.8 (1.1, 3.0)
$100,000 - $149,999	1083	72.3 (69.7, 74.8)	368	24.2 (21.9, 26.7)	26	1.9 (1.3, 2.9)	16	1.6 (0.9, 2.6)	1083	72.4 (69.8, 74.8)	370	24.3 (22.0, 26.8)	23	1.8 (1.1, 2.8)	16	1.5 (0.9, 2.5)
>$150,000	1539	76.7 (74.6, 78.7)	406	20.0 (18.2, 22.0)	33	1.8 (1.2, 2.8)	19	1.4 (0.8, 2.3)	1539	76.6 (74.5, 78.6)	415	20.7 (18.8, 22.7)	27	1.4 (0.9, 2.1)	18	1.3 (0.8, 2.3)
**Census Region**																
New England	285	70.9 (65.8, 75.5)	108	25.2 (20.8, 30.1)	11	2.5 (1.3, 4.5)	5	1.5 (0.6, 3.6)	285	70.8 (65.7, 75.4)	110	25.3 (20.9, 30.1)	11	2.4 (1.3, 4.4)	4	1.6 (0.5, 4.5)
Mid-Atlantic	735	68.3 (65.1, 71.3)	304	26.9 (24.0, 29.9)	31	3.5 (2.3, 5.3)	12	1.3 (0.7, 2.5)	735	68.5 (65.4, 71.6)	304	26.9 (24.1, 29.9)	27	3.2 (2.1, 4.7)	13	1.4 (0.8, 2.6)
East-North Central	861	69.0 (66.2, 71.7)	346	26.9 (24.3, 29.7)	30	2.4 (1.6, 3.4)	19	1.7 (1.0, 2.7)	861	68.9 (66.1, 71.6)	351	27.4 (24.8, 30.2)	29	2.2 (1.5, 3.2)	17	1.4 (0.9, 2.4)
West-North Central	389	62.3 (57.8, 66.6)	184	31.9 (27.8, 36.3)	22	4.7 (3.0, 7.4)	7	1.1 (0.5, 2.4)	389	62.3 (57.8, 66.6)	188	32.4 (28.3, 36.8)	19	4.2 (2.6, 6.8)	7	1.1 (0.5, 2.4)
South Atlantic	1147	66.8 (64.2, 69.3)	492	28.3 (26.0, 30.8)	43	3.1 (2.2, 4.3)	20	1.8 (1.0, 2.9)	1147	66.8 (64.2, 69.3)	502	29.5 (27.1, 32.0)	36	2.5 (1.7, 3.6)	16	1.2 (0.7, 2.0)
East-South Central	284	57.9 (52.7, 62.9)	150	34.4 (29.7, 39.6)	22	5.3 (3.4, 8.1)	8	2.4 (1.0, 5.5)	284	58.0 (52.8, 63.0)	152	35.3 (30.5, 40.5)	22	4.7 (3.0, 7.3)	5	2.0 (0.7, 5.3)
West-South Central	583	64.6 (61.0, 68.1)	279	29.9 (26.7, 33.3)	24	3.2 (2.0, 5.0)	14	2.3 (1.3, 4.1)	583	64.9 (61.3, 68.4)	273	29.4 (26.2, 32.9)	25	3.0 (1.9, 4.6)	15	2.6 (1.5, 4.6)
Mountain	524	70.2 (66.3, 73.7)	189	25.8 (22.4, 29.5)	21	2.8 (1.7, 4.4)	9	1.2 (0.6, 2.5)	524	69.9 (66.0, 73.4)	194	26.1 (22.7, 29.7)	18	2.8 (1.7, 4.7)	9	1.3 (0.6, 2.6)
Pacific	1042	72.5 (69.7, 75.1)	302	21.6 (19.2, 24.2)	41	2.9 (2.1, 4.0)	30	3.1 (2.0, 4.6)	1042	72.5 (69.7, 75.2)	307	21.9 (19.5, 24.5)	39	3.2 (2.3, 4.6)	26	2.3 (1.5, 3.5)

Table S11, continued

Characteristic	An election worker, such as a poll worker or vote counter								A public health official							
	Not asked the question		Not willing		Somewhat willing		Very or completely willing		Not asked the question		Not willing		Somewhat willing		Very or completely willing	
	Unweighted n	Weighted %, 95% CI	Unweighted n	Weighted %, 95% CI	Unweighted n	Weighted %, 95% CI	Unweighted n	Weighted %, 95% CI	Unweighted n	Weighted %, 95% CI	Unweighted n	Weighted %, 95% CI	Unweighted n	Weighted %, 95% CI	Unweighted n	Weighted %, 95% CI
**Age, years**																
18-24	273	60.4 (55.4, 65.1)	149	33.2 (28.7, 38.0)	10	3.0 (1.6, 5.8)	13	3.4 (2.0, 5.9)	273	60.4 (55.4, 65.2)	150	34.0 (29.4, 38.8)	13	3.3 (1.9, 5.7)	9	2.3 (1.2, 4.7)
25-34	726	69.6 (66.4, 72.6)	257	26.0 (23.1, 29.0)	15	1.7 (1.0, 3.0)	23	2.8 (1.8, 4.2)	726	69.6 (66.4, 72.6)	248	25.1 (22.3, 28.1)	26	2.7 (1.8, 4.2)	21	2.6 (1.6, 4.2)
35-44	1022	73.7 (71.2, 76.2)	290	22.0 (19.7, 24.4)	35	2.7 (1.9, 3.8)	21	1.6 (1.0, 2.5)	1022	73.9 (71.3, 76.3)	270	20.6 (18.4, 23.0)	42	3.1 (2.3, 4.2)	31	2.4 (1.7, 3.5)
45-54	864	70.2 (67.4, 72.8)	321	27.5 (25.0, 30.3)	14	1.1 (0.6, 2.0)	13	1.1 (0.6, 2.0)	864	70.2 (67.4, 72.9)	310	26.7 (24.2, 29.4)	24	2.1 (1.4, 3.1)	13	1.0 (0.6, 1.8)
55-64	1262	68.7 (66.3, 70.9)	530	29.5 (27.3, 31.8)	19	1.1 (0.6, 1.7)	12	0.7 (0.4, 1.4)	1262	68.7 (66.3, 70.9)	523	29.1 (26.9, 31.4)	30	1.6 (1.1, 2.4)	8	0.5 (0.3, 1.2)
65-74	1147	63.7 (61.3, 66.0)	592	33.8 (31.5, 36.2)	30	1.8 (1.2, 2.6)	11	0.7 (0.4, 1.3)	1147	63.7 (61.3, 66.0)	587	33.6 (31.3, 35.9)	32	1.8 (1.3, 2.6)	14	0.9 (0.5, 1.7)
75+	556	58.2 (54.7, 61.6)	361	40.1 (36.8, 43.6)	10	1.2 (0.6, 2.4)	4	0.5 (0.2, 1.3)	556	58.4 (54.9, 61.8)	351	38.9 (35.6, 42.4)	16	2.2 (1.3, 3.7)	5	0.5 (0.2, 1.3)
**Gender**																
Male	2784	67.1 (65.4, 68.7)	1237	29.4 (27.8, 31.0)	72	2.0 (1.5, 2.6)	47	1.6 (1.2, 2.2)	2784	67.1 (65.5, 68.7)	1206	28.8 (27.3, 30.4)	98	2.5 (2.0, 3.1)	47	1.6 (1.1, 2.1)
Female	2973	68.6 (67.0, 70.1)	1209	28.4 (26.9, 29.9)	54	1.6 (1.2, 2.2)	46	1.4 (1.0, 2.0)	2973	68.6 (67.0, 70.2)	1179	27.8 (26.3, 29.3)	79	2.2 (1.7, 2.8)	47	1.4 (1.0, 2.0)
Other	60	54.7 (44.0, 65.0)	38	39.0 (29.0, 50.0)	5	2.8 (1.2, 6.7)	3	3.5 (1.0, 11.0)	60	54.7 (44.0, 65.0)	35	34.0 (24.7, 44.6)	4	6.0 (1.9, 17.3)	7	5.3 (2.3, 12.0)
**Race and ethnicity**																
White, non-Hispanic	4130	68.3 (67.0, 69.5)	1781	29.7 (28.5, 31.0)	74	1.2 (1.0, 1.6)	38	0.8 (0.5, 1.1)	4130	68.3 (67.0, 69.6)	1736	28.9 (27.7, 30.1)	107	1.9 (1.5, 2.3)	43	0.9 (0.7, 1.2)
Black, non-Hispanic	538	64.6 (60.8, 68.2)	235	28.6 (25.2, 32.3)	22	2.5 (1.6, 4.1)	28	4.2 (2.8, 6.3)	538	64.7 (60.9, 68.4)	232	27.8 (24.5, 31.5)	30	4.3 (2.9, 6.5)	23	3.1 (1.9, 4.9)
Hispanic, any race	726	67.0 (63.8, 70.0)	306	27.7 (24.9, 30.8)	24	2.6 (1.7, 3.9)	23	2.7 (1.7, 4.1)	726	66.9 (63.7, 69.9)	299	27.9 (25.0, 30.9)	30	2.8 (1.9, 4.1)	25	2.4 (1.6, 3.6)
Asian American / Pacific Islander	225	70.9 (64.7, 76.4)	75	24.3 (19.3, 30.1)	6	3.0 (1.1, 7.6)	6	1.8 (0.8, 4.2)	225	70.9 (64.7, 76.4)	76	25.0 (19.9, 31.0)	7	2.2 (1.0, 4.7)	4	1.9 (0.6, 5.9)
Other (American Indian/Alaskan, 2+ races, other)	231	61.8 (54.1, 69.0)	103	33.6 (26.9, 41.1)	7	4.2 (1.7, 10.2)	2	0.4 (0.1, 1.4)	231	61.9 (54.2, 69.1)	96	30.9 (24.4, 38.3)	9	3.4 (1.5, 7.5)	6	3.8 (1.4, 9.9)
**Education**																
No high school diploma or GED	313	58.3 (53.7, 62.8)	184	32.3 (28.1, 36.7)	26	5.3 (3.5, 8.1)	17	4.1 (2.4, 6.7)	313	58.4 (53.7, 62.9)	179	31.9 (27.8, 36.4)	25	5.0 (3.3, 7.4)	22	4.7 (3.0, 7.4)
High school graduate	1238	58.7 (56.3, 61.0)	824	37.3 (35.0, 39.6)	42	2.1 (1.5, 2.9)	33	1.9 (1.3, 2.8)	1238	58.8 (56.4, 61.1)	800	36.5 (34.2, 38.8)	64	3.1 (2.3, 4.1)	32	1.7 (1.2, 2.5)
Some college or Associates degree	1549	66.7 (64.6, 68.8)	746	30.7 (28.7, 32.8)	38	1.6 (1.1, 2.4)	22	0.9 (0.6, 1.5)	1549	66.8 (64.6, 68.8)	727	29.8 (27.8, 31.8)	53	2.3 (1.7, 3.1)	24	1.2 (0.7, 1.9)
Bachelors degree	1469	75.7 (73.6, 77.8)	442	22.1 (20.2, 24.2)	17	0.8 (0.5, 1.3)	18	1.3 (0.8, 2.1)	1469	75.7 (73.6, 77.7)	441	22.1 (20.1, 24.1)	20	1.1 (0.7, 1.9)	17	1.1 (0.7, 1.8)
Masters degree or higher	1281	80.4 (78.2, 82.4)	304	18.3 (16.3, 20.4)	10	0.6 (0.3, 1.2)	7	0.7 (0.3, 1.5)	1281	80.5 (78.3, 82.5)	292	17.6 (15.7, 19.7)	21	1.3 (0.8, 2.0)	6	0.6 (0.3, 1.4)
**Income**																
<$25,000	552	53.4 (49.9, 56.8)	393	38.9 (35.6, 42.4)	36	3.9 (2.7, 5.6)	31	3.8 (2.6, 5.5)	552	53.6 (50.1, 57.0)	374	37.2 (33.9, 40.7)	51	6.0 (4.4, 8.1)	31	3.2 (2.2, 4.6)
$25,000 - $49,999	885	60.5 (57.6, 63.4)	525	35.4 (32.6, 38.3)	36	2.7 (1.8, 3.9)	17	1.4 (0.8, 2.5)	885	60.6 (57.6, 63.4)	514	34.7 (31.9, 37.5)	40	2.8 (2.0, 3.8)	22	2.0 (1.2, 3.3)
$50,000 - $74,999	967	67.6 (64.7, 70.4)	416	30.0 (27.3, 32.9)	13	1.4 (0.7, 2.7)	10	1.0 (0.5, 2.0)	967	67.7 (64.8, 70.5)	405	29.6 (26.9, 32.5)	27	2.0 (1.3, 3.1)	6	0.7 (0.3, 1.7)
$75,000 - $99,999	824	68.1 (65.1, 71.0)	351	28.7 (25.9, 31.6)	23	1.9 (1.2, 2.9)	11	1.3 (0.7, 2.5)	824	68.1 (65.1, 71.0)	347	28.4 (25.6, 31.3)	23	2.0 (1.3, 3.2)	14	1.5 (0.8, 2.6)
$100,000 - $149,999	1083	72.3 (69.7, 74.8)	387	25.5 (23.1, 28.0)	10	0.8 (0.4, 1.6)	13	1.4 (0.8, 2.4)	1083	72.4 (69.7, 74.8)	376	24.8 (22.4, 27.3)	22	1.8 (1.1, 2.8)	11	1.1 (0.6, 2.0)
>$150,000	1539	76.7 (74.6, 78.7)	428	21.2 (19.3, 23.3)	15	1.0 (0.5, 1.9)	15	1.1 (0.6, 1.9)	1539	76.6 (74.5, 78.6)	423	21.0 (19.1, 23.0)	20	1.1 (0.6, 1.8)	17	1.3 (0.8, 2.3)
**Census Region**																
New England	285	70.8 (65.7, 75.4)	115	26.2 (21.8, 31.1)	6	1.5 (0.6, 3.4)	4	1.6 (0.5, 4.5)	285	70.8 (65.7, 75.4)	111	25.4 (21.0, 30.2)	10	2.9 (1.4, 5.6)	4	1.0 (0.3, 2.9)
Mid-Atlantic	735	68.2 (65.0, 71.2)	314	27.7 (24.8, 30.7)	21	2.5 (1.5, 4.2)	13	1.6 (0.9, 3.0)	735	68.4 (65.2, 71.4)	312	27.9 (25.0, 31.0)	29	2.9 (1.9, 4.3)	6	0.9 (0.4, 2.0)
East-North Central	861	68.9 (66.1, 71.6)	361	28.2 (25.6, 31.0)	20	1.5 (0.9, 2.4)	16	1.4 (0.8, 2.4)	861	69.1 (66.3, 71.8)	356	27.7 (25.1, 30.5)	25	2.2 (1.4, 3.3)	13	1.0 (0.6, 1.8)
West-North Central	389	62.3 (57.8, 66.6)	205	35.8 (31.5, 40.2)	6	1.5 (0.6, 3.7)	3	0.5 (0.2, 1.4)	389	62.3 (57.8, 66.6)	194	33.4 (29.2, 37.8)	13	3.2 (1.7, 5.9)	7	1.2 (0.5, 2.4)
South Atlantic	1147	66.8 (64.2, 69.3)	520	30.5 (28.1, 33.0)	15	1.1 (0.6, 2.1)	19	1.6 (1.0, 2.6)	1147	66.7 (64.1, 69.2)	505	29.3 (27.0, 31.8)	34	2.4 (1.6, 3.5)	18	1.6 (0.9, 2.7)
East-South Central	284	58.0 (52.8, 63.0)	163	37.5 (32.6, 42.7)	10	2.1 (1.1, 4.1)	7	2.3 (1.0, 5.5)	284	58.1 (52.8, 63.1)	158	36.9 (32.0, 42.1)	13	2.5 (1.4, 4.5)	7	2.5 (1.1, 5.6)
West-South Central	583	64.8 (61.2, 68.2)	287	31.0 (27.7, 34.5)	18	2.7 (1.5, 4.6)	11	1.5 (0.8, 2.8)	583	64.8 (61.2, 68.2)	282	30.6 (27.4, 34.1)	19	2.4 (1.4, 4.0)	14	2.2 (1.2, 4.0)
Mountain	524	69.9 (66.1, 73.5)	202	27.3 (23.8, 31.0)	14	2.2 (1.2, 3.9)	4	0.6 (0.2, 1.9)	524	70.0 (66.2, 73.6)	199	27.3 (23.9, 31.1)	13	1.6 (0.9, 3.0)	7	1.0 (0.5, 2.3)
Pacific	1042	72.3 (69.5, 75.0)	333	23.8 (21.4, 26.5)	23	1.7 (1.1, 2.6)	20	2.2 (1.3, 3.5)	1042	72.4 (69.6, 75.1)	322	23.3 (20.8, 26.0)	27	2.1 (1.4, 3.2)	25	2.2 (1.4, 3.4)

Table S11, continued

Characteristic	A member of the military or National Guard								A police officer							
	Not asked the question		Not willing		Somewhat willing		Very or completely willing		Not asked the question		Not willing		Somewhat willing		Very or completely willing	
	Unweighted n	Weighted %, 95% CI	Unweighted n	Weighted %, 95% CI	Unweighted n	Weighted %, 95% CI	Unweighted n	Weighted %, 95% CI	Unweighted n	Weighted %, 95% CI	Unweighted n	Weighted %, 95% CI	Unweighted n	Weighted %, 95% CI	Unweighted n	Weighted %, 95% CI
**Age, years**																
18-24	273	60.2 (55.2, 65.0)	142	31.9 (27.5, 36.7)	17	2.4 (3.9, 6.5)	14	3.9 (2.3, 6.6)	273	60.2 (55.2, 65.0)	139	31.2 (26.8, 36.0)	15	3.8 (2.2, 6.4)	19	4.8 (3.0, 7.5)
25-34	726	69.6 (66.4, 72.6)	228	23.1 (20.4, 26.1)	37	2.5 (3.6, 5.0)	29	3.7 (2.5, 5.5)	726	69.6 (66.4, 72.6)	218	22.4 (19.7, 25.4)	45	4.3 (3.1, 5.9)	32	3.7 (2.6, 5.3)
35-44	1022	73.9 (71.3, 76.3)	269	20.6 (18.4, 23.0)	47	2.5 (3.4, 4.5)	27	2.1 (1.4, 3.1)	1022	73.7 (71.2, 76.2)	270	20.7 (18.4, 23.1)	44	3.2 (2.4, 4.4)	32	2.4 (1.6, 3.4)
45-54	864	70.2 (67.4, 72.8)	314	26.9 (24.4, 29.7)	22	1.2 (1.9, 3.0)	12	1.0 (0.5, 1.8)	864	70.1 (67.3, 72.8)	305	26.3 (23.7, 29.0)	26	2.1 (1.4, 3.2)	18	1.5 (0.9, 2.4)
55-64	1262	68.7 (66.3, 70.9)	521	29.2 (26.9, 31.5)	27	0.9 (1.4, 2.1)	14	0.8 (0.4, 1.4)	1262	68.8 (66.4, 71.0)	518	28.9 (26.7, 31.2)	25	1.3 (0.8, 1.9)	18	1.1 (0.6, 1.8)
65-74	1147	63.7 (61.3, 66.0)	583	33.2 (30.9, 35.6)	32	1.5 (2.1, 3.1)	17	1.0 (0.6, 1.6)	1147	63.7 (61.3, 66.1)	581	33.1 (30.8, 35.4)	38	2.4 (1.7, 3.4)	13	0.8 (0.4, 1.4)
75+	556	58.4 (54.9, 61.7)	352	39.3 (35.9, 42.7)	16	1.2 (2.0, 3.4)	4	0.4 (0.1, 1.0)	556	58.2 (54.8, 61.6)	350	38.9 (35.6, 42.4)	16	2.0 (1.2, 3.4)	8	0.8 (0.4, 1.7)
**Gender**																
Male	2784	67.1 (65.4, 68.7)	1204	28.5 (27.0, 30.1)	97	2.1 (2.6, 3.2)	54	1.8 (1.3, 2.4)	2784	67.1 (65.5, 68.7)	1182	27.9 (26.4, 29.5)	108	3.0 (2.4, 3.7)	64	2.0 (1.5, 2.7)
Female	2973	68.6 (67.0, 70.1)	1160	27.3 (25.8, 28.8)	87	1.9 (2.4, 3.0)	56	1.7 (1.2, 2.3)	2973	68.5 (66.9, 70.1)	1157	27.3 (25.8, 28.8)	89	2.3 (1.8, 2.9)	64	1.9 (1.4, 2.5)
Other	60	54.7 (44.0, 65.0)	29	27.1 (18.8, 37.4)	11	4.9 (9.2, 16.5)	6	9.0 (3.7, 20.4)	60	54.7 (44.0, 65.0)	26	23.4 (15.7, 33.2)	9	9.3 (4.3, 19.1)	11	12.6 (6.7, 22.4)
**Race and ethnicity**																
White, non-Hispanic	4130	68.4 (67.1, 69.6)	1709	28.3 (27.1, 29.6)	116	1.8 (2.2, 2.7)	59	1.1 (0.8, 1.5)	4130	68.3 (67.0, 69.5)	1705	28.2 (26.9, 29.4)	116	2.1 (1.8, 2.6)	72	1.5 (1.1, 1.9)
Black, non-Hispanic	538	64.5 (60.6, 68.1)	229	26.9 (23.6, 30.4)	32	2.8 (4.1, 6.0)	26	4.6 (3.0, 7.0)	538	64.5 (60.7, 68.2)	219	26.5 (23.2, 30.0)	32	3.6 (2.4, 5.4)	35	5.4 (3.7, 7.8)
Hispanic, any race	726	66.9 (63.7, 69.9)	299	27.4 (24.6, 30.5)	34	2.2 (3.2, 4.5)	22	2.5 (1.6, 4.0)	726	67.0 (63.8, 70.0)	290	26.6 (23.7, 29.6)	43	4.3 (3.1, 5.9)	20	2.2 (1.4, 3.5)
Asian American / Pacific Islander	225	70.9 (64.7, 76.4)	77	25.5 (20.3, 31.5)	6	0.9 (2.4, 6.0)	4	1.2 (0.4, 3.3)	225	70.9 (64.7, 76.4)	72	23.8 (18.8, 29.7)	9	3.3 (1.5, 6.9)	6	2.0 (0.8, 4.9)
Other (American Indian/Alaskan, 2+ races, other)	231	61.9 (54.2, 69.1)	95	30.7 (24.2, 38.0)	10	1.3 (2.9, 6.3)	6	4.5 (1.8, 11.0)	231	62.3 (54.5, 69.4)	95	31.6 (24.9, 39.2)	9	2.9 (1.3, 6.3)	7	3.3 (1.4, 7.6)
**Education**																
No high school diploma or GED	313	58.4 (53.8, 62.9)	176	31.4 (27.3, 35.8)	25	2.9 (4.4, 6.5)	25	5.8 (3.8, 8.8)	313	58.3 (53.7, 62.8)	171	30.5 (26.4, 34.9)	32	5.9 (4.1, 8.5)	24	5.3 (3.4, 8.0)
High school graduate	1238	58.7 (56.3, 61.0)	794	35.7 (33.5, 38.0)	63	2.4 (3.2, 4.2)	39	2.4 (1.7, 3.4)	1238	58.7 (56.3, 61.0)	795	35.9 (33.7, 38.2)	59	2.8 (2.1, 3.7)	45	2.6 (1.9, 3.7)
Some college or Associates degree	1549	66.7 (64.6, 68.8)	719	29.3 (27.3, 31.4)	60	2.0 (2.7, 3.7)	26	1.2 (0.8, 1.8)	1549	66.7 (64.5, 68.8)	714	28.9 (26.9, 31.0)	56	2.6 (1.9, 3.5)	38	1.8 (1.3, 2.6)
Bachelors degree	1469	75.7 (73.6, 77.7)	424	21.2 (19.3, 23.2)	34	1.4 (2.0, 2.8)	20	1.1 (0.7, 1.8)	1469	75.8 (73.6, 77.8)	411	20.5 (18.6, 22.5)	41	2.3 (1.6, 3.1)	24	1.5 (1.0, 2.3)
Masters degree or higher	1281	80.5 (78.3, 82.5)	296	17.8 (15.9, 19.9)	16	0.7 (1.2, 2.0)	7	0.6 (0.2, 1.2)	1281	80.5 (78.3, 82.5)	290	17.2 (15.4, 19.3)	21	1.6 (1.0, 2.5)	9	0.7 (0.3, 1.4)
**Income**																
<$25,000	552	53.5 (50.0, 57.0)	372	36.4 (33.1, 39.8)	44	3.7 (5.1, 7.0)	40	4.9 (3.5, 6.9)	552	53.2 (49.8, 56.7)	371	36.6 (33.4, 40.1)	49	5.6 (4.1, 7.5)	42	4.5 (3.3, 6.3)
$25,000 - $49,999	885	60.6 (57.6, 63.4)	495	33.3 (30.6, 36.2)	59	3.1 (4.0, 5.2)	22	2.1 (1.2, 3.5)	885	60.6 (57.6, 63.4)	491	33.1 (30.3, 35.9)	57	3.9 (2.9, 5.1)	29	2.5 (1.6, 3.8)
$50,000 - $74,999	967	67.6 (64.7, 70.4)	398	28.7 (26.1, 31.5)	24	1.3 (2.0, 3.2)	17	1.6 (0.9, 2.9)	967	67.7 (64.8, 70.5)	392	28.1 (25.5, 30.9)	27	2.0 (1.3, 3.1)	20	2.2 (1.3, 3.7)
$75,000 - $99,999	824	68.2 (65.2, 71.0)	346	28.2 (25.5, 31.1)	28	1.8 (2.7, 4.0)	10	1.0 (0.5, 2.0)	824	68.1 (65.1, 71.0)	342	27.9 (25.2, 30.8)	25	2.1 (1.4, 3.2)	17	1.8 (1.1, 3.1)
$100,000 - $149,999	1083	72.3 (69.7, 74.8)	376	24.7 (22.3, 27.2)	22	1.2 (1.9, 2.9)	12	1.1 (0.6, 2.0)	1083	72.3 (69.7, 74.8)	373	24.4 (22.1, 26.9)	21	1.8 (1.1, 2.8)	16	1.5 (0.9, 2.5)
>$150,000	1539	76.6 (74.5, 78.6)	422	20.9 (19.1, 23.0)	21	0.7 (1.2, 2.0)	16	1.2 (0.7, 2.2)	1539	76.7 (74.5, 78.7)	412	20.2 (18.4, 22.2)	30	2.0 (1.3, 3.1)	16	1.1 (0.6, 1.9)
**Census Region**																
New England	285	70.9 (65.8, 75.6)	112	25.7 (21.3, 30.6)	7	0.7 (1.5, 3.3)	5	1.9 (0.7, 4.8)	285	70.9 (65.8, 75.5)	112	26.0 (21.6, 31.0)	6	1.3 (0.6, 3.1)	6	1.8 (0.8, 4.0)
Mid-Atlantic	735	68.2 (65.0, 71.2)	308	27.5 (24.7, 30.6)	27	2.0 (3.0, 4.5)	13	1.3 (0.7, 2.3)	735	68.2 (65.0, 71.2)	304	27.0 (24.2, 30.1)	28	3.0 (2.0, 4.4)	16	1.8 (1.0, 3.1)
East-North Central	861	69.1 (66.3, 71.8)	352	27.4 (24.8, 30.1)	21	1.1 (1.6, 2.6)	20	1.9 (1.2, 3.0)	861	69.0 (66.1, 71.7)	347	26.9 (24.3, 29.6)	30	2.2 (1.5, 3.3)	20	1.9 (1.2, 2.9)
West-North Central	389	62.5 (58.0, 66.8)	188	32.9 (28.8, 37.4)	16	1.8 (3.1, 5.4)	8	1.5 (0.6, 3.3)	389	62.2 (57.7, 66.5)	186	32.1 (28.0, 36.5)	18	3.5 (2.1, 5.8)	11	2.2 (1.1, 4.1)
South Atlantic	1147	66.7 (64.1, 69.2)	499	28.9 (26.5, 31.4)	42	1.9 (2.6, 3.6)	16	1.8 (1.0, 3.2)	1147	66.8 (64.2, 69.3)	492	28.8 (26.4, 31.2)	43	2.8 (2.0, 3.9)	19	1.6 (1.0, 2.7)
East-South Central	284	58.0 (52.8, 63.1)	157	35.5 (30.7, 40.6)	12	1.6 (2.9, 5.3)	11	3.5 (1.8, 6.8)	284	57.9 (52.7, 63.0)	158	36.4 (31.6, 41.6)	9	2.0 (1.0, 3.9)	13	3.6 (1.9, 6.7)
West-South Central	583	64.6 (61.0, 68.1)	282	30.1 (26.9, 33.6)	22	1.9 (3.1, 5.0)	13	2.1 (1.2, 3.7)	583	64.7 (61.0, 68.1)	281	29.6 (26.4, 33.0)	19	2.8 (1.7, 4.7)	17	3.0 (1.8, 4.9)
Mountain	524	70.1 (66.2, 73.7)	189	25.7 (22.3, 29.4)	18	1.7 (2.8, 4.7)	11	1.4 (0.7, 2.7)	524	70.0 (66.1, 73.5)	191	25.8 (22.4, 29.4)	18	2.8 (1.7, 4.7)	11	1.5 (0.8, 2.8)
Pacific	1042	72.4 (69.5, 75.0)	322	22.9 (20.4, 25.5)	33	1.9 (2.8, 4.0)	20	2.0 (1.2, 3.3)	1042	72.4 (69.6, 75.0)	310	21.9 (19.5, 24.5)	38	3.3 (2.3, 4.7)	27	2.5 (1.7, 3.8)

Table S11, continued

Characteristic	A person who does not share your race or ethnicity								A person who does not share your religion							
	Not asked the question		Not willing		Somewhat willing		Very or completely willing		Not asked the question		Not willing		Somewhat willing		Very or completely willing	
	Unweighted n	Weighted %, 95% CI	Unweighted n	Weighted %, 95% CI	Unweighted n	Weighted %, 95% CI	Unweighted n	Weighted %, 95% CI	Unweighted n	Weighted %, 95% CI	Unweighted n	Weighted %, 95% CI	Unweighted n	Weighted %, 95% CI	Unweighted n	Weighted %, 95% CI
**Age, years**																
18-24	273	60.2 (55.2, 65.0)	148	33.0 (28.5, 37.8)	16	4.3 (2.5, 7.1)	9	2.5 (1.3, 4.9)	273	60.2 (55.2, 65.0)	154	34.8 (30.3, 39.7)	8	2.1 (1.0, 4.3)	11	2.9 (1.6, 5.2)
25-34	726	69.6 (66.4, 72.6)	252	25.2 (22.4, 28.2)	23	2.5 (1.6, 3.9)	20	2.6 (1.6, 4.2)	726	69.7 (66.5, 72.7)	250	25.3 (22.5, 28.3)	19	2.4 (1.5, 3.9)	25	2.6 (1.7, 4.0)
35-44	1022	73.9 (71.3, 76.3)	288	22.0 (19.7, 24.4)	28	2.0 (1.4, 2.9)	27	2.1 (1.4, 3.2)	1022	74.1 (71.5, 76.5)	286	21.8 (19.5, 24.3)	33	2.4 (1.7, 3.4)	21	1.8 (1.1, 2.7)
45-54	864	70.1 (67.3, 72.8)	316	27.1 (24.6, 29.9)	16	1.4 (0.8, 2.3)	17	1.4 (0.8, 2.2)	864	70.3 (67.5, 72.9)	314	27.0 (24.5, 29.8)	19	1.6 (1.0, 2.5)	13	1.1 (0.6, 1.9)
55-64	1262	68.8 (66.5, 71.1)	532	29.4 (27.2, 31.7)	19	1.3 (0.8, 2.2)	8	0.5 (0.2, 1.0)	1262	68.8 (66.5, 71.1)	534	29.7 (27.5, 32.1)	15	1.0 (0.6, 1.7)	8	0.5 (0.2, 1.0)
65-74	1147	63.7 (61.3, 66.0)	598	34.1 (31.8, 36.5)	26	1.8 (1.2, 2.7)	7	0.4 (0.2, 0.9)	1147	63.5 (61.1, 65.9)	609	34.8 (32.5, 37.1)	17	1.1 (0.7, 1.8)	10	0.6 (0.3, 1.1)
75+	556	58.2 (54.8, 61.6)	357	39.4 (36.1, 42.8)	10	1.4 (0.7, 2.8)	7	1.0 (0.4, 2.1)	556	58.3 (54.9, 61.7)	359	39.9 (36.6, 43.4)	8	1.2 (0.5, 2.5)	6	0.6 (0.3, 1.4)
**Gender**																
Male	2784	67.1 (65.5, 68.7)	1237	29.2 (27.6, 30.8)	76	2.3 (1.8, 3.0)	41	1.4 (1.0, 2.0)	2784	67.2 (65.5, 68.8)	1240	29.5 (28.0, 31.1)	63	1.8 (1.4, 2.4)	46	1.5 (1.1, 2.0)
Female	2973	68.6 (67.0, 70.2)	1195	28.1 (26.6, 29.7)	59	1.7 (1.3, 2.3)	49	1.5 (1.1, 2.1)	2973	68.6 (67.0, 70.2)	1208	28.5 (27.0, 30.1)	52	1.5 (1.1, 2.1)	44	1.4 (1.0, 1.9)
Other	60	54.7 (44.0, 65.0)	40	38.2 (28.5, 48.9)	2	3.6 (0.7, 17.2)	4	3.5 (1.1, 10.6)	60	55.2 (44.4, 65.6)	39	38.7 (28.8, 49.6)	3	4.4 (1.3, 14.0)	3	1.8 (0.6, 5.5)
**Race and ethnicity**																
White, non-Hispanic	4130	68.3 (67.0, 69.6)	1781	29.7 (28.4, 30.9)	71	1.3 (1.0, 1.7)	36	0.7 (0.5, 1.0)	4130	68.3 (67.0, 69.6)	1789	29.8 (28.6, 31.1)	65	1.2 (0.9, 1.6)	34	0.7 (0.5, 1.0)
Black, non-Hispanic	538	64.7 (60.8, 68.3)	230	27.4 (24.1, 31.0)	29	4.3 (2.8, 6.5)	26	3.7 (2.4, 5.6)	538	64.7 (60.8, 68.3)	238	28.7 (25.3, 32.3)	19	2.6 (1.5, 4.3)	28	4.1 (2.7, 6.0)
Hispanic, any race	726	67.0 (63.8, 70.0)	305	27.7 (24.8, 30.7)	21	2.3 (1.5, 3.6)	27	3.0 (2.0, 4.5)	726	67.1 (64.0, 70.2)	298	27.3 (24.4, 30.3)	27	2.8 (1.9, 4.2)	25	2.8 (1.8, 4.2)
Asian American / Pacific Islander	225	70.9 (64.7, 76.4)	75	24.1 (19.1, 29.9)	9	4.2 (2.0, 8.8)	3	0.7 (0.2, 2.2)	225	71.1 (64.9, 76.6)	79	26.9 (21.5, 33.0)	4	1.3 (0.5, 3.7)	3	0.7 (0.2, 2.2)
Other (American Indian/Alaskan, 2+ races, other)	231	61.9 (54.2, 69.1)	100	32.8 (26.1, 40.3)	8	2.7 (1.2, 6.0)	3	2.6 (0.7, 9.6)	231	62.0 (54.3, 69.1)	102	34.0 (27.2, 41.5)	4	3.3 (1.0, 9.9)	4	0.7 (0.3, 2.1)
**Education**																
No high school diploma or GED	313	58.3 (53.7, 62.8)	186	32.3 (28.2, 36.8)	20	4.2 (2.6, 6.5)	21	5.2 (3.2, 8.2)	313	58.5 (53.9, 63.0)	182	32.1 (27.9, 36.5)	24	4.9 (3.2, 7.6)	19	4.5 (2.8, 7.2)
High school graduate	1238	58.6 (56.3, 61.0)	811	36.8 (34.5, 39.1)	57	3.0 (2.3, 4.1)	29	1.6 (1.1, 2.3)	1238	58.7 (56.3, 61.0)	822	37.4 (35.1, 39.7)	40	2.1 (1.5, 2.9)	34	1.8 (1.3, 2.6)
Some college or Associates degree	1549	66.8 (64.7, 68.9)	746	30.5 (28.4, 32.5)	33	1.6 (1.0, 2.4)	24	1.2 (0.8, 1.8)	1549	66.8 (64.7, 68.9)	754	31.1 (29.0, 33.2)	29	1.3 (0.9, 1.9)	18	0.7 (0.5, 1.2)
Bachelors degree	1469	75.7 (73.6, 77.7)	447	22.2 (20.3, 24.3)	18	1.1 (0.7, 1.8)	14	0.9 (0.5, 1.6)	1469	75.8 (73.7, 77.8)	445	22.3 (20.4, 24.4)	14	0.7 (0.4, 1.3)	17	1.1 (0.7, 1.9)
Masters degree or higher	1281	80.6 (78.4, 82.6)	301	18.0 (16.0, 20.0)	10	0.9 (0.5, 1.8)	7	0.6 (0.3, 1.3)	1281	80.4 (78.2, 82.4)	303	18.1 (16.2, 20.2)	12	1.0 (0.5, 1.8)	6	0.5 (0.2, 1.1)
**Income**																
<$25,000	552	53.3 (49.8, 56.8)	386	38.1 (34.8, 41.6)	40	4.6 (3.3, 6.4)	33	4.0 (2.7, 5.7)	552	53.6 (50.1, 57.1)	391	38.4 (35.0, 41.8)	35	4.3 (3.0, 6.1)	29	3.7 (2.5, 5.5)
$25,000 - $49,999	885	60.5 (57.6, 63.4)	523	34.8 (32.1, 37.7)	37	2.9 (2.0, 4.1)	17	1.8 (1.0, 3.1)	885	60.5 (57.6, 63.4)	531	35.7 (32.9, 38.5)	28	2.3 (1.5, 3.5)	19	1.5 (0.9, 2.6)
$50,000 - $74,999	967	67.7 (64.8, 70.5)	409	29.5 (26.9, 32.4)	20	1.7 (1.0, 2.9)	9	1.0 (0.5, 2.1)	967	67.6 (64.7, 70.4)	414	30.3 (27.6, 33.1)	17	1.3 (0.8, 2.3)	7	0.8 (0.3, 1.8)
$75,000 - $99,999	824	68.2 (65.2, 71.0)	355	29.0 (26.3, 32.0)	15	1.6 (0.9, 2.7)	13	1.2 (0.7, 2.2)	824	68.2 (65.2, 71.1)	357	29.3 (26.5, 32.2)	12	1.2 (0.7, 2.3)	13	1.2 (0.7, 2.3)
$100,000 - $149,999	1083	72.4 (69.8, 74.9)	383	25.3 (23.0, 27.9)	14	1.2 (0.7, 2.2)	11	1.0 (0.5, 1.8)	1083	72.3 (69.7, 74.8)	379	25.2 (22.8, 27.7)	15	1.1 (0.6, 1.8)	16	1.4 (0.8, 2.4)
>$150,000	1539	76.7 (74.6, 78.7)	435	21.3 (19.4, 23.3)	12	1.1 (0.5, 2.1)	12	0.9 (0.5, 1.7)	1539	76.8 (74.7, 78.8)	434	21.6 (19.6, 23.6)	12	0.9 (0.5, 1.7)	10	0.7 (0.4, 1.4)
**Census Region**																
New England	285	70.8 (65.7, 75.4)	117	26.9 (22.4, 31.9)	4	1.2 (0.4, 3.2)	4	1.1 (0.4, 3.1)	285	70.9 (65.8, 75.5)	116	26.6 (22.2, 31.5)	3	0.8 (0.2, 2.5)	5	1.8 (0.7, 4.7)
Mid-Atlantic	735	68.3 (65.1, 71.3)	318	28.2 (25.3, 31.2)	17	2.1 (1.2, 3.6)	13	1.5 (0.8, 2.6)	735	68.6 (65.4, 71.6)	318	28.7 (25.7, 31.8)	17	1.6 (1.0, 2.7)	8	1.1 (0.5, 2.4)
East-North Central	861	69.0 (66.1, 71.7)	355	27.6 (25.0, 30.3)	23	1.8 (1.1, 2.7)	18	1.7 (1.0, 2.8)	861	69.0 (66.2, 71.7)	358	27.8 (25.2, 30.5)	16	1.2 (0.7, 2.0)	22	2.0 (1.3, 3.1)
West-North Central	389	62.3 (57.8, 66.6)	197	32.9 (28.9, 37.2)	14	4.3 (2.4, 7.8)	3	0.5 (0.2, 1.4)	389	62.2 (57.7, 66.5)	201	34.9 (30.7, 39.4)	10	2.2 (1.1, 4.5)	4	0.7 (0.2, 1.7)
South Atlantic	1147	66.8 (64.2, 69.3)	517	30.1 (27.7, 32.6)	25	1.9 (1.2, 2.9)	12	1.2 (0.6, 2.3)	1147	66.7 (64.1, 69.2)	519	30.3 (27.9, 32.8)	21	1.7 (1.1, 2.8)	16	1.3 (0.8, 2.1)
East-South Central	284	58.1 (52.9, 63.2)	161	37.4 (32.5, 42.6)	10	1.9 (1.0, 3.7)	7	2.6 (1.1, 5.8)	284	58.1 (52.9, 63.2)	163	37.9 (33.0, 43.1)	8	2.0 (0.9, 4.1)	6	2.0 (0.8, 5.2)
West-South Central	583	64.8 (61.2, 68.3)	287	31.3 (28.0, 34.8)	12	1.9 (0.9, 3.7)	15	2.0 (1.2, 3.4)	583	64.9 (61.3, 68.3)	286	31.4 (28.0, 34.9)	15	2.1 (1.2, 3.6)	12	1.7 (0.9, 3.1)
Mountain	524	69.9 (66.0, 73.4)	207	28.3 (24.8, 32.1)	11	1.5 (0.8, 2.8)	3	0.4 (0.1, 1.1)	524	69.9 (66.1, 73.5)	210	28.9 (25.3, 32.7)	5	0.7 (0.3, 1.7)	5	0.5 (0.2, 1.2)
Pacific	1042	72.4 (69.6, 75.0)	332	23.3 (20.8, 25.9)	22	2.2 (1.4, 3.4)	20	2.2 (1.3, 3.6)	1042	72.4 (69.6, 75.0)	335	23.5 (21.1, 26.2)	24	2.4 (1.5, 3.7)	16	1.7 (1.0, 3.0)

Table S11, continued

Characteristic	A person who does not share your political beliefs							
	Not asked the question		Not willing		Somewhat willing		Very or completely willing	
	Unweighted n	Weighted %, 95% CI	Unweighted n	Weighted %, 95% CI	Unweighted n	Weighted %, 95% CI	Unweighted n	Weighted %, 95% CI
**Age, years**								
18-24	273	60.2 (55.2, 65.0)	144	32.8 (28.4, 37.7)	23	5.7 (3.6, 8.7)	6	1.3 (0.6, 2.9)
25-34	726	69.6 (66.4, 72.6)	245	24.9 (22.1, 27.9)	27	2.7 (1.8, 4.1)	23	2.8 (1.8, 4.4)
35-44	1022	73.9 (71.4, 76.4)	287	21.9 (19.6, 24.3)	30	2.2 (1.5, 3.2)	25	2.0 (1.3, 2.9)
45-54	864	70.2 (67.4, 72.8)	316	27.2 (24.6, 29.9)	18	1.5 (0.9, 2.4)	14	1.2 (0.7, 2.0)
55-64	1262	68.8 (66.5, 71.0)	523	29.2 (26.9, 31.5)	28	1.5 (1.0, 2.1)	9	0.6 (0.3, 1.2)
65-74	1147	63.6 (61.2, 65.9)	597	34.1 (31.8, 36.5)	27	1.6 (1.1, 2.4)	10	0.6 (0.3, 1.1)
75+	556	58.4 (54.9, 61.7)	357	39.5 (36.2, 43.0)	14	1.8 (1.0, 3.2)	2	0.3 (0.1, 1.4)
**Gender**								
Male	2784	67.1 (65.5, 68.7)	1213	28.9 (27.4, 30.5)	93	2.6 (2.0, 3.3)	48	1.4 (1.1, 1.9)
Female	2973	68.6 (67.0, 70.2)	1202	28.3 (26.8, 29.9)	66	1.9 (1.4, 2.4)	38	1.2 (0.8, 1.7)
Other	60	54.7 (44.0, 65.0)	36	33.6 (24.5, 44.1)	8	9.4 (4.1, 20.0)	2	2.4 (0.5, 10.5)
**Race and ethnicity**								
White, non-Hispanic	4130	68.3 (67.0, 69.6)	1745	29.0 (27.8, 30.3)	99	1.8 (1.4, 2.2)	44	0.9 (0.6, 1.2)
Black, non-Hispanic	538	64.7 (60.8, 68.3)	235	28.2 (24.8, 31.8)	31	4.2 (2.8, 6.4)	19	2.9 (1.8, 4.8)
Hispanic, any race	726	66.9 (63.7, 69.9)	310	28.3 (25.5, 31.4)	23	2.7 (1.7, 4.1)	22	2.1 (1.4, 3.2)
Asian American / Pacific Islander	225	71.1 (64.9, 76.6)	79	25.8 (20.6, 31.7)	4	2.4 (0.7, 7.3)	3	0.8 (0.2, 2.4)
Other (American Indian/Alaskan, 2+ races, other)	231	62.3 (54.6, 69.4)	100	32.2 (25.6, 39.7)	10	3.8 (1.9, 7.6)	1	1.7 (0.2, 10.8)
**Education**								
No high school diploma or GED	313	58.5 (53.8, 62.9)	189	33.6 (29.4, 38.1)	22	4.5 (2.9, 6.9)	15	3.5 (2.0, 6.0)
High school graduate	1238	58.8 (56.4, 61.1)	815	37.0 (34.7, 39.3)	48	2.6 (1.9, 3.7)	31	1.6 (1.1, 2.3)
Some college or Associates degree	1549	66.7 (64.5, 68.8)	741	30.1 (28.1, 32.2)	43	2.1 (1.4, 3.0)	23	1.1 (0.7, 1.7)
Bachelors degree	1469	75.7 (73.5, 77.7)	429	21.4 (19.5, 23.5)	34	1.9 (1.3, 2.7)	16	1.0 (0.6, 1.7)
Masters degree or higher	1281	80.6 (78.4, 82.6)	295	17.7 (15.7, 19.7)	20	1.4 (0.9, 2.2)	4	0.4 (0.1, 1.1)
**Income**								
<$25,000	552	53.6 (50.1, 57.0)	394	39.3 (36.0, 42.8)	36	4.3 (3.0, 6.1)	26	2.8 (1.9, 4.2)
$25,000 - $49,999	885	60.5 (57.6, 63.3)	526	35.0 (32.2, 37.8)	35	2.8 (1.9, 4.1)	17	1.7 (1.0, 3.1)
$50,000 - $74,999	967	67.6 (64.7, 70.4)	410	29.9 (27.1, 32.7)	21	1.8 (1.1, 2.9)	9	0.8 (0.4, 1.6)
$75,000 - $99,999	824	68.3 (65.3, 71.1)	342	28.1 (25.4, 31.0)	26	2.0 (1.3, 3.0)	15	1.6 (0.9, 2.8)
$100,000 - $149,999	1083	72.4 (69.8, 74.8)	376	24.8 (22.5, 27.4)	21	1.5 (1.0, 2.4)	12	1.2 (0.7, 2.2)
>$150,000	1539	76.6 (74.5, 78.6)	421	20.8 (18.9, 22.8)	28	2.1 (1.3, 3.2)	10	0.5 (0.3, 1.0)
**Census Region**								
New England	285	70.8 (65.7, 75.4)	115	26.3 (21.9, 31.2)	6	1.8 (0.8, 4.2)	4	1.1 (0.4, 3.1)
Mid-Atlantic	735	68.3 (65.2, 71.4)	322	29.1 (26.2, 32.2)	18	1.8 (1.1, 3.0)	7	0.7 (0.3, 1.6)
East-North Central	861	69.0 (66.2, 71.7)	359	27.9 (25.3, 30.6)	22	1.7 (1.1, 2.7)	15	1.3 (0.8, 2.3)
West-North Central	389	62.3 (57.8, 66.6)	195	33.0 (28.9, 37.3)	13	3.7 (1.9, 6.8)	6	1.1 (0.5, 2.4)
South Atlantic	1147	66.8 (64.2, 69.3)	506	29.6 (27.2, 32.1)	32	2.2 (1.5, 3.3)	15	1.4 (0.8, 2.4)
East-South Central	284	58.1 (52.9, 63.1)	164	38.1 (33.2, 43.3)	8	1.6 (0.8, 3.3)	6	2.2 (0.9, 5.5)
West-South Central	583	64.7 (61.1, 68.2)	281	30.4 (27.1, 33.8)	20	2.9 (1.7, 4.9)	15	2.0 (1.2, 3.4)
Mountain	524	69.9 (66.1, 73.5)	197	26.6 (23.2, 30.3)	19	3.0 (1.8, 4.9)	4	0.5 (0.2, 1.3)
Pacific	1042	72.3 (69.5, 75.0)	330	23.7 (21.2, 26.4)	29	2.4 (1.6, 3.7)	17	1.5 (0.9, 2.5)

Table S12. Variation with respondent characteristics in future likelihood of firearm possession and use in a situation where political violence is perceived as justified

Characteristic	I will be armed with a gun.						I will carry a gun openly, so that people know I am armed.					
	Not likely		Somewhat likely		Very or extremely likely		Not likely		Somewhat likely		Very or extremely likely	
	Unweighted n	Weighted %, 95% CI	Unweighted n	Weighted %, 95% CI	Unweighted n	Weighted %, 95% CI	Unweighted n	Weighted %, 95% CI	Unweighted n	Weighted %, 95% CI	Unweighted n	Weighted %, 95% CI
**Age, years**												
18-24	325	72.2 (67.3, 76.6)	66	16.4 (12.8, 20.6)	47	11.5 (8.6, 15.2)	373	83.6 (79.3, 87.2)	32	8.0 (5.6, 11.5)	33	8.3 (5.8, 11.8)
25-34	786	76.5 (73.5, 79.3)	135	13.8 (11.6, 16.3)	89	9.7 (7.8, 11.9)	899	87.7 (85.2, 89.8)	62	7.2 (5.5, 9.2)	47	5.1 (3.8, 6.9)
35-44	1080	79.3 (76.9, 81.5)	151	11.5 (9.8, 13.4)	123	9.2 (7.7, 11.0)	1217	89.2 (87.3, 90.9)	85	6.7 (5.4, 8.3)	51	4.1 (3.1, 5.4)
45-54	961	79.1 (76.6, 81.5)	128	10.7 (9.0, 12.7)	116	10.1 (8.4, 12.1)	1081	89.4 (87.4, 91.1)	66	5.3 (4.1, 6.8)	58	5.3 (4.0, 6.9)
55-64	1535	84.5 (82.6, 86.2)	179	10.1 (8.7, 11.7)	97	5.4 (4.4, 6.7)	1684	92.9 (91.5, 94.0)	84	4.7 (3.8, 5.9)	42	2.4 (1.7, 3.3)
65-74	1570	88.4 (86.7, 89.9)	127	7.5 (6.3, 8.9)	69	4.0 (3.2, 5.2)	1646	93.0 (91.6, 94.2)	82	4.8 (3.9, 6.0)	36	2.2 (1.6, 3.0)
75+	850	91.2 (89.0, 93.0)	47	5.3 (3.9, 7.2)	31	3.4 (2.4, 5.0)	879	94.8 (92.9, 96.1)	24	2.7 (1.8, 4.0)	22	2.6 (1.6, 4.0)
**Gender**												
Male	3337	78.8 (77.3, 80.3)	411	11.3 (10.1, 12.5)	354	9.9 (8.9, 11.1)	3715	88.9 (87.7, 90.1)	211	5.8 (5.0, 6.8)	171	5.2 (4.4, 6.2)
Female	3647	83.7 (82.3, 84.9)	404	10.5 (9.5, 11.7)	206	5.8 (5.0, 6.7)	3928	91.1 (90.0, 92.1)	214	5.7 (4.9, 6.6)	111	3.2 (2.6, 3.9)
Other	85	76.9 (65.8, 85.2)	13	14.7 (8.0, 25.4)	8	8.4 (3.9, 17.1)	95	91.0 (83.6, 95.2)	6	4.2 (1.8, 9.3)	5	4.8 (1.8, 12.0)
**Race and ethnicity**												
White, non-Hispanic	5073	83.1 (82.0, 84.1)	525	9.6 (8.8, 10.5)	372	7.3 (6.6, 8.1)	5499	91.0 (90.1, 91.8)	285	5.3 (4.7, 5.9)	183	3.7 (3.2, 4.4)
Black, non-Hispanic	636	74.4 (70.7, 77.7)	105	14.0 (11.4, 17.1)	83	11.6 (9.2, 14.5)	711	83.8 (80.5, 86.7)	65	9.1 (7.0, 11.8)	47	7.1 (5.1, 9.7)
Hispanic, any race	872	79.9 (77.1, 82.5)	132	12.8 (10.7, 15.3)	70	7.2 (5.7, 9.2)	991	91.7 (89.5, 93.4)	46	4.6 (3.3, 6.3)	34	3.7 (2.6, 5.3)
Asian American / Pacific Islander	265	85.0 (79.5, 89.3)	30	10.3 (6.9, 15.2)	11	4.7 (2.4, 8.9)	284	91.2 (86.0, 94.6)	15	6.0 (3.3, 10.6)	6	2.8 (1.1, 6.9)
Other (American Indian/Alaskan, 2+ races, other)	261	71.5 (63.7, 78.2)	41	17.1 (11.6, 24.4)	36	11.4 (7.3, 17.5)	294	83.7 (76.9, 88.7)	24	9.6 (5.6, 15.9)	19	6.8 (4.0, 11.3)
**Education**												
No high school diploma or GED	409	76.0 (71.6, 79.8)	57	11.3 (8.6, 14.7)	65	12.8 (9.9, 16.4)	446	83.0 (79.0, 86.4)	32	6.8 (4.6, 9.8)	52	10.3 (7.7, 13.6)
High school graduate	1673	76.3 (74.1, 78.4)	265	14.1 (12.4, 16.0)	179	9.6 (8.2, 11.2)	1854	85.9 (84.0, 87.6)	151	8.1 (6.8, 9.6)	107	6.0 (4.8, 7.4)
Some college or Associates degree	1893	79.3 (77.4, 81.2)	261	12.2 (10.8, 13.8)	179	8.4 (7.2, 9.8)	2107	90.1 (88.6, 91.3)	140	6.2 (5.2, 7.4)	83	3.8 (3.0, 4.8)
Bachelors degree	1691	86.5 (84.7, 88.2)	161	8.7 (7.4, 10.2)	86	4.8 (3.8, 5.9)	1847	94.8 (93.5, 95.9)	64	3.6 (2.8, 4.8)	26	1.6 (1.0, 2.3)
Masters degree or higher	1441	90.0 (88.3, 91.5)	89	5.8 (4.6, 7.2)	63	4.2 (3.3, 5.5)	1525	95.7 (94.5, 96.6)	48	3.0 (2.2, 4.0)	21	1.4 (0.9, 2.1)
**Income**												
<$25,000	772	75.6 (72.5, 78.5)	137	14.7 (12.4, 17.4)	94	9.7 (7.8, 11.9)	845	83.0 (80.2, 85.5)	81	8.7 (6.9, 10.9)	74	8.3 (6.5, 10.5)
$25,000 - $49,999	1155	76.6 (73.8, 79.1)	174	13.1 (11.2, 15.4)	124	10.3 (8.5, 12.4)	1297	87.3 (85.0, 89.3)	86	7.2 (5.6, 9.1)	68	5.6 (4.3, 7.2)
$50,000 - $74,999	1145	79.5 (76.8, 82.0)	154	12.5 (10.5, 14.8)	97	8.0 (6.4, 10.0)	1261	88.7 (86.4, 90.7)	86	7.1 (5.6, 9.0)	47	4.2 (3.0, 5.9)
$75,000 - $99,999	1003	81.1 (78.3, 83.6)	112	11.1 (9.2, 13.5)	80	7.8 (6.1, 9.8)	1100	90.8 (88.7, 92.5)	56	5.4 (4.1, 7.1)	39	3.7 (2.7, 5.2)
$100,000 - $149,999	1277	84.7 (82.4, 86.7)	119	8.4 (7.0, 10.2)	86	6.9 (5.5, 8.6)	1385	92.6 (90.9, 94.1)	69	4.7 (3.6, 6.0)	28	2.7 (1.8, 4.1)
>$150,000	1755	86.5 (84.6, 88.2)	137	8.0 (6.7, 9.5)	91	5.5 (4.4, 6.8)	1891	94.5 (93.1, 95.6)	57	3.2 (2.4, 4.3)	33	2.3 (1.6, 3.3)
**Census Region**												
New England	357	86.3 (81.8, 89.8)	33	9.2 (6.4, 13.0)	18	4.6 (2.6, 7.9)	386	93.2 (89.4, 95.7)	15	4.4 (2.5, 7.4)	7	2.4 (1.0, 5.8)
Mid-Atlantic	941	85.3 (82.5, 87.8)	84	8.6 (6.8, 10.8)	48	6.0 (4.4, 8.2)	1005	92.3 (89.9, 94.2)	45	4.7 (3.4, 6.6)	22	2.9 (1.8, 4.9)
East-North Central	1033	81.0 (78.3, 83.4)	132	12.2 (10.2, 14.6)	87	6.8 (5.4, 8.6)	1143	89.8 (87.6, 91.7)	64	5.9 (4.6, 7.7)	44	4.2 (3.0, 5.9)
West-North Central	507	82.3 (78.4, 85.6)	50	9.6 (7.1, 13.0)	43	8.1 (5.9, 10.9)	548	89.5 (86.1, 92.2)	28	6.0 (3.9, 9.1)	23	4.5 (2.9, 6.8)
South Atlantic	1405	80.2 (77.8, 82.3)	170	11.2 (9.6, 13.1)	120	8.6 (7.1, 10.4)	1528	88.5 (86.5, 90.3)	98	6.7 (5.4, 8.3)	65	4.8 (3.7, 6.2)
East-South Central	342	72.9 (68.0, 77.4)	60	14.1 (10.7, 18.3)	54	12.9 (9.8, 16.9)	396	85.5 (81.3, 88.9)	32	6.3 (4.3, 9.1)	29	8.2 (5.6, 11.9)
West-South Central	703	77.4 (74.1, 80.4)	100	11.8 (9.5, 14.4)	88	10.9 (8.7, 13.5)	784	86.9 (84.1, 89.3)	58	7.1 (5.3, 9.4)	49	6.0 (4.4, 8.1)
Mountain	593	78.0 (74.3, 81.3)	87	13.1 (10.5, 16.3)	56	8.8 (6.7, 11.6)	664	89.5 (86.8, 91.8)	49	6.9 (5.1, 9.2)	24	3.6 (2.3, 5.5)
Pacific	1226	85.2 (82.8, 87.3)	117	9.6 (7.9, 11.7)	58	5.2 (3.9, 6.9)	1325	93.8 (92.0, 95.2)	46	3.9 (2.8, 5.4)	26	2.3 (1.4, 3.5)

Table S12, continued

Characteristic	I will threaten someone with a gun.						I will shoot someone with a gun.					
	Not likely		Somewhat likely		Very or extremely likely		Not likely		Somewhat likely		Very or extremely likely	
	Unweighted n	Weighted %, 95% CI	Unweighted n	Weighted %, 95% CI	Unweighted n	Weighted %, 95% CI	Unweighted n	Weighted %, 95% CI	Unweighted n	Weighted %, 95% CI	Unweighted n	Weighted %, 95% CI
**Age, years**												
18-24	422	95.8 (93.1, 97.5)	5	1.4 (0.6, 3.3)	10	2.8 (1.5, 5.2)	415	93.6 (90.4, 95.8)	15	4.2 (2.5, 7.0)	8	2.3 (1.1, 4.6)
25-34	977	96.5 (94.9, 97.6)	15	1.9 (1.1, 3.2)	16	1.6 (1.0, 2.7)	964	94.6 (92.7, 96.1)	32	4.0 (2.8, 5.8)	13	1.4 (0.8, 2.4)
35-44	1311	96.5 (95.2, 97.4)	32	2.6 (1.8, 3.7)	11	0.9 (0.5, 1.7)	1294	95.5 (94.2, 96.5)	39	3.1 (2.2, 4.3)	19	1.4 (0.9, 2.2)
45-54	1182	98.0 (96.9, 98.7)	12	1.1 (0.6, 1.9)	11	1.0 (0.5, 1.7)	1165	96.5 (95.2, 97.5)	29	2.5 (1.7, 3.6)	11	1.0 (0.5, 1.8)
55-64	1788	99.0 (98.3, 99.4)	10	0.7 (0.3, 1.3)	8	0.4 (0.2, 0.8)	1761	97.1 (96.1, 97.9)	34	1.9 (1.3, 2.7)	16	1.0 (0.6, 1.7)
65-74	1753	99.0 (98.4, 99.4)	15	0.9 (0.5, 1.5)	2	0.1 (0.0, 0.4)	1728	97.3 (96.3, 98.1)	34	2.3 (1.6, 3.2)	6	0.4 (0.2, 0.8)
75+	918	98.9 (97.6, 99.5)	4	0.5 (0.2, 1.4)	3	0.6 (0.2, 1.8)	908	97.9 (96.5, 98.7)	15	1.7 (1.0, 2.9)	3	0.4 (0.1, 1.4)
**Gender**												
Male	4028	97.5 (96.8, 98.1)	35	1.2 (0.8, 1.7)	34	1.3 (0.9, 1.8)	3952	95.2 (94.3, 96.0)	106	3.3 (2.7, 4.1)	43	1.5 (1.1, 2.1)
Female	4177	97.8 (97.2, 98.3)	54	1.5 (1.1, 2.0)	25	0.7 (0.5, 1.1)	4137	96.8 (96.1, 97.4)	87	2.4 (1.9, 3.0)	31	0.8 (0.5, 1.1)
Other	100	95.0 (88.0, 98.0)	4	2.2 (0.8, 5.8)	2	2.8 (0.7, 11.1)	100	94.9 (87.9, 97.9)	4	2.8 (1.0, 7.7)	2	2.3 (0.4, 10.6)
**Race and ethnicity**												
White, non-Hispanic	5897	98.6 (98.2, 98.9)	40	0.7 (0.5, 1.0)	29	0.7 (0.4, 1.0)	5848	97.6 (97.1, 98.0)	93	1.8 (1.4, 2.2)	29	0.6 (0.4, 0.9)
Black, non-Hispanic	787	95.0 (93.0, 96.5)	19	2.8 (1.7, 4.5)	16	2.2 (1.3, 3.7)	749	90.4 (87.8, 92.6)	47	6.4 (4.6, 8.7)	25	3.2 (2.1, 5.0)
Hispanic, any race	1039	96.4 (94.8, 97.4)	20	1.9 (1.2, 3.1)	14	1.7 (1.0, 3.0)	1020	94.6 (92.9, 96.0)	37	3.6 (2.5, 5.1)	16	1.8 (1.0, 2.9)
Asian American / Pacific Islander	298	98.0 (95.7, 99.1)	5	1.3 (0.5, 3.4)	2	0.7 (0.2, 2.6)	296	97.0 (94.2, 98.5)	10	3.0 (1.5, 5.8)	0	0 (N/A)
Other (American Indian/Alaskan, 2+ races, other)	330	94.2 (87.8, 97.4)	9	5.8 (2.6, 12.2)	0	0 (N/A)	322	91.6 (84.9, 95.5)	11	6.4 (3.0, 13.3)	6	1.9 (0.7, 5.0)
**Education**												
No high school diploma or GED	502	93.8 (90.9, 95.9)	14	3.0 (1.6, 5.3)	14	3.2 (1.8, 5.6)	487	91.7 (88.7, 94.0)	28	4.9 (3.2, 7.4)	16	3.4 (2.0, 5.7)
High school graduate	2061	97.0 (95.9, 97.7)	31	1.8 (1.3, 2.7)	20	1.2 (0.8, 1.9)	2018	94.1 (92.7, 95.3)	71	4.3 (3.3, 5.5)	27	1.6 (1.1, 2.4)
Some college or Associates degree	2289	97.9 (97.1, 98.5)	25	1.2 (0.8, 1.8)	19	0.9 (0.6, 1.5)	2253	96.5 (95.6, 97.2)	62	2.8 (2.1, 3.6)	19	0.7 (0.4, 1.1)
Bachelors degree	1912	98.6 (97.8, 99.1)	16	0.9 (0.5, 1.5)	8	0.5 (0.3, 1.2)	1902	97.9 (97.0, 98.6)	25	1.5 (0.9, 2.2)	10	0.6 (0.3, 1.2)
Masters degree or higher	1587	99.4 (98.7, 99.7)	7	0.6 (0.3, 1.3)	0	0 (N/A)	1575	98.9 (98.1, 99.3)	12	0.9 (0.5, 1.7)	4	0.2 (0.1, 0.6)
**Income**												
<$25,000	955	94.2 (92.1, 95.7)	26	3.3 (2.1, 5.0)	22	2.5 (1.6, 4.0)	932	92.0 (89.8, 93.7)	46	5.1 (3.7, 6.9)	26	3.0 (1.9, 4.5)
$25,000 - $49,999	1410	96.7 (95.3, 97.7)	26	1.9 (1.2, 3.1)	16	1.4 (0.8, 2.3)	1387	95.0 (93.4, 96.2)	47	3.6 (2.6, 5.1)	18	1.4 (0.8, 2.3)
$50,000 - $74,999	1378	98.7 (97.8, 99.3)	6	0.6 (0.2, 1.3)	8	0.7 (0.3, 1.5)	1354	96.6 (95.3, 97.6)	36	2.9 (2.0, 4.2)	6	0.4 (0.2, 1.1)
$75,000 - $99,999	1174	98.0 (96.7, 98.8)	11	1.1 (0.6, 2.1)	7	0.9 (0.4, 1.9)	1157	95.6 (93.7, 97.0)	24	3.0 (1.9, 4.8)	13	1.4 (0.7, 2.5)
$100,000 - $149,999	1465	98.2 (97.1, 98.9)	13	1.1 (0.6, 1.9)	5	0.7 (0.3, 1.7)	1453	97.2 (95.8, 98.1)	24	2.2 (1.4, 3.4)	5	0.6 (0.2, 1.6)
>$150,000	1969	98.8 (97.9, 99.3)	11	0.8 (0.4, 1.5)	3	0.4 (0.1, 1.3)	1952	98.0 (97.1, 98.7)	21	1.4 (0.9, 2.1)	8	0.6 (0.3, 1.4)
**Census Region**												
New England	406	99.8 (98.5, 100.0)	1	0.2 (0.0, 1.5)	0	0 (N/A)	404	98.8 (96.8, 99.5)	5	1.2 (0.5, 3.2)	0	0 (N/A)
Mid-Atlantic	1050	97.3 (95.7, 98.3)	14	1.8 (1.0, 3.2)	7	0.9 (0.4, 1.9)	1043	96.5 (94.8, 97.7)	21	2.5 (1.5, 4.0)	8	1.0 (0.5, 2.1)
East-North Central	1229	97.7 (96.5, 98.5)	13	1.2 (0.7, 2.2)	11	1.1 (0.6, 2.0)	1208	95.9 (94.2, 97.1)	29	2.9 (1.9, 4.5)	13	1.1 (0.6, 2.0)
West-North Central	592	98.8 (97.3, 99.5)	1	0.3 (0.0, 2.1)	5	0.9 (0.4, 2.2)	586	97.3 (95.2, 98.4)	9	1.6 (0.8, 3.3)	5	1.1 (0.4, 2.8)
South Atlantic	1667	98.1 (97.0, 98.8)	16	1.3 (0.7, 2.3)	9	0.7 (0.3, 1.3)	1653	97.1 (95.9, 98.0)	30	2.1 (1.4, 3.2)	10	0.8 (0.4, 1.5)
East-South Central	442	96.0 (93.4, 97.6)	8	1.9 (0.9, 4.0)	8	2.0 (1.0, 4.2)	429	93.1 (89.8, 95.3)	22	5.5 (3.5, 8.6)	6	1.4 (0.6, 3.2)
West-South Central	864	95.9 (93.9, 97.2)	17	2.3 (1.4, 4.0)	11	1.8 (1.0, 3.3)	840	92.6 (90.2, 94.5)	34	4.9 (3.4, 7.0)	17	2.5 (1.4, 4.2)
Mountain	727	99.1 (97.9, 99.6)	5	0.7 (0.3, 1.8)	2	0.2 (0.1, 0.9)	716	96.7 (94.7, 97.9)	15	2.5 (1.4, 4.4)	6	0.8 (0.3, 1.9)
Pacific	1374	97.4 (95.9, 98.3)	18	1.4 (0.9, 2.3)	8	1.2 (0.6, 2.6)	1356	96.4 (95.0, 97.4)	33	2.6 (1.8, 3.7)	11	1.0 (0.5, 2.1)

Figure S1. Observed and expected monthly counts of National Instant Criminal Background Check System background checks for firearm purchases, January 2014 to July 2023

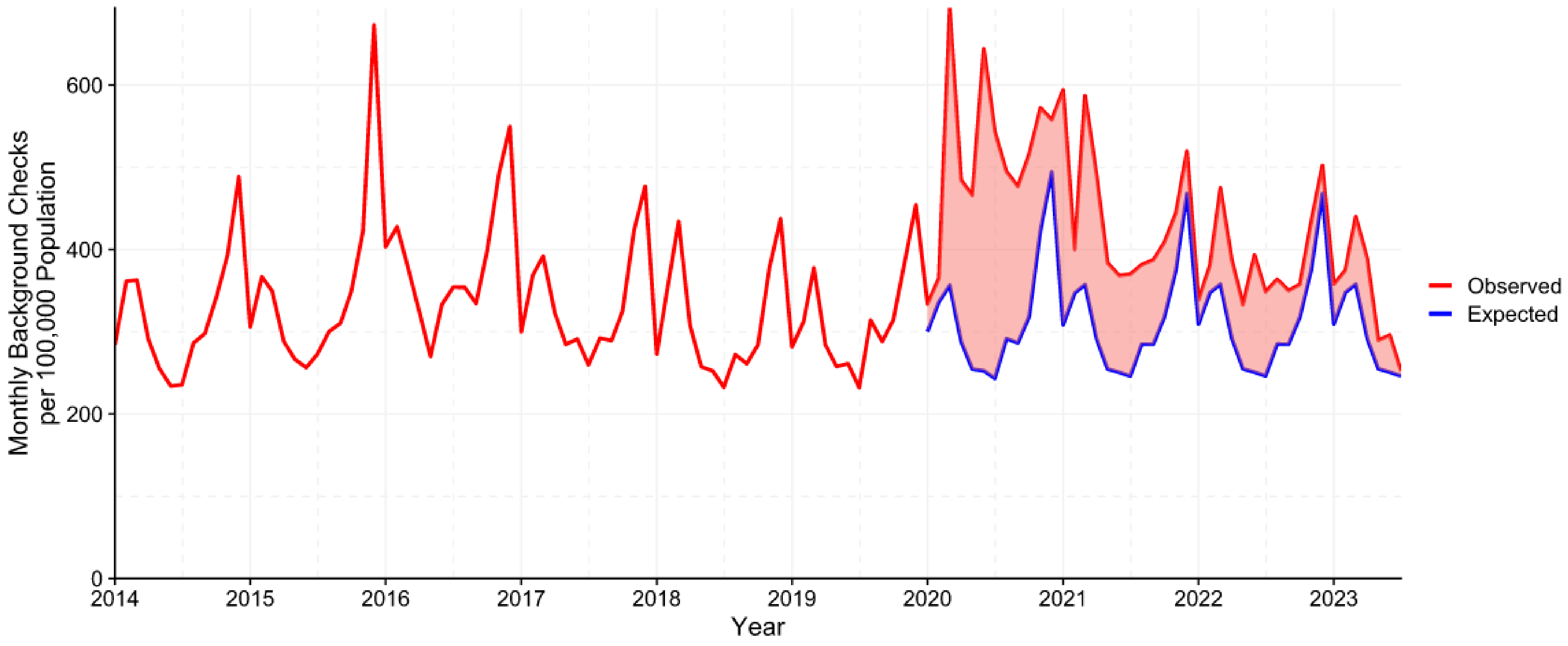

Expected counts for 2020 to 2023 were obtained by fitting an ARIMA model to data for January 2007 through December 2019.

Figure S2. Association between respondents' age, gender, education, and income and their views of democracy and society and support for political violence in the United States

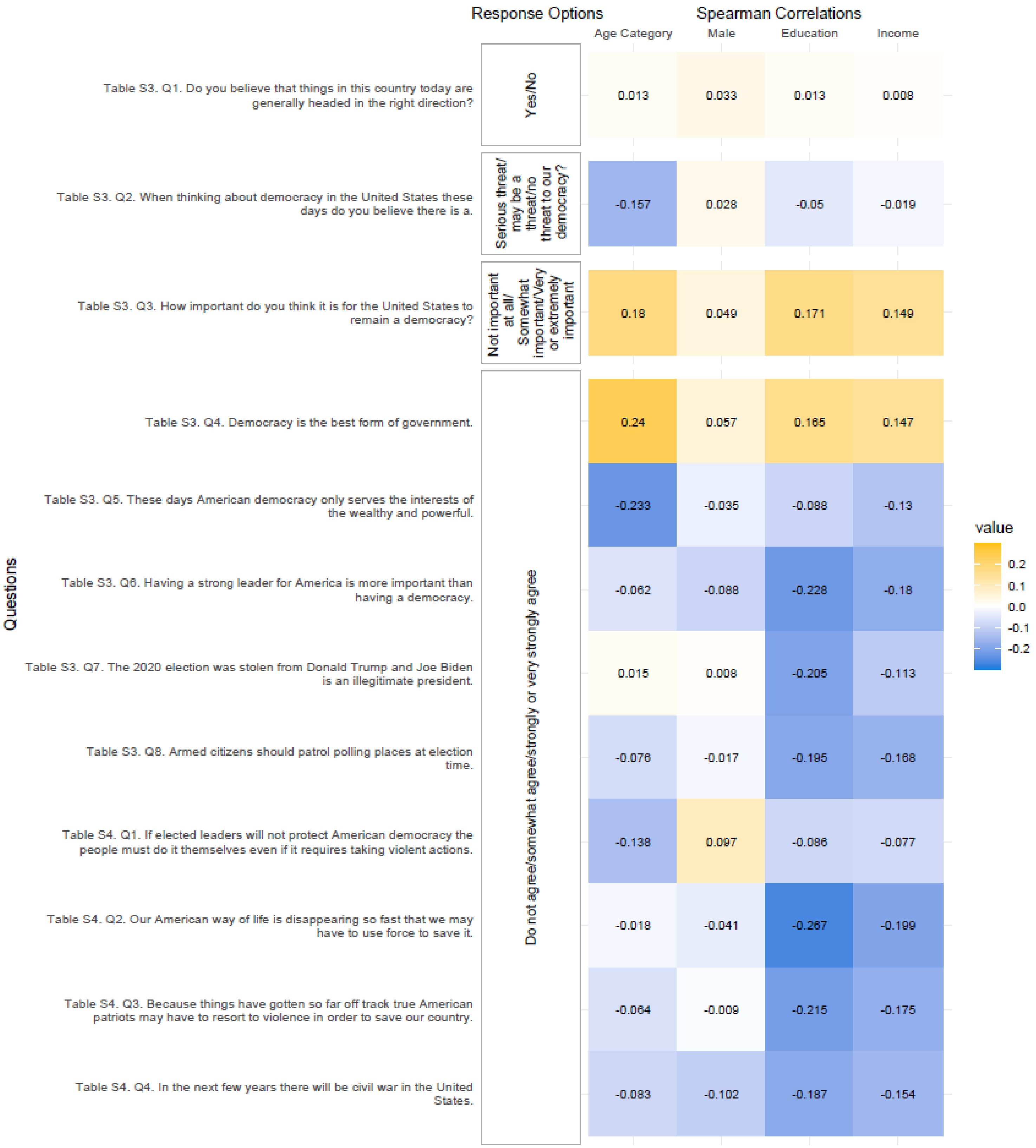

Figure S2, continued

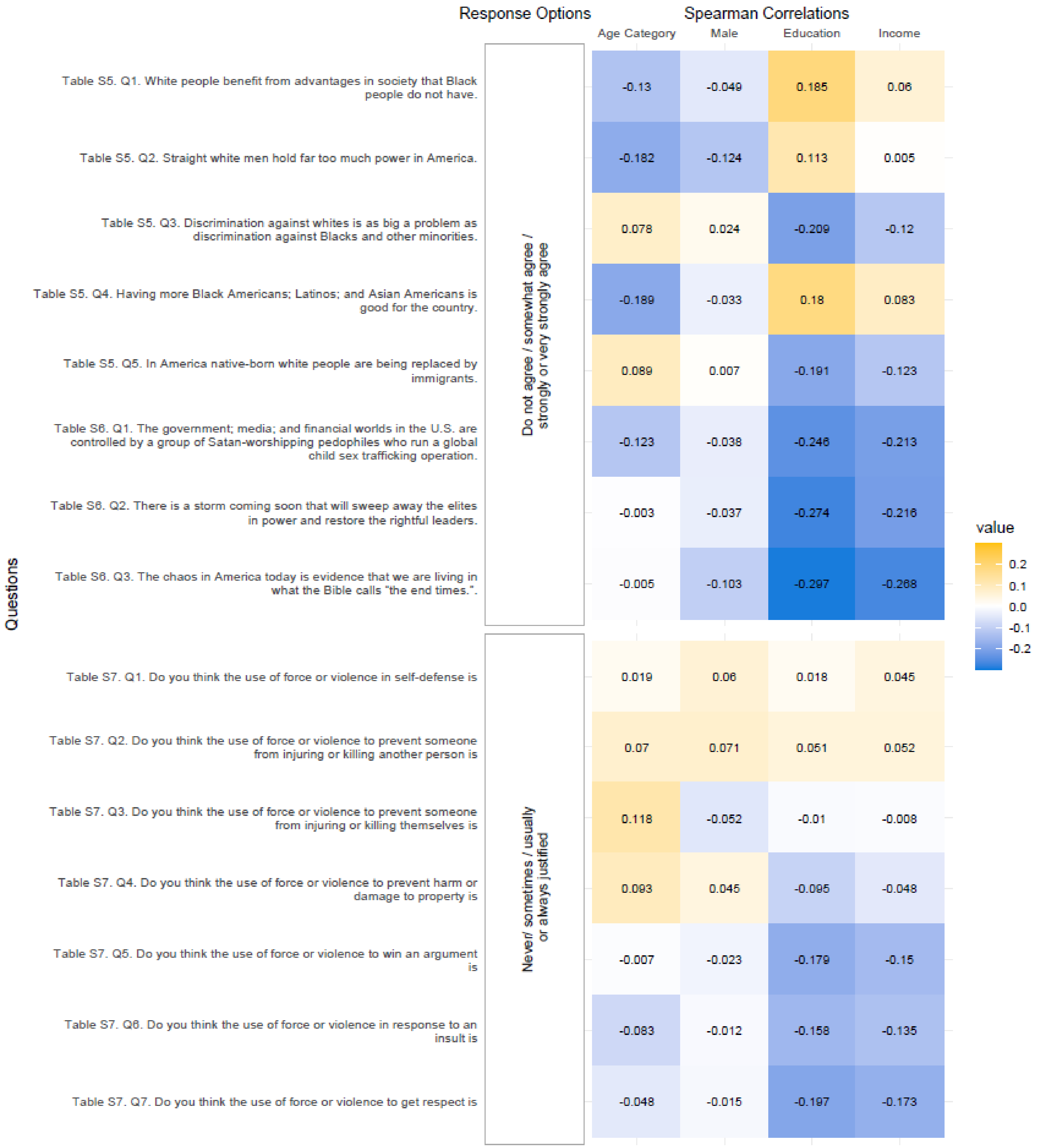

Figure S2, continued

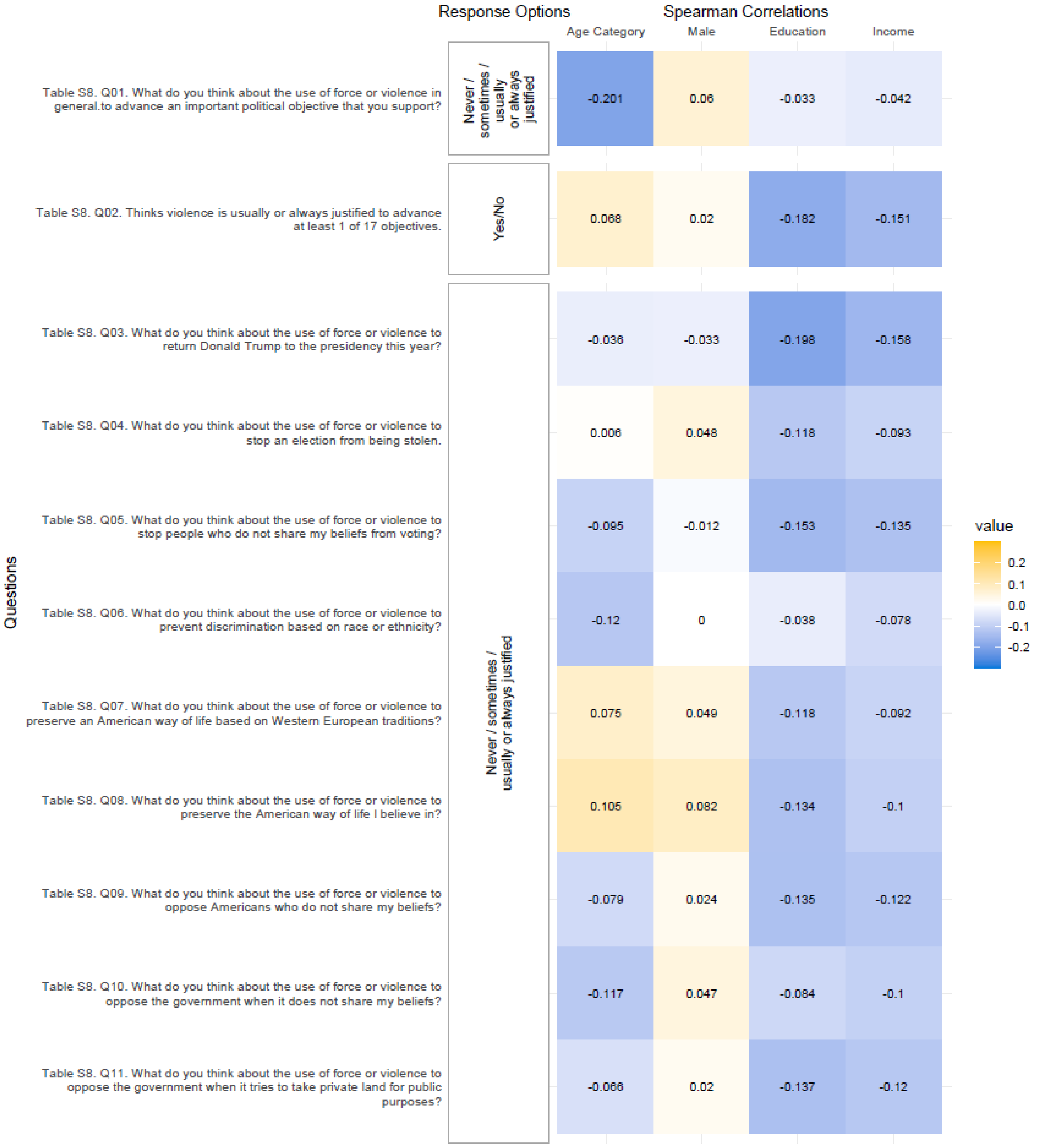

Figure S2, continued

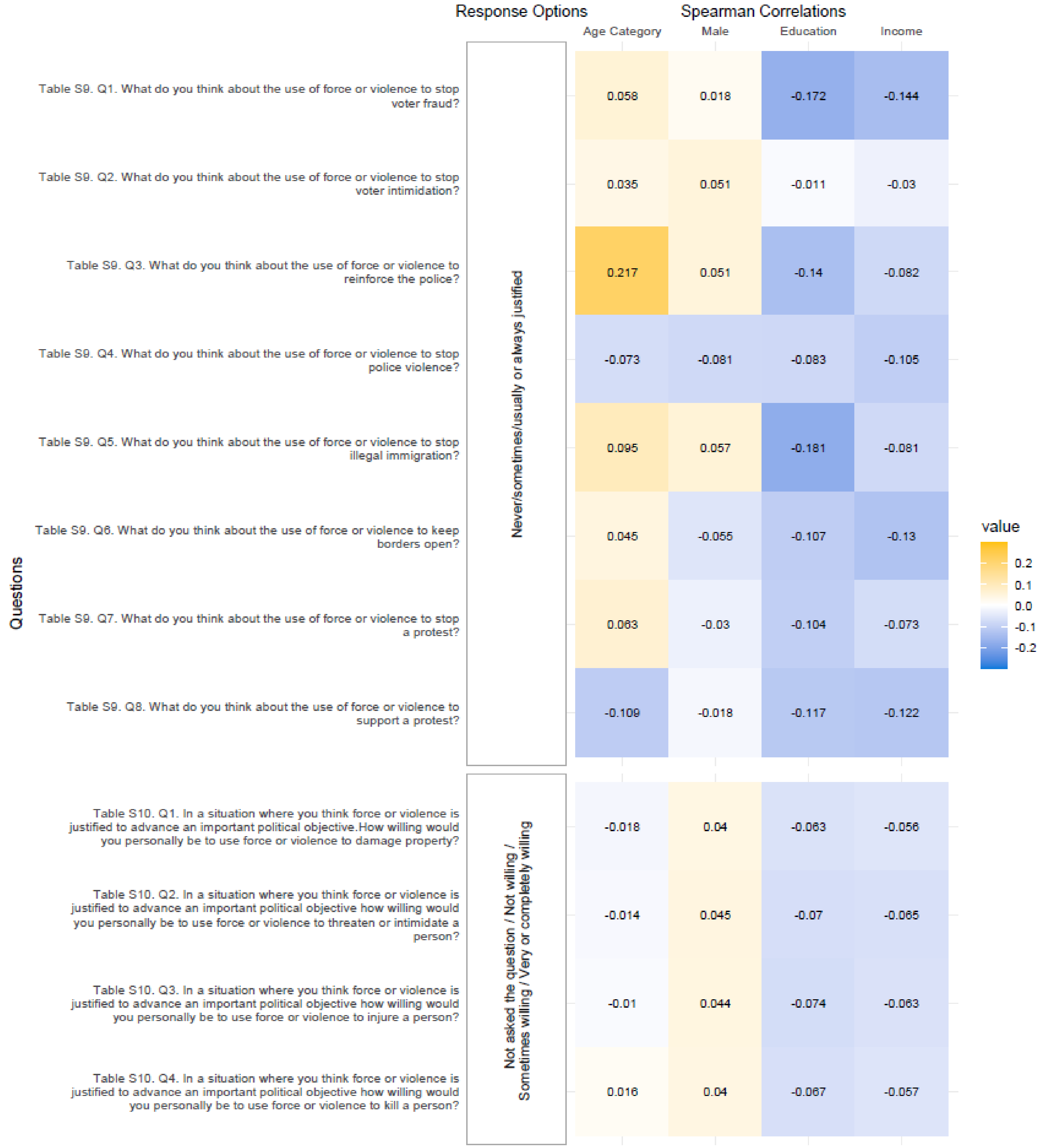

Figure S2, continued

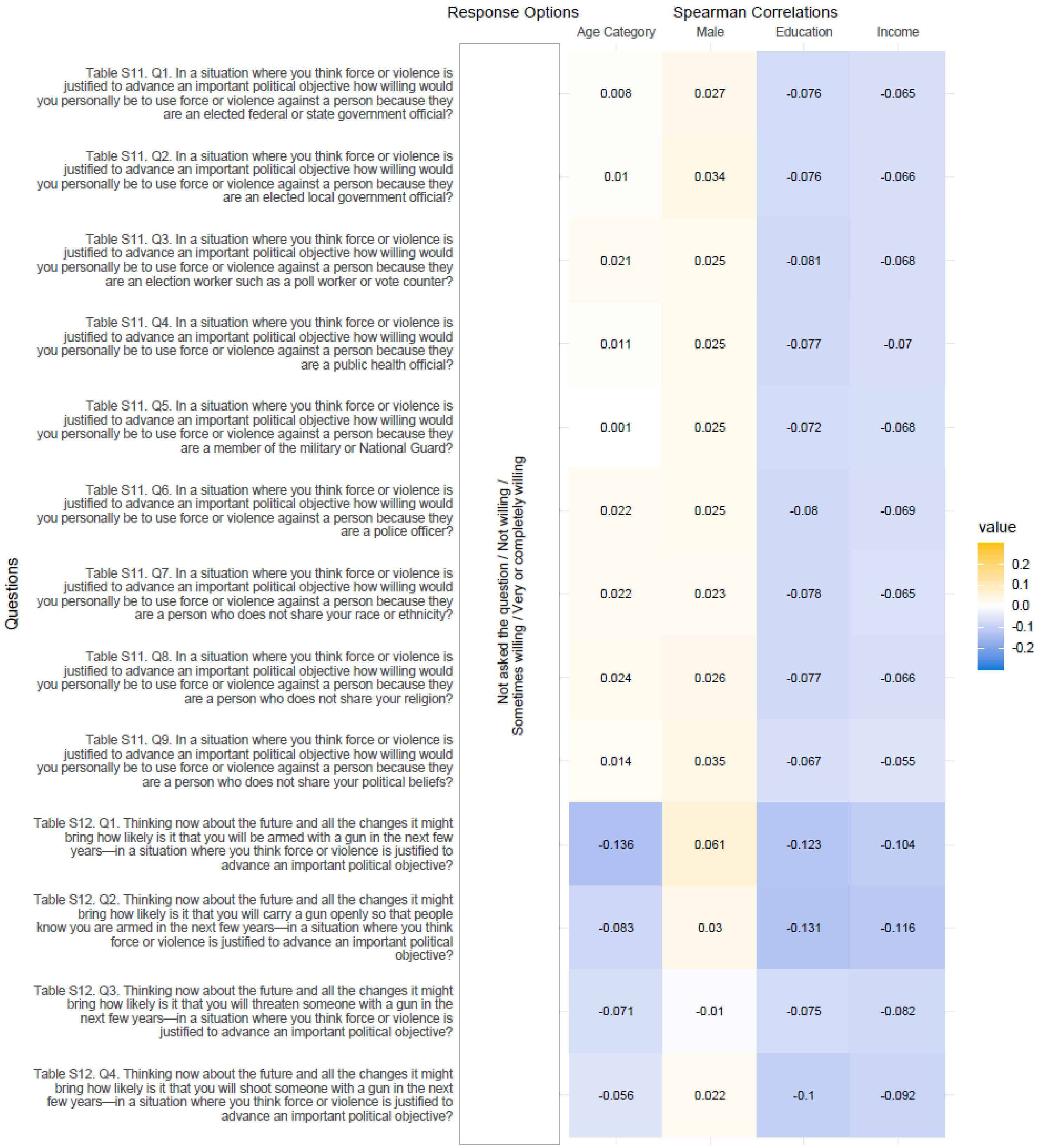

Supplement: Supplementary file 1 — Additional file 1. Supplemental materials. [file 40621_2023_456_MOESM1_ESM.pdf]
